# Evaluating the impact of calibration of patient-reported outcomes measures on results from randomized clinical trials: a simulation study based on Rasch measurement theory

**DOI:** 10.1186/s12874-022-01680-z

**Published:** 2022-08-12

**Authors:** Angély Loubert, Antoine Regnault, Véronique Sébille, Jean-Benoit Hardouin

**Affiliations:** 1 Modus Outcomes, a division of THREAD, Lyon, France; 2 UMR INSERM 1246 – SPHERE, Nantes, France; 3 University Hospital Platform of Methodology and Biostatistics, Nantes, France

**Keywords:** Clinical trials, Patient-reported outcomes, Rasch measurement theory, Calibration

## Abstract

**Background:**

Meaningfully interpreting patient-reported outcomes (PRO) results from randomized clinical trials requires that the PRO scores obtained in the trial have the same meaning across patients and previous applications of the PRO instrument. Calibration of PRO instruments warrants this property. In the Rasch measurement theory (RMT) framework, calibration is performed by fixing the item parameter estimates when measuring the targeted concept for each individual of the trial. The item parameter estimates used for this purpose are typically obtained from a previous “calibration” study. But imposing this constraint on item parameters, instead of freely estimating them directly in the specific sample of the trial, may hamper the ability to detect a treatment effect. The objective of this simulation study was to explore the potential negative impact of calibration of PRO instruments that were developed using RMT on the comparison of results between treatment groups, using different analysis methods.

**Methods:**

PRO results were simulated following a polytomous Rasch model, for a calibration and a trial sample. Scenarios included varying sample sizes, with instrument of varying number of items and modalities, and varying item parameters distributions. Different treatment effect sizes and distributions of the two patient samples were also explored. Cross-sectional comparison of treatment groups was performed using different methods based on a random effect Rasch model. Calibrated and non-calibrated approaches were compared based on type-I error, power, bias, and variance of the estimates for the difference between groups.

**Results:**

There was no impact of the calibration approach on type-I error, power, bias, and dispersion of the estimates. Among other findings, mistargeting between the PRO instrument and patients from the trial sample (regarding the level of measured concept) resulted in a lower power and higher position bias than appropriate targeting.

**Conclusions:**

Calibration does not compromise the ability to accurately assess a treatment effect using a PRO instrument developed within the RMT paradigm in randomized clinical trials. Thus, given its essential role in producing interpretable results, calibration should always be performed when using a PRO instrument developed using RMT as an endpoint in a randomized clinical trial.

**Supplementary Information:**

The online version contains supplementary material available at 10.1186/s12874-022-01680-z.

## Background

Patient-Reported Outcomes (PRO) are defined as “any report of the status of a patient’s health condition that comes directly from the patient” [1]. PRO instruments are typically questionnaires for which the responses of patients to a set of items (questions) lead to the calculation of scores that are used to measure unobservable variables (also known as latent traits), such as pain, fatigue or anxiety. PRO scores are increasingly used as key endpoints to demonstrate the efficacy of new treatments in randomized clinical trials [2]..

PRO scores are produced based on a scoring algorithm, or scoring rules. The “scoring” typically has a range, and defines what is assumed to be a metric or unit (e.g., the European Organization for Research and Treatment of Cancer Quality of Life Questionnaire 30-item [EORTC QLQ-C30] Physical Functioning score ranges, after transformation, from 0 to 100, with increments of 6.67, based on the number of items and response categories [3]). In order to interpret these scores and a clinical trial result, it is necessary that scores have the same meaning between patients and samples, at the individual and at the group level [4–6]. For example, a score of “50” should express the same level of latent construct in all patients, for all applications. In metrology (i.e., the science of measurement), preserving of the same unit through different uses of an instrument is referred to as “traceability” [7]. Traceability is obtained through calibration of the instrument [7]. While calibration is primarily used for the measurement of physical quantities, it also plays an important role in other human sciences. For example, in education science, calibration ensures that scores from major educational tests, such as the Scholastic Aptitude Test (SAT), are calculated the same way and lead to comparable scores between each student [8]. In practice, calibration for PRO instruments can be based on the results of a reference application of the PRO instrument in a sample of reference, either from a dedicated calibration study or a psychometric “validation study” of the instrument.

The question of calibration of PRO instruments became more critical with the growing use of recent psychometric methods. PRO instruments used in clinical trials were initially developed in the classical test theory (CTT) paradigm [9], where the measurement result was obtained by a raw sum score. Raw scores do not need estimates from any specific sample to be calculated, so they are calibrated by construction. But, as this approach presents several theoretical limitations [10], alternative psychometric approaches (“modern psychometric methods”) are increasingly being preferred over CTT for the evaluation of PROs. Rasch Measurement Theory (RMT) is one such approach. Based on the Rasch model, it offers a different framework for calibration. The Rasch model separates the parameters of interest in the process of measurement of latent traits: item parameters (“difficulty” of the items, i.e., whether they discriminate more or less severe patients regarding their latent trait) and person parameters (measurements of the patient latent traits) [11–13]. This property ensures independence between the sample and the instrument (“specific objectivity”), and thus, allows proper calibration (i.e., estimation of item parameters that are independent from the samples on which they have been obtained).

Considering the RMT framework, calibration of PRO instruments first requires performing an RMT analysis on data from a “calibration” sample of patients. Obtained estimates of item parameters are then set to fixed in a formal RMT analysis of the clinical trial. Per its definition, this process allows making sure that obtained PRO scores are in the same unit, which is essential for their interpretation. Several PRO instruments developed in the RMT paradigm are used in clinical trials, with existing calibration solutions, such as the BREAST-Q [14] and the Rasch-built Overall Disability Scale (R-ODS) [15].

Calibration thus provide, per definition, some desirable properties for interpretability of the PRO results. But this major advantage could have a cost: it might in some cases negatively impact the detection of treatment effect by the clinical trial. In particular, if the sample size and heterogeneity of the calibration sample is not sufficient, with patients very different from those expected from the clinical trial regarding the concept of interest (e.g., more severe symptoms), some item parameters values to be used for calibration might be misspecified. In such cases, directly running the Rasch model on the trial sample (without a preliminary calibration step, i.e., non-calibration) could lead to more precise estimations of item parameters that are specifically targeted to the patients included. This in turn might lead to better conditions for evaluating treatment effect, despite putting the results at risk of being less interpretable. In a comparative, randomized trial, the impact of calibration might also differ depending on the method used for comparison of treatment groups. A possibility is to use a random effect Rasch model, directly including a covariate for group effect or first estimating the latent traits of the patients before performing a t-test, [16, 17] and the best approach still needs to be identified.

Previous simulation studies explored to some extent the impact of calibration on clinical trial results [18, 19]. However, calibration was not the main focus of these studies, and the impact of the characteristics of the calibration sample and its differences with the clinical trial sample were not evaluated. Also, these studies only explored the case where PRO instruments included only dichotomous items (with only two possible response options), which is not the most common structure for a PRO instrument in health studies.

The objective of this research was to further explore the potential negative impact of calibration on the statistical comparison of PRO measurements between treatment groups from a randomized clinical trial. Considering that calibration represent a benefit in itself for interpretability of the results, this research examined to which extent calibration can perform as good as non-calibration in the demonstration of treatment effect in randomized clinical trials. The research focused on calibration of PRO instruments that were developed and analysed in the RMT framework. For this purpose, we conducted a simulation study aiming to compare the use of calibrated and non-calibrated approaches on simulated polytomous PRO data from a randomized clinical trial, in the specific case of a cross-sectional endpoint. The impact of calibration was assessed for two different cross-sectional analysis methods and for different characteristics of the PRO instrument and of the samples of patients used in the calibration process.

## Methods

### The Rasch model

The Rasch model is a measurement, probabilistic model used to measure unobserved latent traits based on observed responses to items from a questionnaire (PRO instrument) [20]. The polytomous Rasch model (Partial Credit Model, PCM) is the generalization of the original Rasch model for ordered polytomous data (i.e. with more than 2, ordered, response options, of the Likert-scale type) [21]. Considering a PRO instrument including J items with the same number of response options M (modalities, coded from 0 to M-1) the model can be written as follows:

1 $$P\left({X}_{ij}=k|{\theta}_i,{\boldsymbol{\delta}}_{\boldsymbol{j}}\right)=\frac{\mathit{\exp}\left(k{\theta}_i-\sum_{l=1}^k\ {\delta}_{jl}\right)}{\sum_{r=0}^{M-1}\mathit{\exp}\left(r{\theta}_i-\sum_{l=1}^r\ {\delta}_{jl}\right)}$$

Where k is the response to patient *i* (i = 1, ..., N) to item *j* (j = 1, ..., J), realization of the random variable *X*_*ij*_ (*k* ∈ {0, …, *M* − 1}), *θ*_*i*_ the latent trait for patient *i*, and ***δ***_***j***_ the vector of dimension M-1 containing all category thresholds parameters *δ*_*jl*_ associated to categories *l* (*l* = 1, …, *M* − 1) of items *j*.

Considering the patient latent traits as realizations of a random variable assumed to be normally distributed results in a random effect PCM. Since the objective of a clinical trial is to compare treatments, a corresponding group covariate for treatment effect can be added to the model [16]. Denoting *γ* the parameter for the treatment effect (mean difference in latent trait between placebo and treated groups), patient latent traits are thus decomposed into a group effect (*μ*_0_ + *g*_*i*_*γ*) and an individual effect (${\theta}_{re{s}_i}$). The random effect PCM with treatment group effect can then be written as: 

2 $${\displaystyle \begin{array}{c}P\left({X}_{ij}=k|{\mu}_0,\gamma, {\theta}_{re{s}_i},{\boldsymbol{\delta}}_{\boldsymbol{j}}\right)=\frac{\mathit{\exp}\left(k\left({\mu}_0+{g}_i\gamma +{\theta}_{re{s}_i}\right)-{\delta}_{jl}\right)}{\sum_{r=0}^M\mathit{\exp}\left(r\left({\mu}_0+{g}_i\gamma +{\theta}_{re{s}_i}\right)-\sum_{l=1}^r{\delta}_{jl}\right)}\end{array}}$$

With *g*_*i*_ = 0 if patient i is in the placebo group, and *g*_*i*_ = 1 in the treated group, and thus *μ*_0_ corresponding to the mean of latent traits in the placebo group.

### Simulation of PRO data

Patient responses to multi-item PRO instruments with polytomous responses were generated using Monte Carlo simulations with a random effect PCM [21]. This assumes that the simulated PRO instrument was previously validated with RMT. For each iteration, we generated two samples:

- One for a calibration (or validation) study of the PRO instrument.
- One for a two parallel groups (treatment vs. placebo group) randomized clinical trial, at a post treatment occasion (cross-sectional data).

**Fig. 1:**
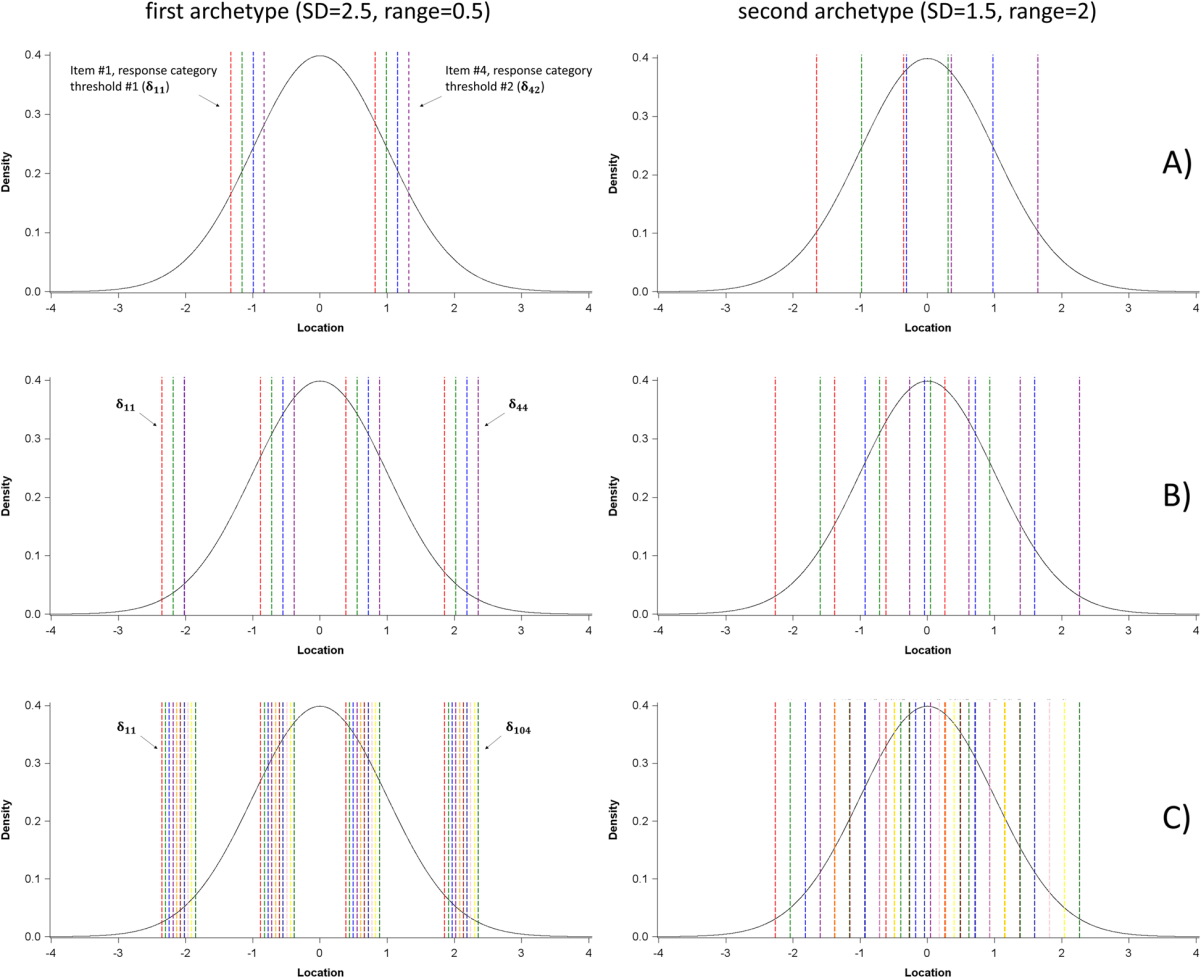
Illustration of the archetypes of items distribution, for different scenarios. Legend: Vertical dashed lines represent the item response category thresholds (δ_jl_, with each color corresponding to a different item) in different scenarios, and the probability density function curve represents the distribution of the latent trait in the calibration sample (case with a variance = 1). The left part of the figure includes cases where the item locations δ_j_ have a low dispersion (range = 0.5) and the δ_jl_ have a high dispersion (SD = 2.5). The right part of the figure includes cases where the item locations δ_j_ have a high dispersion (range = 2) and the δ_jl_ have a low dispersion (SD = 1.5). Each line corresponds to different scenarios regarding the number of item and modalities: A) J = 4 items, M = 3 modalities. B) J = 4 items, M = 5 modalities. C) J = 10 items, M = 5 modalities. Full values for the response category thresholds δ_jl_ are provided in supplementary materials (Additional file 1)

Calibration and trial samples shared the same PRO instrument characteristics, which varied based on several parameters between different scenarios:

- The number of items J from the PRO instrument varied between 4 and 10, in accordance with the size of the subscales of PRO instruments that are commonly used in clinical research.
- The number of response categories M was of 3 or 5, in accordance with commonly encountered number of possible response options in PRO instruments with items of the Likert-scale type (ordered response options). Response categories were coded from 0 to M-1.
- Distribution of the thresholds, *δ*_*jl*_ (which corresponds to the level of latent trait for which an patient has the same probability to endorse one or the other of two subsequent ordered response categories, with *l* the response option, from 1 to *M* − 1, of the item *j*) and associated item locations *δ*_*j*_ (which corresponds to the mean of the category thresholds for each given item) was designed to reflect two typical archetypes of PRO instruments encountered in practice (see Fig. 1 for an illustration of the two cases):
  - A first archetype where the item locations *δ*_*j*_ had a low dispersion on the continuum measured by the instrument (*δ*_*j*_ regularly spaced from − 0.25 to 0.25), with highly dispersed category thresholds *δ*_*jl*_ regularly spaced for one given item, based on the percentiles of a normal distribution (if the items have 3 response categories, thresholds were set to the 33rd and 66th percentiles of the distribution; If the items have 5 response categories, thresholds were set to the 20th, 40th, 60th and 80th percentiles) centered on *δ*_*j*_ with a standard deviation (SD) of 2.5. This is typically observed with instruments in which the variability over the latent trait is supposed to be captured by varying levels of the response scale. Such item distributions can be observed with instruments developed using CTT methods, as “redundancy” of the items on the continuum (items with very close category thresholds *δ*_*jl*_) is not identified as problematic using CTT methods [22] (in fact this pattern reflects the theoretical notion of “parallel items sets” of the CTT paradigm [23]).
  - A second archetype where the item locations *δ*_*j*_ were highly dispersed on the continuum measured by the instrument (*δ*_*j*_ regularly spaced from − 1 to 1), with response category thresholds *δ*_*jl*_ with low dispersion, regularly spaced for one given item, based on the percentiles of a normal distribution centered on *δ*_*j*_ with a SD of 1.5. This corresponds to PRO instruments in which the variability over the latent trait is supposed to be captured with items representing different levels on the continuum (“item hierarchy”). It is commonly observed with instrument developed using RMT [14, 15].
- The mean of item parameters was set to 0 (following the specified distribution for item parameters).

Calibration samples varied between scenarios based on several parameters:

- The full sample size of the calibration sample *N*_*calibration*_ varied between 100 and 500. Values were selected to reflect the range of sample sizes that can be encountered in clinical research studies for validation of PRO instruments [14, 15, 24].
- The latent trait distribution was defined as normal, in line with the hypothesis underlying the use of a random effect PCM model.
- The mean of the latent trait distribution was set to 0 in the calibration sample, to reflect a perfect targeting between the sample and the PRO instrument.
- Variance of the latent trait distribution was set to 1 or 2, to explore different cases of heterogeneity of the calibration population.

Trial samples varied between scenarios based on several parameters:

- The sample size within each treatment group, *N*_*trial*_, varied between 50 and 500 (equal size between the two groups). Values were selected to reflect the range of sample sizes that can be encountered in clinical trials.
- The effect size of the treatment (standardized mean difference of patients’ latent traits between treatment groups), γ, varied between 0 and 0.8 to explore various scenarios from no to large difference between treatment groups.
- The mean μ of the latent traits varied from 0 to 2.5 to explore cases where the trial sample and the PRO instrument showed perfect targeting to high mistargeting. Mistargeting may typically occur in practice when the trial population differs from the population of the validation study of the PRO instrument used for calibration (e.g., more or less severe sample with regards to the disease). Within treatment groups, the mean of latent traits was thus respectively μ_0_ for placebo and μ_0_ + γ for treatment group.
- Variance within each treatment group was set to 1.

Each simulation scenario resulted in a set of PRO responses for a calibration sample and a trial sample and was replicated 500 times. Details of all simulation parameters with their possible values are described in Table 1. Data were simulated using the -simirt- module from STATA software [25].

**Table 1:** Values of simulation parameters

Characteristics	Parameter	Values
PRO instrument	Number of items J	4, 7, 10
	Number of response categories M (response options from 0 to M-1)	3, 5
	Item locations (*δ*_*j*_) and category thresholds (*δ*_*jl*_) distribution	First archetype: ${\delta}_j=-0.25+\frac{0.5}{J-1}\left(j-1\right),\kern0.5em j=1\dots J$ (Regularly spaced between − 0.25 + 0.25) *δ*_*jl*_ defined as the percentiles from a normal distribution with SD of 2.5 Second archetype: ${\delta}_j=-1+\frac{2}{J-1}\left(j-1\right),\kern0.5em j=1\dots J$ (Regularly spaced between −1 + 1), *δ*_*jl*_ defined as the percentiles from a normal distribution with SD of 1.5
Calibration sample	Sample size *N*_*validation*_	100, 250, 500
	Variance	1, 2
	Mean of latent trait	0
Trial sample	Sample size *N*_*trial*_	50, 100, 200, 500 (per group)
	Effect size (Standardized mean difference between groups) γ	0, 0.2, 0.5
	Mean of latent trait *μ*	0, 0.5, 2
	Variance within each group	1

### Estimation

Simulated PRO data from each sample (calibration and trial) and within each scenario were analysed using a random effect PCM. A treatment group covariate (fixed effect) was also included in the model for the analysis of the trial samples (Eq. 2). Treatment effect parameter (γ) and difficulties associated to category thresholds of each item (*δ*_*jl*_) were estimated by maximizing the marginal likelihood (MML) [26]. In the trial samples, the estimators of each patient latent trait were also obtained using expected a posteriori Bayesian estimates [17].

### Calibration

The calibrated and non-calibrated approaches were used, for each scenario. In the calibrated approach, item parameters were estimated based on the calibration sample. The obtained values for $\hat{\delta_{jl}}$ were then assumed to be known without error and considered as fixed for the analysis of the trial sample.

In the non-calibrated approach, the calibration sample was not considered, and the estimation of item parameters was directly conducted on the trial sample.

### Comparison of treatment groups

Two methods were used to compare treatment groups in the trial sample, for each simulated scenario, and for calibrated and non-calibrated approaches:

- Direct estimation of treatment group effect $\hat{\gamma}$, and testing of the nullity of the parameter using a Wald test.
- Comparison of expected a posteriori Bayesian patient latent trait parameter $\hat{\theta_i}$ between the treatment groups using a t-test.

### Criteria for comparison of approaches

The calibrated and non-calibrated approaches were compared along with the method used for comparing treatment groups based on the following criteria:

- Type-I error (α risk), which was obtained by computing the proportion of rejection of the null hypothesis among the 500 replications of each scenario with no simulated a priori difference between treatment groups (γ = 0).
- Power (1-β), which was obtained by computing the proportion of rejection of the null hypothesis among the 500 replications of each scenario with simulated a priori difference between treatment groups (γ ≠ 0).
- Position bias on the estimation of the treatment effect, which was obtained by computing the mean of the observed differences between $\hat{\gamma}$ and *γ* based on the 500 replications of each scenario.
- Standard deviation of the estimate of treatment effect, which was obtained by computing the standard deviation of the obtained $\hat{\gamma}$ from the 500 replications of each scenario.

Analyses were performed using STATA software, version 14.

## Results

Table 2 displays, for selected scenarios of interest, the results of the simulation study: type-I error, power, position bias and SD of the estimates for the difference between treatment groups. The scenarios were selected to focus on the parameters that showed an impact on any of these criteria, and to retain medium values for power, for a better interpretability of the results (to avoid a ceiling effect, i.e., a power of 100%). The following 36 scenarios are presented: J of 4, 7 or 10, M of 3 or 5, distribution of item parameters corresponding to the second archetype (SD = 1.5, range = 2), N_calibration_ of 250, variance of 1, N_trial_ of 200 or 500, μ of 0, 0.5 or 2, γ of 0.2 (0 for the calculation of type-I error). Comprehensive results for other scenarios can be found in supplementary materials (Additional file 2). Overall, the type-I error was well controlled and remained unchanged for all explored scenarios, i.e. calibration approaches and comparison of groups methods.

**Table 2:** Type-I error, power, position bias and SD of the treatment effect estimates

J	M	N_trial_	μ	γ = 0				γ = 0.2											
				Type-I error				Power				Position bias				SD of the estimates			
				Non calibrated		Calibrated		Non calibrated		Calibrated		Non calibrated		Calibrated		Non calibrated		Calibrated	
				$\hat{\gamma}$	t test on $\hat{\theta_i}$	$\hat{\gamma}$	t test on $\hat{\theta_i}$	$\hat{\gamma}$	t test on $\hat{\theta_i}$	$\hat{\gamma}$	t test on $\hat{\theta_i}$	$\hat{\gamma}$	t test on $\hat{\theta_i}$	$\hat{\gamma}$	t test on $\hat{\theta_i}$	$\hat{\gamma}$	t test on $\hat{\theta_i}$	$\hat{\gamma}$	t test on $\hat{\theta_i}$
4	3	200	0	5.6	5.6	5.6	5.6	32.8	32.8	32.8	32.6	0.01	0.08	0.01	0.08	0.13	0.08	0.13	0.08
			0.5	4.4	4.4	4.6	4.4	32.8	32.8	33.2	33.0	0.00	0.08	0.00	0.08	0.13	0.08	0.13	0.08
			2	5.8	5.6	5.6	5.4	29.6	29.6	29.8	29.6	0.00	0.10	0.01	0.10	0.15	0.07	0.15	0.07
		500	0	6.8	6.6	6.8	6.8	68.0	68.0	68.0	68.0	0.00	0.08	0.00	0.08	0.08	0.05	0.08	0.05
			0.5	2.6	2.6	2.6	2.6	68.4	68.4	68.6	68.4	0.00	0.08	0.00	0.08	0.08	0.05	0.08	0.05
			2	4.6	4.6	4.6	4.6	58.6	58.6	58.4	58.4	0.00	0.11	0.00	0.10	0.09	0.04	0.09	0.05
	5	200	0	4.6	4.6	4.8	4.6	41.2	41.2	41.2	40.6	0.00	0.05	0.00	0.05	0.12	0.09	0.12	0.09
			0.5	4.6	4.6	4.6	4.4	40.8	40.8	40.6	40.4	0.00	0.05	0.00	0.05	0.12	0.09	0.12	0.09
			2	5.4	5.4	5.6	5.4	32.2	32.4	32.6	32.4	0.00	0.08	0.00	0.07	0.13	0.08	0.13	0.08
		500	0	5.2	5.2	5.2	5.2	76.6	76.6	76.8	76.6	0.00	0.05	0.00	0.05	0.07	0.06	0.07	0.06
			0.5	4.6	4.6	4.6	4.6	75.6	75.6	75.8	75.8	0.00	0.05	0.00	0.05	0.08	0.06	0.08	0.06
			2	4.2	4.2	4.2	4.2	69.8	69.8	69.4	69.4	0.00	0.08	0.00	0.07	0.08	0.05	0.08	0.05
7	3	200	0	7.6	7.2	7.4	7.2	40.8	40.8	40.8	40.8	0.00	0.05	0.00	0.05	0.12	0.09	0.12	0.09
			0.5	5.4	5.2	5.6	5.2	40.0	40.0	40.2	40.0	0.00	0.06	0.00	0.06	0.12	0.08	0.12	0.08
			2	5.2	5.2	5.2	5.2	36.6	36.6	36.6	36.4	0.01	0.08	0.01	0.08	0.13	0.08	0.13	0.08
		500	0	5.2	5.2	5.2	5.2	76.8	76.6	77.0	76.8	0.00	0.05	0.00	0.05	0.07	0.05	0.07	0.05
			0.5	5.2	5.2	5.2	5.2	75.0	75.0	75.0	75.0	0.00	0.05	0.00	0.05	0.08	0.06	0.08	0.06
			2	3.6	3.6	3.6	3.6	67.8	67.8	67.8	67.8	0.00	0.08	0.00	0.08	0.08	0.05	0.09	0.05
	5	200	0	5.2	5.2	5.2	5.2	42.6	42.6	42.8	42.6	0.00	0.03	0.00	0.03	0.11	0.09	0.11	0.09
			0.5	5.2	5.0	5.2	5.0	46.0	45.6	46.0	46.0	0.00	0.03	0.00	0.03	0.11	0.10	0.11	0.10
			2	6.4	6.4	6.4	6.4	41.2	40.6	41.2	40.6	0.00	0.05	0.01	0.05	0.12	0.09	0.12	0.09
		500	0	6.8	6.8	6.8	6.8	80.8	80.8	80.8	80.6	0.00	0.03	0.00	0.03	0.07	0.06	0.07	0.06
			0.5	3.2	3.0	3.2	3.2	80.8	80.8	80.6	80.6	0.00	0.03	0.00	0.03	0.07	0.06	0.07	0.06
			2	5.2	5.2	5.4	5.2	73.6	73.6	73.4	73.2	0.01	0.06	0.01	0.06	0.07	0.05	0.08	0.06
10	3	200	0	4.2	4.2	4.2	4.2	42.6	42.4	42.6	42.2	0.00	0.04	0.00	0.04	0.11	0.09	0.12	0.09
			0.5	5.2	5.2	5.2	5.2	42.0	41.6	42.2	41.6	0.00	0.04	0.00	0.04	0.11	0.09	0.11	0.09
			2	4.0	3.6	4.0	3.6	39.2	39.0	39.2	39.0	0.00	0.07	0.00	0.07	0.13	0.08	0.13	0.09
		500	0	4.8	4.4	4.6	4.4	79.4	79.2	79.4	79.2	0.00	0.04	0.00	0.04	0.07	0.06	0.07	0.06
			0.5	6.2	6.2	6.2	6.2	78.8	78.8	78.8	78.8	0.00	0.04	0.00	0.04	0.07	0.06	0.07	0.06
			2	8.2	8.2	8.2	8.2	76.0	75.8	75.8	75.6	0.00	0.07	0.00	0.06	0.08	0.05	0.08	0.05
	5	200	0	5.0	5.0	5.2	5.0	46.0	45.8	45.4	45.4	0.00	0.02	0.00	0.02	0.10	0.09	0.10	0.09
			0.5	5.6	5.6	6.0	5.6	47.2	46.8	47.2	46.4	0.00	0.02	0.00	0.02	0.11	0.09	0.11	0.10
			2	4.4	4.2	4.4	4.0	42.0	41.6	42.0	41.6	0.00	0.04	0.00	0.04	0.11	0.09	0.11	0.09
		500	0	5.6	5.4	5.6	5.6	84.4	84.2	84.4	84.2	0.00	0.02	0.00	0.02	0.07	0.06	0.07	0.06
			0.5	6.2	6.2	6.2	6.2	83.8	83.8	84.2	83.8	0.00	0.02	0.00	0.02	0.07	0.06	0.07	0.06
			2	6.6	6.4	6.6	6.4	81.8	81.6	81.8	81.6	0.00	0.04	0.00	0.04	0.07	0.06	0.07	0.06

Legend: results are presented for selected scenarios, with *N*_*calibration*_ = 250, distribution of the item parameters = second archetype with SD of 1.5 and range of 2, and variance of the calibration sample = 1

### Impact of calibration

**Fig. 2:**
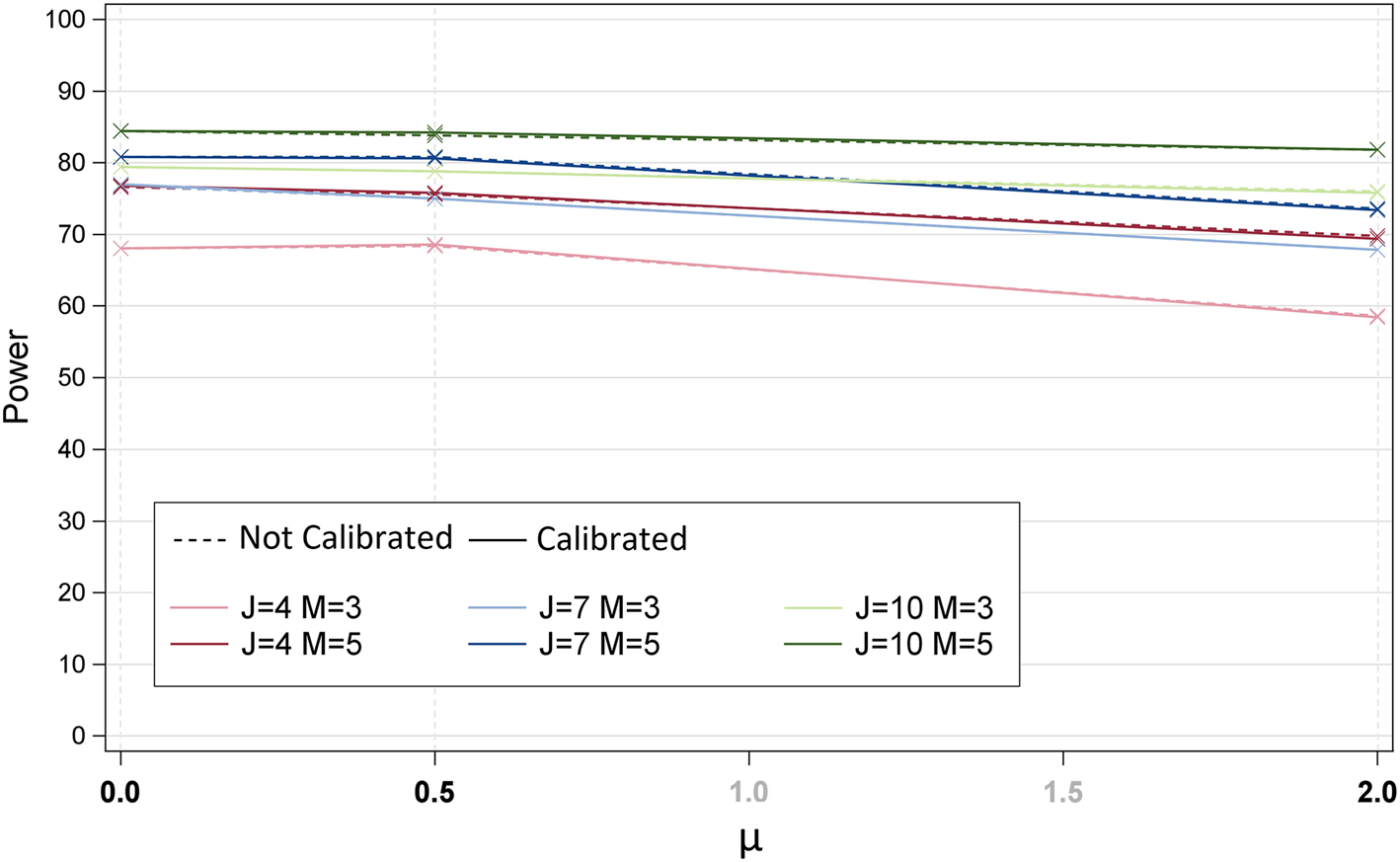
Power using calibrated and non-calibrated approaches, depending on mistargeting of the trial sample μ. Legend: power is presented for instruments with varying number of items J and modalities M. Presented results are for comparison of treatment groups based on $\hat{\gamma}$, for scenarios with the distribution of the item parameters = second archetype with SD of 1.5 and range of 2, γ = 0.2, *N*_*trial*_ = 500, *N*_*calibration*_ = 250, variance of the calibration sample = 1

The simulations did not show any impact of the use of the calibration approach on the type-I error, power, position bias and SD of the estimates (Table 2). In particular, there was no impact even in the most disadvantageous cases for the calibration approach as compared to non-calibration (cases where the item parameters estimated from the calibration sample are expected to be less precise than the ones estimated from the trial): high mistargeting μ, small *N*_*calibration*_ and large *N*_*trial*_, small variance of the calibration sample. There was thus no impact of the calibration sample parameters (*N*_*calibration*_ and variance of the sample) on any criteria. The absence of impact of the calibration approach is visible in the example scenario presented in Fig. 2, as the power was similar for the calibrated and non-calibrated approaches (the curves overlap), for all levels of mistargeting.

### Impact of comparison of treatment groups method

The simulations did not show any impact of the comparison of treatment groups method on the power and type-I error (Table 2). There was no position bias when estimating the treatment effect using a group covariate. However, a position bias was found when using patient latent trait estimates, the difference between groups being all the more underestimated as the number of items J was small and mistargeting was large. Of note, the SD of the estimates was higher when using a direct estimation of treatment group effect as compared to using a posteriori Bayesian patient latent trait estimates.

### Impact of trial sample characteristics

As expected, an increase of the power with the sample size of the trial (*N*_*trial*_, see Table 2) and the effect size (γ, data not shown) was observed. Results with a small mistargeting (μ = 0.5) or optimal targeting (μ = 0) resulted in comparable power. A large mistargeting of the sample (μ = 2) resulted in a lower power (Fig. 2 and Table 2).

### Impact of PRO instrument characteristics

Increased number of items and response categories resulted in increased power (Fig. 2 and Table 2). Also, the position bias observed using a posteriori Bayesian patient latent trait estimates was reduced when the number of items and response categories increased (Table 2). The number of item and response categories did not show any impact on the type-I error and the SD of the estimate of treatment effect (Table 2). The distribution of the item and response categories did not show any clear impact on any criteria.

## Discussion

This simulation study explored the impact of calibration of polytomous PRO instruments on the comparison of treatment groups in a randomized clinical trial. This impact was evaluated within the RMT framework, considering different methods for comparison of treatment groups, and various settings (characteristics of the PRO instrument, calibration and trial samples). The lack of impact of calibration observed in the study showed that the benefit in terms of interpretability, brought by the traceability property warranted by calibration, is not obtained at the expense of the ability to show a true difference between treatment groups or in terms of proper control of the type-I error. Given its important added value in terms of interpretability of the results, calibration should thus always be performed when a PRO instrument developed and analysed in the RMT framework is used as an endpoint in a randomized clinical trial.

The simulations consistently showed that the type-I error, power of test for the comparison of the two groups, bias, and dispersion of the estimated difference between treatment groups were similar for calibrated and non-calibrated approaches. Calibration did not have any impact even in the most favorable cases for the use of non-calibrated estimates, i.e., when the calibration sample size was small with low variance and when the trial sample size was large with high mistargeting. The present results also confirmed previous simulation studies. Blanchin et al. explored the impact of misspecification of dichotomous item parameters at the design stage, while attempting to estimate the power of a clinical trial [19]. They showed that such misspecification had no impact on power, which indirectly support calibration: errors in the item parameters used for calibration would not likely impact power [19]. Findings were also consistent with simulation studies from Sébille et al. and Hamel et al., which included comparison of cases where the dichotomous item parameters were considered as known (i.e., use of calibration) or unknown and estimated from the trial data (non-calibration) [18, 27].

Regardless of the calibration situation, mistargeting of the PRO instrument to the clinical trial sample impacted the ability to detect a treatment effect in the clinical trial. Indeed, a large mistargeting of the sample resulted in lower power and higher dispersion of the estimates of the treatment effect. This is consistent with the findings from a previous simulation study, where mistargeting between the PRO instrument (with dichotomous items) and the sample was associated to lower power [28]. This confirms that PRO instruments should be properly targeted to the level of severity of the patient population included in the trial, to be able to effectively detect treatment effect. This is especially true when the mistargeting results in floor or ceiling effect (i.e., when no items are included to capture low or high level of the measured concept), as it was the case in this work for the scenarios with large mistargeting. A small mistargeting did not seem to impact the results, but this should be interpreted cautiously, as it may be affected by the exact distribution of the items and patients over the continuum: lower levels of mistargeting might still show an impact when item distribution is very uneven or associated to less homogenous or non-normally distributed patient samples.

Additionally, and as already flagged by multiple studies, higher number of items and response categories resulted in higher power [18, 27]. This impact on power can be compared to the one, well known, of the number of patients included in the trial. Considering the case of a trial with 200 patients with an effect size of 0.2, our simulations showed that shifting from 4 items with 3 response categories to 10 items with 5 response categories represented an increase of power from 30 to 45%. Considering the same example case and the simulation results, this is approximately similar to the impact on power that would be observed from adding 100 patients to the trial. This confirms the importance of using PRO instruments that include enough items in small sample studies. This aspect should be carefully considered when shorter instruments are recommended, typically to “minimize patients’ response burden” [29]. Also, interestingly, the decrease of power due to a high mistargeting was lower when the PRO instruments included a large number of items and response categories (note that, as noted above, this finding may be somewhat dependent on the specific distribution of items and patients used in our simulations).

This study came with several limitations and further necessary developments can be underlined. First, the calibration process only investigated the case where patients only differed based on their level of latent trait between the calibration and the trial sample. But in real-life studies, patients can differ on other characteristics, such as their demographics, etc. In some cases, these characteristics impact the way patients respond to the items, despite having the same level of latent trait: item parameter values may differ depending on these characteristics, which is known as differential item functioning (DIF) [30]. If patients from the calibration and the trial sample differ based on a characteristic that creates DIF (e.g., they have different disease subtypes, or different countries imply cultural differences despite having the same language, etc.), the item parameter values used from the calibration sample will not be fully adequate for the clinical trial. A solution may be to obtain different sets of item parameters values from different calibration samples, to be used alternatively to calibrate the measure depending on the population of the trial. For example, different sets of calibration are proposed to calculate PROMIS scores [6]. But in many cases when conducting a clinical trial, there is no available calibration set perfectly suited to the population of interest. In this situation, it is possible that calibration with wrongly specified parameters would hinder the ability of the trial to accurately assess an effect of the treatment. A previous simulation study showed that DIF, if ignored in the analysis, could result in biased estimates of the difference between groups [31, 32]. The impact of calibration in the presence of DIF should thus be further explored. Another limitation stands in the approaches for comparison of treatment groups that were explored in this study. Our simulations only considered results from a random effect PCM. Considering statistical methods that compare individual PRO measures in a different estimation context would be informative. Typically, investigating the implication of using statistical methods that compare PRO estimates from a fixed effect PCM with pairwise conditional maximum likelihood (as performed in RUMM, one of the currently most commonly used software for RMT analysis [33]) would allow gaining a better understanding of the various options for the analysis of PRO measures resulting from a RMT paradigm in a clinical trial, and the relative impact of calibration in these various cases. Of note, our between-group comparisons were based on cross-sectional comparison of treatment group at a given timepoint. It may not be the method of choice in other longitudinal designs (especially in non-randomized clinical trials). Other methods such as comparison of patient trajectories over time using a repeated measurement model may also be encountered. We do not know whether the use of these other methods would result in different conclusions regarding the impact of calibration, in particular as randomization allows for controlling differences of baseline levels between treatment groups. Another limitation stands in the distributions that were used within this simulation study, which were normal and with the patient data showing an optimal fit to the model. While this represents a theoretically ideal case, this might have resulted in an overestimated performance of a random effect PCM as compared to analysis on real observed data. Based on simulations, further studies using non-normal PRO data, or with a non-perfect (yet, “good enough”) fit to the Rasch model would also be of interest. An illustrative example of the use of calibration and non-calibration approaches on real clinical trial data might also be of interest for future research. Finally, we deliberately restricted the scope of these analyses to the RMT framework and did not address the question in the context of the competing modern psychometric paradigm, Item-Response Theory (IRT). While a similar process as the one used here for RMT can be used for calibration based on IRT models, different findings and recommendations may be obtained. Previous research has already suggested that larger calibration sample is needed in order to obtain reliable estimates of individual patient latent traits for IRT models [34]. Additionally, the “specific objectivity” property of the Rasch model, resulting from the separation of the item and person parameters in the model, is central in the calibration process. Further research should be conducted to explore whether our conclusions are confirmed using data generated and analysed with an IRT model.

This work showed that calibration was always an appropriate option when analysing PRO endpoints from a randomized clinical trial, in PRO instruments developed in an RMT framework. For calibration to be possible, the PRO instrument must have previously undergone RMT analysis, with a set of item parameters available in the literature (set of values to be re-used in different trials). Some instruments developed in the RMT paradigm provide the possibility to calibrate the estimates in other studies, such as the BREAST-Q [14], FACE-Q [35] and other instruments from the “Q-portfolio”, the R-ODS [15], the StomaQoL [36] or the 88-item Multiple Sclerosis Spasticity Scale (MSSS-88) [37]. The benefit of new treatments in terms of PROs using these RMT-calibrated instruments has been investigated in randomized clinical trials [38–41] and non-comparative trials [42, 43]. Similarly, the PROMIS or the EORTC QLQ-C30 computerized adaptative testing (CAT) also use calibration, but in an IRT paradigm [44, 45]. However, it does not seem to be systematically the case [46, 47]. Based on the findings of our simulations, we would recommend that calibration is consistently considered by developers of new PRO instruments using the RMT framework, and by clinical trial statisticians who are analysing data from these instruments. Using a formal RMT analysis, the treatment groups can be compared by including a covariate in a random effect Rasch model. Patient latent traits can also be estimated, based on a random or fixed effect Rasch model, before comparing the groups (e.g., using a t-test). Another, simpler option to obtain calibrated measures of patient latent traits is to use conversion tables that allows transforming raw scores to approximated measurements from the Rasch model. Shortcomings of this approach include that patient measurements cannot be assessed in the presence of missing item. While these different methods allowing for calibration seem to perform differently (e.g., performing a t-test on patient estimated latent traits from a random effect Rasch model showed to be biased, as observed in the current study [18]), there is no definitive consensus on the method to be preferred. The methods used will also have to be carefully considered to appropriately take benefit of the metrological advantages of the PRO measures underpinned by the Rasch model (i.e., possibility of having interval-level scales and measurement uncertainty at the individual level).

## Conclusions

The RMT framework allows for proper calibration of PRO instruments in randomized clinical trials. In such context, our simulation study showed that calibration of the PRO instruments resulted in similar ability of the trial to demonstrate treatment effect as compared to non-calibration. As a consequence, calibration should be consistently performed since it guarantees per definition expressing PRO results in the same unit (traceability), which is an important added value for interpretability. For calibration to be possible, proper sets of item parameters values or conversion tables obtained from calibration samples should be provided for the PRO instruments developed with RMT.

## Supplementary Information

**Additional file 1.** Values of the response category thresholds for the scenarios presented in Fig. 1.

**Additional file 2.** Type-I error, power, position bias and SD of the estimate of the difference between treatment groups (full results of the simulations).

## Data Availability

The datasets generated and analysed during the current study are available in the OSF repository, https://osf.io/prbaj/?view_only=9c7493c7ba6548bf9819e4d730077b3e. The Stata^©^ programs used to analyse the datasets are available from the corresponding author.

## Abbreviations

CAT: Computerized adaptative testing
CTT: Classical test theory
DIF: Differential item functioning
EORTC QLQ-C30: European Organization for Research and Treatment of Cancer Quality of Life Questionnaire
IRT: Item response theory
MML: Marginal maximum likelihood
MSSS-88: 88-item Multiple Sclerosis Spasticity Scale
PCM: Partial Credit Model
PRO: Patient-reported outcome
PROMIS: PRO Measurement Information System
RMT: Rasch measurement theory
R-ODS: Rasch-built overall disability scale
SAT: Scholastic aptitude test
SD: Standard deviation
