# Supplementary material for: Evaluating the impact of calibration of patient-reported outcomes measures on results from randomized clinical trials: a simulation study based on Rasch measurement theory
Source: BMC Med Res Methodol. 2022 Aug 12;22:224. doi: 10.1186/s12874-022-01680-z (PMC9375403; doi:10.1186/s12874-022-01680-z)
Supplement: Supplementary file 1 — Additional file 1. Values of the response category thresholds for the scenarios presented in Fig. 1. [file 12874_2022_1680_MOESM1_ESM.docx]

Table 1. Values of the response category thresholds for the scenarios presented in Figure 1

$\delta_{\mathrm{jl}}$=response category threshold for item j (from 1 to J) and response option l (from 1 to M-1)

| First archetype (SD=2.5, range=0.5), J=4 items, M=3 modalities | Second archetype (SD=1.5, range=2), J=4 items, M=3 modalities |
| --- | --- |
| $\delta_{11}$=-1.3291 $\delta_{12}$=0.8222  $\delta_{21}$=-1.1624 $\delta_{22}$=0.9889  $\delta_{31}$=-0.9958 $\delta_{32}$=1.1556  $\delta_{41}$=-0.8291 $\delta_{42}$=1.3222 | $\delta_{11}$=-1.6475 $\delta_{12}$=-0.3567    $\delta_{21}$=-0.9808 $\delta_{22}$=0.3100  $\delta_{31}$=-0.3142 $\delta_{32}$=0.9767  $\delta_{33}$=0.3525 $\delta_{34}$=1.6433 |
| First archetype (SD=2.5, range=0.5), 4 items, M=5 modalities | Second archetype (SD=1.5, range=2), 4 items, M=5 modalities |
| $\delta_{11}$=-2.3541 $\delta_{12}$=-0.8834 $\delta_{13}$=0.3834 $\delta_{14}$=1.8540    $\delta_{21}$=-2.1874 $\delta_{22}$=-0.7167 $\delta_{23}$=0.5500 $\delta_{24}$=2.0208  $\delta_{31}$=-2.0207 $\delta_{32}$=-0.5500 $\delta_{33}$=0.7167 $\delta_{34}$=2.1874    $\delta_{41}$=-2.0207 $\delta_{42}$=-0.3834 $\delta_{43}$=0.8834 $\delta_{44}$=2.3540 | $\delta_{11}$=-2.2624 $\delta_{12}$=-1.3800 $\delta_{13}$=-0.6200 $\delta_{14}$=0.2624    $\delta_{21}$=-1.5954 $\delta_{22}$=-0.7130 $\delta_{23}$=0.04702 $\delta_{24}$=0.9294  $\delta_{31}$=-0.9294 $\delta_{32}$=-0.0470 $\delta_{33}$=0.7130 $\delta_{34}$=1.5954    $\delta_{41}$=-0.2624 $\delta_{42}$=0.6200 $\delta_{43}$=1.3800 $\delta_{44}$=2.2624 |
| First archetype (SD=2.5, range=0.5), J=10 items, M=5 modalities | Second archetype (SD=1.5, range=2), J=10 items, M=5 modalities |
| $\delta_{11}$=-2.3540 $\delta_{12}$=-0.8834 $\delta_{13}$=0.3834 $\delta_{14}$=1.8541    $\delta_{21}$=-2.2985 $\delta_{22}$=-0.8278 $\delta_{23}$=0.4389 $\delta_{24}$=1.9096    $\delta_{31}$=-2.2429 $\delta_{32}$=-0.7723 $\delta_{33}$=0.4945 $\delta_{34}$=1.9652    $\delta_{41}$=-2.1874 $\delta_{42}$=-0.7167 $\delta_{43}$=0.5500 $\delta_{44}$=2.0207  $\delta_{51}$=-2.1318 $\delta_{52}$=-0.6611 $\delta_{53}$=0.6056 $\delta_{54}$=2.0763    $\delta_{61}$=-2.0763 $\delta_{62}$=-0.6056 $\delta_{63}$=0.6611 $\delta_{64}$=2.1318    $\delta_{71}$=-2.0207 $\delta_{72}$=-0.5500 $\delta_{73}$=0.7167 $\delta_{74}$=2.1874    $\delta_{81}$=-1.9652 $\delta_{82}$=-0.4945 $\delta_{83}$=0.7723 $\delta_{84}$=2.2429  $\delta_{91}$=-1.9096 $\delta_{91}$=-0.4389 $\delta_{93}$=0.8278 $\delta_{94}$=2.2985    $\delta_{101}$=-1.8541 $\delta_{102}$=-0.3834 $\delta_{103}$=0.8834 $\delta_{104}$=2.3541 | $\delta_{11}$=-2.2624 $\delta_{12}$=-1.3800 $\delta_{13}$=-0.6200 $\delta_{14}$=0.2624  $\delta_{21}$=-2.0402 $\delta_{22}$=-1.1578 $\delta_{23}$=-0.3978 $\delta_{24}$=0.4846    $\delta_{31}$=-1.8179 $\delta_{32}$=-0.9355 $\delta_{33}$=-0.1755 $\delta_{34}$=0.7069    $\delta_{41}$=-1.5957 $\delta_{42}$=-0.7133 $\delta_{43}$=0.0467 $\delta_{44}$=0.9291    $\delta_{51}$=-1.3735 $\delta_{52}$=-0.4911 $\delta_{53}$=0.2689 $\delta_{54}$=1.1513    $\delta_{61}$=-1.1513 $\delta_{62}$=-0.2689 $\delta_{63}$=0.4911 $\delta_{64}$=1.3735    $\delta_{71}$=-0.9291 $\delta_{72}$=-0.0467 $\delta_{73}$=0.7133 $\delta_{74}$=1.5957  $\delta_{81}$=-0.7069 $\delta_{82}$=0.1755 $\delta_{83}$=0.9355 $\delta_{84}$=1.8179    $\delta_{91}$=-0.4846 $\delta_{92}$=0.3978 $\delta_{93}$=1.1578 $\delta_{94}$=2.0402  $\delta_{101}$=-0.2624 $\delta_{102}$=0.6200 $\delta_{103}$=1.3800 $\delta_{104}$=2.2624 |
