# Supplementary material for: Evaluating the impact of calibration of patient-reported outcomes measures on results from randomized clinical trials: a simulation study based on Rasch measurement theory
Source: BMC Med Res Methodol. 2022 Aug 12;22:224. doi: 10.1186/s12874-022-01680-z (PMC9375403; doi:10.1186/s12874-022-01680-z)
Supplement: Supplementary file 2 — Additional file 2. Type-I error, power, position bias and SD of the estimate of the difference between treatment groups (full results of the simulations). [file 12874_2022_1680_MOESM2_ESM.docx]

Table 1. Type-I error, power, position bias and SD of the estimate of the difference between treatment groups (full results of the simulations)

Legend: J=number of items, M=number of modalities, $N_{trial}$=sample size in the trial sample (by group), $N_{\mathrm{cal}}$=sample size in the calibration sample, μ=mean of the latent trait in the trial sample (mistargeting), γ=treatment effect size, SD=standard deviation of the distribution of the item response category thresholds, r=range of the item locations, V=variance of latent traits in the calibration sample. “Num. Rep” corresponds to the number of effective replications: if it was not possible to calculate all item parameters from the calibration sample, the replication was discarded. Of note, power corresponds to the type I error in scenarios where γ=0.

| J | M | $N_{\mathrm{trial}}$ | $N_{\mathrm{cal}}$ | μ | γ | SD | r | V | Power (or type-I error) | | | | Position bias | | | | SD of the estimates | | | | Num. Rep. |
| --- | --- | --- | --- | --- | --- | --- | --- | --- | --- | --- | --- | --- | --- | --- | --- | --- | --- | --- | --- | --- | --- |
|  |  |  |  |  |  |  |  |  | Non-calibrated | | Calibrated | | Non-calibrated | | Calibrated | | Non-calibrated | | Calibrated | |  |
|  |  |  |  |  |  |  |  |  | $\hat{\gamma}$ | t test on $\hat{\theta_{i}}$ | $\hat{\gamma}$ | t test on $\hat{\theta_{i}}$ | $\hat{\gamma}$ | t test on $\hat{\theta_{i}}$ | $\hat{\gamma}$ | t test on $\hat{\theta_{i}}$ | $\hat{\gamma}$ | t test on $\hat{\theta_{i}}$ | $\hat{\gamma}$ | t test on $\hat{\theta_{i}}$ |  |
| 4 | 3 | 50 | 100 | 0 | 0 | 1.5 | 2 | 1 | 5.0 | 5.0 | 5.2 | 5.0 | 0.00 | 0.00 | 0.00 | 0.00 | 0.26 | 0.16 | 0.26 | 0.16 | 500 |
| 4 | 3 | 50 | 100 | 0 | 0 | 1.5 | 2 | 2 | 5.8 | 5.4 | 5.8 | 5.4 | 0.02 | 0.01 | 0.02 | 0.01 | 0.26 | 0.16 | 0.26 | 0.16 | 500 |
| 4 | 3 | 50 | 100 | 0 | 0 | 2.5 | 0.5 | 1 | 4.6 | 4.4 | 4.6 | 4.6 | 0.00 | 0.00 | 0.00 | 0.00 | 0.26 | 0.16 | 0.26 | 0.15 | 500 |
| 4 | 3 | 50 | 100 | 0 | 0 | 2.5 | 0.5 | 2 | 5.2 | 5.2 | 5.6 | 5.0 | 0.01 | 0.01 | 0.01 | 0.01 | 0.26 | 0.16 | 0.26 | 0.16 | 500 |
| 4 | 3 | 50 | 100 | 0 | 0.2 | 1.5 | 2 | 1 | 11.4 | 11.0 | 11.4 | 11.0 | 0.01 | 0.07 | 0.01 | 0.07 | 0.26 | 0.16 | 0.26 | 0.16 | 500 |
| 4 | 3 | 50 | 100 | 0 | 0.2 | 1.5 | 2 | 2 | 14.4 | 14.0 | 14.8 | 14.0 | 0.01 | 0.07 | 0.01 | 0.07 | 0.28 | 0.18 | 0.28 | 0.18 | 500 |
| 4 | 3 | 50 | 100 | 0 | 0.2 | 2.5 | 0.5 | 1 | 13.8 | 13.8 | 14.4 | 13.8 | 0.00 | 0.08 | 0.00 | 0.08 | 0.27 | 0.17 | 0.27 | 0.16 | 500 |
| 4 | 3 | 50 | 100 | 0 | 0.2 | 2.5 | 0.5 | 2 | 11.0 | 11.0 | 11.4 | 11.0 | 0.01 | 0.08 | 0.01 | 0.08 | 0.28 | 0.17 | 0.28 | 0.17 | 500 |
| 4 | 3 | 50 | 100 | 0 | 0.5 | 1.5 | 2 | 1 | 53.8 | 53.0 | 54.8 | 53.4 | 0.03 | 0.17 | 0.03 | 0.17 | 0.28 | 0.19 | 0.28 | 0.19 | 500 |
| 4 | 3 | 50 | 100 | 0 | 0.5 | 1.5 | 2 | 2 | 46.4 | 46.0 | 48.2 | 45.8 | 0.01 | 0.19 | 0.01 | 0.19 | 0.26 | 0.18 | 0.26 | 0.18 | 500 |
| 4 | 3 | 50 | 100 | 0 | 0.5 | 2.5 | 0.5 | 1 | 48.6 | 48.2 | 49.4 | 48.0 | 0.00 | 0.20 | 0.00 | 0.20 | 0.27 | 0.18 | 0.27 | 0.18 | 500 |
| 4 | 3 | 50 | 100 | 0 | 0.5 | 2.5 | 0.5 | 2 | 48.8 | 48.0 | 49.6 | 48.0 | 0.02 | 0.18 | 0.02 | 0.18 | 0.28 | 0.19 | 0.28 | 0.19 | 500 |
| 4 | 3 | 50 | 100 | 0.5 | 0 | 1.5 | 2 | 1 | 5.2 | 4.8 | 5.4 | 4.8 | 0.01 | 0.00 | 0.01 | 0.00 | 0.27 | 0.16 | 0.27 | 0.16 | 500 |
| 4 | 3 | 50 | 100 | 0.5 | 0 | 1.5 | 2 | 2 | 5.4 | 5.2 | 5.6 | 5.2 | 0.02 | 0.01 | 0.02 | 0.01 | 0.27 | 0.16 | 0.27 | 0.16 | 500 |
| 4 | 3 | 50 | 100 | 0.5 | 0 | 2.5 | 0.5 | 1 | 6.8 | 6.4 | 7.2 | 6.4 | 0.01 | 0.01 | 0.01 | 0.01 | 0.27 | 0.16 | 0.27 | 0.16 | 500 |
| 4 | 3 | 50 | 100 | 0.5 | 0 | 2.5 | 0.5 | 2 | 4.4 | 4.2 | 4.6 | 4.2 | 0.02 | 0.01 | 0.02 | 0.01 | 0.26 | 0.15 | 0.26 | 0.16 | 500 |
| 4 | 3 | 50 | 100 | 0.5 | 0.2 | 1.5 | 2 | 1 | 15.2 | 15.2 | 15.0 | 14.8 | 0.00 | 0.08 | 0.00 | 0.08 | 0.27 | 0.16 | 0.27 | 0.17 | 500 |
| 4 | 3 | 50 | 100 | 0.5 | 0.2 | 1.5 | 2 | 2 | 11.4 | 11.2 | 11.6 | 11.2 | 0.01 | 0.07 | 0.01 | 0.07 | 0.26 | 0.16 | 0.26 | 0.16 | 500 |
| 4 | 3 | 50 | 100 | 0.5 | 0.2 | 2.5 | 0.5 | 1 | 12.8 | 12.8 | 13.8 | 12.8 | 0.00 | 0.08 | 0.00 | 0.08 | 0.28 | 0.17 | 0.27 | 0.16 | 500 |
| 4 | 3 | 50 | 100 | 0.5 | 0.2 | 2.5 | 0.5 | 2 | 12.2 | 12.0 | 12.4 | 12.0 | 0.01 | 0.08 | 0.01 | 0.07 | 0.27 | 0.17 | 0.27 | 0.17 | 500 |
| 4 | 3 | 50 | 100 | 0.5 | 0.5 | 1.5 | 2 | 1 | 49.4 | 48.6 | 50.4 | 48.0 | 0.01 | 0.19 | 0.01 | 0.19 | 0.27 | 0.18 | 0.27 | 0.18 | 500 |
| 4 | 3 | 50 | 100 | 0.5 | 0.5 | 1.5 | 2 | 2 | 45.6 | 45.2 | 47.4 | 45.8 | 0.01 | 0.19 | 0.01 | 0.19 | 0.27 | 0.18 | 0.27 | 0.18 | 500 |
| 4 | 3 | 50 | 100 | 0.5 | 0.5 | 2.5 | 0.5 | 1 | 53.6 | 52.6 | 54.4 | 52.6 | 0.05 | 0.17 | 0.05 | 0.17 | 0.28 | 0.19 | 0.28 | 0.18 | 500 |
| 4 | 3 | 50 | 100 | 0.5 | 0.5 | 2.5 | 0.5 | 2 | 46.6 | 45.0 | 47.4 | 45.0 | 0.00 | 0.20 | 0.01 | 0.19 | 0.26 | 0.17 | 0.27 | 0.18 | 500 |
| 4 | 3 | 50 | 100 | 2 | 0 | 1.5 | 2 | 1 | 6.2 | 5.6 | 6.2 | 5.8 | 0.00 | 0.00 | 0.00 | 0.00 | 0.31 | 0.15 | 0.31 | 0.15 | 500 |
| 4 | 3 | 50 | 100 | 2 | 0 | 1.5 | 2 | 2 | 5.8 | 5.6 | 5.8 | 5.4 | 0.01 | 0.01 | 0.01 | 0.01 | 0.32 | 0.16 | 0.32 | 0.16 | 500 |
| 4 | 3 | 50 | 100 | 2 | 0 | 2.5 | 0.5 | 1 | 6.0 | 5.8 | 6.6 | 5.6 | 0.01 | 0.01 | 0.01 | 0.01 | 0.32 | 0.16 | 0.32 | 0.15 | 500 |
| 4 | 3 | 50 | 100 | 2 | 0 | 2.5 | 0.5 | 2 | 5.2 | 4.4 | 5.0 | 4.4 | 0.00 | 0.00 | 0.00 | 0.00 | 0.30 | 0.14 | 0.30 | 0.15 | 500 |
| 4 | 3 | 50 | 100 | 2 | 0.2 | 1.5 | 2 | 1 | 10.8 | 10.8 | 11.4 | 10.2 | 0.02 | 0.10 | 0.01 | 0.10 | 0.31 | 0.15 | 0.31 | 0.16 | 500 |
| 4 | 3 | 50 | 100 | 2 | 0.2 | 1.5 | 2 | 2 | 10.2 | 10.0 | 10.8 | 10.0 | 0.01 | 0.11 | 0.01 | 0.11 | 0.31 | 0.15 | 0.31 | 0.15 | 500 |
| 4 | 3 | 50 | 100 | 2 | 0.2 | 2.5 | 0.5 | 1 | 12.0 | 11.4 | 12.4 | 11.6 | 0.02 | 0.09 | 0.02 | 0.09 | 0.30 | 0.15 | 0.30 | 0.15 | 500 |
| 4 | 3 | 50 | 100 | 2 | 0.2 | 2.5 | 0.5 | 2 | 12.2 | 12.2 | 12.6 | 12.2 | 0.01 | 0.11 | 0.02 | 0.11 | 0.31 | 0.16 | 0.31 | 0.15 | 500 |
| 4 | 3 | 50 | 100 | 2 | 0.5 | 1.5 | 2 | 1 | 41.6 | 40.8 | 42.2 | 41.0 | 0.01 | 0.25 | 0.01 | 0.25 | 0.31 | 0.17 | 0.30 | 0.16 | 500 |
| 4 | 3 | 50 | 100 | 2 | 0.5 | 1.5 | 2 | 2 | 36.0 | 35.6 | 36.8 | 35.6 | 0.00 | 0.26 | 0.01 | 0.25 | 0.30 | 0.16 | 0.30 | 0.16 | 500 |
| 4 | 3 | 50 | 100 | 2 | 0.5 | 2.5 | 0.5 | 1 | 41.6 | 41.4 | 41.8 | 41.2 | 0.04 | 0.24 | 0.04 | 0.24 | 0.31 | 0.17 | 0.30 | 0.17 | 500 |
| 4 | 3 | 50 | 100 | 2 | 0.5 | 2.5 | 0.5 | 2 | 40.0 | 39.0 | 40.2 | 39.2 | 0.00 | 0.26 | 0.00 | 0.26 | 0.28 | 0.15 | 0.28 | 0.15 | 500 |
| 4 | 3 | 50 | 250 | 0 | 0 | 1.5 | 2 | 1 | 7.4 | 6.8 | 7.6 | 6.8 | 0.00 | 0.00 | 0.00 | 0.00 | 0.27 | 0.17 | 0.27 | 0.16 | 500 |
| 4 | 3 | 50 | 250 | 0 | 0 | 1.5 | 2 | 2 | 5.2 | 5.2 | 5.6 | 5.2 | 0.00 | 0.00 | 0.00 | 0.00 | 0.26 | 0.16 | 0.26 | 0.16 | 500 |
| 4 | 3 | 50 | 250 | 0 | 0 | 2.5 | 0.5 | 1 | 4.6 | 4.4 | 4.8 | 4.4 | 0.02 | 0.01 | 0.02 | 0.01 | 0.26 | 0.16 | 0.26 | 0.15 | 500 |
| 4 | 3 | 50 | 250 | 0 | 0 | 2.5 | 0.5 | 2 | 6.6 | 6.6 | 6.6 | 6.6 | 0.01 | 0.01 | 0.01 | 0.01 | 0.28 | 0.17 | 0.27 | 0.16 | 500 |
| 4 | 3 | 50 | 250 | 0 | 0.2 | 1.5 | 2 | 1 | 13.4 | 13.0 | 14.0 | 13.0 | 0.02 | 0.06 | 0.02 | 0.07 | 0.26 | 0.16 | 0.25 | 0.16 | 500 |
| 4 | 3 | 50 | 250 | 0 | 0.2 | 1.5 | 2 | 2 | 11.6 | 11.6 | 11.8 | 11.6 | 0.01 | 0.08 | 0.01 | 0.09 | 0.27 | 0.17 | 0.26 | 0.16 | 500 |
| 4 | 3 | 50 | 250 | 0 | 0.2 | 2.5 | 0.5 | 1 | 11.8 | 11.6 | 12.0 | 11.6 | 0.01 | 0.07 | 0.01 | 0.08 | 0.26 | 0.16 | 0.25 | 0.15 | 500 |
| 4 | 3 | 50 | 250 | 0 | 0.2 | 2.5 | 0.5 | 2 | 12.2 | 11.8 | 12.2 | 11.8 | 0.01 | 0.08 | 0.00 | 0.08 | 0.27 | 0.16 | 0.27 | 0.16 | 500 |
| 4 | 3 | 50 | 250 | 0 | 0.5 | 1.5 | 2 | 1 | 50.6 | 50.4 | 51.4 | 50.4 | 0.02 | 0.17 | 0.01 | 0.18 | 0.28 | 0.19 | 0.27 | 0.18 | 500 |
| 4 | 3 | 50 | 250 | 0 | 0.5 | 1.5 | 2 | 2 | 48.6 | 48.0 | 49.8 | 48.0 | 0.01 | 0.18 | 0.01 | 0.18 | 0.27 | 0.18 | 0.26 | 0.18 | 500 |
| 4 | 3 | 50 | 250 | 0 | 0.5 | 2.5 | 0.5 | 1 | 46.6 | 45.8 | 47.2 | 46.0 | 0.01 | 0.19 | 0.01 | 0.19 | 0.26 | 0.18 | 0.27 | 0.18 | 500 |
| 4 | 3 | 50 | 250 | 0 | 0.5 | 2.5 | 0.5 | 2 | 47.8 | 47.6 | 48.2 | 47.8 | 0.02 | 0.18 | 0.02 | 0.18 | 0.29 | 0.19 | 0.28 | 0.18 | 500 |
| 4 | 3 | 50 | 250 | 0.5 | 0 | 1.5 | 2 | 1 | 4.6 | 4.6 | 4.6 | 4.6 | 0.01 | 0.00 | 0.01 | 0.01 | 0.26 | 0.16 | 0.25 | 0.15 | 500 |
| 4 | 3 | 50 | 250 | 0.5 | 0 | 1.5 | 2 | 2 | 5.6 | 5.6 | 5.6 | 5.6 | 0.00 | 0.00 | 0.00 | 0.00 | 0.26 | 0.16 | 0.26 | 0.16 | 500 |
| 4 | 3 | 50 | 250 | 0.5 | 0 | 2.5 | 0.5 | 1 | 5.6 | 5.6 | 5.8 | 5.6 | 0.01 | 0.00 | 0.01 | 0.01 | 0.28 | 0.17 | 0.27 | 0.16 | 500 |
| 4 | 3 | 50 | 250 | 0.5 | 0 | 2.5 | 0.5 | 2 | 5.2 | 4.6 | 5.2 | 4.6 | 0.01 | 0.01 | 0.01 | 0.01 | 0.27 | 0.16 | 0.26 | 0.15 | 500 |
| 4 | 3 | 50 | 250 | 0.5 | 0.2 | 1.5 | 2 | 1 | 13.0 | 12.8 | 13.4 | 13.0 | 0.00 | 0.08 | 0.00 | 0.08 | 0.26 | 0.16 | 0.26 | 0.16 | 500 |
| 4 | 3 | 50 | 250 | 0.5 | 0.2 | 1.5 | 2 | 2 | 13.6 | 13.4 | 14.0 | 13.4 | 0.01 | 0.07 | 0.01 | 0.08 | 0.26 | 0.16 | 0.26 | 0.16 | 500 |
| 4 | 3 | 50 | 250 | 0.5 | 0.2 | 2.5 | 0.5 | 1 | 11.0 | 10.8 | 11.2 | 10.8 | 0.02 | 0.07 | 0.02 | 0.07 | 0.25 | 0.15 | 0.25 | 0.15 | 500 |
| 4 | 3 | 50 | 250 | 0.5 | 0.2 | 2.5 | 0.5 | 2 | 11.6 | 11.6 | 12.0 | 11.6 | 0.01 | 0.08 | 0.00 | 0.08 | 0.27 | 0.16 | 0.27 | 0.16 | 500 |
| 4 | 3 | 50 | 250 | 0.5 | 0.5 | 1.5 | 2 | 1 | 48.6 | 48.2 | 49.0 | 48.0 | 0.00 | 0.19 | 0.00 | 0.20 | 0.27 | 0.18 | 0.27 | 0.17 | 500 |
| 4 | 3 | 50 | 250 | 0.5 | 0.5 | 1.5 | 2 | 2 | 46.6 | 46.2 | 47.2 | 46.2 | 0.01 | 0.19 | 0.01 | 0.19 | 0.26 | 0.17 | 0.26 | 0.17 | 500 |
| 4 | 3 | 50 | 250 | 0.5 | 0.5 | 2.5 | 0.5 | 1 | 45.8 | 44.8 | 46.6 | 45.0 | 0.01 | 0.21 | 0.02 | 0.21 | 0.27 | 0.17 | 0.26 | 0.17 | 500 |
| 4 | 3 | 50 | 250 | 0.5 | 0.5 | 2.5 | 0.5 | 2 | 47.6 | 46.6 | 48.0 | 46.6 | 0.03 | 0.18 | 0.02 | 0.19 | 0.27 | 0.18 | 0.27 | 0.18 | 500 |
| 4 | 3 | 50 | 250 | 2 | 0 | 1.5 | 2 | 1 | 5.4 | 5.4 | 5.6 | 5.4 | 0.02 | 0.01 | 0.02 | 0.01 | 0.31 | 0.15 | 0.31 | 0.15 | 500 |
| 4 | 3 | 50 | 250 | 2 | 0 | 1.5 | 2 | 2 | 5.0 | 4.8 | 5.0 | 4.8 | 0.01 | 0.00 | 0.01 | 0.00 | 0.29 | 0.14 | 0.29 | 0.14 | 500 |
| 4 | 3 | 50 | 250 | 2 | 0 | 2.5 | 0.5 | 1 | 5.4 | 5.4 | 5.6 | 5.2 | 0.02 | 0.01 | 0.02 | 0.01 | 0.30 | 0.15 | 0.30 | 0.14 | 500 |
| 4 | 3 | 50 | 250 | 2 | 0 | 2.5 | 0.5 | 2 | 5.4 | 5.0 | 5.4 | 5.0 | 0.00 | 0.00 | 0.00 | 0.00 | 0.29 | 0.14 | 0.29 | 0.14 | 500 |
| 4 | 3 | 50 | 250 | 2 | 0.2 | 1.5 | 2 | 1 | 8.0 | 8.0 | 8.4 | 8.2 | 0.01 | 0.10 | 0.01 | 0.10 | 0.28 | 0.13 | 0.28 | 0.13 | 500 |
| 4 | 3 | 50 | 250 | 2 | 0.2 | 1.5 | 2 | 2 | 7.8 | 7.6 | 8.4 | 7.8 | 0.00 | 0.11 | 0.01 | 0.11 | 0.30 | 0.15 | 0.29 | 0.14 | 500 |
| 4 | 3 | 50 | 250 | 2 | 0.2 | 2.5 | 0.5 | 1 | 10.8 | 10.8 | 11.4 | 11.0 | 0.00 | 0.10 | 0.00 | 0.11 | 0.30 | 0.15 | 0.29 | 0.14 | 500 |
| 4 | 3 | 50 | 250 | 2 | 0.2 | 2.5 | 0.5 | 2 | 10.6 | 10.2 | 10.8 | 10.2 | 0.01 | 0.10 | 0.00 | 0.10 | 0.29 | 0.14 | 0.28 | 0.14 | 500 |
| 4 | 3 | 50 | 250 | 2 | 0.5 | 1.5 | 2 | 1 | 35.8 | 36.0 | 37.8 | 36.2 | 0.01 | 0.27 | 0.01 | 0.27 | 0.31 | 0.17 | 0.31 | 0.16 | 500 |
| 4 | 3 | 50 | 250 | 2 | 0.5 | 1.5 | 2 | 2 | 39.6 | 39.8 | 40.4 | 39.2 | 0.02 | 0.25 | 0.02 | 0.25 | 0.31 | 0.17 | 0.31 | 0.17 | 500 |
| 4 | 3 | 50 | 250 | 2 | 0.5 | 2.5 | 0.5 | 1 | 38.8 | 37.8 | 39.6 | 37.6 | 0.01 | 0.25 | 0.01 | 0.25 | 0.29 | 0.17 | 0.28 | 0.16 | 500 |
| 4 | 3 | 50 | 250 | 2 | 0.5 | 2.5 | 0.5 | 2 | 41.0 | 40.0 | 41.8 | 40.0 | 0.01 | 0.25 | 0.01 | 0.25 | 0.31 | 0.17 | 0.30 | 0.16 | 500 |
| 4 | 3 | 50 | 500 | 0 | 0 | 1.5 | 2 | 1 | 2.8 | 2.8 | 3.0 | 2.8 | 0.02 | 0.01 | 0.02 | 0.01 | 0.25 | 0.15 | 0.24 | 0.15 | 500 |
| 4 | 3 | 50 | 500 | 0 | 0 | 1.5 | 2 | 2 | 5.2 | 4.8 | 5.4 | 4.8 | 0.01 | 0.00 | 0.01 | 0.00 | 0.26 | 0.16 | 0.26 | 0.16 | 500 |
| 4 | 3 | 50 | 500 | 0 | 0 | 2.5 | 0.5 | 1 | 6.0 | 5.4 | 6.4 | 5.4 | 0.02 | 0.01 | 0.01 | 0.01 | 0.27 | 0.16 | 0.27 | 0.16 | 500 |
| 4 | 3 | 50 | 500 | 0 | 0 | 2.5 | 0.5 | 2 | 5.2 | 5.2 | 5.2 | 5.2 | 0.00 | 0.00 | 0.01 | 0.00 | 0.27 | 0.16 | 0.26 | 0.16 | 500 |
| 4 | 3 | 50 | 500 | 0 | 0.2 | 1.5 | 2 | 1 | 14.0 | 13.8 | 14.4 | 13.4 | 0.01 | 0.07 | 0.01 | 0.07 | 0.27 | 0.17 | 0.27 | 0.17 | 500 |
| 4 | 3 | 50 | 500 | 0 | 0.2 | 1.5 | 2 | 2 | 11.2 | 10.8 | 11.6 | 11.0 | 0.01 | 0.07 | 0.01 | 0.08 | 0.26 | 0.16 | 0.25 | 0.16 | 500 |
| 4 | 3 | 50 | 500 | 0 | 0.2 | 2.5 | 0.5 | 1 | 10.0 | 9.8 | 10.2 | 9.8 | 0.01 | 0.08 | 0.00 | 0.08 | 0.26 | 0.16 | 0.25 | 0.15 | 500 |
| 4 | 3 | 50 | 500 | 0 | 0.2 | 2.5 | 0.5 | 2 | 13.4 | 12.6 | 13.6 | 12.6 | 0.00 | 0.08 | 0.00 | 0.08 | 0.26 | 0.16 | 0.26 | 0.16 | 500 |
| 4 | 3 | 50 | 500 | 0 | 0.5 | 1.5 | 2 | 1 | 50.4 | 50.2 | 50.8 | 50.2 | 0.01 | 0.18 | 0.00 | 0.19 | 0.27 | 0.19 | 0.26 | 0.18 | 500 |
| 4 | 3 | 50 | 500 | 0 | 0.5 | 1.5 | 2 | 2 | 47.8 | 47.0 | 48.0 | 47.0 | 0.00 | 0.19 | 0.01 | 0.20 | 0.28 | 0.20 | 0.27 | 0.18 | 500 |
| 4 | 3 | 50 | 500 | 0 | 0.5 | 2.5 | 0.5 | 1 | 47.4 | 46.0 | 48.0 | 46.0 | 0.00 | 0.20 | 0.01 | 0.20 | 0.27 | 0.18 | 0.27 | 0.17 | 500 |
| 4 | 3 | 50 | 500 | 0 | 0.5 | 2.5 | 0.5 | 2 | 46.2 | 45.2 | 46.6 | 45.2 | 0.00 | 0.19 | 0.01 | 0.20 | 0.27 | 0.18 | 0.27 | 0.18 | 500 |
| 4 | 3 | 50 | 500 | 0.5 | 0 | 1.5 | 2 | 1 | 5.2 | 5.0 | 5.2 | 5.0 | 0.01 | 0.01 | 0.01 | 0.01 | 0.26 | 0.16 | 0.26 | 0.16 | 500 |
| 4 | 3 | 50 | 500 | 0.5 | 0 | 1.5 | 2 | 2 | 4.0 | 3.8 | 4.2 | 4.0 | 0.02 | 0.01 | 0.02 | 0.01 | 0.25 | 0.15 | 0.25 | 0.15 | 500 |
| 4 | 3 | 50 | 500 | 0.5 | 0 | 2.5 | 0.5 | 1 | 5.2 | 5.0 | 5.8 | 5.0 | 0.00 | 0.00 | 0.00 | 0.00 | 0.26 | 0.16 | 0.26 | 0.15 | 500 |
| 4 | 3 | 50 | 500 | 0.5 | 0 | 2.5 | 0.5 | 2 | 4.0 | 3.8 | 4.0 | 3.8 | 0.02 | 0.01 | 0.02 | 0.01 | 0.27 | 0.16 | 0.26 | 0.15 | 500 |
| 4 | 3 | 50 | 500 | 0.5 | 0.2 | 1.5 | 2 | 1 | 9.6 | 9.4 | 10.0 | 9.4 | 0.01 | 0.08 | 0.01 | 0.09 | 0.26 | 0.16 | 0.26 | 0.16 | 500 |
| 4 | 3 | 50 | 500 | 0.5 | 0.2 | 1.5 | 2 | 2 | 14.2 | 14.0 | 14.8 | 14.0 | 0.01 | 0.07 | 0.00 | 0.08 | 0.27 | 0.17 | 0.27 | 0.16 | 500 |
| 4 | 3 | 50 | 500 | 0.5 | 0.2 | 2.5 | 0.5 | 1 | 13.2 | 12.4 | 13.4 | 12.4 | 0.02 | 0.07 | 0.02 | 0.07 | 0.26 | 0.16 | 0.26 | 0.15 | 500 |
| 4 | 3 | 50 | 500 | 0.5 | 0.2 | 2.5 | 0.5 | 2 | 13.0 | 12.8 | 13.2 | 12.8 | 0.02 | 0.09 | 0.03 | 0.10 | 0.28 | 0.17 | 0.27 | 0.16 | 500 |
| 4 | 3 | 50 | 500 | 0.5 | 0.5 | 1.5 | 2 | 1 | 45.6 | 45.4 | 46.0 | 45.4 | 0.00 | 0.19 | 0.01 | 0.20 | 0.26 | 0.17 | 0.25 | 0.16 | 500 |
| 4 | 3 | 50 | 500 | 0.5 | 0.5 | 1.5 | 2 | 2 | 47.2 | 46.6 | 48.0 | 46.4 | 0.01 | 0.19 | 0.00 | 0.19 | 0.27 | 0.18 | 0.26 | 0.17 | 500 |
| 4 | 3 | 50 | 500 | 0.5 | 0.5 | 2.5 | 0.5 | 1 | 48.6 | 48.0 | 48.6 | 48.0 | 0.01 | 0.19 | 0.00 | 0.20 | 0.27 | 0.17 | 0.26 | 0.17 | 500 |
| 4 | 3 | 50 | 500 | 0.5 | 0.5 | 2.5 | 0.5 | 2 | 51.6 | 50.8 | 52.0 | 50.8 | 0.02 | 0.19 | 0.02 | 0.19 | 0.28 | 0.18 | 0.28 | 0.18 | 500 |
| 4 | 3 | 50 | 500 | 2 | 0 | 1.5 | 2 | 1 | 4.0 | 4.0 | 4.0 | 3.8 | 0.02 | 0.01 | 0.02 | 0.01 | 0.30 | 0.14 | 0.30 | 0.14 | 500 |
| 4 | 3 | 50 | 500 | 2 | 0 | 1.5 | 2 | 2 | 6.0 | 6.0 | 6.6 | 6.0 | 0.00 | 0.00 | 0.00 | 0.00 | 0.31 | 0.15 | 0.31 | 0.15 | 500 |
| 4 | 3 | 50 | 500 | 2 | 0 | 2.5 | 0.5 | 1 | 3.4 | 3.4 | 3.4 | 3.4 | 0.01 | 0.00 | 0.01 | 0.00 | 0.29 | 0.14 | 0.28 | 0.13 | 500 |
| 4 | 3 | 50 | 500 | 2 | 0 | 2.5 | 0.5 | 2 | 4.4 | 4.2 | 4.6 | 4.2 | 0.01 | 0.01 | 0.01 | 0.01 | 0.29 | 0.14 | 0.28 | 0.13 | 500 |
| 4 | 3 | 50 | 500 | 2 | 0.2 | 1.5 | 2 | 1 | 12.4 | 12.0 | 12.6 | 12.0 | 0.02 | 0.09 | 0.02 | 0.09 | 0.30 | 0.15 | 0.30 | 0.14 | 500 |
| 4 | 3 | 50 | 500 | 2 | 0.2 | 1.5 | 2 | 2 | 7.4 | 7.2 | 7.8 | 7.0 | 0.03 | 0.12 | 0.03 | 0.12 | 0.30 | 0.14 | 0.30 | 0.14 | 500 |
| 4 | 3 | 50 | 500 | 2 | 0.2 | 2.5 | 0.5 | 1 | 11.0 | 10.8 | 11.8 | 11.0 | 0.01 | 0.11 | 0.01 | 0.11 | 0.30 | 0.15 | 0.30 | 0.15 | 500 |
| 4 | 3 | 50 | 500 | 2 | 0.2 | 2.5 | 0.5 | 2 | 10.2 | 9.8 | 10.2 | 10.0 | 0.02 | 0.10 | 0.02 | 0.10 | 0.28 | 0.14 | 0.28 | 0.14 | 500 |
| 4 | 3 | 50 | 500 | 2 | 0.5 | 1.5 | 2 | 1 | 39.6 | 39.2 | 40.2 | 39.2 | 0.01 | 0.25 | 0.01 | 0.26 | 0.31 | 0.17 | 0.30 | 0.16 | 500 |
| 4 | 3 | 50 | 500 | 2 | 0.5 | 1.5 | 2 | 2 | 38.2 | 37.0 | 38.8 | 37.8 | 0.01 | 0.25 | 0.01 | 0.26 | 0.31 | 0.17 | 0.31 | 0.16 | 500 |
| 4 | 3 | 50 | 500 | 2 | 0.5 | 2.5 | 0.5 | 1 | 41.4 | 41.2 | 42.0 | 41.2 | 0.01 | 0.25 | 0.00 | 0.26 | 0.29 | 0.16 | 0.28 | 0.15 | 500 |
| 4 | 3 | 50 | 500 | 2 | 0.5 | 2.5 | 0.5 | 2 | 41.6 | 40.4 | 42.2 | 40.6 | 0.02 | 0.24 | 0.01 | 0.25 | 0.31 | 0.17 | 0.30 | 0.16 | 500 |
| 4 | 3 | 100 | 100 | 0 | 0 | 1.5 | 2 | 1 | 4.2 | 4.2 | 4.2 | 4.2 | 0.01 | 0.01 | 0.01 | 0.01 | 0.17 | 0.10 | 0.17 | 0.11 | 500 |
| 4 | 3 | 100 | 100 | 0 | 0 | 1.5 | 2 | 2 | 4.6 | 4.4 | 4.6 | 4.6 | 0.00 | 0.00 | 0.00 | 0.00 | 0.18 | 0.11 | 0.18 | 0.11 | 500 |
| 4 | 3 | 100 | 100 | 0 | 0 | 2.5 | 0.5 | 1 | 6.4 | 6.2 | 6.6 | 6.2 | 0.01 | 0.01 | 0.01 | 0.01 | 0.18 | 0.11 | 0.19 | 0.11 | 500 |
| 4 | 3 | 100 | 100 | 0 | 0 | 2.5 | 0.5 | 2 | 5.4 | 5.4 | 5.4 | 5.4 | 0.01 | 0.00 | 0.01 | 0.00 | 0.18 | 0.11 | 0.19 | 0.11 | 500 |
| 4 | 3 | 100 | 100 | 0 | 0.2 | 1.5 | 2 | 1 | 18.4 | 18.0 | 19.0 | 18.8 | 0.01 | 0.08 | 0.01 | 0.08 | 0.18 | 0.11 | 0.18 | 0.12 | 500 |
| 4 | 3 | 100 | 100 | 0 | 0.2 | 1.5 | 2 | 2 | 19.2 | 19.0 | 19.4 | 18.8 | 0.00 | 0.08 | 0.00 | 0.07 | 0.19 | 0.12 | 0.19 | 0.12 | 500 |
| 4 | 3 | 100 | 100 | 0 | 0.2 | 2.5 | 0.5 | 1 | 19.0 | 18.4 | 19.4 | 18.4 | 0.01 | 0.08 | 0.01 | 0.08 | 0.19 | 0.11 | 0.19 | 0.11 | 500 |
| 4 | 3 | 100 | 100 | 0 | 0.2 | 2.5 | 0.5 | 2 | 21.4 | 21.2 | 21.4 | 21.2 | 0.02 | 0.07 | 0.02 | 0.07 | 0.20 | 0.12 | 0.20 | 0.12 | 500 |
| 4 | 3 | 100 | 100 | 0 | 0.5 | 1.5 | 2 | 1 | 77.4 | 77.4 | 77.6 | 77.4 | 0.00 | 0.19 | 0.00 | 0.19 | 0.19 | 0.13 | 0.19 | 0.13 | 500 |
| 4 | 3 | 100 | 100 | 0 | 0.5 | 1.5 | 2 | 2 | 77.6 | 77.2 | 78.2 | 77.4 | 0.00 | 0.19 | 0.00 | 0.19 | 0.18 | 0.12 | 0.18 | 0.13 | 500 |
| 4 | 3 | 100 | 100 | 0 | 0.5 | 2.5 | 0.5 | 1 | 78.4 | 78.2 | 78.6 | 78.2 | 0.01 | 0.19 | 0.02 | 0.18 | 0.18 | 0.12 | 0.19 | 0.13 | 500 |
| 4 | 3 | 100 | 100 | 0 | 0.5 | 2.5 | 0.5 | 2 | 75.8 | 75.6 | 76.0 | 75.2 | 0.00 | 0.20 | 0.00 | 0.19 | 0.19 | 0.13 | 0.20 | 0.13 | 500 |
| 4 | 3 | 100 | 100 | 0.5 | 0 | 1.5 | 2 | 1 | 9.0 | 8.8 | 9.0 | 8.8 | 0.00 | 0.00 | 0.00 | 0.00 | 0.20 | 0.12 | 0.21 | 0.12 | 500 |
| 4 | 3 | 100 | 100 | 0.5 | 0 | 1.5 | 2 | 2 | 5.0 | 4.8 | 4.8 | 4.8 | 0.00 | 0.00 | 0.00 | 0.00 | 0.19 | 0.11 | 0.19 | 0.11 | 500 |
| 4 | 3 | 100 | 100 | 0.5 | 0 | 2.5 | 0.5 | 1 | 6.2 | 5.8 | 6.2 | 5.8 | 0.01 | 0.01 | 0.01 | 0.00 | 0.19 | 0.11 | 0.19 | 0.11 | 500 |
| 4 | 3 | 100 | 100 | 0.5 | 0 | 2.5 | 0.5 | 2 | 5.6 | 5.6 | 5.6 | 5.6 | 0.01 | 0.01 | 0.01 | 0.01 | 0.19 | 0.11 | 0.19 | 0.12 | 500 |
| 4 | 3 | 100 | 100 | 0.5 | 0.2 | 1.5 | 2 | 1 | 17.8 | 17.6 | 18.6 | 17.6 | 0.00 | 0.08 | 0.00 | 0.08 | 0.19 | 0.11 | 0.19 | 0.11 | 500 |
| 4 | 3 | 100 | 100 | 0.5 | 0.2 | 1.5 | 2 | 2 | 21.6 | 21.6 | 21.4 | 20.8 | 0.00 | 0.08 | 0.00 | 0.08 | 0.20 | 0.12 | 0.20 | 0.12 | 500 |
| 4 | 3 | 100 | 100 | 0.5 | 0.2 | 2.5 | 0.5 | 1 | 19.0 | 19.0 | 19.0 | 19.0 | 0.01 | 0.09 | 0.01 | 0.09 | 0.20 | 0.12 | 0.20 | 0.12 | 500 |
| 4 | 3 | 100 | 100 | 0.5 | 0.2 | 2.5 | 0.5 | 2 | 20.0 | 19.8 | 20.4 | 19.8 | 0.00 | 0.08 | 0.01 | 0.08 | 0.19 | 0.11 | 0.19 | 0.11 | 500 |
| 4 | 3 | 100 | 100 | 0.5 | 0.5 | 1.5 | 2 | 1 | 77.2 | 77.0 | 77.6 | 77.2 | 0.01 | 0.19 | 0.02 | 0.18 | 0.19 | 0.13 | 0.20 | 0.13 | 500 |
| 4 | 3 | 100 | 100 | 0.5 | 0.5 | 1.5 | 2 | 2 | 78.2 | 78.0 | 78.4 | 78.0 | 0.01 | 0.19 | 0.01 | 0.19 | 0.19 | 0.12 | 0.19 | 0.13 | 500 |
| 4 | 3 | 100 | 100 | 0.5 | 0.5 | 2.5 | 0.5 | 1 | 74.6 | 74.6 | 74.8 | 74.6 | 0.00 | 0.20 | 0.00 | 0.20 | 0.20 | 0.13 | 0.20 | 0.13 | 500 |
| 4 | 3 | 100 | 100 | 0.5 | 0.5 | 2.5 | 0.5 | 2 | 79.2 | 79.0 | 79.6 | 79.2 | 0.02 | 0.19 | 0.02 | 0.19 | 0.20 | 0.13 | 0.19 | 0.13 | 500 |
| 4 | 3 | 100 | 100 | 2 | 0 | 1.5 | 2 | 1 | 5.2 | 5.2 | 5.4 | 5.2 | 0.01 | 0.01 | 0.01 | 0.01 | 0.22 | 0.10 | 0.22 | 0.11 | 500 |
| 4 | 3 | 100 | 100 | 2 | 0 | 1.5 | 2 | 2 | 4.2 | 4.2 | 4.4 | 4.2 | 0.00 | 0.00 | 0.00 | 0.00 | 0.21 | 0.10 | 0.21 | 0.10 | 500 |
| 4 | 3 | 100 | 100 | 2 | 0 | 2.5 | 0.5 | 1 | 3.4 | 3.4 | 3.6 | 3.4 | 0.01 | 0.00 | 0.01 | 0.00 | 0.21 | 0.10 | 0.21 | 0.10 | 500 |
| 4 | 3 | 100 | 100 | 2 | 0 | 2.5 | 0.5 | 2 | 4.8 | 4.8 | 4.8 | 4.8 | 0.01 | 0.00 | 0.01 | 0.00 | 0.21 | 0.10 | 0.22 | 0.10 | 500 |
| 4 | 3 | 100 | 100 | 2 | 0.2 | 1.5 | 2 | 1 | 18.0 | 17.8 | 18.6 | 17.8 | 0.01 | 0.10 | 0.02 | 0.10 | 0.22 | 0.11 | 0.23 | 0.11 | 500 |
| 4 | 3 | 100 | 100 | 2 | 0.2 | 1.5 | 2 | 2 | 14.6 | 14.4 | 14.6 | 14.4 | 0.00 | 0.11 | 0.01 | 0.10 | 0.20 | 0.10 | 0.20 | 0.10 | 500 |
| 4 | 3 | 100 | 100 | 2 | 0.2 | 2.5 | 0.5 | 1 | 13.6 | 13.6 | 13.6 | 13.4 | 0.01 | 0.11 | 0.01 | 0.11 | 0.21 | 0.10 | 0.21 | 0.10 | 500 |
| 4 | 3 | 100 | 100 | 2 | 0.2 | 2.5 | 0.5 | 2 | 13.2 | 13.2 | 13.2 | 13.2 | 0.01 | 0.11 | 0.01 | 0.11 | 0.21 | 0.10 | 0.21 | 0.10 | 500 |
| 4 | 3 | 100 | 100 | 2 | 0.5 | 1.5 | 2 | 1 | 67.6 | 67.2 | 67.8 | 67.4 | 0.00 | 0.26 | 0.01 | 0.26 | 0.21 | 0.11 | 0.21 | 0.12 | 500 |
| 4 | 3 | 100 | 100 | 2 | 0.5 | 1.5 | 2 | 2 | 69.6 | 69.4 | 70.0 | 69.4 | 0.01 | 0.25 | 0.02 | 0.25 | 0.21 | 0.11 | 0.21 | 0.12 | 500 |
| 4 | 3 | 100 | 100 | 2 | 0.5 | 2.5 | 0.5 | 1 | 66.0 | 66.0 | 66.2 | 66.0 | 0.01 | 0.25 | 0.01 | 0.25 | 0.21 | 0.12 | 0.21 | 0.12 | 500 |
| 4 | 3 | 100 | 100 | 2 | 0.5 | 2.5 | 0.5 | 2 | 70.4 | 69.8 | 70.6 | 70.0 | 0.02 | 0.25 | 0.02 | 0.25 | 0.20 | 0.11 | 0.20 | 0.11 | 500 |
| 4 | 3 | 100 | 250 | 0 | 0 | 1.5 | 2 | 1 | 4.4 | 4.4 | 4.4 | 4.2 | 0.01 | 0.00 | 0.01 | 0.00 | 0.18 | 0.11 | 0.18 | 0.11 | 500 |
| 4 | 3 | 100 | 250 | 0 | 0 | 1.5 | 2 | 2 | 3.8 | 3.8 | 4.2 | 4.0 | 0.00 | 0.00 | 0.00 | 0.00 | 0.18 | 0.11 | 0.18 | 0.11 | 500 |
| 4 | 3 | 100 | 250 | 0 | 0 | 2.5 | 0.5 | 1 | 5.0 | 5.0 | 5.2 | 5.0 | 0.01 | 0.00 | 0.00 | 0.00 | 0.19 | 0.11 | 0.19 | 0.11 | 500 |
| 4 | 3 | 100 | 250 | 0 | 0 | 2.5 | 0.5 | 2 | 4.4 | 4.4 | 4.6 | 4.4 | 0.00 | 0.00 | 0.00 | 0.00 | 0.18 | 0.11 | 0.18 | 0.11 | 500 |
| 4 | 3 | 100 | 250 | 0 | 0.2 | 1.5 | 2 | 1 | 19.0 | 19.0 | 19.4 | 19.2 | 0.01 | 0.07 | 0.01 | 0.08 | 0.18 | 0.11 | 0.17 | 0.11 | 500 |
| 4 | 3 | 100 | 250 | 0 | 0.2 | 1.5 | 2 | 2 | 20.8 | 20.6 | 21.4 | 20.4 | 0.00 | 0.08 | 0.00 | 0.08 | 0.19 | 0.12 | 0.19 | 0.12 | 500 |
| 4 | 3 | 100 | 250 | 0 | 0.2 | 2.5 | 0.5 | 1 | 19.2 | 19.0 | 19.4 | 19.2 | 0.00 | 0.08 | 0.00 | 0.08 | 0.19 | 0.11 | 0.19 | 0.11 | 500 |
| 4 | 3 | 100 | 250 | 0 | 0.2 | 2.5 | 0.5 | 2 | 18.0 | 17.6 | 18.4 | 17.8 | 0.00 | 0.08 | 0.00 | 0.08 | 0.19 | 0.11 | 0.19 | 0.11 | 500 |
| 4 | 3 | 100 | 250 | 0 | 0.5 | 1.5 | 2 | 1 | 81.2 | 81.2 | 81.4 | 81.0 | 0.01 | 0.18 | 0.01 | 0.18 | 0.19 | 0.13 | 0.19 | 0.13 | 500 |
| 4 | 3 | 100 | 250 | 0 | 0.5 | 1.5 | 2 | 2 | 77.2 | 77.2 | 77.2 | 77.2 | 0.01 | 0.18 | 0.01 | 0.19 | 0.19 | 0.13 | 0.19 | 0.13 | 500 |
| 4 | 3 | 100 | 250 | 0 | 0.5 | 2.5 | 0.5 | 1 | 80.2 | 80.2 | 80.4 | 80.2 | 0.01 | 0.19 | 0.01 | 0.19 | 0.18 | 0.12 | 0.18 | 0.11 | 500 |
| 4 | 3 | 100 | 250 | 0 | 0.5 | 2.5 | 0.5 | 2 | 73.2 | 73.0 | 73.4 | 73.2 | 0.01 | 0.20 | 0.01 | 0.20 | 0.20 | 0.13 | 0.20 | 0.13 | 500 |
| 4 | 3 | 100 | 250 | 0.5 | 0 | 1.5 | 2 | 1 | 6.0 | 6.0 | 6.2 | 6.0 | 0.01 | 0.01 | 0.01 | 0.01 | 0.19 | 0.11 | 0.19 | 0.11 | 500 |
| 4 | 3 | 100 | 250 | 0.5 | 0 | 1.5 | 2 | 2 | 5.8 | 5.6 | 6.0 | 5.6 | 0.01 | 0.00 | 0.01 | 0.00 | 0.18 | 0.11 | 0.19 | 0.11 | 500 |
| 4 | 3 | 100 | 250 | 0.5 | 0 | 2.5 | 0.5 | 1 | 5.8 | 5.6 | 6.0 | 5.6 | 0.02 | 0.01 | 0.02 | 0.01 | 0.19 | 0.11 | 0.18 | 0.11 | 500 |
| 4 | 3 | 100 | 250 | 0.5 | 0 | 2.5 | 0.5 | 2 | 5.0 | 5.0 | 5.0 | 5.0 | 0.01 | 0.01 | 0.01 | 0.01 | 0.18 | 0.11 | 0.18 | 0.11 | 500 |
| 4 | 3 | 100 | 250 | 0.5 | 0.2 | 1.5 | 2 | 1 | 20.8 | 20.2 | 21.2 | 20.6 | 0.01 | 0.07 | 0.01 | 0.07 | 0.19 | 0.12 | 0.19 | 0.11 | 500 |
| 4 | 3 | 100 | 250 | 0.5 | 0.2 | 1.5 | 2 | 2 | 20.2 | 20.0 | 20.2 | 20.0 | 0.00 | 0.08 | 0.00 | 0.08 | 0.18 | 0.11 | 0.18 | 0.11 | 500 |
| 4 | 3 | 100 | 250 | 0.5 | 0.2 | 2.5 | 0.5 | 1 | 18.0 | 17.8 | 18.2 | 17.8 | 0.00 | 0.08 | 0.00 | 0.08 | 0.19 | 0.11 | 0.19 | 0.11 | 500 |
| 4 | 3 | 100 | 250 | 0.5 | 0.2 | 2.5 | 0.5 | 2 | 16.8 | 16.4 | 17.2 | 16.2 | 0.00 | 0.08 | 0.00 | 0.08 | 0.18 | 0.11 | 0.18 | 0.11 | 500 |
| 4 | 3 | 100 | 250 | 0.5 | 0.5 | 1.5 | 2 | 1 | 77.0 | 76.8 | 77.0 | 76.6 | 0.01 | 0.19 | 0.01 | 0.19 | 0.20 | 0.13 | 0.20 | 0.13 | 500 |
| 4 | 3 | 100 | 250 | 0.5 | 0.5 | 1.5 | 2 | 2 | 74.0 | 73.4 | 74.2 | 73.6 | 0.00 | 0.19 | 0.00 | 0.19 | 0.21 | 0.14 | 0.20 | 0.13 | 500 |
| 4 | 3 | 100 | 250 | 0.5 | 0.5 | 2.5 | 0.5 | 1 | 73.4 | 73.4 | 74.0 | 73.4 | 0.00 | 0.20 | 0.00 | 0.20 | 0.20 | 0.13 | 0.20 | 0.13 | 500 |
| 4 | 3 | 100 | 250 | 0.5 | 0.5 | 2.5 | 0.5 | 2 | 73.0 | 73.0 | 73.4 | 73.0 | 0.01 | 0.21 | 0.01 | 0.21 | 0.19 | 0.12 | 0.19 | 0.12 | 500 |
| 4 | 3 | 100 | 250 | 2 | 0 | 1.5 | 2 | 1 | 4.8 | 4.8 | 4.8 | 4.6 | 0.00 | 0.00 | 0.00 | 0.00 | 0.21 | 0.10 | 0.21 | 0.10 | 500 |
| 4 | 3 | 100 | 250 | 2 | 0 | 1.5 | 2 | 2 | 6.0 | 6.0 | 6.0 | 6.0 | 0.02 | 0.01 | 0.02 | 0.01 | 0.21 | 0.10 | 0.21 | 0.10 | 500 |
| 4 | 3 | 100 | 250 | 2 | 0 | 2.5 | 0.5 | 1 | 7.0 | 6.8 | 7.0 | 6.8 | 0.01 | 0.01 | 0.01 | 0.01 | 0.22 | 0.10 | 0.22 | 0.10 | 500 |
| 4 | 3 | 100 | 250 | 2 | 0 | 2.5 | 0.5 | 2 | 3.4 | 3.4 | 3.4 | 3.4 | 0.00 | 0.00 | 0.00 | 0.00 | 0.19 | 0.09 | 0.19 | 0.09 | 500 |
| 4 | 3 | 100 | 250 | 2 | 0.2 | 1.5 | 2 | 1 | 15.4 | 15.2 | 15.6 | 15.2 | 0.00 | 0.11 | 0.00 | 0.11 | 0.21 | 0.10 | 0.21 | 0.10 | 500 |
| 4 | 3 | 100 | 250 | 2 | 0.2 | 1.5 | 2 | 2 | 15.4 | 15.6 | 16.0 | 15.8 | 0.00 | 0.11 | 0.01 | 0.10 | 0.21 | 0.10 | 0.22 | 0.10 | 500 |
| 4 | 3 | 100 | 250 | 2 | 0.2 | 2.5 | 0.5 | 1 | 16.2 | 16.2 | 16.2 | 16.2 | 0.01 | 0.11 | 0.01 | 0.11 | 0.23 | 0.11 | 0.23 | 0.11 | 500 |
| 4 | 3 | 100 | 250 | 2 | 0.2 | 2.5 | 0.5 | 2 | 14.4 | 14.2 | 14.4 | 14.0 | 0.01 | 0.11 | 0.01 | 0.11 | 0.20 | 0.10 | 0.20 | 0.10 | 500 |
| 4 | 3 | 100 | 250 | 2 | 0.5 | 1.5 | 2 | 1 | 66.0 | 65.8 | 65.8 | 65.8 | 0.00 | 0.26 | 0.00 | 0.26 | 0.23 | 0.12 | 0.22 | 0.12 | 500 |
| 4 | 3 | 100 | 250 | 2 | 0.5 | 1.5 | 2 | 2 | 63.8 | 63.8 | 64.2 | 64.0 | 0.02 | 0.27 | 0.02 | 0.27 | 0.21 | 0.11 | 0.21 | 0.11 | 500 |
| 4 | 3 | 100 | 250 | 2 | 0.5 | 2.5 | 0.5 | 1 | 66.0 | 65.8 | 66.8 | 66.0 | 0.01 | 0.25 | 0.01 | 0.25 | 0.23 | 0.12 | 0.22 | 0.12 | 500 |
| 4 | 3 | 100 | 250 | 2 | 0.5 | 2.5 | 0.5 | 2 | 67.6 | 67.6 | 68.2 | 67.6 | 0.00 | 0.25 | 0.00 | 0.26 | 0.21 | 0.12 | 0.21 | 0.11 | 500 |
| 4 | 3 | 100 | 500 | 0 | 0 | 1.5 | 2 | 1 | 4.6 | 4.6 | 4.8 | 4.6 | 0.01 | 0.01 | 0.01 | 0.01 | 0.18 | 0.11 | 0.18 | 0.11 | 500 |
| 4 | 3 | 100 | 500 | 0 | 0 | 1.5 | 2 | 2 | 6.0 | 6.0 | 6.0 | 6.0 | 0.00 | 0.00 | 0.00 | 0.00 | 0.18 | 0.11 | 0.18 | 0.11 | 500 |
| 4 | 3 | 100 | 500 | 0 | 0 | 2.5 | 0.5 | 1 | 5.2 | 5.0 | 5.4 | 5.2 | 0.00 | 0.00 | 0.00 | 0.00 | 0.19 | 0.11 | 0.19 | 0.11 | 500 |
| 4 | 3 | 100 | 500 | 0 | 0 | 2.5 | 0.5 | 2 | 5.8 | 5.8 | 5.8 | 5.8 | 0.00 | 0.00 | 0.01 | 0.00 | 0.20 | 0.12 | 0.19 | 0.11 | 500 |
| 4 | 3 | 100 | 500 | 0 | 0.2 | 1.5 | 2 | 1 | 21.6 | 21.2 | 21.6 | 21.2 | 0.01 | 0.07 | 0.01 | 0.07 | 0.19 | 0.12 | 0.18 | 0.11 | 500 |
| 4 | 3 | 100 | 500 | 0 | 0.2 | 1.5 | 2 | 2 | 19.8 | 19.8 | 20.2 | 19.8 | 0.00 | 0.08 | 0.00 | 0.08 | 0.19 | 0.12 | 0.19 | 0.11 | 500 |
| 4 | 3 | 100 | 500 | 0 | 0.2 | 2.5 | 0.5 | 1 | 18.8 | 18.4 | 19.4 | 18.4 | 0.01 | 0.09 | 0.01 | 0.09 | 0.17 | 0.10 | 0.17 | 0.10 | 500 |
| 4 | 3 | 100 | 500 | 0 | 0.2 | 2.5 | 0.5 | 2 | 18.8 | 18.6 | 19.2 | 18.6 | 0.00 | 0.08 | 0.00 | 0.08 | 0.19 | 0.11 | 0.19 | 0.11 | 500 |
| 4 | 3 | 100 | 500 | 0 | 0.5 | 1.5 | 2 | 1 | 81.2 | 81.0 | 81.4 | 80.8 | 0.01 | 0.18 | 0.01 | 0.18 | 0.18 | 0.12 | 0.18 | 0.12 | 500 |
| 4 | 3 | 100 | 500 | 0 | 0.5 | 1.5 | 2 | 2 | 77.6 | 77.2 | 78.2 | 77.4 | 0.00 | 0.19 | 0.00 | 0.19 | 0.19 | 0.13 | 0.19 | 0.13 | 500 |
| 4 | 3 | 100 | 500 | 0 | 0.5 | 2.5 | 0.5 | 1 | 76.2 | 75.4 | 76.8 | 75.4 | 0.01 | 0.20 | 0.01 | 0.20 | 0.18 | 0.12 | 0.18 | 0.12 | 500 |
| 4 | 3 | 100 | 500 | 0 | 0.5 | 2.5 | 0.5 | 2 | 80.2 | 79.8 | 80.6 | 80.0 | 0.00 | 0.19 | 0.00 | 0.20 | 0.19 | 0.12 | 0.19 | 0.12 | 500 |
| 4 | 3 | 100 | 500 | 0.5 | 0 | 1.5 | 2 | 1 | 5.4 | 5.2 | 5.4 | 5.2 | 0.01 | 0.01 | 0.01 | 0.00 | 0.18 | 0.11 | 0.18 | 0.11 | 500 |
| 4 | 3 | 100 | 500 | 0.5 | 0 | 1.5 | 2 | 2 | 6.0 | 5.8 | 6.0 | 5.6 | 0.00 | 0.00 | 0.00 | 0.00 | 0.19 | 0.12 | 0.19 | 0.11 | 500 |
| 4 | 3 | 100 | 500 | 0.5 | 0 | 2.5 | 0.5 | 1 | 4.4 | 4.4 | 4.4 | 4.4 | 0.02 | 0.01 | 0.02 | 0.01 | 0.18 | 0.11 | 0.18 | 0.11 | 500 |
| 4 | 3 | 100 | 500 | 0.5 | 0 | 2.5 | 0.5 | 2 | 6.2 | 6.0 | 6.2 | 6.0 | 0.00 | 0.00 | 0.00 | 0.00 | 0.19 | 0.11 | 0.19 | 0.11 | 500 |
| 4 | 3 | 100 | 500 | 0.5 | 0.2 | 1.5 | 2 | 1 | 20.2 | 20.0 | 20.6 | 20.2 | 0.01 | 0.08 | 0.01 | 0.08 | 0.19 | 0.11 | 0.18 | 0.11 | 500 |
| 4 | 3 | 100 | 500 | 0.5 | 0.2 | 1.5 | 2 | 2 | 18.8 | 18.4 | 18.8 | 18.6 | 0.02 | 0.09 | 0.02 | 0.09 | 0.18 | 0.11 | 0.18 | 0.11 | 500 |
| 4 | 3 | 100 | 500 | 0.5 | 0.2 | 2.5 | 0.5 | 1 | 22.6 | 22.6 | 23.0 | 22.6 | 0.02 | 0.07 | 0.01 | 0.07 | 0.20 | 0.12 | 0.20 | 0.12 | 500 |
| 4 | 3 | 100 | 500 | 0.5 | 0.2 | 2.5 | 0.5 | 2 | 17.4 | 17.2 | 17.4 | 17.2 | 0.00 | 0.09 | 0.00 | 0.09 | 0.18 | 0.11 | 0.18 | 0.10 | 500 |
| 4 | 3 | 100 | 500 | 0.5 | 0.5 | 1.5 | 2 | 1 | 74.6 | 74.2 | 75.2 | 74.0 | 0.02 | 0.20 | 0.02 | 0.21 | 0.18 | 0.12 | 0.18 | 0.12 | 500 |
| 4 | 3 | 100 | 500 | 0.5 | 0.5 | 1.5 | 2 | 2 | 77.2 | 76.8 | 77.6 | 76.8 | 0.00 | 0.19 | 0.00 | 0.20 | 0.19 | 0.13 | 0.19 | 0.13 | 500 |
| 4 | 3 | 100 | 500 | 0.5 | 0.5 | 2.5 | 0.5 | 1 | 74.4 | 74.0 | 74.6 | 74.0 | 0.01 | 0.20 | 0.01 | 0.21 | 0.19 | 0.12 | 0.19 | 0.12 | 500 |
| 4 | 3 | 100 | 500 | 0.5 | 0.5 | 2.5 | 0.5 | 2 | 77.6 | 77.4 | 77.8 | 77.4 | 0.01 | 0.20 | 0.00 | 0.20 | 0.19 | 0.12 | 0.18 | 0.12 | 500 |
| 4 | 3 | 100 | 500 | 2 | 0 | 1.5 | 2 | 1 | 3.8 | 3.8 | 3.8 | 3.8 | 0.00 | 0.00 | 0.00 | 0.00 | 0.21 | 0.10 | 0.20 | 0.10 | 500 |
| 4 | 3 | 100 | 500 | 2 | 0 | 1.5 | 2 | 2 | 6.0 | 5.8 | 6.2 | 5.6 | 0.01 | 0.00 | 0.01 | 0.00 | 0.22 | 0.10 | 0.22 | 0.10 | 500 |
| 4 | 3 | 100 | 500 | 2 | 0 | 2.5 | 0.5 | 1 | 6.2 | 6.0 | 6.2 | 6.0 | 0.01 | 0.00 | 0.01 | 0.00 | 0.22 | 0.11 | 0.22 | 0.10 | 500 |
| 4 | 3 | 100 | 500 | 2 | 0 | 2.5 | 0.5 | 2 | 3.6 | 3.6 | 3.6 | 3.6 | 0.00 | 0.00 | 0.00 | 0.00 | 0.20 | 0.10 | 0.20 | 0.09 | 500 |
| 4 | 3 | 100 | 500 | 2 | 0.2 | 1.5 | 2 | 1 | 16.6 | 16.6 | 16.6 | 16.6 | 0.00 | 0.10 | 0.00 | 0.11 | 0.22 | 0.11 | 0.22 | 0.10 | 500 |
| 4 | 3 | 100 | 500 | 2 | 0.2 | 1.5 | 2 | 2 | 15.8 | 16.0 | 16.2 | 16.2 | 0.00 | 0.10 | 0.00 | 0.11 | 0.22 | 0.10 | 0.22 | 0.10 | 500 |
| 4 | 3 | 100 | 500 | 2 | 0.2 | 2.5 | 0.5 | 1 | 16.8 | 16.4 | 16.8 | 16.4 | 0.01 | 0.10 | 0.01 | 0.10 | 0.20 | 0.10 | 0.20 | 0.09 | 500 |
| 4 | 3 | 100 | 500 | 2 | 0.2 | 2.5 | 0.5 | 2 | 17.6 | 17.4 | 18.0 | 17.6 | 0.01 | 0.10 | 0.01 | 0.10 | 0.21 | 0.10 | 0.21 | 0.10 | 500 |
| 4 | 3 | 100 | 500 | 2 | 0.5 | 1.5 | 2 | 1 | 67.8 | 67.8 | 67.8 | 67.8 | 0.01 | 0.26 | 0.01 | 0.26 | 0.21 | 0.11 | 0.21 | 0.11 | 500 |
| 4 | 3 | 100 | 500 | 2 | 0.5 | 1.5 | 2 | 2 | 69.4 | 69.6 | 70.2 | 69.6 | 0.01 | 0.26 | 0.01 | 0.26 | 0.21 | 0.11 | 0.20 | 0.11 | 500 |
| 4 | 3 | 100 | 500 | 2 | 0.5 | 2.5 | 0.5 | 1 | 64.0 | 64.0 | 64.6 | 63.6 | 0.00 | 0.26 | 0.00 | 0.26 | 0.21 | 0.12 | 0.21 | 0.11 | 500 |
| 4 | 3 | 100 | 500 | 2 | 0.5 | 2.5 | 0.5 | 2 | 61.8 | 61.6 | 62.6 | 61.8 | 0.02 | 0.27 | 0.02 | 0.27 | 0.19 | 0.10 | 0.19 | 0.10 | 500 |
| 4 | 3 | 200 | 100 | 0 | 0 | 1.5 | 2 | 1 | 7.2 | 7.2 | 7.2 | 7.2 | 0.01 | 0.00 | 0.01 | 0.00 | 0.13 | 0.08 | 0.13 | 0.08 | 500 |
| 4 | 3 | 200 | 100 | 0 | 0 | 1.5 | 2 | 2 | 5.2 | 5.2 | 5.2 | 5.2 | 0.01 | 0.01 | 0.01 | 0.01 | 0.13 | 0.08 | 0.14 | 0.09 | 500 |
| 4 | 3 | 200 | 100 | 0 | 0 | 2.5 | 0.5 | 1 | 4.6 | 4.6 | 4.8 | 4.6 | 0.00 | 0.00 | 0.00 | 0.00 | 0.13 | 0.08 | 0.14 | 0.08 | 500 |
| 4 | 3 | 200 | 100 | 0 | 0 | 2.5 | 0.5 | 2 | 4.6 | 4.6 | 4.6 | 4.6 | 0.01 | 0.00 | 0.01 | 0.00 | 0.13 | 0.08 | 0.13 | 0.08 | 500 |
| 4 | 3 | 200 | 100 | 0 | 0.2 | 1.5 | 2 | 1 | 33.8 | 33.8 | 33.8 | 33.6 | 0.00 | 0.08 | 0.00 | 0.07 | 0.13 | 0.08 | 0.13 | 0.08 | 500 |
| 4 | 3 | 200 | 100 | 0 | 0.2 | 1.5 | 2 | 2 | 34.4 | 34.4 | 34.4 | 34.4 | 0.00 | 0.08 | 0.00 | 0.08 | 0.14 | 0.09 | 0.14 | 0.09 | 500 |
| 4 | 3 | 200 | 100 | 0 | 0.2 | 2.5 | 0.5 | 1 | 33.8 | 33.4 | 34.0 | 33.4 | 0.00 | 0.08 | 0.00 | 0.08 | 0.13 | 0.08 | 0.13 | 0.08 | 500 |
| 4 | 3 | 200 | 100 | 0 | 0.2 | 2.5 | 0.5 | 2 | 32.6 | 32.6 | 33.0 | 32.6 | 0.00 | 0.08 | 0.00 | 0.08 | 0.13 | 0.08 | 0.13 | 0.08 | 500 |
| 4 | 3 | 200 | 100 | 0 | 0.5 | 1.5 | 2 | 1 | 96.2 | 96.2 | 96.2 | 96.2 | 0.00 | 0.19 | 0.00 | 0.18 | 0.14 | 0.09 | 0.15 | 0.10 | 500 |
| 4 | 3 | 200 | 100 | 0 | 0.5 | 1.5 | 2 | 2 | 97.0 | 97.0 | 97.0 | 97.0 | 0.00 | 0.19 | 0.01 | 0.18 | 0.13 | 0.09 | 0.14 | 0.10 | 500 |
| 4 | 3 | 200 | 100 | 0 | 0.5 | 2.5 | 0.5 | 1 | 96.4 | 96.4 | 96.4 | 96.4 | 0.01 | 0.19 | 0.01 | 0.19 | 0.14 | 0.09 | 0.14 | 0.10 | 500 |
| 4 | 3 | 200 | 100 | 0 | 0.5 | 2.5 | 0.5 | 2 | 98.0 | 98.0 | 98.0 | 98.0 | 0.01 | 0.19 | 0.02 | 0.18 | 0.13 | 0.09 | 0.14 | 0.10 | 500 |
| 4 | 3 | 200 | 100 | 0.5 | 0 | 1.5 | 2 | 1 | 4.2 | 4.2 | 4.4 | 4.2 | 0.01 | 0.00 | 0.01 | 0.00 | 0.13 | 0.08 | 0.13 | 0.08 | 500 |
| 4 | 3 | 200 | 100 | 0.5 | 0 | 1.5 | 2 | 2 | 7.2 | 7.0 | 7.4 | 7.0 | 0.01 | 0.01 | 0.01 | 0.01 | 0.14 | 0.08 | 0.14 | 0.08 | 500 |
| 4 | 3 | 200 | 100 | 0.5 | 0 | 2.5 | 0.5 | 1 | 4.2 | 4.2 | 4.2 | 4.2 | 0.00 | 0.00 | 0.00 | 0.00 | 0.13 | 0.08 | 0.13 | 0.08 | 500 |
| 4 | 3 | 200 | 100 | 0.5 | 0 | 2.5 | 0.5 | 2 | 6.2 | 6.2 | 6.4 | 6.4 | 0.01 | 0.01 | 0.01 | 0.01 | 0.13 | 0.08 | 0.14 | 0.08 | 500 |
| 4 | 3 | 200 | 100 | 0.5 | 0.2 | 1.5 | 2 | 1 | 33.8 | 33.6 | 33.6 | 33.2 | 0.00 | 0.08 | 0.00 | 0.08 | 0.13 | 0.08 | 0.13 | 0.08 | 500 |
| 4 | 3 | 200 | 100 | 0.5 | 0.2 | 1.5 | 2 | 2 | 35.6 | 35.4 | 35.8 | 35.2 | 0.01 | 0.07 | 0.01 | 0.07 | 0.13 | 0.08 | 0.13 | 0.08 | 500 |
| 4 | 3 | 200 | 100 | 0.5 | 0.2 | 2.5 | 0.5 | 1 | 32.6 | 32.6 | 32.8 | 32.6 | 0.00 | 0.08 | 0.00 | 0.08 | 0.14 | 0.08 | 0.14 | 0.08 | 500 |
| 4 | 3 | 200 | 100 | 0.5 | 0.2 | 2.5 | 0.5 | 2 | 33.4 | 33.4 | 33.6 | 33.4 | 0.00 | 0.08 | 0.01 | 0.08 | 0.13 | 0.08 | 0.13 | 0.08 | 500 |
| 4 | 3 | 200 | 100 | 0.5 | 0.5 | 1.5 | 2 | 1 | 98.6 | 98.6 | 98.6 | 98.6 | 0.01 | 0.19 | 0.01 | 0.18 | 0.12 | 0.08 | 0.13 | 0.09 | 500 |
| 4 | 3 | 200 | 100 | 0.5 | 0.5 | 1.5 | 2 | 2 | 97.4 | 97.4 | 97.2 | 97.2 | 0.01 | 0.19 | 0.02 | 0.18 | 0.13 | 0.09 | 0.14 | 0.10 | 500 |
| 4 | 3 | 200 | 100 | 0.5 | 0.5 | 2.5 | 0.5 | 1 | 96.4 | 96.4 | 96.4 | 96.4 | 0.00 | 0.20 | 0.01 | 0.19 | 0.13 | 0.09 | 0.14 | 0.09 | 500 |
| 4 | 3 | 200 | 100 | 0.5 | 0.5 | 2.5 | 0.5 | 2 | 96.6 | 96.6 | 96.6 | 96.6 | 0.01 | 0.20 | 0.01 | 0.19 | 0.13 | 0.09 | 0.15 | 0.10 | 500 |
| 4 | 3 | 200 | 100 | 2 | 0 | 1.5 | 2 | 1 | 3.6 | 3.6 | 3.6 | 3.6 | 0.01 | 0.00 | 0.01 | 0.00 | 0.14 | 0.07 | 0.14 | 0.07 | 500 |
| 4 | 3 | 200 | 100 | 2 | 0 | 1.5 | 2 | 2 | 4.8 | 4.8 | 4.8 | 4.8 | 0.01 | 0.00 | 0.00 | 0.00 | 0.15 | 0.07 | 0.15 | 0.07 | 500 |
| 4 | 3 | 200 | 100 | 2 | 0 | 2.5 | 0.5 | 1 | 5.2 | 5.2 | 5.2 | 5.0 | 0.01 | 0.00 | 0.01 | 0.00 | 0.15 | 0.07 | 0.15 | 0.07 | 500 |
| 4 | 3 | 200 | 100 | 2 | 0 | 2.5 | 0.5 | 2 | 4.4 | 4.4 | 4.4 | 4.4 | 0.00 | 0.00 | 0.01 | 0.00 | 0.15 | 0.07 | 0.15 | 0.07 | 500 |
| 4 | 3 | 200 | 100 | 2 | 0.2 | 1.5 | 2 | 1 | 24.6 | 24.4 | 24.6 | 24.6 | 0.01 | 0.11 | 0.00 | 0.11 | 0.15 | 0.07 | 0.15 | 0.08 | 500 |
| 4 | 3 | 200 | 100 | 2 | 0.2 | 1.5 | 2 | 2 | 26.2 | 26.2 | 26.6 | 26.2 | 0.00 | 0.11 | 0.00 | 0.10 | 0.15 | 0.07 | 0.15 | 0.08 | 500 |
| 4 | 3 | 200 | 100 | 2 | 0.2 | 2.5 | 0.5 | 1 | 27.8 | 27.8 | 28.0 | 27.6 | 0.00 | 0.11 | 0.00 | 0.10 | 0.15 | 0.07 | 0.15 | 0.07 | 500 |
| 4 | 3 | 200 | 100 | 2 | 0.2 | 2.5 | 0.5 | 2 | 28.0 | 28.0 | 28.2 | 28.0 | 0.01 | 0.10 | 0.01 | 0.10 | 0.15 | 0.07 | 0.15 | 0.07 | 500 |
| 4 | 3 | 200 | 100 | 2 | 0.5 | 1.5 | 2 | 1 | 92.2 | 92.2 | 92.0 | 91.8 | 0.00 | 0.26 | 0.01 | 0.25 | 0.15 | 0.08 | 0.16 | 0.09 | 500 |
| 4 | 3 | 200 | 100 | 2 | 0.5 | 1.5 | 2 | 2 | 90.4 | 90.4 | 91.0 | 90.8 | 0.01 | 0.26 | 0.02 | 0.25 | 0.15 | 0.08 | 0.17 | 0.10 | 500 |
| 4 | 3 | 200 | 100 | 2 | 0.5 | 2.5 | 0.5 | 1 | 92.4 | 92.2 | 92.4 | 92.2 | 0.00 | 0.26 | 0.00 | 0.25 | 0.14 | 0.08 | 0.15 | 0.08 | 500 |
| 4 | 3 | 200 | 100 | 2 | 0.5 | 2.5 | 0.5 | 2 | 93.8 | 93.8 | 93.8 | 93.8 | 0.00 | 0.26 | 0.00 | 0.26 | 0.14 | 0.07 | 0.14 | 0.08 | 500 |
| 4 | 3 | 200 | 250 | 0 | 0 | 1.5 | 2 | 1 | 5.6 | 5.6 | 5.6 | 5.6 | 0.01 | 0.00 | 0.00 | 0.00 | 0.14 | 0.08 | 0.14 | 0.08 | 500 |
| 4 | 3 | 200 | 250 | 0 | 0 | 1.5 | 2 | 2 | 7.2 | 7.2 | 7.2 | 7.0 | 0.00 | 0.00 | 0.00 | 0.00 | 0.14 | 0.08 | 0.14 | 0.08 | 500 |
| 4 | 3 | 200 | 250 | 0 | 0 | 2.5 | 0.5 | 1 | 4.6 | 4.6 | 4.6 | 4.6 | 0.00 | 0.00 | 0.00 | 0.00 | 0.13 | 0.08 | 0.13 | 0.08 | 500 |
| 4 | 3 | 200 | 250 | 0 | 0 | 2.5 | 0.5 | 2 | 4.8 | 4.8 | 4.8 | 4.6 | 0.01 | 0.00 | 0.01 | 0.00 | 0.13 | 0.08 | 0.13 | 0.08 | 500 |
| 4 | 3 | 200 | 250 | 0 | 0.2 | 1.5 | 2 | 1 | 32.8 | 32.8 | 32.8 | 32.6 | 0.01 | 0.08 | 0.01 | 0.08 | 0.13 | 0.08 | 0.13 | 0.08 | 500 |
| 4 | 3 | 200 | 250 | 0 | 0.2 | 1.5 | 2 | 2 | 35.8 | 35.8 | 35.8 | 35.4 | 0.01 | 0.07 | 0.01 | 0.07 | 0.13 | 0.08 | 0.13 | 0.08 | 500 |
| 4 | 3 | 200 | 250 | 0 | 0.2 | 2.5 | 0.5 | 1 | 30.6 | 30.6 | 30.8 | 30.6 | 0.00 | 0.08 | 0.00 | 0.08 | 0.13 | 0.08 | 0.13 | 0.08 | 500 |
| 4 | 3 | 200 | 250 | 0 | 0.2 | 2.5 | 0.5 | 2 | 37.2 | 37.2 | 37.2 | 37.2 | 0.01 | 0.08 | 0.01 | 0.08 | 0.13 | 0.08 | 0.13 | 0.08 | 500 |
| 4 | 3 | 200 | 250 | 0 | 0.5 | 1.5 | 2 | 1 | 99.2 | 99.2 | 99.2 | 99.2 | 0.01 | 0.18 | 0.01 | 0.18 | 0.12 | 0.09 | 0.13 | 0.09 | 500 |
| 4 | 3 | 200 | 250 | 0 | 0.5 | 1.5 | 2 | 2 | 97.4 | 97.4 | 97.4 | 97.4 | 0.00 | 0.19 | 0.00 | 0.19 | 0.13 | 0.09 | 0.13 | 0.09 | 500 |
| 4 | 3 | 200 | 250 | 0 | 0.5 | 2.5 | 0.5 | 1 | 96.0 | 95.8 | 96.0 | 95.8 | 0.00 | 0.20 | 0.00 | 0.20 | 0.13 | 0.09 | 0.14 | 0.09 | 500 |
| 4 | 3 | 200 | 250 | 0 | 0.5 | 2.5 | 0.5 | 2 | 97.6 | 97.6 | 97.6 | 97.6 | 0.01 | 0.19 | 0.01 | 0.19 | 0.13 | 0.09 | 0.13 | 0.09 | 500 |
| 4 | 3 | 200 | 250 | 0.5 | 0 | 1.5 | 2 | 1 | 4.4 | 4.4 | 4.6 | 4.4 | 0.00 | 0.00 | 0.00 | 0.00 | 0.13 | 0.08 | 0.13 | 0.08 | 500 |
| 4 | 3 | 200 | 250 | 0.5 | 0 | 1.5 | 2 | 2 | 5.6 | 5.4 | 5.6 | 5.6 | 0.01 | 0.00 | 0.00 | 0.00 | 0.13 | 0.08 | 0.13 | 0.08 | 500 |
| 4 | 3 | 200 | 250 | 0.5 | 0 | 2.5 | 0.5 | 1 | 3.4 | 3.4 | 3.4 | 3.4 | 0.01 | 0.00 | 0.01 | 0.00 | 0.13 | 0.07 | 0.13 | 0.07 | 500 |
| 4 | 3 | 200 | 250 | 0.5 | 0 | 2.5 | 0.5 | 2 | 4.2 | 4.2 | 4.2 | 4.2 | 0.00 | 0.00 | 0.00 | 0.00 | 0.13 | 0.08 | 0.13 | 0.08 | 500 |
| 4 | 3 | 200 | 250 | 0.5 | 0.2 | 1.5 | 2 | 1 | 32.8 | 32.8 | 33.2 | 33.0 | 0.00 | 0.08 | 0.00 | 0.08 | 0.13 | 0.08 | 0.13 | 0.08 | 500 |
| 4 | 3 | 200 | 250 | 0.5 | 0.2 | 1.5 | 2 | 2 | 30.2 | 30.2 | 30.2 | 30.2 | 0.00 | 0.08 | 0.00 | 0.08 | 0.13 | 0.08 | 0.13 | 0.08 | 500 |
| 4 | 3 | 200 | 250 | 0.5 | 0.2 | 2.5 | 0.5 | 1 | 31.4 | 31.4 | 31.8 | 31.4 | 0.01 | 0.09 | 0.01 | 0.09 | 0.13 | 0.08 | 0.13 | 0.08 | 500 |
| 4 | 3 | 200 | 250 | 0.5 | 0.2 | 2.5 | 0.5 | 2 | 27.6 | 27.4 | 28.0 | 27.4 | 0.01 | 0.09 | 0.01 | 0.09 | 0.12 | 0.07 | 0.12 | 0.08 | 500 |
| 4 | 3 | 200 | 250 | 0.5 | 0.5 | 1.5 | 2 | 1 | 97.2 | 97.0 | 97.2 | 97.0 | 0.00 | 0.19 | 0.00 | 0.19 | 0.13 | 0.09 | 0.13 | 0.09 | 500 |
| 4 | 3 | 200 | 250 | 0.5 | 0.5 | 1.5 | 2 | 2 | 97.6 | 97.6 | 97.8 | 97.6 | 0.01 | 0.19 | 0.01 | 0.19 | 0.14 | 0.09 | 0.14 | 0.09 | 500 |
| 4 | 3 | 200 | 250 | 0.5 | 0.5 | 2.5 | 0.5 | 1 | 97.2 | 97.2 | 97.2 | 97.2 | 0.00 | 0.20 | 0.00 | 0.20 | 0.13 | 0.09 | 0.13 | 0.09 | 500 |
| 4 | 3 | 200 | 250 | 0.5 | 0.5 | 2.5 | 0.5 | 2 | 95.8 | 95.6 | 95.8 | 95.6 | 0.00 | 0.20 | 0.00 | 0.20 | 0.14 | 0.09 | 0.14 | 0.09 | 500 |
| 4 | 3 | 200 | 250 | 2 | 0 | 1.5 | 2 | 1 | 5.8 | 5.6 | 5.6 | 5.4 | 0.01 | 0.00 | 0.01 | 0.00 | 0.15 | 0.07 | 0.15 | 0.07 | 500 |
| 4 | 3 | 200 | 250 | 2 | 0 | 1.5 | 2 | 2 | 4.6 | 4.4 | 4.4 | 4.4 | 0.00 | 0.00 | 0.00 | 0.00 | 0.14 | 0.07 | 0.14 | 0.07 | 500 |
| 4 | 3 | 200 | 250 | 2 | 0 | 2.5 | 0.5 | 1 | 4.6 | 4.6 | 4.8 | 4.6 | 0.01 | 0.00 | 0.01 | 0.00 | 0.15 | 0.07 | 0.14 | 0.07 | 500 |
| 4 | 3 | 200 | 250 | 2 | 0 | 2.5 | 0.5 | 2 | 5.2 | 5.2 | 5.2 | 5.0 | 0.01 | 0.01 | 0.01 | 0.01 | 0.15 | 0.07 | 0.15 | 0.07 | 500 |
| 4 | 3 | 200 | 250 | 2 | 0.2 | 1.5 | 2 | 1 | 27.6 | 27.2 | 27.6 | 27.2 | 0.01 | 0.11 | 0.00 | 0.11 | 0.15 | 0.07 | 0.15 | 0.07 | 500 |
| 4 | 3 | 200 | 250 | 2 | 0.2 | 1.5 | 2 | 2 | 27.4 | 27.4 | 27.4 | 27.2 | 0.01 | 0.11 | 0.00 | 0.11 | 0.15 | 0.07 | 0.15 | 0.07 | 500 |
| 4 | 3 | 200 | 250 | 2 | 0.2 | 2.5 | 0.5 | 1 | 25.6 | 25.6 | 25.6 | 25.6 | 0.00 | 0.11 | 0.00 | 0.11 | 0.14 | 0.07 | 0.14 | 0.07 | 500 |
| 4 | 3 | 200 | 250 | 2 | 0.2 | 2.5 | 0.5 | 2 | 31.6 | 31.6 | 31.8 | 31.6 | 0.01 | 0.10 | 0.01 | 0.10 | 0.15 | 0.07 | 0.15 | 0.07 | 500 |
| 4 | 3 | 200 | 250 | 2 | 0.5 | 1.5 | 2 | 1 | 93.6 | 93.6 | 93.4 | 93.4 | 0.02 | 0.25 | 0.02 | 0.25 | 0.16 | 0.08 | 0.16 | 0.09 | 500 |
| 4 | 3 | 200 | 250 | 2 | 0.5 | 1.5 | 2 | 2 | 92.4 | 92.4 | 92.4 | 92.4 | 0.01 | 0.26 | 0.01 | 0.26 | 0.15 | 0.08 | 0.15 | 0.08 | 500 |
| 4 | 3 | 200 | 250 | 2 | 0.5 | 2.5 | 0.5 | 1 | 94.0 | 93.8 | 94.0 | 93.8 | 0.01 | 0.25 | 0.01 | 0.25 | 0.15 | 0.08 | 0.15 | 0.08 | 500 |
| 4 | 3 | 200 | 250 | 2 | 0.5 | 2.5 | 0.5 | 2 | 93.0 | 92.8 | 93.0 | 92.8 | 0.02 | 0.25 | 0.02 | 0.25 | 0.15 | 0.08 | 0.15 | 0.08 | 500 |
| 4 | 3 | 200 | 500 | 0 | 0 | 1.5 | 2 | 1 | 6.4 | 6.2 | 6.4 | 6.2 | 0.00 | 0.00 | 0.00 | 0.00 | 0.13 | 0.08 | 0.13 | 0.08 | 500 |
| 4 | 3 | 200 | 500 | 0 | 0 | 1.5 | 2 | 2 | 4.8 | 4.8 | 4.8 | 4.8 | 0.00 | 0.00 | 0.00 | 0.00 | 0.13 | 0.08 | 0.13 | 0.08 | 500 |
| 4 | 3 | 200 | 500 | 0 | 0 | 2.5 | 0.5 | 1 | 4.6 | 4.6 | 4.6 | 4.6 | 0.00 | 0.00 | 0.00 | 0.00 | 0.13 | 0.08 | 0.13 | 0.08 | 500 |
| 4 | 3 | 200 | 500 | 0 | 0 | 2.5 | 0.5 | 2 | 3.8 | 3.8 | 4.0 | 3.6 | 0.00 | 0.00 | 0.00 | 0.00 | 0.13 | 0.08 | 0.13 | 0.08 | 500 |
| 4 | 3 | 200 | 500 | 0 | 0.2 | 1.5 | 2 | 1 | 37.6 | 37.4 | 38.0 | 37.6 | 0.01 | 0.07 | 0.01 | 0.07 | 0.13 | 0.08 | 0.13 | 0.08 | 500 |
| 4 | 3 | 200 | 500 | 0 | 0.2 | 1.5 | 2 | 2 | 34.6 | 34.6 | 35.2 | 34.4 | 0.00 | 0.08 | 0.00 | 0.08 | 0.13 | 0.08 | 0.13 | 0.08 | 500 |
| 4 | 3 | 200 | 500 | 0 | 0.2 | 2.5 | 0.5 | 1 | 38.4 | 37.8 | 38.4 | 37.8 | 0.01 | 0.08 | 0.01 | 0.08 | 0.13 | 0.08 | 0.13 | 0.08 | 500 |
| 4 | 3 | 200 | 500 | 0 | 0.2 | 2.5 | 0.5 | 2 | 35.0 | 35.0 | 35.0 | 35.0 | 0.00 | 0.08 | 0.00 | 0.08 | 0.13 | 0.08 | 0.13 | 0.08 | 500 |
| 4 | 3 | 200 | 500 | 0 | 0.5 | 1.5 | 2 | 1 | 97.2 | 97.2 | 97.2 | 97.2 | 0.00 | 0.19 | 0.00 | 0.19 | 0.13 | 0.08 | 0.13 | 0.08 | 500 |
| 4 | 3 | 200 | 500 | 0 | 0.5 | 1.5 | 2 | 2 | 96.6 | 96.6 | 96.6 | 96.6 | 0.01 | 0.19 | 0.01 | 0.18 | 0.13 | 0.09 | 0.13 | 0.09 | 500 |
| 4 | 3 | 200 | 500 | 0 | 0.5 | 2.5 | 0.5 | 1 | 96.0 | 95.8 | 96.0 | 95.8 | 0.01 | 0.19 | 0.01 | 0.19 | 0.14 | 0.09 | 0.14 | 0.09 | 500 |
| 4 | 3 | 200 | 500 | 0 | 0.5 | 2.5 | 0.5 | 2 | 97.6 | 97.6 | 97.8 | 97.6 | 0.00 | 0.20 | 0.00 | 0.20 | 0.13 | 0.09 | 0.13 | 0.09 | 500 |
| 4 | 3 | 200 | 500 | 0.5 | 0 | 1.5 | 2 | 1 | 5.0 | 5.0 | 5.0 | 5.0 | 0.00 | 0.00 | 0.00 | 0.00 | 0.13 | 0.08 | 0.13 | 0.08 | 500 |
| 4 | 3 | 200 | 500 | 0.5 | 0 | 1.5 | 2 | 2 | 5.2 | 5.2 | 5.4 | 5.2 | 0.01 | 0.00 | 0.01 | 0.00 | 0.13 | 0.08 | 0.13 | 0.08 | 500 |
| 4 | 3 | 200 | 500 | 0.5 | 0 | 2.5 | 0.5 | 1 | 7.2 | 6.8 | 7.2 | 6.8 | 0.00 | 0.00 | 0.00 | 0.00 | 0.14 | 0.08 | 0.14 | 0.08 | 500 |
| 4 | 3 | 200 | 500 | 0.5 | 0 | 2.5 | 0.5 | 2 | 6.4 | 6.4 | 6.4 | 6.4 | 0.00 | 0.00 | 0.00 | 0.00 | 0.14 | 0.08 | 0.14 | 0.08 | 500 |
| 4 | 3 | 200 | 500 | 0.5 | 0.2 | 1.5 | 2 | 1 | 33.2 | 32.8 | 33.2 | 32.8 | 0.00 | 0.08 | 0.00 | 0.08 | 0.12 | 0.08 | 0.12 | 0.08 | 500 |
| 4 | 3 | 200 | 500 | 0.5 | 0.2 | 1.5 | 2 | 2 | 33.2 | 33.2 | 33.4 | 33.4 | 0.01 | 0.08 | 0.01 | 0.09 | 0.13 | 0.08 | 0.13 | 0.08 | 500 |
| 4 | 3 | 200 | 500 | 0.5 | 0.2 | 2.5 | 0.5 | 1 | 31.4 | 31.2 | 31.8 | 31.4 | 0.00 | 0.09 | 0.00 | 0.09 | 0.12 | 0.07 | 0.13 | 0.07 | 500 |
| 4 | 3 | 200 | 500 | 0.5 | 0.2 | 2.5 | 0.5 | 2 | 32.2 | 32.2 | 32.2 | 32.2 | 0.00 | 0.08 | 0.00 | 0.08 | 0.13 | 0.08 | 0.13 | 0.08 | 500 |
| 4 | 3 | 200 | 500 | 0.5 | 0.5 | 1.5 | 2 | 1 | 98.0 | 97.8 | 98.0 | 98.0 | 0.01 | 0.19 | 0.01 | 0.19 | 0.13 | 0.09 | 0.13 | 0.09 | 500 |
| 4 | 3 | 200 | 500 | 0.5 | 0.5 | 1.5 | 2 | 2 | 97.4 | 97.4 | 97.4 | 97.4 | 0.00 | 0.19 | 0.00 | 0.20 | 0.12 | 0.08 | 0.12 | 0.08 | 500 |
| 4 | 3 | 200 | 500 | 0.5 | 0.5 | 2.5 | 0.5 | 1 | 97.0 | 96.8 | 97.0 | 97.0 | 0.01 | 0.20 | 0.00 | 0.20 | 0.13 | 0.09 | 0.13 | 0.08 | 500 |
| 4 | 3 | 200 | 500 | 0.5 | 0.5 | 2.5 | 0.5 | 2 | 98.4 | 98.4 | 98.4 | 98.4 | 0.00 | 0.20 | 0.00 | 0.20 | 0.13 | 0.08 | 0.13 | 0.08 | 500 |
| 4 | 3 | 200 | 500 | 2 | 0 | 1.5 | 2 | 1 | 5.2 | 5.2 | 5.0 | 5.0 | 0.00 | 0.00 | 0.00 | 0.00 | 0.15 | 0.07 | 0.15 | 0.07 | 500 |
| 4 | 3 | 200 | 500 | 2 | 0 | 1.5 | 2 | 2 | 6.0 | 6.0 | 6.2 | 5.8 | 0.01 | 0.00 | 0.01 | 0.00 | 0.16 | 0.07 | 0.16 | 0.07 | 500 |
| 4 | 3 | 200 | 500 | 2 | 0 | 2.5 | 0.5 | 1 | 6.6 | 6.6 | 6.6 | 6.6 | 0.00 | 0.00 | 0.00 | 0.00 | 0.15 | 0.07 | 0.15 | 0.07 | 500 |
| 4 | 3 | 200 | 500 | 2 | 0 | 2.5 | 0.5 | 2 | 5.2 | 5.2 | 5.2 | 5.2 | 0.00 | 0.00 | 0.00 | 0.00 | 0.14 | 0.07 | 0.14 | 0.07 | 500 |
| 4 | 3 | 200 | 500 | 2 | 0.2 | 1.5 | 2 | 1 | 29.6 | 29.6 | 29.8 | 29.6 | 0.00 | 0.10 | 0.01 | 0.10 | 0.15 | 0.07 | 0.15 | 0.07 | 500 |
| 4 | 3 | 200 | 500 | 2 | 0.2 | 1.5 | 2 | 2 | 29.4 | 29.4 | 29.6 | 29.4 | 0.01 | 0.10 | 0.01 | 0.10 | 0.16 | 0.07 | 0.15 | 0.07 | 500 |
| 4 | 3 | 200 | 500 | 2 | 0.2 | 2.5 | 0.5 | 1 | 24.2 | 24.2 | 24.2 | 24.2 | 0.01 | 0.11 | 0.01 | 0.11 | 0.14 | 0.07 | 0.14 | 0.07 | 500 |
| 4 | 3 | 200 | 500 | 2 | 0.2 | 2.5 | 0.5 | 2 | 28.0 | 28.0 | 28.0 | 28.0 | 0.01 | 0.10 | 0.00 | 0.10 | 0.14 | 0.07 | 0.14 | 0.07 | 500 |
| 4 | 3 | 200 | 500 | 2 | 0.5 | 1.5 | 2 | 1 | 92.6 | 92.6 | 92.6 | 92.6 | 0.01 | 0.25 | 0.01 | 0.25 | 0.16 | 0.09 | 0.16 | 0.09 | 500 |
| 4 | 3 | 200 | 500 | 2 | 0.5 | 1.5 | 2 | 2 | 92.8 | 92.8 | 93.0 | 92.8 | 0.00 | 0.26 | 0.00 | 0.26 | 0.15 | 0.08 | 0.15 | 0.08 | 500 |
| 4 | 3 | 200 | 500 | 2 | 0.5 | 2.5 | 0.5 | 1 | 91.8 | 91.8 | 91.8 | 91.8 | 0.00 | 0.26 | 0.00 | 0.26 | 0.15 | 0.08 | 0.15 | 0.08 | 500 |
| 4 | 3 | 200 | 500 | 2 | 0.5 | 2.5 | 0.5 | 2 | 93.6 | 93.6 | 93.6 | 93.6 | 0.01 | 0.26 | 0.00 | 0.26 | 0.15 | 0.08 | 0.15 | 0.08 | 500 |
| 4 | 3 | 500 | 100 | 0 | 0 | 1.5 | 2 | 1 | 3.8 | 3.6 | 4.0 | 4.0 | 0.00 | 0.00 | 0.00 | 0.00 | 0.08 | 0.05 | 0.08 | 0.05 | 500 |
| 4 | 3 | 500 | 100 | 0 | 0 | 1.5 | 2 | 2 | 5.8 | 5.8 | 6.0 | 5.8 | 0.00 | 0.00 | 0.00 | 0.00 | 0.09 | 0.05 | 0.09 | 0.05 | 500 |
| 4 | 3 | 500 | 100 | 0 | 0 | 2.5 | 0.5 | 1 | 4.4 | 4.4 | 4.4 | 4.4 | 0.01 | 0.00 | 0.01 | 0.00 | 0.08 | 0.05 | 0.08 | 0.05 | 500 |
| 4 | 3 | 500 | 100 | 0 | 0 | 2.5 | 0.5 | 2 | 5.4 | 5.4 | 5.4 | 5.4 | 0.00 | 0.00 | 0.00 | 0.00 | 0.08 | 0.05 | 0.08 | 0.05 | 500 |
| 4 | 3 | 500 | 100 | 0 | 0.2 | 1.5 | 2 | 1 | 69.0 | 69.0 | 69.0 | 69.0 | 0.00 | 0.08 | 0.00 | 0.08 | 0.08 | 0.05 | 0.09 | 0.05 | 500 |
| 4 | 3 | 500 | 100 | 0 | 0.2 | 1.5 | 2 | 2 | 69.4 | 69.4 | 69.6 | 69.2 | 0.00 | 0.08 | 0.00 | 0.08 | 0.08 | 0.05 | 0.08 | 0.05 | 500 |
| 4 | 3 | 500 | 100 | 0 | 0.2 | 2.5 | 0.5 | 1 | 71.8 | 71.4 | 71.6 | 71.2 | 0.00 | 0.08 | 0.01 | 0.08 | 0.08 | 0.05 | 0.08 | 0.05 | 500 |
| 4 | 3 | 500 | 100 | 0 | 0.2 | 2.5 | 0.5 | 2 | 67.4 | 67.4 | 67.6 | 67.6 | 0.00 | 0.08 | 0.01 | 0.08 | 0.08 | 0.05 | 0.09 | 0.05 | 500 |
| 4 | 3 | 500 | 100 | 0 | 0.5 | 1.5 | 2 | 1 | 100.0 | 100.0 | 100.0 | 100.0 | 0.01 | 0.20 | 0.00 | 0.19 | 0.08 | 0.06 | 0.09 | 0.07 | 500 |
| 4 | 3 | 500 | 100 | 0 | 0.5 | 1.5 | 2 | 2 | 100.0 | 100.0 | 100.0 | 100.0 | 0.00 | 0.19 | 0.01 | 0.18 | 0.08 | 0.05 | 0.09 | 0.07 | 500 |
| 4 | 3 | 500 | 100 | 0 | 0.5 | 2.5 | 0.5 | 1 | 100.0 | 100.0 | 100.0 | 100.0 | 0.00 | 0.20 | 0.01 | 0.19 | 0.08 | 0.05 | 0.10 | 0.07 | 500 |
| 4 | 3 | 500 | 100 | 0 | 0.5 | 2.5 | 0.5 | 2 | 100.0 | 100.0 | 100.0 | 100.0 | 0.00 | 0.20 | 0.01 | 0.19 | 0.09 | 0.06 | 0.09 | 0.07 | 500 |
| 4 | 3 | 500 | 100 | 0.5 | 0 | 1.5 | 2 | 1 | 5.0 | 5.0 | 5.2 | 5.0 | 0.00 | 0.00 | 0.00 | 0.00 | 0.08 | 0.05 | 0.08 | 0.05 | 500 |
| 4 | 3 | 500 | 100 | 0.5 | 0 | 1.5 | 2 | 2 | 4.6 | 4.4 | 4.6 | 4.6 | 0.00 | 0.00 | 0.00 | 0.00 | 0.08 | 0.05 | 0.08 | 0.05 | 500 |
| 4 | 3 | 500 | 100 | 0.5 | 0 | 2.5 | 0.5 | 1 | 6.0 | 6.0 | 6.0 | 6.0 | 0.00 | 0.00 | 0.00 | 0.00 | 0.09 | 0.05 | 0.09 | 0.05 | 500 |
| 4 | 3 | 500 | 100 | 0.5 | 0 | 2.5 | 0.5 | 2 | 5.8 | 5.8 | 5.8 | 5.8 | 0.01 | 0.00 | 0.01 | 0.00 | 0.08 | 0.05 | 0.08 | 0.05 | 500 |
| 4 | 3 | 500 | 100 | 0.5 | 0.2 | 1.5 | 2 | 1 | 68.8 | 68.8 | 68.8 | 68.8 | 0.00 | 0.08 | 0.00 | 0.08 | 0.08 | 0.05 | 0.08 | 0.05 | 500 |
| 4 | 3 | 500 | 100 | 0.5 | 0.2 | 1.5 | 2 | 2 | 66.6 | 66.4 | 66.6 | 66.4 | 0.00 | 0.08 | 0.00 | 0.08 | 0.08 | 0.05 | 0.08 | 0.05 | 500 |
| 4 | 3 | 500 | 100 | 0.5 | 0.2 | 2.5 | 0.5 | 1 | 64.8 | 64.8 | 64.8 | 64.8 | 0.00 | 0.08 | 0.00 | 0.08 | 0.08 | 0.05 | 0.08 | 0.05 | 500 |
| 4 | 3 | 500 | 100 | 0.5 | 0.2 | 2.5 | 0.5 | 2 | 67.2 | 67.2 | 67.2 | 67.2 | 0.00 | 0.08 | 0.00 | 0.08 | 0.09 | 0.05 | 0.09 | 0.06 | 500 |
| 4 | 3 | 500 | 100 | 0.5 | 0.5 | 1.5 | 2 | 1 | 100.0 | 100.0 | 100.0 | 100.0 | 0.00 | 0.20 | 0.01 | 0.19 | 0.08 | 0.05 | 0.09 | 0.07 | 500 |
| 4 | 3 | 500 | 100 | 0.5 | 0.5 | 1.5 | 2 | 2 | 100.0 | 100.0 | 100.0 | 100.0 | 0.00 | 0.19 | 0.01 | 0.18 | 0.08 | 0.06 | 0.09 | 0.06 | 500 |
| 4 | 3 | 500 | 100 | 0.5 | 0.5 | 2.5 | 0.5 | 1 | 100.0 | 100.0 | 100.0 | 100.0 | 0.00 | 0.20 | 0.01 | 0.19 | 0.08 | 0.05 | 0.09 | 0.06 | 500 |
| 4 | 3 | 500 | 100 | 0.5 | 0.5 | 2.5 | 0.5 | 2 | 100.0 | 100.0 | 100.0 | 100.0 | 0.00 | 0.20 | 0.01 | 0.19 | 0.08 | 0.05 | 0.09 | 0.07 | 500 |
| 4 | 3 | 500 | 100 | 2 | 0 | 1.5 | 2 | 1 | 5.8 | 5.8 | 6.0 | 5.8 | 0.00 | 0.00 | 0.00 | 0.00 | 0.09 | 0.04 | 0.09 | 0.04 | 500 |
| 4 | 3 | 500 | 100 | 2 | 0 | 1.5 | 2 | 2 | 4.8 | 4.8 | 4.8 | 4.8 | 0.00 | 0.00 | 0.00 | 0.00 | 0.09 | 0.04 | 0.10 | 0.05 | 500 |
| 4 | 3 | 500 | 100 | 2 | 0 | 2.5 | 0.5 | 1 | 5.0 | 5.0 | 5.0 | 5.0 | 0.01 | 0.00 | 0.01 | 0.00 | 0.09 | 0.04 | 0.09 | 0.04 | 500 |
| 4 | 3 | 500 | 100 | 2 | 0 | 2.5 | 0.5 | 2 | 3.4 | 3.4 | 3.4 | 3.4 | 0.00 | 0.00 | 0.00 | 0.00 | 0.09 | 0.04 | 0.09 | 0.04 | 500 |
| 4 | 3 | 500 | 100 | 2 | 0.2 | 1.5 | 2 | 1 | 57.8 | 57.8 | 58.2 | 58.0 | 0.01 | 0.10 | 0.01 | 0.10 | 0.10 | 0.05 | 0.10 | 0.05 | 500 |
| 4 | 3 | 500 | 100 | 2 | 0.2 | 1.5 | 2 | 2 | 63.0 | 63.0 | 63.2 | 63.2 | 0.01 | 0.10 | 0.01 | 0.10 | 0.09 | 0.04 | 0.09 | 0.05 | 500 |
| 4 | 3 | 500 | 100 | 2 | 0.2 | 2.5 | 0.5 | 1 | 56.4 | 56.4 | 56.6 | 56.2 | 0.00 | 0.11 | 0.00 | 0.10 | 0.09 | 0.04 | 0.09 | 0.05 | 500 |
| 4 | 3 | 500 | 100 | 2 | 0.2 | 2.5 | 0.5 | 2 | 56.2 | 56.2 | 56.4 | 56.2 | 0.00 | 0.11 | 0.00 | 0.11 | 0.09 | 0.04 | 0.09 | 0.05 | 500 |
| 4 | 3 | 500 | 100 | 2 | 0.5 | 1.5 | 2 | 1 | 100.0 | 100.0 | 100.0 | 100.0 | 0.00 | 0.26 | 0.01 | 0.25 | 0.09 | 0.05 | 0.10 | 0.06 | 500 |
| 4 | 3 | 500 | 100 | 2 | 0.5 | 1.5 | 2 | 2 | 100.0 | 100.0 | 100.0 | 100.0 | 0.01 | 0.26 | 0.00 | 0.26 | 0.10 | 0.05 | 0.10 | 0.06 | 500 |
| 4 | 3 | 500 | 100 | 2 | 0.5 | 2.5 | 0.5 | 1 | 100.0 | 100.0 | 100.0 | 100.0 | 0.00 | 0.26 | 0.01 | 0.25 | 0.09 | 0.05 | 0.10 | 0.06 | 500 |
| 4 | 3 | 500 | 100 | 2 | 0.5 | 2.5 | 0.5 | 2 | 100.0 | 100.0 | 100.0 | 100.0 | 0.00 | 0.26 | 0.00 | 0.25 | 0.09 | 0.05 | 0.10 | 0.06 | 500 |
| 4 | 3 | 500 | 250 | 0 | 0 | 1.5 | 2 | 1 | 6.8 | 6.6 | 6.8 | 6.8 | 0.00 | 0.00 | 0.00 | 0.00 | 0.08 | 0.05 | 0.08 | 0.05 | 500 |
| 4 | 3 | 500 | 250 | 0 | 0 | 1.5 | 2 | 2 | 6.2 | 6.2 | 6.2 | 6.2 | 0.00 | 0.00 | 0.00 | 0.00 | 0.08 | 0.05 | 0.08 | 0.05 | 500 |
| 4 | 3 | 500 | 250 | 0 | 0 | 2.5 | 0.5 | 1 | 6.2 | 6.0 | 6.0 | 6.0 | 0.01 | 0.00 | 0.01 | 0.00 | 0.08 | 0.05 | 0.08 | 0.05 | 500 |
| 4 | 3 | 500 | 250 | 0 | 0 | 2.5 | 0.5 | 2 | 5.2 | 5.0 | 5.4 | 5.0 | 0.00 | 0.00 | 0.00 | 0.00 | 0.08 | 0.05 | 0.08 | 0.05 | 500 |
| 4 | 3 | 500 | 250 | 0 | 0.2 | 1.5 | 2 | 1 | 68.0 | 68.0 | 68.0 | 68.0 | 0.00 | 0.08 | 0.00 | 0.08 | 0.08 | 0.05 | 0.08 | 0.05 | 500 |
| 4 | 3 | 500 | 250 | 0 | 0.2 | 1.5 | 2 | 2 | 69.4 | 69.4 | 69.6 | 69.4 | 0.00 | 0.08 | 0.00 | 0.08 | 0.08 | 0.05 | 0.08 | 0.05 | 500 |
| 4 | 3 | 500 | 250 | 0 | 0.2 | 2.5 | 0.5 | 1 | 70.2 | 70.0 | 70.4 | 70.2 | 0.00 | 0.08 | 0.00 | 0.08 | 0.08 | 0.05 | 0.08 | 0.05 | 500 |
| 4 | 3 | 500 | 250 | 0 | 0.2 | 2.5 | 0.5 | 2 | 68.8 | 68.6 | 69.2 | 68.8 | 0.00 | 0.08 | 0.00 | 0.08 | 0.08 | 0.05 | 0.08 | 0.05 | 500 |
| 4 | 3 | 500 | 250 | 0 | 0.5 | 1.5 | 2 | 1 | 100.0 | 100.0 | 100.0 | 100.0 | 0.00 | 0.19 | 0.00 | 0.19 | 0.08 | 0.05 | 0.08 | 0.05 | 500 |
| 4 | 3 | 500 | 250 | 0 | 0.5 | 1.5 | 2 | 2 | 100.0 | 100.0 | 100.0 | 100.0 | 0.01 | 0.19 | 0.01 | 0.19 | 0.08 | 0.05 | 0.08 | 0.06 | 500 |
| 4 | 3 | 500 | 250 | 0 | 0.5 | 2.5 | 0.5 | 1 | 100.0 | 100.0 | 100.0 | 100.0 | 0.00 | 0.20 | 0.00 | 0.20 | 0.08 | 0.05 | 0.09 | 0.06 | 500 |
| 4 | 3 | 500 | 250 | 0 | 0.5 | 2.5 | 0.5 | 2 | 100.0 | 100.0 | 100.0 | 100.0 | 0.00 | 0.20 | 0.01 | 0.19 | 0.09 | 0.06 | 0.09 | 0.06 | 500 |
| 4 | 3 | 500 | 250 | 0.5 | 0 | 1.5 | 2 | 1 | 2.6 | 2.6 | 2.6 | 2.6 | 0.00 | 0.00 | 0.00 | 0.00 | 0.08 | 0.05 | 0.08 | 0.05 | 500 |
| 4 | 3 | 500 | 250 | 0.5 | 0 | 1.5 | 2 | 2 | 2.6 | 2.6 | 2.6 | 2.6 | 0.00 | 0.00 | 0.00 | 0.00 | 0.08 | 0.05 | 0.08 | 0.05 | 500 |
| 4 | 3 | 500 | 250 | 0.5 | 0 | 2.5 | 0.5 | 1 | 5.6 | 5.6 | 5.6 | 5.6 | 0.00 | 0.00 | 0.00 | 0.00 | 0.09 | 0.05 | 0.09 | 0.05 | 500 |
| 4 | 3 | 500 | 250 | 0.5 | 0 | 2.5 | 0.5 | 2 | 4.6 | 4.4 | 4.6 | 4.6 | 0.00 | 0.00 | 0.00 | 0.00 | 0.08 | 0.05 | 0.08 | 0.05 | 500 |
| 4 | 3 | 500 | 250 | 0.5 | 0.2 | 1.5 | 2 | 1 | 68.4 | 68.4 | 68.6 | 68.4 | 0.00 | 0.08 | 0.00 | 0.08 | 0.08 | 0.05 | 0.08 | 0.05 | 500 |
| 4 | 3 | 500 | 250 | 0.5 | 0.2 | 1.5 | 2 | 2 | 67.8 | 67.6 | 67.6 | 67.6 | 0.00 | 0.08 | 0.00 | 0.08 | 0.08 | 0.05 | 0.08 | 0.05 | 500 |
| 4 | 3 | 500 | 250 | 0.5 | 0.2 | 2.5 | 0.5 | 1 | 66.8 | 66.8 | 66.8 | 66.8 | 0.00 | 0.09 | 0.00 | 0.08 | 0.08 | 0.05 | 0.08 | 0.05 | 500 |
| 4 | 3 | 500 | 250 | 0.5 | 0.2 | 2.5 | 0.5 | 2 | 68.2 | 68.2 | 68.2 | 68.2 | 0.00 | 0.08 | 0.00 | 0.08 | 0.08 | 0.05 | 0.09 | 0.05 | 500 |
| 4 | 3 | 500 | 250 | 0.5 | 0.5 | 1.5 | 2 | 1 | 100.0 | 100.0 | 100.0 | 100.0 | 0.00 | 0.19 | 0.00 | 0.19 | 0.08 | 0.05 | 0.09 | 0.06 | 500 |
| 4 | 3 | 500 | 250 | 0.5 | 0.5 | 1.5 | 2 | 2 | 100.0 | 100.0 | 100.0 | 100.0 | 0.00 | 0.20 | 0.00 | 0.20 | 0.08 | 0.05 | 0.09 | 0.06 | 500 |
| 4 | 3 | 500 | 250 | 0.5 | 0.5 | 2.5 | 0.5 | 1 | 100.0 | 100.0 | 100.0 | 100.0 | 0.00 | 0.20 | 0.01 | 0.19 | 0.08 | 0.06 | 0.09 | 0.06 | 500 |
| 4 | 3 | 500 | 250 | 0.5 | 0.5 | 2.5 | 0.5 | 2 | 100.0 | 100.0 | 100.0 | 100.0 | 0.00 | 0.20 | 0.00 | 0.20 | 0.09 | 0.06 | 0.09 | 0.06 | 500 |
| 4 | 3 | 500 | 250 | 2 | 0 | 1.5 | 2 | 1 | 4.6 | 4.6 | 4.6 | 4.6 | 0.00 | 0.00 | 0.00 | 0.00 | 0.09 | 0.04 | 0.09 | 0.04 | 500 |
| 4 | 3 | 500 | 250 | 2 | 0 | 1.5 | 2 | 2 | 4.6 | 4.6 | 4.6 | 4.6 | 0.00 | 0.00 | 0.00 | 0.00 | 0.09 | 0.04 | 0.09 | 0.04 | 500 |
| 4 | 3 | 500 | 250 | 2 | 0 | 2.5 | 0.5 | 1 | 5.4 | 5.4 | 5.4 | 5.4 | 0.00 | 0.00 | 0.00 | 0.00 | 0.09 | 0.04 | 0.09 | 0.04 | 500 |
| 4 | 3 | 500 | 250 | 2 | 0 | 2.5 | 0.5 | 2 | 5.4 | 5.4 | 5.4 | 5.4 | 0.00 | 0.00 | 0.00 | 0.00 | 0.09 | 0.04 | 0.09 | 0.04 | 500 |
| 4 | 3 | 500 | 250 | 2 | 0.2 | 1.5 | 2 | 1 | 58.6 | 58.6 | 58.4 | 58.4 | 0.00 | 0.11 | 0.00 | 0.10 | 0.09 | 0.04 | 0.09 | 0.05 | 500 |
| 4 | 3 | 500 | 250 | 2 | 0.2 | 1.5 | 2 | 2 | 58.4 | 58.4 | 58.4 | 58.4 | 0.00 | 0.11 | 0.00 | 0.11 | 0.09 | 0.04 | 0.09 | 0.05 | 500 |
| 4 | 3 | 500 | 250 | 2 | 0.2 | 2.5 | 0.5 | 1 | 58.4 | 58.2 | 58.6 | 58.6 | 0.00 | 0.10 | 0.00 | 0.10 | 0.09 | 0.05 | 0.09 | 0.05 | 500 |
| 4 | 3 | 500 | 250 | 2 | 0.2 | 2.5 | 0.5 | 2 | 58.4 | 58.2 | 58.6 | 58.6 | 0.00 | 0.10 | 0.00 | 0.10 | 0.09 | 0.05 | 0.09 | 0.05 | 500 |
| 4 | 3 | 500 | 250 | 2 | 0.5 | 1.5 | 2 | 1 | 100.0 | 100.0 | 100.0 | 100.0 | 0.01 | 0.26 | 0.01 | 0.26 | 0.09 | 0.05 | 0.09 | 0.05 | 500 |
| 4 | 3 | 500 | 250 | 2 | 0.5 | 1.5 | 2 | 2 | 100.0 | 100.0 | 100.0 | 100.0 | 0.00 | 0.26 | 0.00 | 0.26 | 0.09 | 0.05 | 0.09 | 0.05 | 500 |
| 4 | 3 | 500 | 250 | 2 | 0.5 | 2.5 | 0.5 | 1 | 100.0 | 100.0 | 100.0 | 100.0 | 0.00 | 0.26 | 0.00 | 0.26 | 0.09 | 0.05 | 0.09 | 0.05 | 500 |
| 4 | 3 | 500 | 250 | 2 | 0.5 | 2.5 | 0.5 | 2 | 100.0 | 100.0 | 100.0 | 100.0 | 0.01 | 0.25 | 0.01 | 0.25 | 0.10 | 0.05 | 0.10 | 0.05 | 500 |
| 4 | 3 | 500 | 500 | 0 | 0 | 1.5 | 2 | 1 | 6.4 | 6.4 | 6.4 | 6.4 | 0.00 | 0.00 | 0.00 | 0.00 | 0.09 | 0.05 | 0.09 | 0.05 | 500 |
| 4 | 3 | 500 | 500 | 0 | 0 | 1.5 | 2 | 2 | 4.0 | 4.0 | 4.0 | 4.0 | 0.00 | 0.00 | 0.00 | 0.00 | 0.08 | 0.05 | 0.08 | 0.05 | 500 |
| 4 | 3 | 500 | 500 | 0 | 0 | 2.5 | 0.5 | 1 | 5.6 | 5.6 | 5.6 | 5.6 | 0.01 | 0.00 | 0.01 | 0.00 | 0.08 | 0.05 | 0.08 | 0.05 | 500 |
| 4 | 3 | 500 | 500 | 0 | 0 | 2.5 | 0.5 | 2 | 4.0 | 4.0 | 4.0 | 4.0 | 0.00 | 0.00 | 0.00 | 0.00 | 0.08 | 0.05 | 0.08 | 0.05 | 500 |
| 4 | 3 | 500 | 500 | 0 | 0.2 | 1.5 | 2 | 1 | 68.6 | 68.6 | 68.8 | 68.6 | 0.00 | 0.08 | 0.00 | 0.08 | 0.08 | 0.05 | 0.08 | 0.05 | 500 |
| 4 | 3 | 500 | 500 | 0 | 0.2 | 1.5 | 2 | 2 | 69.4 | 69.4 | 69.8 | 69.6 | 0.00 | 0.08 | 0.00 | 0.08 | 0.08 | 0.05 | 0.08 | 0.05 | 500 |
| 4 | 3 | 500 | 500 | 0 | 0.2 | 2.5 | 0.5 | 1 | 66.0 | 66.0 | 66.0 | 66.0 | 0.00 | 0.08 | 0.00 | 0.08 | 0.09 | 0.05 | 0.09 | 0.05 | 500 |
| 4 | 3 | 500 | 500 | 0 | 0.2 | 2.5 | 0.5 | 2 | 65.6 | 65.6 | 65.6 | 65.6 | 0.00 | 0.08 | 0.00 | 0.08 | 0.08 | 0.05 | 0.08 | 0.05 | 500 |
| 4 | 3 | 500 | 500 | 0 | 0.5 | 1.5 | 2 | 1 | 100.0 | 100.0 | 100.0 | 100.0 | 0.00 | 0.19 | 0.00 | 0.19 | 0.08 | 0.06 | 0.08 | 0.06 | 500 |
| 4 | 3 | 500 | 500 | 0 | 0.5 | 1.5 | 2 | 2 | 100.0 | 100.0 | 100.0 | 100.0 | 0.00 | 0.19 | 0.00 | 0.19 | 0.08 | 0.06 | 0.09 | 0.06 | 500 |
| 4 | 3 | 500 | 500 | 0 | 0.5 | 2.5 | 0.5 | 1 | 100.0 | 100.0 | 100.0 | 100.0 | 0.00 | 0.20 | 0.00 | 0.19 | 0.09 | 0.06 | 0.09 | 0.06 | 500 |
| 4 | 3 | 500 | 500 | 0 | 0.5 | 2.5 | 0.5 | 2 | 100.0 | 100.0 | 100.0 | 100.0 | 0.00 | 0.20 | 0.00 | 0.20 | 0.08 | 0.05 | 0.08 | 0.05 | 500 |
| 4 | 3 | 500 | 500 | 0.5 | 0 | 1.5 | 2 | 1 | 3.2 | 3.2 | 3.0 | 3.0 | 0.00 | 0.00 | 0.00 | 0.00 | 0.08 | 0.05 | 0.08 | 0.05 | 500 |
| 4 | 3 | 500 | 500 | 0.5 | 0 | 1.5 | 2 | 2 | 4.8 | 4.8 | 4.8 | 4.8 | 0.00 | 0.00 | 0.00 | 0.00 | 0.08 | 0.05 | 0.08 | 0.05 | 500 |
| 4 | 3 | 500 | 500 | 0.5 | 0 | 2.5 | 0.5 | 1 | 6.0 | 6.0 | 6.0 | 6.0 | 0.00 | 0.00 | 0.00 | 0.00 | 0.09 | 0.05 | 0.09 | 0.05 | 500 |
| 4 | 3 | 500 | 500 | 0.5 | 0 | 2.5 | 0.5 | 2 | 4.6 | 4.6 | 4.6 | 4.6 | 0.00 | 0.00 | 0.00 | 0.00 | 0.08 | 0.05 | 0.08 | 0.05 | 500 |
| 4 | 3 | 500 | 500 | 0.5 | 0.2 | 1.5 | 2 | 1 | 66.8 | 66.8 | 66.8 | 66.8 | 0.00 | 0.08 | 0.00 | 0.08 | 0.08 | 0.05 | 0.08 | 0.05 | 500 |
| 4 | 3 | 500 | 500 | 0.5 | 0.2 | 1.5 | 2 | 2 | 66.8 | 66.8 | 66.8 | 66.6 | 0.00 | 0.08 | 0.00 | 0.08 | 0.08 | 0.05 | 0.08 | 0.05 | 500 |
| 4 | 3 | 500 | 500 | 0.5 | 0.2 | 2.5 | 0.5 | 1 | 66.0 | 66.0 | 66.0 | 66.0 | 0.00 | 0.08 | 0.00 | 0.08 | 0.08 | 0.05 | 0.08 | 0.05 | 500 |
| 4 | 3 | 500 | 500 | 0.5 | 0.2 | 2.5 | 0.5 | 2 | 68.2 | 67.8 | 68.2 | 67.8 | 0.00 | 0.08 | 0.00 | 0.08 | 0.08 | 0.05 | 0.08 | 0.05 | 500 |
| 4 | 3 | 500 | 500 | 0.5 | 0.5 | 1.5 | 2 | 1 | 100.0 | 100.0 | 100.0 | 100.0 | 0.00 | 0.19 | 0.01 | 0.19 | 0.08 | 0.05 | 0.08 | 0.06 | 500 |
| 4 | 3 | 500 | 500 | 0.5 | 0.5 | 1.5 | 2 | 2 | 100.0 | 100.0 | 100.0 | 100.0 | 0.00 | 0.19 | 0.00 | 0.19 | 0.08 | 0.05 | 0.08 | 0.05 | 500 |
| 4 | 3 | 500 | 500 | 0.5 | 0.5 | 2.5 | 0.5 | 1 | 100.0 | 100.0 | 100.0 | 100.0 | 0.00 | 0.20 | 0.00 | 0.20 | 0.09 | 0.06 | 0.09 | 0.06 | 500 |
| 4 | 3 | 500 | 500 | 0.5 | 0.5 | 2.5 | 0.5 | 2 | 100.0 | 100.0 | 100.0 | 100.0 | 0.00 | 0.20 | 0.00 | 0.20 | 0.09 | 0.06 | 0.09 | 0.06 | 500 |
| 4 | 3 | 500 | 500 | 2 | 0 | 1.5 | 2 | 1 | 6.8 | 6.6 | 7.0 | 6.8 | 0.01 | 0.00 | 0.01 | 0.00 | 0.10 | 0.04 | 0.10 | 0.05 | 500 |
| 4 | 3 | 500 | 500 | 2 | 0 | 1.5 | 2 | 2 | 4.2 | 4.2 | 4.2 | 4.2 | 0.01 | 0.00 | 0.01 | 0.00 | 0.09 | 0.04 | 0.09 | 0.04 | 500 |
| 4 | 3 | 500 | 500 | 2 | 0 | 2.5 | 0.5 | 1 | 3.2 | 3.2 | 3.2 | 3.2 | 0.00 | 0.00 | 0.00 | 0.00 | 0.09 | 0.04 | 0.09 | 0.04 | 500 |
| 4 | 3 | 500 | 500 | 2 | 0 | 2.5 | 0.5 | 2 | 6.2 | 6.2 | 6.2 | 6.2 | 0.01 | 0.00 | 0.01 | 0.00 | 0.09 | 0.04 | 0.09 | 0.04 | 500 |
| 4 | 3 | 500 | 500 | 2 | 0.2 | 1.5 | 2 | 1 | 58.6 | 58.4 | 58.6 | 58.4 | 0.01 | 0.10 | 0.01 | 0.10 | 0.10 | 0.05 | 0.09 | 0.04 | 500 |
| 4 | 3 | 500 | 500 | 2 | 0.2 | 1.5 | 2 | 2 | 56.0 | 56.0 | 55.8 | 55.8 | 0.00 | 0.11 | 0.00 | 0.11 | 0.09 | 0.04 | 0.09 | 0.04 | 500 |
| 4 | 3 | 500 | 500 | 2 | 0.2 | 2.5 | 0.5 | 1 | 57.4 | 57.2 | 57.4 | 57.4 | 0.00 | 0.11 | 0.00 | 0.11 | 0.10 | 0.05 | 0.10 | 0.05 | 500 |
| 4 | 3 | 500 | 500 | 2 | 0.2 | 2.5 | 0.5 | 2 | 56.4 | 56.4 | 56.6 | 56.6 | 0.00 | 0.11 | 0.00 | 0.11 | 0.10 | 0.05 | 0.09 | 0.05 | 500 |
| 4 | 3 | 500 | 500 | 2 | 0.5 | 1.5 | 2 | 1 | 100.0 | 100.0 | 100.0 | 100.0 | 0.00 | 0.26 | 0.00 | 0.26 | 0.09 | 0.05 | 0.09 | 0.05 | 500 |
| 4 | 3 | 500 | 500 | 2 | 0.5 | 1.5 | 2 | 2 | 99.8 | 99.8 | 99.8 | 99.8 | 0.01 | 0.26 | 0.01 | 0.26 | 0.10 | 0.05 | 0.10 | 0.05 | 500 |
| 4 | 3 | 500 | 500 | 2 | 0.5 | 2.5 | 0.5 | 1 | 100.0 | 100.0 | 100.0 | 100.0 | 0.01 | 0.25 | 0.01 | 0.25 | 0.09 | 0.05 | 0.09 | 0.05 | 500 |
| 4 | 3 | 500 | 500 | 2 | 0.5 | 2.5 | 0.5 | 2 | 100.0 | 100.0 | 100.0 | 100.0 | 0.00 | 0.26 | 0.00 | 0.26 | 0.10 | 0.05 | 0.10 | 0.05 | 500 |
| 4 | 5 | 50 | 100 | 0 | 0 | 1.5 | 2 | 1 | 4.8 | 4.4 | 5.6 | 4.6 | 0.01 | 0.01 | 0.01 | 0.01 | 0.24 | 0.18 | 0.24 | 0.18 | 481 |
| 4 | 5 | 50 | 100 | 0 | 0 | 1.5 | 2 | 2 | 4.6 | 4.4 | 5.2 | 4.6 | 0.01 | 0.00 | 0.01 | 0.00 | 0.23 | 0.17 | 0.24 | 0.18 | 499 |
| 4 | 5 | 50 | 100 | 0 | 0 | 2.5 | 0.5 | 1 | 6.3 | 6.0 | 7.0 | 5.8 | 0.01 | 0.00 | 0.01 | 0.00 | 0.25 | 0.18 | 0.25 | 0.18 | 494 |
| 4 | 5 | 50 | 100 | 0 | 0 | 2.5 | 0.5 | 2 | 4.4 | 4.4 | 4.6 | 4.4 | 0.00 | 0.00 | 0.00 | 0.00 | 0.24 | 0.18 | 0.24 | 0.18 | 500 |
| 4 | 5 | 50 | 100 | 0 | 0.2 | 1.5 | 2 | 1 | 14.9 | 14.2 | 15.0 | 14.4 | 0.00 | 0.05 | 0.00 | 0.05 | 0.24 | 0.18 | 0.24 | 0.19 | 484 |
| 4 | 5 | 50 | 100 | 0 | 0.2 | 1.5 | 2 | 2 | 16.8 | 16.0 | 16.8 | 16.2 | 0.01 | 0.04 | 0.01 | 0.04 | 0.25 | 0.19 | 0.24 | 0.19 | 500 |
| 4 | 5 | 50 | 100 | 0 | 0.2 | 2.5 | 0.5 | 1 | 13.3 | 12.4 | 13.8 | 12.6 | 0.01 | 0.06 | 0.01 | 0.06 | 0.25 | 0.18 | 0.24 | 0.18 | 490 |
| 4 | 5 | 50 | 100 | 0 | 0.2 | 2.5 | 0.5 | 2 | 16.0 | 15.6 | 16.8 | 15.2 | 0.01 | 0.05 | 0.01 | 0.05 | 0.25 | 0.19 | 0.25 | 0.19 | 500 |
| 4 | 5 | 50 | 100 | 0 | 0.5 | 1.5 | 2 | 1 | 56.1 | 53.6 | 54.8 | 53.4 | 0.01 | 0.11 | 0.02 | 0.10 | 0.25 | 0.20 | 0.25 | 0.20 | 481 |
| 4 | 5 | 50 | 100 | 0 | 0.5 | 1.5 | 2 | 2 | 61.0 | 60.6 | 62.6 | 61.0 | 0.02 | 0.10 | 0.03 | 0.09 | 0.24 | 0.19 | 0.24 | 0.19 | 500 |
| 4 | 5 | 50 | 100 | 0 | 0.5 | 2.5 | 0.5 | 1 | 18.4 | 18.4 | 18.4 | 18.4 | 0.01 | 0.13 | 0.01 | 0.12 | 0.24 | 0.18 | 0.24 | 0.19 | 490 |
| 4 | 5 | 50 | 100 | 0 | 0.5 | 2.5 | 0.5 | 2 | 55.2 | 54.6 | 55.8 | 55.6 | 0.01 | 0.12 | 0.01 | 0.12 | 0.25 | 0.19 | 0.24 | 0.19 | 500 |
| 4 | 5 | 50 | 100 | 0.5 | 0 | 1.5 | 2 | 1 | 6.9 | 6.6 | 7.2 | 6.8 | 0.02 | 0.01 | 0.02 | 0.01 | 0.25 | 0.18 | 0.25 | 0.19 | 481 |
| 4 | 5 | 50 | 100 | 0.5 | 0 | 1.5 | 2 | 2 | 3.8 | 3.6 | 4.4 | 3.4 | 0.01 | 0.00 | 0.01 | 0.01 | 0.23 | 0.18 | 0.23 | 0.17 | 500 |
| 4 | 5 | 50 | 100 | 0.5 | 0 | 2.5 | 0.5 | 1 | 5.4 | 4.8 | 5.6 | 4.8 | 0.00 | 0.00 | 0.00 | 0.00 | 0.25 | 0.18 | 0.25 | 0.18 | 497 |
| 4 | 5 | 50 | 100 | 0.5 | 0 | 2.5 | 0.5 | 2 | 3.6 | 3.4 | 3.6 | 3.4 | 0.01 | 0.01 | 0.01 | 0.01 | 0.23 | 0.17 | 0.23 | 0.17 | 500 |
| 4 | 5 | 50 | 100 | 0.5 | 0.2 | 1.5 | 2 | 1 | 16.3 | 15.4 | 16.4 | 16.2 | 0.02 | 0.04 | 0.02 | 0.03 | 0.25 | 0.19 | 0.24 | 0.19 | 484 |
| 4 | 5 | 50 | 100 | 0.5 | 0.2 | 1.5 | 2 | 2 | 17.8 | 17.8 | 18.2 | 17.8 | 0.01 | 0.04 | 0.01 | 0.04 | 0.24 | 0.18 | 0.24 | 0.18 | 500 |
| 4 | 5 | 50 | 100 | 0.5 | 0.2 | 2.5 | 0.5 | 1 | 14.0 | 13.4 | 14.0 | 13.2 | 0.01 | 0.06 | 0.01 | 0.06 | 0.26 | 0.19 | 0.26 | 0.19 | 492 |
| 4 | 5 | 50 | 100 | 0.5 | 0.2 | 2.5 | 0.5 | 2 | 14.2 | 13.8 | 14.6 | 13.8 | 0.00 | 0.05 | 0.01 | 0.05 | 0.24 | 0.18 | 0.24 | 0.18 | 500 |
| 4 | 5 | 50 | 100 | 0.5 | 0.5 | 1.5 | 2 | 1 | 57.1 | 57.1 | 57.1 | 57.1 | 0.01 | 0.11 | 0.02 | 0.11 | 0.23 | 0.18 | 0.23 | 0.19 | 482 |
| 4 | 5 | 50 | 100 | 0.5 | 0.5 | 1.5 | 2 | 2 | 56.8 | 56.0 | 57.2 | 56.2 | 0.01 | 0.11 | 0.01 | 0.11 | 0.25 | 0.20 | 0.25 | 0.20 | 498 |
| 4 | 5 | 50 | 100 | 0.5 | 0.5 | 2.5 | 0.5 | 1 | 60.0 | 59.0 | 60.0 | 58.8 | 0.03 | 0.11 | 0.03 | 0.11 | 0.25 | 0.19 | 0.25 | 0.19 | 495 |
| 4 | 5 | 50 | 100 | 0.5 | 0.5 | 2.5 | 0.5 | 2 | 58.1 | 57.4 | 59.2 | 57.2 | 0.03 | 0.11 | 0.03 | 0.11 | 0.24 | 0.19 | 0.24 | 0.19 | 499 |
| 4 | 5 | 50 | 100 | 2 | 0 | 1.5 | 2 | 1 | 5.0 | 4.6 | 5.2 | 4.8 | 0.01 | 0.00 | 0.01 | 0.01 | 0.26 | 0.17 | 0.27 | 0.17 | 479 |
| 4 | 5 | 50 | 100 | 2 | 0 | 1.5 | 2 | 2 | 6.2 | 6.4 | 6.8 | 6.4 | 0.01 | 0.00 | 0.01 | 0.00 | 0.27 | 0.17 | 0.28 | 0.18 | 500 |
| 4 | 5 | 50 | 100 | 2 | 0 | 2.5 | 0.5 | 1 | 4.9 | 4.6 | 5.4 | 4.2 | 0.01 | 0.01 | 0.01 | 0.01 | 0.26 | 0.17 | 0.27 | 0.18 | 491 |
| 4 | 5 | 50 | 100 | 2 | 0 | 2.5 | 0.5 | 2 | 5.2 | 4.6 | 5.8 | 4.8 | 0.01 | 0.00 | 0.01 | 0.01 | 0.25 | 0.16 | 0.26 | 0.17 | 500 |
| 4 | 5 | 50 | 100 | 2 | 0.2 | 1.5 | 2 | 1 | 11.9 | 11.4 | 12.0 | 11.4 | 0.00 | 0.08 | 0.01 | 0.07 | 0.25 | 0.16 | 0.27 | 0.17 | 479 |
| 4 | 5 | 50 | 100 | 2 | 0.2 | 1.5 | 2 | 2 | 15.0 | 14.6 | 16.0 | 15.0 | 0.03 | 0.05 | 0.04 | 0.05 | 0.27 | 0.18 | 0.28 | 0.19 | 500 |
| 4 | 5 | 50 | 100 | 2 | 0.2 | 2.5 | 0.5 | 1 | 15.0 | 14.4 | 15.4 | 14.4 | 0.01 | 0.06 | 0.02 | 0.06 | 0.27 | 0.18 | 0.28 | 0.18 | 495 |
| 4 | 5 | 50 | 100 | 2 | 0.2 | 2.5 | 0.5 | 2 | 12.6 | 12.2 | 13.2 | 12.4 | 0.01 | 0.07 | 0.01 | 0.06 | 0.25 | 0.17 | 0.26 | 0.17 | 499 |
| 4 | 5 | 50 | 100 | 2 | 0.5 | 1.5 | 2 | 1 | 50.3 | 47.8 | 49.8 | 48.2 | 0.02 | 0.17 | 0.04 | 0.15 | 0.27 | 0.18 | 0.27 | 0.19 | 483 |
| 4 | 5 | 50 | 100 | 2 | 0.5 | 1.5 | 2 | 2 | 47.8 | 47.4 | 49.0 | 47.4 | 0.00 | 0.18 | 0.02 | 0.17 | 0.26 | 0.18 | 0.28 | 0.19 | 500 |
| 4 | 5 | 50 | 100 | 2 | 0.5 | 2.5 | 0.5 | 1 | 51.6 | 50.8 | 52.4 | 51.0 | 0.03 | 0.15 | 0.05 | 0.13 | 0.27 | 0.19 | 0.28 | 0.20 | 494 |
| 4 | 5 | 50 | 100 | 2 | 0.5 | 2.5 | 0.5 | 2 | 54.2 | 53.2 | 55.4 | 53.2 | 0.03 | 0.15 | 0.05 | 0.13 | 0.27 | 0.19 | 0.28 | 0.20 | 500 |
| 4 | 5 | 50 | 250 | 0 | 0 | 1.5 | 2 | 1 | 6.8 | 6.8 | 7.8 | 6.8 | 0.00 | 0.00 | 0.00 | 0.00 | 0.25 | 0.19 | 0.25 | 0.19 | 500 |
| 4 | 5 | 50 | 250 | 0 | 0 | 1.5 | 2 | 2 | 4.4 | 4.2 | 4.6 | 4.0 | 0.01 | 0.00 | 0.01 | 0.00 | 0.24 | 0.18 | 0.23 | 0.18 | 500 |
| 4 | 5 | 50 | 250 | 0 | 0 | 2.5 | 0.5 | 1 | 4.6 | 4.6 | 4.8 | 4.6 | 0.01 | 0.00 | 0.01 | 0.00 | 0.24 | 0.17 | 0.24 | 0.17 | 500 |
| 4 | 5 | 50 | 250 | 0 | 0 | 2.5 | 0.5 | 2 | 4.2 | 3.8 | 4.2 | 3.8 | 0.00 | 0.00 | 0.00 | 0.00 | 0.24 | 0.18 | 0.24 | 0.17 | 500 |
| 4 | 5 | 50 | 250 | 0 | 0.2 | 1.5 | 2 | 1 | 14.4 | 14.4 | 15.0 | 14.4 | 0.00 | 0.04 | 0.00 | 0.05 | 0.24 | 0.18 | 0.24 | 0.18 | 500 |
| 4 | 5 | 50 | 250 | 0 | 0.2 | 1.5 | 2 | 2 | 14.6 | 14.4 | 14.8 | 14.2 | 0.01 | 0.04 | 0.02 | 0.04 | 0.23 | 0.18 | 0.24 | 0.18 | 500 |
| 4 | 5 | 50 | 250 | 0 | 0.2 | 2.5 | 0.5 | 1 | 16.0 | 15.6 | 16.4 | 15.6 | 0.02 | 0.04 | 0.01 | 0.05 | 0.25 | 0.18 | 0.24 | 0.18 | 500 |
| 4 | 5 | 50 | 250 | 0 | 0.2 | 2.5 | 0.5 | 2 | 16.2 | 16.0 | 17.0 | 15.8 | 0.02 | 0.04 | 0.01 | 0.05 | 0.25 | 0.19 | 0.25 | 0.18 | 500 |
| 4 | 5 | 50 | 250 | 0 | 0.5 | 1.5 | 2 | 1 | 56.8 | 56.4 | 57.2 | 56.4 | 0.01 | 0.11 | 0.00 | 0.12 | 0.24 | 0.19 | 0.23 | 0.19 | 500 |
| 4 | 5 | 50 | 250 | 0 | 0.5 | 1.5 | 2 | 2 | 60.4 | 59.8 | 61.8 | 59.4 | 0.02 | 0.10 | 0.02 | 0.10 | 0.25 | 0.21 | 0.24 | 0.20 | 500 |
| 4 | 5 | 50 | 250 | 0 | 0.5 | 2.5 | 0.5 | 1 | 60.6 | 59.6 | 61.4 | 59.2 | 0.02 | 0.11 | 0.02 | 0.12 | 0.24 | 0.19 | 0.24 | 0.19 | 500 |
| 4 | 5 | 50 | 250 | 0 | 0.5 | 2.5 | 0.5 | 2 | 56.4 | 56.2 | 57.4 | 56.2 | 0.03 | 0.11 | 0.02 | 0.12 | 0.26 | 0.20 | 0.25 | 0.19 | 500 |
| 4 | 5 | 50 | 250 | 0.5 | 0 | 1.5 | 2 | 1 | 5.0 | 5.0 | 5.4 | 5.0 | 0.01 | 0.01 | 0.01 | 0.01 | 0.25 | 0.19 | 0.24 | 0.18 | 500 |
| 4 | 5 | 50 | 250 | 0.5 | 0 | 1.5 | 2 | 2 | 4.8 | 4.4 | 5.0 | 4.6 | 0.01 | 0.01 | 0.02 | 0.01 | 0.24 | 0.18 | 0.24 | 0.18 | 500 |
| 4 | 5 | 50 | 250 | 0.5 | 0 | 2.5 | 0.5 | 1 | 5.2 | 4.8 | 5.6 | 4.8 | 0.00 | 0.00 | 0.00 | 0.00 | 0.24 | 0.17 | 0.23 | 0.17 | 500 |
| 4 | 5 | 50 | 250 | 0.5 | 0 | 2.5 | 0.5 | 2 | 6.8 | 6.8 | 7.2 | 6.8 | 0.00 | 0.00 | 0.00 | 0.00 | 0.25 | 0.18 | 0.25 | 0.18 | 500 |
| 4 | 5 | 50 | 250 | 0.5 | 0.2 | 1.5 | 2 | 1 | 12.6 | 12.4 | 13.0 | 12.2 | 0.01 | 0.05 | 0.01 | 0.06 | 0.24 | 0.18 | 0.23 | 0.18 | 500 |
| 4 | 5 | 50 | 250 | 0.5 | 0.2 | 1.5 | 2 | 2 | 12.6 | 12.4 | 12.8 | 12.0 | 0.00 | 0.05 | 0.01 | 0.05 | 0.23 | 0.18 | 0.23 | 0.17 | 500 |
| 4 | 5 | 50 | 250 | 0.5 | 0.2 | 2.5 | 0.5 | 1 | 11.8 | 11.8 | 12.4 | 11.8 | 0.00 | 0.06 | 0.01 | 0.06 | 0.24 | 0.18 | 0.24 | 0.18 | 500 |
| 4 | 5 | 50 | 250 | 0.5 | 0.2 | 2.5 | 0.5 | 2 | 14.4 | 14.0 | 15.2 | 14.4 | 0.00 | 0.06 | 0.00 | 0.06 | 0.25 | 0.18 | 0.25 | 0.18 | 500 |
| 4 | 5 | 50 | 250 | 0.5 | 0.5 | 1.5 | 2 | 1 | 58.6 | 58.0 | 59.0 | 57.8 | 0.01 | 0.11 | 0.01 | 0.11 | 0.24 | 0.19 | 0.24 | 0.19 | 500 |
| 4 | 5 | 50 | 250 | 0.5 | 0.5 | 1.5 | 2 | 2 | 61.2 | 60.8 | 62.0 | 61.0 | 0.03 | 0.09 | 0.02 | 0.10 | 0.26 | 0.21 | 0.25 | 0.20 | 500 |
| 4 | 5 | 50 | 250 | 0.5 | 0.5 | 2.5 | 0.5 | 1 | 56.8 | 56.2 | 58.4 | 56.6 | 0.02 | 0.12 | 0.01 | 0.13 | 0.26 | 0.20 | 0.26 | 0.20 | 500 |
| 4 | 5 | 50 | 250 | 0.5 | 0.5 | 2.5 | 0.5 | 2 | 53.6 | 52.6 | 54.8 | 53.0 | 0.01 | 0.13 | 0.00 | 0.13 | 0.25 | 0.19 | 0.24 | 0.18 | 500 |
| 4 | 5 | 50 | 250 | 2 | 0 | 1.5 | 2 | 1 | 5.0 | 4.8 | 5.0 | 4.8 | 0.00 | 0.00 | 0.00 | 0.00 | 0.26 | 0.16 | 0.26 | 0.17 | 500 |
| 4 | 5 | 50 | 250 | 2 | 0 | 1.5 | 2 | 2 | 4.4 | 4.4 | 5.0 | 4.2 | 0.00 | 0.00 | 0.00 | 0.00 | 0.26 | 0.16 | 0.26 | 0.16 | 500 |
| 4 | 5 | 50 | 250 | 2 | 0 | 2.5 | 0.5 | 1 | 5.6 | 5.4 | 5.6 | 5.4 | 0.01 | 0.01 | 0.01 | 0.01 | 0.26 | 0.17 | 0.26 | 0.17 | 500 |
| 4 | 5 | 50 | 250 | 2 | 0 | 2.5 | 0.5 | 2 | 4.4 | 4.4 | 4.6 | 4.4 | 0.00 | 0.00 | 0.00 | 0.00 | 0.26 | 0.17 | 0.26 | 0.17 | 500 |
| 4 | 5 | 50 | 250 | 2 | 0.2 | 1.5 | 2 | 1 | 14.6 | 14.2 | 14.8 | 14.2 | 0.01 | 0.07 | 0.01 | 0.07 | 0.28 | 0.18 | 0.28 | 0.18 | 500 |
| 4 | 5 | 50 | 250 | 2 | 0.2 | 1.5 | 2 | 2 | 15.2 | 15.2 | 15.8 | 15.0 | 0.02 | 0.06 | 0.02 | 0.06 | 0.28 | 0.18 | 0.28 | 0.18 | 500 |
| 4 | 5 | 50 | 250 | 2 | 0.2 | 2.5 | 0.5 | 1 | 11.2 | 11.2 | 12.6 | 11.0 | 0.02 | 0.06 | 0.02 | 0.06 | 0.25 | 0.17 | 0.25 | 0.17 | 500 |
| 4 | 5 | 50 | 250 | 2 | 0.2 | 2.5 | 0.5 | 2 | 13.8 | 13.2 | 14.4 | 13.4 | 0.01 | 0.06 | 0.02 | 0.06 | 0.26 | 0.17 | 0.26 | 0.17 | 500 |
| 4 | 5 | 50 | 250 | 2 | 0.5 | 1.5 | 2 | 1 | 50.6 | 50.6 | 51.2 | 50.8 | 0.02 | 0.17 | 0.02 | 0.17 | 0.29 | 0.20 | 0.29 | 0.19 | 500 |
| 4 | 5 | 50 | 250 | 2 | 0.5 | 1.5 | 2 | 2 | 50.0 | 50.0 | 51.0 | 50.2 | 0.01 | 0.18 | 0.01 | 0.17 | 0.26 | 0.18 | 0.26 | 0.18 | 500 |
| 4 | 5 | 50 | 250 | 2 | 0.5 | 2.5 | 0.5 | 1 | 51.4 | 50.2 | 52.2 | 50.4 | 0.03 | 0.15 | 0.03 | 0.15 | 0.25 | 0.18 | 0.26 | 0.18 | 500 |
| 4 | 5 | 50 | 250 | 2 | 0.5 | 2.5 | 0.5 | 2 | 52.0 | 51.4 | 53.0 | 51.8 | 0.01 | 0.16 | 0.01 | 0.16 | 0.25 | 0.18 | 0.25 | 0.18 | 500 |
| 4 | 5 | 50 | 500 | 0 | 0 | 1.5 | 2 | 1 | 5.8 | 5.6 | 6.0 | 5.8 | 0.01 | 0.01 | 0.01 | 0.01 | 0.25 | 0.19 | 0.24 | 0.18 | 500 |
| 4 | 5 | 50 | 500 | 0 | 0 | 1.5 | 2 | 2 | 4.8 | 4.8 | 5.2 | 4.8 | 0.00 | 0.00 | 0.00 | 0.00 | 0.24 | 0.18 | 0.23 | 0.18 | 500 |
| 4 | 5 | 50 | 500 | 0 | 0 | 2.5 | 0.5 | 1 | 5.8 | 5.8 | 6.2 | 5.8 | 0.00 | 0.00 | 0.01 | 0.00 | 0.25 | 0.18 | 0.24 | 0.17 | 500 |
| 4 | 5 | 50 | 500 | 0 | 0 | 2.5 | 0.5 | 2 | 5.4 | 5.4 | 5.6 | 5.4 | 0.00 | 0.00 | 0.00 | 0.00 | 0.25 | 0.18 | 0.24 | 0.18 | 500 |
| 4 | 5 | 50 | 500 | 0 | 0.2 | 1.5 | 2 | 1 | 15.2 | 15.2 | 15.6 | 15.0 | 0.01 | 0.04 | 0.01 | 0.04 | 0.23 | 0.18 | 0.23 | 0.18 | 500 |
| 4 | 5 | 50 | 500 | 0 | 0.2 | 1.5 | 2 | 2 | 12.6 | 12.6 | 13.4 | 12.8 | 0.01 | 0.06 | 0.02 | 0.06 | 0.25 | 0.19 | 0.24 | 0.18 | 500 |
| 4 | 5 | 50 | 500 | 0 | 0.2 | 2.5 | 0.5 | 1 | 13.8 | 13.6 | 14.4 | 13.6 | 0.02 | 0.04 | 0.02 | 0.04 | 0.24 | 0.18 | 0.23 | 0.17 | 500 |
| 4 | 5 | 50 | 500 | 0 | 0.2 | 2.5 | 0.5 | 2 | 14.6 | 14.2 | 15.4 | 14.2 | 0.02 | 0.04 | 0.02 | 0.04 | 0.23 | 0.17 | 0.23 | 0.17 | 500 |
| 4 | 5 | 50 | 500 | 0 | 0.5 | 1.5 | 2 | 1 | 59.4 | 59.2 | 60.4 | 59.2 | 0.02 | 0.10 | 0.01 | 0.11 | 0.25 | 0.20 | 0.24 | 0.19 | 500 |
| 4 | 5 | 50 | 500 | 0 | 0.5 | 1.5 | 2 | 2 | 59.8 | 59.4 | 60.8 | 59.0 | 0.02 | 0.11 | 0.01 | 0.11 | 0.24 | 0.19 | 0.23 | 0.18 | 500 |
| 4 | 5 | 50 | 500 | 0 | 0.5 | 2.5 | 0.5 | 1 | 56.8 | 56.2 | 58.2 | 56.4 | 0.01 | 0.12 | 0.00 | 0.13 | 0.24 | 0.19 | 0.23 | 0.18 | 500 |
| 4 | 5 | 50 | 500 | 0 | 0.5 | 2.5 | 0.5 | 2 | 59.6 | 59.2 | 60.6 | 58.8 | 0.02 | 0.12 | 0.01 | 0.13 | 0.24 | 0.18 | 0.23 | 0.17 | 500 |
| 4 | 5 | 50 | 500 | 0.5 | 0 | 1.5 | 2 | 1 | 6.0 | 5.6 | 6.4 | 5.8 | 0.00 | 0.00 | 0.00 | 0.00 | 0.25 | 0.19 | 0.24 | 0.18 | 500 |
| 4 | 5 | 50 | 500 | 0.5 | 0 | 1.5 | 2 | 2 | 4.6 | 4.4 | 5.4 | 4.4 | 0.00 | 0.00 | 0.00 | 0.00 | 0.24 | 0.18 | 0.23 | 0.17 | 500 |
| 4 | 5 | 50 | 500 | 0.5 | 0 | 2.5 | 0.5 | 1 | 6.0 | 5.8 | 6.2 | 5.8 | 0.00 | 0.00 | 0.00 | 0.00 | 0.25 | 0.19 | 0.25 | 0.18 | 500 |
| 4 | 5 | 50 | 500 | 0.5 | 0 | 2.5 | 0.5 | 2 | 7.2 | 7.2 | 7.2 | 7.0 | 0.00 | 0.00 | 0.00 | 0.00 | 0.25 | 0.18 | 0.25 | 0.18 | 500 |
| 4 | 5 | 50 | 500 | 0.5 | 0.2 | 1.5 | 2 | 1 | 12.8 | 12.4 | 13.8 | 12.4 | 0.00 | 0.05 | 0.00 | 0.05 | 0.24 | 0.18 | 0.23 | 0.17 | 500 |
| 4 | 5 | 50 | 500 | 0.5 | 0.2 | 1.5 | 2 | 2 | 12.8 | 12.8 | 13.2 | 12.6 | 0.02 | 0.04 | 0.01 | 0.04 | 0.23 | 0.17 | 0.23 | 0.17 | 500 |
| 4 | 5 | 50 | 500 | 0.5 | 0.2 | 2.5 | 0.5 | 1 | 15.0 | 15.0 | 15.0 | 14.8 | 0.02 | 0.04 | 0.02 | 0.04 | 0.23 | 0.17 | 0.23 | 0.17 | 500 |
| 4 | 5 | 50 | 500 | 0.5 | 0.2 | 2.5 | 0.5 | 2 | 12.8 | 12.4 | 13.4 | 12.2 | 0.01 | 0.05 | 0.01 | 0.05 | 0.23 | 0.17 | 0.23 | 0.16 | 500 |
| 4 | 5 | 50 | 500 | 0.5 | 0.5 | 1.5 | 2 | 1 | 57.0 | 56.4 | 58.0 | 56.2 | 0.01 | 0.13 | 0.02 | 0.13 | 0.25 | 0.20 | 0.24 | 0.19 | 500 |
| 4 | 5 | 50 | 500 | 0.5 | 0.5 | 1.5 | 2 | 2 | 54.8 | 53.8 | 56.4 | 53.6 | 0.00 | 0.12 | 0.00 | 0.12 | 0.25 | 0.20 | 0.24 | 0.19 | 500 |
| 4 | 5 | 50 | 500 | 0.5 | 0.5 | 2.5 | 0.5 | 1 | 58.2 | 57.4 | 58.2 | 57.6 | 0.02 | 0.12 | 0.01 | 0.13 | 0.25 | 0.19 | 0.24 | 0.18 | 500 |
| 4 | 5 | 50 | 500 | 0.5 | 0.5 | 2.5 | 0.5 | 2 | 55.8 | 55.4 | 56.6 | 54.8 | 0.00 | 0.13 | 0.00 | 0.14 | 0.24 | 0.18 | 0.24 | 0.18 | 500 |
| 4 | 5 | 50 | 500 | 2 | 0 | 1.5 | 2 | 1 | 3.8 | 3.8 | 4.0 | 3.8 | 0.01 | 0.01 | 0.01 | 0.01 | 0.25 | 0.15 | 0.25 | 0.16 | 500 |
| 4 | 5 | 50 | 500 | 2 | 0 | 1.5 | 2 | 2 | 4.8 | 4.6 | 4.8 | 4.8 | 0.00 | 0.00 | 0.00 | 0.00 | 0.25 | 0.16 | 0.25 | 0.16 | 500 |
| 4 | 5 | 50 | 500 | 2 | 0 | 2.5 | 0.5 | 1 | 6.2 | 6.0 | 6.2 | 6.0 | 0.01 | 0.00 | 0.01 | 0.01 | 0.26 | 0.17 | 0.27 | 0.17 | 500 |
| 4 | 5 | 50 | 500 | 2 | 0 | 2.5 | 0.5 | 2 | 4.8 | 4.6 | 4.8 | 4.6 | 0.00 | 0.00 | 0.01 | 0.00 | 0.25 | 0.16 | 0.26 | 0.16 | 500 |
| 4 | 5 | 50 | 500 | 2 | 0.2 | 1.5 | 2 | 1 | 14.2 | 14.0 | 15.0 | 14.2 | 0.00 | 0.07 | 0.00 | 0.07 | 0.27 | 0.18 | 0.27 | 0.18 | 500 |
| 4 | 5 | 50 | 500 | 2 | 0.2 | 1.5 | 2 | 2 | 12.8 | 13.0 | 13.2 | 13.0 | 0.01 | 0.07 | 0.01 | 0.07 | 0.26 | 0.16 | 0.26 | 0.16 | 500 |
| 4 | 5 | 50 | 500 | 2 | 0.2 | 2.5 | 0.5 | 1 | 10.4 | 10.2 | 11.2 | 10.2 | 0.00 | 0.07 | 0.00 | 0.07 | 0.26 | 0.17 | 0.25 | 0.17 | 500 |
| 4 | 5 | 50 | 500 | 2 | 0.2 | 2.5 | 0.5 | 2 | 11.4 | 11.2 | 11.8 | 10.8 | 0.00 | 0.07 | 0.00 | 0.07 | 0.25 | 0.16 | 0.25 | 0.16 | 500 |
| 4 | 5 | 50 | 500 | 2 | 0.5 | 1.5 | 2 | 1 | 53.4 | 52.4 | 54.8 | 52.8 | 0.04 | 0.16 | 0.04 | 0.16 | 0.26 | 0.18 | 0.25 | 0.17 | 500 |
| 4 | 5 | 50 | 500 | 2 | 0.5 | 1.5 | 2 | 2 | 49.6 | 49.2 | 51.0 | 49.4 | 0.01 | 0.17 | 0.01 | 0.17 | 0.27 | 0.19 | 0.27 | 0.18 | 500 |
| 4 | 5 | 50 | 500 | 2 | 0.5 | 2.5 | 0.5 | 1 | 47.0 | 45.8 | 47.6 | 45.8 | 0.01 | 0.18 | 0.01 | 0.18 | 0.25 | 0.18 | 0.25 | 0.17 | 500 |
| 4 | 5 | 50 | 500 | 2 | 0.5 | 2.5 | 0.5 | 2 | 50.8 | 50.2 | 52.0 | 49.8 | 0.02 | 0.16 | 0.02 | 0.16 | 0.26 | 0.19 | 0.26 | 0.18 | 500 |
| 4 | 5 | 100 | 100 | 0 | 0 | 1.5 | 2 | 1 | 4.4 | 4.2 | 4.2 | 4.2 | 0.00 | 0.00 | 0.00 | 0.00 | 0.17 | 0.13 | 0.17 | 0.13 | 476 |
| 4 | 5 | 100 | 100 | 0 | 0 | 1.5 | 2 | 2 | 4.6 | 4.6 | 4.8 | 4.8 | 0.00 | 0.00 | 0.00 | 0.00 | 0.16 | 0.12 | 0.17 | 0.13 | 500 |
| 4 | 5 | 100 | 100 | 0 | 0 | 2.5 | 0.5 | 1 | 6.1 | 6.0 | 6.2 | 6.0 | 0.01 | 0.00 | 0.01 | 0.01 | 0.17 | 0.12 | 0.17 | 0.12 | 495 |
| 4 | 5 | 100 | 100 | 0 | 0 | 2.5 | 0.5 | 2 | 4.2 | 4.2 | 4.4 | 4.0 | 0.00 | 0.00 | 0.00 | 0.00 | 0.17 | 0.12 | 0.17 | 0.13 | 500 |
| 4 | 5 | 100 | 100 | 0 | 0.2 | 1.5 | 2 | 1 | 26.0 | 26.0 | 26.0 | 26.0 | 0.00 | 0.05 | 0.00 | 0.05 | 0.17 | 0.13 | 0.17 | 0.13 | 474 |
| 4 | 5 | 100 | 100 | 0 | 0.2 | 1.5 | 2 | 2 | 23.8 | 23.4 | 24.2 | 23.4 | 0.01 | 0.04 | 0.01 | 0.04 | 0.16 | 0.12 | 0.16 | 0.13 | 500 |
| 4 | 5 | 100 | 100 | 0 | 0.2 | 2.5 | 0.5 | 1 | 19.8 | 19.4 | 19.4 | 19.2 | 0.00 | 0.06 | 0.00 | 0.05 | 0.17 | 0.13 | 0.17 | 0.13 | 495 |
| 4 | 5 | 100 | 100 | 0 | 0.2 | 2.5 | 0.5 | 2 | 22.8 | 22.6 | 23.0 | 22.4 | 0.00 | 0.06 | 0.00 | 0.05 | 0.17 | 0.13 | 0.17 | 0.13 | 500 |
| 4 | 5 | 100 | 100 | 0 | 0.5 | 1.5 | 2 | 1 | 88.8 | 88.8 | 88.8 | 88.8 | 0.02 | 0.10 | 0.03 | 0.09 | 0.17 | 0.14 | 0.18 | 0.14 | 483 |
| 4 | 5 | 100 | 100 | 0 | 0.5 | 1.5 | 2 | 2 | 83.0 | 83.0 | 83.6 | 83.0 | 0.01 | 0.13 | 0.01 | 0.12 | 0.16 | 0.13 | 0.17 | 0.13 | 500 |
| 4 | 5 | 100 | 100 | 0 | 0.5 | 2.5 | 0.5 | 1 | 86.1 | 85.4 | 85.6 | 85.4 | 0.01 | 0.13 | 0.02 | 0.12 | 0.17 | 0.13 | 0.18 | 0.14 | 496 |
| 4 | 5 | 100 | 100 | 0 | 0.5 | 2.5 | 0.5 | 2 | 85.2 | 85.2 | 85.4 | 85.2 | 0.01 | 0.12 | 0.02 | 0.11 | 0.18 | 0.14 | 0.18 | 0.14 | 500 |
| 4 | 5 | 100 | 100 | 0.5 | 0 | 1.5 | 2 | 1 | 6.4 | 6.2 | 6.8 | 6.0 | 0.00 | 0.00 | 0.00 | 0.00 | 0.18 | 0.13 | 0.18 | 0.13 | 481 |
| 4 | 5 | 100 | 100 | 0.5 | 0 | 1.5 | 2 | 2 | 3.8 | 3.8 | 4.2 | 3.8 | 0.00 | 0.00 | 0.00 | 0.00 | 0.17 | 0.12 | 0.17 | 0.13 | 500 |
| 4 | 5 | 100 | 100 | 0.5 | 0 | 2.5 | 0.5 | 1 | 5.7 | 5.4 | 6.2 | 5.8 | 0.00 | 0.00 | 0.00 | 0.00 | 0.17 | 0.13 | 0.18 | 0.13 | 495 |
| 4 | 5 | 100 | 100 | 0.5 | 0 | 2.5 | 0.5 | 2 | 5.8 | 5.8 | 5.8 | 5.8 | 0.00 | 0.00 | 0.00 | 0.00 | 0.17 | 0.12 | 0.17 | 0.12 | 498 |
| 4 | 5 | 100 | 100 | 0.5 | 0.2 | 1.5 | 2 | 1 | 22.4 | 21.8 | 22.4 | 21.6 | 0.01 | 0.04 | 0.01 | 0.04 | 0.17 | 0.13 | 0.17 | 0.13 | 486 |
| 4 | 5 | 100 | 100 | 0.5 | 0.2 | 1.5 | 2 | 2 | 20.9 | 20.8 | 21.2 | 20.6 | 0.00 | 0.05 | 0.00 | 0.05 | 0.16 | 0.12 | 0.16 | 0.12 | 498 |
| 4 | 5 | 100 | 100 | 0.5 | 0.2 | 2.5 | 0.5 | 1 | 22.7 | 22.2 | 22.4 | 22.2 | 0.00 | 0.06 | 0.00 | 0.05 | 0.18 | 0.13 | 0.18 | 0.14 | 494 |
| 4 | 5 | 100 | 100 | 0.5 | 0.2 | 2.5 | 0.5 | 2 | 22.6 | 22.6 | 23.0 | 21.8 | 0.00 | 0.05 | 0.01 | 0.05 | 0.17 | 0.12 | 0.17 | 0.13 | 500 |
| 4 | 5 | 100 | 100 | 0.5 | 0.5 | 1.5 | 2 | 1 | 87.6 | 87.6 | 87.6 | 87.6 | 0.02 | 0.11 | 0.03 | 0.09 | 0.17 | 0.13 | 0.17 | 0.14 | 490 |
| 4 | 5 | 100 | 100 | 0.5 | 0.5 | 1.5 | 2 | 2 | 86.0 | 86.0 | 86.2 | 85.8 | 0.01 | 0.11 | 0.02 | 0.11 | 0.18 | 0.14 | 0.18 | 0.15 | 500 |
| 4 | 5 | 100 | 100 | 0.5 | 0.5 | 2.5 | 0.5 | 1 | 84.7 | 83.6 | 83.8 | 82.8 | 0.01 | 0.12 | 0.02 | 0.12 | 0.17 | 0.13 | 0.18 | 0.14 | 495 |
| 4 | 5 | 100 | 100 | 0.5 | 0.5 | 2.5 | 0.5 | 2 | 83.4 | 83.2 | 83.4 | 83.0 | 0.00 | 0.13 | 0.01 | 0.12 | 0.17 | 0.13 | 0.17 | 0.14 | 500 |
| 4 | 5 | 100 | 100 | 2 | 0 | 1.5 | 2 | 1 | 5.2 | 4.8 | 4.8 | 4.8 | 0.01 | 0.01 | 0.01 | 0.01 | 0.19 | 0.12 | 0.19 | 0.12 | 483 |
| 4 | 5 | 100 | 100 | 2 | 0 | 1.5 | 2 | 2 | 5.2 | 5.2 | 5.8 | 5.2 | 0.00 | 0.00 | 0.00 | 0.00 | 0.19 | 0.12 | 0.19 | 0.12 | 500 |
| 4 | 5 | 100 | 100 | 2 | 0 | 2.5 | 0.5 | 1 | 3.8 | 3.8 | 4.0 | 4.0 | 0.01 | 0.00 | 0.01 | 0.00 | 0.18 | 0.12 | 0.19 | 0.12 | 495 |
| 4 | 5 | 100 | 100 | 2 | 0 | 2.5 | 0.5 | 2 | 7.0 | 7.0 | 7.0 | 7.0 | 0.01 | 0.01 | 0.01 | 0.01 | 0.18 | 0.12 | 0.19 | 0.12 | 500 |
| 4 | 5 | 100 | 100 | 2 | 0.2 | 1.5 | 2 | 1 | 18.4 | 18.4 | 18.4 | 18.4 | 0.01 | 0.08 | 0.00 | 0.07 | 0.19 | 0.12 | 0.19 | 0.12 | 474 |
| 4 | 5 | 100 | 100 | 2 | 0.2 | 1.5 | 2 | 2 | 19.4 | 19.2 | 19.0 | 19.0 | 0.00 | 0.07 | 0.00 | 0.07 | 0.19 | 0.12 | 0.19 | 0.12 | 500 |
| 4 | 5 | 100 | 100 | 2 | 0.2 | 2.5 | 0.5 | 1 | 16.0 | 15.6 | 16.2 | 15.4 | 0.00 | 0.07 | 0.01 | 0.06 | 0.16 | 0.11 | 0.17 | 0.12 | 495 |
| 4 | 5 | 100 | 100 | 2 | 0.2 | 2.5 | 0.5 | 2 | 20.6 | 20.6 | 21.2 | 20.8 | 0.00 | 0.07 | 0.01 | 0.06 | 0.18 | 0.12 | 0.18 | 0.12 | 500 |
| 4 | 5 | 100 | 100 | 2 | 0.5 | 1.5 | 2 | 1 | 80.5 | 80.5 | 80.5 | 80.5 | 0.01 | 0.17 | 0.03 | 0.16 | 0.18 | 0.12 | 0.19 | 0.13 | 477 |
| 4 | 5 | 100 | 100 | 2 | 0.5 | 1.5 | 2 | 2 | 81.6 | 81.6 | 81.8 | 81.6 | 0.02 | 0.17 | 0.04 | 0.16 | 0.18 | 0.12 | 0.19 | 0.14 | 499 |
| 4 | 5 | 100 | 100 | 2 | 0.5 | 2.5 | 0.5 | 1 | 79.0 | 77.4 | 78.8 | 77.6 | 0.00 | 0.18 | 0.02 | 0.16 | 0.18 | 0.13 | 0.20 | 0.14 | 495 |
| 4 | 5 | 100 | 100 | 2 | 0.5 | 2.5 | 0.5 | 2 | 80.8 | 80.4 | 81.2 | 80.4 | 0.01 | 0.17 | 0.03 | 0.15 | 0.17 | 0.12 | 0.19 | 0.14 | 500 |
| 4 | 5 | 100 | 250 | 0 | 0 | 1.5 | 2 | 1 | 5.2 | 5.0 | 5.4 | 5.0 | 0.01 | 0.00 | 0.01 | 0.00 | 0.17 | 0.13 | 0.17 | 0.13 | 500 |
| 4 | 5 | 100 | 250 | 0 | 0 | 1.5 | 2 | 2 | 6.2 | 6.2 | 6.2 | 6.0 | 0.01 | 0.01 | 0.01 | 0.00 | 0.16 | 0.12 | 0.16 | 0.12 | 500 |
| 4 | 5 | 100 | 250 | 0 | 0 | 2.5 | 0.5 | 1 | 5.8 | 5.6 | 6.6 | 5.6 | 0.01 | 0.00 | 0.01 | 0.01 | 0.18 | 0.13 | 0.18 | 0.13 | 500 |
| 4 | 5 | 100 | 250 | 0 | 0 | 2.5 | 0.5 | 2 | 4.6 | 4.4 | 4.6 | 4.4 | 0.01 | 0.01 | 0.01 | 0.01 | 0.16 | 0.12 | 0.16 | 0.12 | 500 |
| 4 | 5 | 100 | 250 | 0 | 0.2 | 1.5 | 2 | 1 | 21.6 | 21.4 | 21.6 | 21.6 | 0.00 | 0.05 | 0.00 | 0.05 | 0.17 | 0.13 | 0.17 | 0.13 | 500 |
| 4 | 5 | 100 | 250 | 0 | 0.2 | 1.5 | 2 | 2 | 24.0 | 23.8 | 24.6 | 24.0 | 0.00 | 0.05 | 0.00 | 0.05 | 0.17 | 0.13 | 0.17 | 0.13 | 500 |
| 4 | 5 | 100 | 250 | 0 | 0.2 | 2.5 | 0.5 | 1 | 23.6 | 23.2 | 24.2 | 23.4 | 0.01 | 0.05 | 0.01 | 0.05 | 0.17 | 0.12 | 0.17 | 0.12 | 500 |
| 4 | 5 | 100 | 250 | 0 | 0.2 | 2.5 | 0.5 | 2 | 19.2 | 19.0 | 19.8 | 19.4 | 0.01 | 0.06 | 0.01 | 0.06 | 0.17 | 0.12 | 0.17 | 0.12 | 500 |
| 4 | 5 | 100 | 250 | 0 | 0.5 | 1.5 | 2 | 1 | 85.4 | 85.4 | 85.6 | 85.6 | 0.02 | 0.10 | 0.02 | 0.10 | 0.18 | 0.14 | 0.17 | 0.14 | 500 |
| 4 | 5 | 100 | 250 | 0 | 0.5 | 1.5 | 2 | 2 | 85.8 | 85.8 | 86.0 | 85.4 | 0.00 | 0.11 | 0.00 | 0.12 | 0.17 | 0.13 | 0.17 | 0.13 | 500 |
| 4 | 5 | 100 | 250 | 0 | 0.5 | 2.5 | 0.5 | 1 | 83.4 | 83.2 | 83.6 | 83.2 | 0.00 | 0.13 | 0.00 | 0.14 | 0.17 | 0.13 | 0.17 | 0.13 | 500 |
| 4 | 5 | 100 | 250 | 0 | 0.5 | 2.5 | 0.5 | 2 | 84.2 | 83.8 | 84.0 | 83.8 | 0.00 | 0.13 | 0.00 | 0.13 | 0.18 | 0.14 | 0.18 | 0.14 | 500 |
| 4 | 5 | 100 | 250 | 0.5 | 0 | 1.5 | 2 | 1 | 6.0 | 6.0 | 6.4 | 6.0 | 0.01 | 0.01 | 0.01 | 0.01 | 0.17 | 0.12 | 0.17 | 0.13 | 500 |
| 4 | 5 | 100 | 250 | 0.5 | 0 | 1.5 | 2 | 2 | 6.6 | 6.4 | 7.0 | 6.4 | 0.00 | 0.00 | 0.00 | 0.00 | 0.18 | 0.13 | 0.18 | 0.13 | 500 |
| 4 | 5 | 100 | 250 | 0.5 | 0 | 2.5 | 0.5 | 1 | 4.6 | 4.4 | 4.6 | 4.4 | 0.00 | 0.00 | 0.01 | 0.00 | 0.17 | 0.12 | 0.17 | 0.12 | 500 |
| 4 | 5 | 100 | 250 | 0.5 | 0 | 2.5 | 0.5 | 2 | 5.0 | 5.0 | 5.0 | 5.0 | 0.00 | 0.00 | 0.00 | 0.00 | 0.17 | 0.13 | 0.17 | 0.13 | 500 |
| 4 | 5 | 100 | 250 | 0.5 | 0.2 | 1.5 | 2 | 1 | 25.0 | 24.8 | 25.2 | 24.6 | 0.01 | 0.05 | 0.00 | 0.05 | 0.18 | 0.13 | 0.17 | 0.13 | 500 |
| 4 | 5 | 100 | 250 | 0.5 | 0.2 | 1.5 | 2 | 2 | 26.0 | 26.0 | 26.6 | 26.0 | 0.00 | 0.05 | 0.01 | 0.05 | 0.17 | 0.13 | 0.18 | 0.13 | 500 |
| 4 | 5 | 100 | 250 | 0.5 | 0.2 | 2.5 | 0.5 | 1 | 23.4 | 23.2 | 24.0 | 23.2 | 0.00 | 0.06 | 0.00 | 0.05 | 0.17 | 0.12 | 0.17 | 0.13 | 500 |
| 4 | 5 | 100 | 250 | 0.5 | 0.2 | 2.5 | 0.5 | 2 | 24.6 | 24.6 | 24.8 | 24.4 | 0.01 | 0.05 | 0.01 | 0.05 | 0.17 | 0.12 | 0.17 | 0.12 | 500 |
| 4 | 5 | 100 | 250 | 0.5 | 0.5 | 1.5 | 2 | 1 | 85.6 | 85.2 | 85.8 | 85.2 | 0.00 | 0.12 | 0.00 | 0.12 | 0.17 | 0.13 | 0.17 | 0.14 | 500 |
| 4 | 5 | 100 | 250 | 0.5 | 0.5 | 1.5 | 2 | 2 | 87.4 | 87.4 | 87.8 | 87.2 | 0.01 | 0.11 | 0.01 | 0.12 | 0.17 | 0.13 | 0.17 | 0.13 | 500 |
| 4 | 5 | 100 | 250 | 0.5 | 0.5 | 2.5 | 0.5 | 1 | 84.6 | 84.6 | 84.8 | 84.6 | 0.02 | 0.12 | 0.01 | 0.12 | 0.18 | 0.14 | 0.18 | 0.14 | 500 |
| 4 | 5 | 100 | 250 | 0.5 | 0.5 | 2.5 | 0.5 | 2 | 85.6 | 85.2 | 85.8 | 85.6 | 0.01 | 0.13 | 0.01 | 0.13 | 0.17 | 0.13 | 0.17 | 0.13 | 500 |
| 4 | 5 | 100 | 250 | 2 | 0 | 1.5 | 2 | 1 | 3.6 | 3.4 | 3.8 | 3.6 | 0.00 | 0.00 | 0.01 | 0.00 | 0.18 | 0.11 | 0.18 | 0.11 | 500 |
| 4 | 5 | 100 | 250 | 2 | 0 | 1.5 | 2 | 2 | 6.6 | 6.6 | 6.6 | 6.6 | 0.01 | 0.00 | 0.01 | 0.01 | 0.19 | 0.12 | 0.19 | 0.12 | 500 |
| 4 | 5 | 100 | 250 | 2 | 0 | 2.5 | 0.5 | 1 | 4.4 | 4.2 | 4.8 | 4.0 | 0.01 | 0.00 | 0.01 | 0.00 | 0.17 | 0.11 | 0.18 | 0.11 | 500 |
| 4 | 5 | 100 | 250 | 2 | 0 | 2.5 | 0.5 | 2 | 6.6 | 6.6 | 6.8 | 6.6 | 0.01 | 0.01 | 0.01 | 0.01 | 0.19 | 0.12 | 0.19 | 0.13 | 500 |
| 4 | 5 | 100 | 250 | 2 | 0.2 | 1.5 | 2 | 1 | 18.6 | 18.6 | 19.0 | 18.4 | 0.01 | 0.07 | 0.01 | 0.07 | 0.18 | 0.11 | 0.18 | 0.11 | 500 |
| 4 | 5 | 100 | 250 | 2 | 0.2 | 1.5 | 2 | 2 | 19.0 | 18.8 | 19.2 | 19.0 | 0.00 | 0.08 | 0.00 | 0.07 | 0.18 | 0.11 | 0.18 | 0.12 | 500 |
| 4 | 5 | 100 | 250 | 2 | 0.2 | 2.5 | 0.5 | 1 | 22.2 | 21.4 | 22.8 | 21.6 | 0.02 | 0.06 | 0.02 | 0.06 | 0.18 | 0.12 | 0.19 | 0.12 | 500 |
| 4 | 5 | 100 | 250 | 2 | 0.2 | 2.5 | 0.5 | 2 | 17.8 | 17.4 | 18.0 | 17.4 | 0.01 | 0.08 | 0.01 | 0.07 | 0.18 | 0.11 | 0.18 | 0.12 | 500 |
| 4 | 5 | 100 | 250 | 2 | 0.5 | 1.5 | 2 | 1 | 80.6 | 80.6 | 80.8 | 80.8 | 0.01 | 0.18 | 0.02 | 0.17 | 0.19 | 0.13 | 0.20 | 0.14 | 500 |
| 4 | 5 | 100 | 250 | 2 | 0.5 | 1.5 | 2 | 2 | 76.6 | 76.6 | 76.8 | 76.6 | 0.00 | 0.18 | 0.01 | 0.18 | 0.19 | 0.13 | 0.19 | 0.13 | 500 |
| 4 | 5 | 100 | 250 | 2 | 0.5 | 2.5 | 0.5 | 1 | 79.2 | 79.2 | 79.4 | 78.8 | 0.00 | 0.17 | 0.01 | 0.16 | 0.19 | 0.13 | 0.19 | 0.13 | 500 |
| 4 | 5 | 100 | 250 | 2 | 0.5 | 2.5 | 0.5 | 2 | 79.8 | 79.8 | 80.0 | 79.6 | 0.00 | 0.17 | 0.01 | 0.17 | 0.18 | 0.13 | 0.19 | 0.13 | 500 |
| 4 | 5 | 100 | 500 | 0 | 0 | 1.5 | 2 | 1 | 5.6 | 5.2 | 5.8 | 5.2 | 0.01 | 0.00 | 0.01 | 0.00 | 0.16 | 0.12 | 0.16 | 0.12 | 500 |
| 4 | 5 | 100 | 500 | 0 | 0 | 1.5 | 2 | 2 | 5.2 | 5.2 | 5.2 | 5.2 | 0.00 | 0.00 | 0.00 | 0.00 | 0.16 | 0.12 | 0.16 | 0.12 | 500 |
| 4 | 5 | 100 | 500 | 0 | 0 | 2.5 | 0.5 | 1 | 6.8 | 6.8 | 7.4 | 6.8 | 0.01 | 0.01 | 0.01 | 0.00 | 0.18 | 0.13 | 0.17 | 0.13 | 500 |
| 4 | 5 | 100 | 500 | 0 | 0 | 2.5 | 0.5 | 2 | 3.8 | 3.4 | 3.8 | 3.8 | 0.00 | 0.00 | 0.00 | 0.00 | 0.16 | 0.11 | 0.16 | 0.11 | 500 |
| 4 | 5 | 100 | 500 | 0 | 0.2 | 1.5 | 2 | 1 | 20.0 | 20.0 | 20.2 | 20.2 | 0.01 | 0.05 | 0.01 | 0.05 | 0.17 | 0.13 | 0.17 | 0.13 | 500 |
| 4 | 5 | 100 | 500 | 0 | 0.2 | 1.5 | 2 | 2 | 24.8 | 24.6 | 25.4 | 24.8 | 0.02 | 0.04 | 0.01 | 0.04 | 0.17 | 0.13 | 0.16 | 0.13 | 500 |
| 4 | 5 | 100 | 500 | 0 | 0.2 | 2.5 | 0.5 | 1 | 20.0 | 19.6 | 20.8 | 20.0 | 0.01 | 0.06 | 0.01 | 0.06 | 0.17 | 0.12 | 0.17 | 0.12 | 500 |
| 4 | 5 | 100 | 500 | 0 | 0.2 | 2.5 | 0.5 | 2 | 20.4 | 19.6 | 20.6 | 20.0 | 0.01 | 0.06 | 0.01 | 0.06 | 0.16 | 0.12 | 0.16 | 0.12 | 500 |
| 4 | 5 | 100 | 500 | 0 | 0.5 | 1.5 | 2 | 1 | 86.4 | 86.4 | 86.4 | 86.2 | 0.01 | 0.11 | 0.01 | 0.11 | 0.17 | 0.14 | 0.17 | 0.14 | 500 |
| 4 | 5 | 100 | 500 | 0 | 0.5 | 1.5 | 2 | 2 | 87.8 | 87.6 | 88.0 | 87.8 | 0.03 | 0.09 | 0.02 | 0.10 | 0.18 | 0.14 | 0.17 | 0.14 | 500 |
| 4 | 5 | 100 | 500 | 0 | 0.5 | 2.5 | 0.5 | 1 | 84.8 | 84.8 | 85.2 | 84.6 | 0.00 | 0.13 | 0.00 | 0.14 | 0.17 | 0.13 | 0.17 | 0.13 | 500 |
| 4 | 5 | 100 | 500 | 0 | 0.5 | 2.5 | 0.5 | 2 | 88.4 | 88.4 | 88.4 | 88.2 | 0.02 | 0.12 | 0.01 | 0.12 | 0.16 | 0.13 | 0.16 | 0.13 | 500 |
| 4 | 5 | 100 | 500 | 0.5 | 0 | 1.5 | 2 | 1 | 4.2 | 4.0 | 4.4 | 4.0 | 0.01 | 0.00 | 0.01 | 0.00 | 0.16 | 0.12 | 0.16 | 0.12 | 500 |
| 4 | 5 | 100 | 500 | 0.5 | 0 | 1.5 | 2 | 2 | 4.8 | 4.8 | 5.0 | 4.8 | 0.01 | 0.01 | 0.01 | 0.01 | 0.16 | 0.12 | 0.16 | 0.12 | 500 |
| 4 | 5 | 100 | 500 | 0.5 | 0 | 2.5 | 0.5 | 1 | 3.2 | 3.2 | 3.2 | 3.2 | 0.01 | 0.00 | 0.01 | 0.00 | 0.16 | 0.12 | 0.16 | 0.12 | 500 |
| 4 | 5 | 100 | 500 | 0.5 | 0 | 2.5 | 0.5 | 2 | 4.2 | 4.2 | 4.8 | 4.4 | 0.02 | 0.01 | 0.02 | 0.01 | 0.17 | 0.12 | 0.17 | 0.12 | 500 |
| 4 | 5 | 100 | 500 | 0.5 | 0.2 | 1.5 | 2 | 1 | 25.0 | 24.8 | 25.0 | 24.8 | 0.01 | 0.04 | 0.01 | 0.04 | 0.17 | 0.13 | 0.17 | 0.13 | 500 |
| 4 | 5 | 100 | 500 | 0.5 | 0.2 | 1.5 | 2 | 2 | 24.4 | 24.4 | 25.0 | 24.4 | 0.01 | 0.04 | 0.01 | 0.05 | 0.17 | 0.13 | 0.17 | 0.13 | 500 |
| 4 | 5 | 100 | 500 | 0.5 | 0.2 | 2.5 | 0.5 | 1 | 22.8 | 22.8 | 23.4 | 22.6 | 0.01 | 0.05 | 0.01 | 0.05 | 0.17 | 0.12 | 0.17 | 0.12 | 500 |
| 4 | 5 | 100 | 500 | 0.5 | 0.2 | 2.5 | 0.5 | 2 | 20.4 | 20.4 | 21.0 | 20.0 | 0.00 | 0.06 | 0.00 | 0.06 | 0.17 | 0.12 | 0.17 | 0.12 | 500 |
| 4 | 5 | 100 | 500 | 0.5 | 0.5 | 1.5 | 2 | 1 | 84.8 | 84.8 | 85.4 | 85.0 | 0.00 | 0.12 | 0.00 | 0.12 | 0.16 | 0.13 | 0.16 | 0.13 | 500 |
| 4 | 5 | 100 | 500 | 0.5 | 0.5 | 1.5 | 2 | 2 | 86.6 | 86.6 | 86.6 | 86.6 | 0.00 | 0.12 | 0.00 | 0.12 | 0.17 | 0.13 | 0.16 | 0.13 | 500 |
| 4 | 5 | 100 | 500 | 0.5 | 0.5 | 2.5 | 0.5 | 1 | 85.6 | 85.0 | 86.8 | 85.2 | 0.00 | 0.14 | 0.01 | 0.14 | 0.17 | 0.13 | 0.16 | 0.12 | 500 |
| 4 | 5 | 100 | 500 | 0.5 | 0.5 | 2.5 | 0.5 | 2 | 88.0 | 87.6 | 87.8 | 87.6 | 0.01 | 0.12 | 0.01 | 0.13 | 0.17 | 0.13 | 0.17 | 0.13 | 500 |
| 4 | 5 | 100 | 500 | 2 | 0 | 1.5 | 2 | 1 | 3.0 | 3.0 | 3.0 | 3.0 | 0.00 | 0.00 | 0.00 | 0.00 | 0.18 | 0.11 | 0.18 | 0.11 | 500 |
| 4 | 5 | 100 | 500 | 2 | 0 | 1.5 | 2 | 2 | 6.0 | 6.0 | 6.0 | 6.0 | 0.01 | 0.01 | 0.01 | 0.01 | 0.19 | 0.12 | 0.19 | 0.12 | 500 |
| 4 | 5 | 100 | 500 | 2 | 0 | 2.5 | 0.5 | 1 | 4.6 | 4.6 | 4.8 | 4.6 | 0.00 | 0.00 | 0.00 | 0.00 | 0.18 | 0.12 | 0.18 | 0.12 | 500 |
| 4 | 5 | 100 | 500 | 2 | 0 | 2.5 | 0.5 | 2 | 5.4 | 5.4 | 5.8 | 5.4 | 0.02 | 0.01 | 0.02 | 0.01 | 0.18 | 0.12 | 0.18 | 0.12 | 500 |
| 4 | 5 | 100 | 500 | 2 | 0.2 | 1.5 | 2 | 1 | 19.0 | 18.8 | 19.0 | 19.0 | 0.01 | 0.08 | 0.01 | 0.08 | 0.19 | 0.12 | 0.19 | 0.12 | 500 |
| 4 | 5 | 100 | 500 | 2 | 0.2 | 1.5 | 2 | 2 | 21.6 | 21.0 | 22.0 | 20.8 | 0.01 | 0.07 | 0.01 | 0.07 | 0.19 | 0.12 | 0.19 | 0.12 | 500 |
| 4 | 5 | 100 | 500 | 2 | 0.2 | 2.5 | 0.5 | 1 | 21.0 | 21.0 | 21.8 | 21.0 | 0.00 | 0.07 | 0.01 | 0.07 | 0.19 | 0.12 | 0.19 | 0.13 | 500 |
| 4 | 5 | 100 | 500 | 2 | 0.2 | 2.5 | 0.5 | 2 | 19.2 | 18.8 | 19.4 | 19.0 | 0.01 | 0.08 | 0.00 | 0.07 | 0.18 | 0.12 | 0.18 | 0.12 | 500 |
| 4 | 5 | 100 | 500 | 2 | 0.5 | 1.5 | 2 | 1 | 75.4 | 75.2 | 75.6 | 75.2 | 0.00 | 0.18 | 0.00 | 0.18 | 0.19 | 0.13 | 0.19 | 0.13 | 500 |
| 4 | 5 | 100 | 500 | 2 | 0.5 | 1.5 | 2 | 2 | 79.6 | 79.6 | 80.2 | 79.6 | 0.01 | 0.17 | 0.01 | 0.17 | 0.19 | 0.13 | 0.18 | 0.12 | 500 |
| 4 | 5 | 100 | 500 | 2 | 0.5 | 2.5 | 0.5 | 1 | 80.8 | 80.6 | 80.8 | 80.4 | 0.01 | 0.18 | 0.00 | 0.17 | 0.17 | 0.12 | 0.17 | 0.12 | 500 |
| 4 | 5 | 100 | 500 | 2 | 0.5 | 2.5 | 0.5 | 2 | 81.6 | 81.0 | 82.0 | 81.2 | 0.01 | 0.17 | 0.02 | 0.16 | 0.18 | 0.13 | 0.18 | 0.13 | 500 |
| 4 | 5 | 200 | 100 | 0 | 0 | 1.5 | 2 | 1 | 4.8 | 4.4 | 4.4 | 4.4 | 0.00 | 0.00 | 0.00 | 0.00 | 0.11 | 0.08 | 0.11 | 0.09 | 481 |
| 4 | 5 | 200 | 100 | 0 | 0 | 1.5 | 2 | 2 | 4.4 | 4.4 | 4.6 | 4.6 | 0.00 | 0.00 | 0.00 | 0.00 | 0.11 | 0.08 | 0.11 | 0.09 | 500 |
| 4 | 5 | 200 | 100 | 0 | 0 | 2.5 | 0.5 | 1 | 5.9 | 5.4 | 5.8 | 5.2 | 0.00 | 0.00 | 0.00 | 0.00 | 0.12 | 0.09 | 0.12 | 0.09 | 488 |
| 4 | 5 | 200 | 100 | 0 | 0 | 2.5 | 0.5 | 2 | 4.6 | 4.6 | 4.6 | 4.4 | 0.00 | 0.00 | 0.00 | 0.00 | 0.12 | 0.08 | 0.12 | 0.09 | 500 |
| 4 | 5 | 200 | 100 | 0 | 0.2 | 1.5 | 2 | 1 | 40.4 | 40.4 | 40.4 | 40.4 | 0.00 | 0.05 | 0.00 | 0.05 | 0.11 | 0.08 | 0.11 | 0.09 | 475 |
| 4 | 5 | 200 | 100 | 0 | 0.2 | 1.5 | 2 | 2 | 38.4 | 38.4 | 39.0 | 38.8 | 0.00 | 0.05 | 0.00 | 0.05 | 0.12 | 0.09 | 0.12 | 0.09 | 500 |
| 4 | 5 | 200 | 100 | 0 | 0.2 | 2.5 | 0.5 | 1 | 34.2 | 33.4 | 34.2 | 33.6 | 0.01 | 0.07 | 0.01 | 0.06 | 0.12 | 0.09 | 0.12 | 0.09 | 491 |
| 4 | 5 | 200 | 100 | 0 | 0.2 | 2.5 | 0.5 | 2 | 38.6 | 38.6 | 39.0 | 38.8 | 0.01 | 0.05 | 0.01 | 0.05 | 0.13 | 0.09 | 0.13 | 0.09 | 500 |
| 4 | 5 | 200 | 100 | 0 | 0.5 | 1.5 | 2 | 1 | 98.3 | 98.3 | 98.3 | 98.3 | 0.01 | 0.11 | 0.02 | 0.10 | 0.12 | 0.10 | 0.14 | 0.11 | 483 |
| 4 | 5 | 200 | 100 | 0 | 0.5 | 1.5 | 2 | 2 | 99.0 | 99.0 | 99.0 | 99.0 | 0.00 | 0.12 | 0.01 | 0.11 | 0.12 | 0.09 | 0.12 | 0.10 | 500 |
| 4 | 5 | 200 | 100 | 0 | 0.5 | 2.5 | 0.5 | 1 | 98.0 | 98.0 | 98.0 | 98.0 | 0.00 | 0.13 | 0.00 | 0.13 | 0.12 | 0.10 | 0.13 | 0.10 | 493 |
| 4 | 5 | 200 | 100 | 0 | 0.5 | 2.5 | 0.5 | 2 | 99.0 | 98.8 | 98.6 | 98.6 | 0.00 | 0.13 | 0.01 | 0.12 | 0.12 | 0.09 | 0.13 | 0.10 | 499 |
| 4 | 5 | 200 | 100 | 0.5 | 0 | 1.5 | 2 | 1 | 4.2 | 4.0 | 4.0 | 4.0 | 0.00 | 0.00 | 0.00 | 0.00 | 0.11 | 0.09 | 0.12 | 0.09 | 474 |
| 4 | 5 | 200 | 100 | 0.5 | 0 | 1.5 | 2 | 2 | 4.6 | 4.6 | 4.8 | 4.8 | 0.00 | 0.00 | 0.00 | 0.00 | 0.11 | 0.08 | 0.12 | 0.09 | 500 |
| 4 | 5 | 200 | 100 | 0.5 | 0 | 2.5 | 0.5 | 1 | 5.9 | 5.8 | 5.8 | 5.8 | 0.00 | 0.00 | 0.00 | 0.00 | 0.11 | 0.08 | 0.12 | 0.08 | 496 |
| 4 | 5 | 200 | 100 | 0.5 | 0 | 2.5 | 0.5 | 2 | 4.6 | 4.4 | 4.8 | 4.6 | 0.02 | 0.01 | 0.02 | 0.01 | 0.12 | 0.09 | 0.12 | 0.09 | 500 |
| 4 | 5 | 200 | 100 | 0.5 | 0.2 | 1.5 | 2 | 1 | 41.9 | 40.4 | 40.6 | 40.4 | 0.00 | 0.05 | 0.01 | 0.05 | 0.12 | 0.09 | 0.13 | 0.10 | 482 |
| 4 | 5 | 200 | 100 | 0.5 | 0.2 | 1.5 | 2 | 2 | 42.8 | 42.6 | 43.2 | 42.6 | 0.01 | 0.05 | 0.01 | 0.04 | 0.12 | 0.09 | 0.12 | 0.10 | 498 |
| 4 | 5 | 200 | 100 | 0.5 | 0.2 | 2.5 | 0.5 | 1 | 40.6 | 39.6 | 40.0 | 39.8 | 0.00 | 0.05 | 0.00 | 0.05 | 0.12 | 0.09 | 0.12 | 0.09 | 495 |
| 4 | 5 | 200 | 100 | 0.5 | 0.2 | 2.5 | 0.5 | 2 | 39.2 | 39.2 | 39.4 | 39.2 | 0.00 | 0.06 | 0.00 | 0.05 | 0.11 | 0.08 | 0.12 | 0.09 | 500 |
| 4 | 5 | 200 | 100 | 0.5 | 0.5 | 1.5 | 2 | 1 | 98.5 | 98.5 | 98.5 | 98.5 | 0.00 | 0.12 | 0.01 | 0.11 | 0.12 | 0.10 | 0.13 | 0.11 | 481 |
| 4 | 5 | 200 | 100 | 0.5 | 0.5 | 1.5 | 2 | 2 | 98.6 | 98.6 | 98.6 | 98.6 | 0.00 | 0.12 | 0.01 | 0.12 | 0.12 | 0.09 | 0.12 | 0.10 | 500 |
| 4 | 5 | 200 | 100 | 0.5 | 0.5 | 2.5 | 0.5 | 1 | 98.6 | 98.6 | 98.6 | 98.6 | 0.01 | 0.13 | 0.02 | 0.12 | 0.12 | 0.09 | 0.13 | 0.10 | 493 |
| 4 | 5 | 200 | 100 | 0.5 | 0.5 | 2.5 | 0.5 | 2 | 98.8 | 98.8 | 98.8 | 98.8 | 0.00 | 0.14 | 0.01 | 0.13 | 0.12 | 0.09 | 0.13 | 0.11 | 500 |
| 4 | 5 | 200 | 100 | 2 | 0 | 1.5 | 2 | 1 | 6.9 | 6.6 | 6.8 | 6.6 | 0.00 | 0.00 | 0.00 | 0.00 | 0.13 | 0.08 | 0.14 | 0.09 | 492 |
| 4 | 5 | 200 | 100 | 2 | 0 | 1.5 | 2 | 2 | 4.4 | 4.0 | 4.2 | 3.8 | 0.01 | 0.00 | 0.01 | 0.00 | 0.12 | 0.07 | 0.13 | 0.08 | 500 |
| 4 | 5 | 200 | 100 | 2 | 0 | 2.5 | 0.5 | 1 | 6.1 | 6.0 | 6.0 | 6.0 | 0.01 | 0.01 | 0.01 | 0.01 | 0.13 | 0.08 | 0.14 | 0.09 | 492 |
| 4 | 5 | 200 | 100 | 2 | 0 | 2.5 | 0.5 | 2 | 6.2 | 6.2 | 6.2 | 6.2 | 0.01 | 0.01 | 0.01 | 0.01 | 0.12 | 0.08 | 0.13 | 0.08 | 500 |
| 4 | 5 | 200 | 100 | 2 | 0.2 | 1.5 | 2 | 1 | 31.6 | 30.6 | 31.2 | 30.8 | 0.01 | 0.08 | 0.00 | 0.07 | 0.13 | 0.08 | 0.13 | 0.09 | 484 |
| 4 | 5 | 200 | 100 | 2 | 0.2 | 1.5 | 2 | 2 | 35.6 | 35.6 | 36.4 | 36.0 | 0.00 | 0.07 | 0.01 | 0.07 | 0.13 | 0.08 | 0.13 | 0.09 | 500 |
| 4 | 5 | 200 | 100 | 2 | 0.2 | 2.5 | 0.5 | 1 | 39.0 | 38.0 | 38.6 | 38.0 | 0.00 | 0.07 | 0.01 | 0.06 | 0.12 | 0.08 | 0.13 | 0.09 | 492 |
| 4 | 5 | 200 | 100 | 2 | 0.2 | 2.5 | 0.5 | 2 | 34.4 | 34.4 | 34.4 | 34.0 | 0.01 | 0.08 | 0.00 | 0.07 | 0.13 | 0.08 | 0.13 | 0.09 | 500 |
| 4 | 5 | 200 | 100 | 2 | 0.5 | 1.5 | 2 | 1 | 97.7 | 97.1 | 97.7 | 97.1 | 0.01 | 0.19 | 0.01 | 0.17 | 0.13 | 0.09 | 0.15 | 0.11 | 476 |
| 4 | 5 | 200 | 100 | 2 | 0.5 | 1.5 | 2 | 2 | 96.4 | 96.0 | 96.2 | 96.0 | 0.00 | 0.18 | 0.02 | 0.16 | 0.14 | 0.09 | 0.15 | 0.11 | 499 |
| 4 | 5 | 200 | 100 | 2 | 0.5 | 2.5 | 0.5 | 1 | 98.4 | 98.4 | 98.4 | 98.4 | 0.00 | 0.17 | 0.02 | 0.15 | 0.12 | 0.09 | 0.14 | 0.10 | 496 |
| 4 | 5 | 200 | 100 | 2 | 0.5 | 2.5 | 0.5 | 2 | 96.8 | 96.6 | 96.8 | 96.8 | 0.00 | 0.17 | 0.02 | 0.16 | 0.13 | 0.09 | 0.15 | 0.11 | 500 |
| 4 | 5 | 200 | 250 | 0 | 0 | 1.5 | 2 | 1 | 4.6 | 4.6 | 4.8 | 4.6 | 0.00 | 0.00 | 0.00 | 0.00 | 0.12 | 0.09 | 0.12 | 0.09 | 500 |
| 4 | 5 | 200 | 250 | 0 | 0 | 1.5 | 2 | 2 | 5.2 | 5.2 | 5.4 | 5.2 | 0.00 | 0.00 | 0.00 | 0.00 | 0.11 | 0.09 | 0.11 | 0.09 | 500 |
| 4 | 5 | 200 | 250 | 0 | 0 | 2.5 | 0.5 | 1 | 5.4 | 5.4 | 5.4 | 5.4 | 0.00 | 0.00 | 0.00 | 0.00 | 0.12 | 0.09 | 0.13 | 0.09 | 500 |
| 4 | 5 | 200 | 250 | 0 | 0 | 2.5 | 0.5 | 2 | 3.0 | 3.0 | 3.0 | 3.0 | 0.00 | 0.00 | 0.00 | 0.00 | 0.11 | 0.08 | 0.11 | 0.08 | 500 |
| 4 | 5 | 200 | 250 | 0 | 0.2 | 1.5 | 2 | 1 | 41.2 | 41.2 | 41.2 | 40.6 | 0.00 | 0.05 | 0.00 | 0.05 | 0.12 | 0.09 | 0.12 | 0.09 | 500 |
| 4 | 5 | 200 | 250 | 0 | 0.2 | 1.5 | 2 | 2 | 39.6 | 39.4 | 40.4 | 39.6 | 0.00 | 0.05 | 0.00 | 0.05 | 0.12 | 0.09 | 0.12 | 0.09 | 500 |
| 4 | 5 | 200 | 250 | 0 | 0.2 | 2.5 | 0.5 | 1 | 39.2 | 38.6 | 39.2 | 39.0 | 0.00 | 0.05 | 0.00 | 0.05 | 0.12 | 0.09 | 0.12 | 0.09 | 500 |
| 4 | 5 | 200 | 250 | 0 | 0.2 | 2.5 | 0.5 | 2 | 41.4 | 41.0 | 41.2 | 40.8 | 0.01 | 0.05 | 0.01 | 0.05 | 0.12 | 0.09 | 0.12 | 0.09 | 500 |
| 4 | 5 | 200 | 250 | 0 | 0.5 | 1.5 | 2 | 1 | 98.2 | 98.2 | 98.2 | 98.2 | 0.01 | 0.12 | 0.01 | 0.12 | 0.12 | 0.10 | 0.12 | 0.10 | 500 |
| 4 | 5 | 200 | 250 | 0 | 0.5 | 1.5 | 2 | 2 | 99.4 | 99.4 | 99.4 | 99.4 | 0.00 | 0.12 | 0.00 | 0.11 | 0.11 | 0.09 | 0.11 | 0.09 | 500 |
| 4 | 5 | 200 | 250 | 0 | 0.5 | 2.5 | 0.5 | 1 | 98.6 | 98.6 | 98.6 | 98.6 | 0.01 | 0.13 | 0.01 | 0.13 | 0.13 | 0.10 | 0.13 | 0.10 | 500 |
| 4 | 5 | 200 | 250 | 0 | 0.5 | 2.5 | 0.5 | 2 | 98.2 | 98.2 | 98.2 | 98.2 | 0.00 | 0.13 | 0.00 | 0.13 | 0.12 | 0.09 | 0.12 | 0.09 | 500 |
| 4 | 5 | 200 | 250 | 0.5 | 0 | 1.5 | 2 | 1 | 4.6 | 4.6 | 4.6 | 4.4 | 0.01 | 0.01 | 0.01 | 0.00 | 0.11 | 0.08 | 0.11 | 0.08 | 500 |
| 4 | 5 | 200 | 250 | 0.5 | 0 | 1.5 | 2 | 2 | 4.2 | 3.8 | 4.0 | 3.8 | 0.00 | 0.00 | 0.00 | 0.00 | 0.11 | 0.08 | 0.11 | 0.08 | 500 |
| 4 | 5 | 200 | 250 | 0.5 | 0 | 2.5 | 0.5 | 1 | 6.8 | 6.8 | 7.0 | 6.8 | 0.00 | 0.00 | 0.00 | 0.00 | 0.13 | 0.09 | 0.13 | 0.09 | 500 |
| 4 | 5 | 200 | 250 | 0.5 | 0 | 2.5 | 0.5 | 2 | 4.6 | 4.4 | 4.4 | 4.2 | 0.01 | 0.01 | 0.01 | 0.01 | 0.12 | 0.09 | 0.12 | 0.09 | 500 |
| 4 | 5 | 200 | 250 | 0.5 | 0.2 | 1.5 | 2 | 1 | 40.8 | 40.8 | 40.6 | 40.4 | 0.00 | 0.05 | 0.00 | 0.05 | 0.12 | 0.09 | 0.12 | 0.09 | 500 |
| 4 | 5 | 200 | 250 | 0.5 | 0.2 | 1.5 | 2 | 2 | 37.4 | 36.8 | 37.2 | 36.6 | 0.01 | 0.06 | 0.01 | 0.06 | 0.12 | 0.09 | 0.12 | 0.09 | 500 |
| 4 | 5 | 200 | 250 | 0.5 | 0.2 | 2.5 | 0.5 | 1 | 38.4 | 38.2 | 38.8 | 37.8 | 0.01 | 0.05 | 0.01 | 0.05 | 0.12 | 0.08 | 0.12 | 0.09 | 500 |
| 4 | 5 | 200 | 250 | 0.5 | 0.2 | 2.5 | 0.5 | 2 | 42.8 | 42.8 | 42.8 | 42.6 | 0.00 | 0.05 | 0.00 | 0.05 | 0.12 | 0.09 | 0.12 | 0.09 | 500 |
| 4 | 5 | 200 | 250 | 0.5 | 0.5 | 1.5 | 2 | 1 | 99.0 | 98.8 | 98.8 | 98.8 | 0.01 | 0.11 | 0.01 | 0.11 | 0.12 | 0.09 | 0.12 | 0.10 | 499 |
| 4 | 5 | 200 | 250 | 0.5 | 0.5 | 1.5 | 2 | 2 | 99.6 | 99.6 | 99.6 | 99.6 | 0.01 | 0.12 | 0.01 | 0.11 | 0.12 | 0.10 | 0.12 | 0.10 | 500 |
| 4 | 5 | 200 | 250 | 0.5 | 0.5 | 2.5 | 0.5 | 1 | 99.4 | 99.4 | 99.4 | 99.4 | 0.00 | 0.13 | 0.01 | 0.13 | 0.12 | 0.09 | 0.12 | 0.09 | 500 |
| 4 | 5 | 200 | 250 | 0.5 | 0.5 | 2.5 | 0.5 | 2 | 98.6 | 98.6 | 98.6 | 98.6 | 0.00 | 0.14 | 0.00 | 0.13 | 0.12 | 0.09 | 0.12 | 0.10 | 500 |
| 4 | 5 | 200 | 250 | 2 | 0 | 1.5 | 2 | 1 | 5.4 | 5.4 | 5.6 | 5.4 | 0.00 | 0.00 | 0.00 | 0.00 | 0.13 | 0.08 | 0.13 | 0.08 | 500 |
| 4 | 5 | 200 | 250 | 2 | 0 | 1.5 | 2 | 2 | 4.0 | 4.0 | 4.0 | 3.8 | 0.00 | 0.00 | 0.00 | 0.00 | 0.13 | 0.08 | 0.13 | 0.08 | 500 |
| 4 | 5 | 200 | 250 | 2 | 0 | 2.5 | 0.5 | 1 | 5.2 | 4.8 | 5.2 | 5.0 | 0.01 | 0.01 | 0.02 | 0.01 | 0.12 | 0.08 | 0.13 | 0.08 | 500 |
| 4 | 5 | 200 | 250 | 2 | 0 | 2.5 | 0.5 | 2 | 5.6 | 5.6 | 5.6 | 5.6 | 0.01 | 0.00 | 0.01 | 0.00 | 0.13 | 0.08 | 0.13 | 0.09 | 500 |
| 4 | 5 | 200 | 250 | 2 | 0.2 | 1.5 | 2 | 1 | 32.2 | 32.4 | 32.6 | 32.4 | 0.00 | 0.08 | 0.00 | 0.07 | 0.13 | 0.08 | 0.13 | 0.08 | 500 |
| 4 | 5 | 200 | 250 | 2 | 0.2 | 1.5 | 2 | 2 | 38.4 | 38.2 | 39.2 | 39.2 | 0.01 | 0.07 | 0.01 | 0.07 | 0.13 | 0.08 | 0.13 | 0.08 | 500 |
| 4 | 5 | 200 | 250 | 2 | 0.2 | 2.5 | 0.5 | 1 | 37.0 | 36.4 | 37.0 | 36.2 | 0.00 | 0.07 | 0.00 | 0.07 | 0.13 | 0.08 | 0.13 | 0.08 | 500 |
| 4 | 5 | 200 | 250 | 2 | 0.2 | 2.5 | 0.5 | 2 | 31.8 | 31.6 | 32.2 | 32.0 | 0.01 | 0.08 | 0.01 | 0.08 | 0.13 | 0.08 | 0.13 | 0.08 | 500 |
| 4 | 5 | 200 | 250 | 2 | 0.5 | 1.5 | 2 | 1 | 97.8 | 97.8 | 98.0 | 98.0 | 0.00 | 0.19 | 0.01 | 0.18 | 0.13 | 0.09 | 0.13 | 0.09 | 500 |
| 4 | 5 | 200 | 250 | 2 | 0.5 | 1.5 | 2 | 2 | 97.2 | 97.2 | 97.4 | 97.2 | 0.01 | 0.18 | 0.01 | 0.17 | 0.13 | 0.09 | 0.13 | 0.09 | 500 |
| 4 | 5 | 200 | 250 | 2 | 0.5 | 2.5 | 0.5 | 1 | 98.4 | 98.4 | 98.4 | 98.4 | 0.02 | 0.16 | 0.02 | 0.15 | 0.13 | 0.09 | 0.13 | 0.10 | 500 |
| 4 | 5 | 200 | 250 | 2 | 0.5 | 2.5 | 0.5 | 2 | 97.4 | 97.4 | 97.4 | 97.4 | 0.00 | 0.17 | 0.01 | 0.17 | 0.13 | 0.09 | 0.14 | 0.10 | 500 |
| 4 | 5 | 200 | 500 | 0 | 0 | 1.5 | 2 | 1 | 6.6 | 6.6 | 6.8 | 6.8 | 0.01 | 0.01 | 0.01 | 0.01 | 0.12 | 0.09 | 0.12 | 0.09 | 500 |
| 4 | 5 | 200 | 500 | 0 | 0 | 1.5 | 2 | 2 | 3.8 | 3.8 | 3.8 | 3.8 | 0.00 | 0.00 | 0.00 | 0.00 | 0.11 | 0.08 | 0.11 | 0.08 | 500 |
| 4 | 5 | 200 | 500 | 0 | 0 | 2.5 | 0.5 | 1 | 4.2 | 4.2 | 4.4 | 4.2 | 0.00 | 0.00 | 0.00 | 0.00 | 0.12 | 0.09 | 0.12 | 0.09 | 500 |
| 4 | 5 | 200 | 500 | 0 | 0 | 2.5 | 0.5 | 2 | 3.8 | 3.8 | 4.4 | 3.8 | 0.00 | 0.00 | 0.00 | 0.00 | 0.12 | 0.09 | 0.12 | 0.09 | 500 |
| 4 | 5 | 200 | 500 | 0 | 0.2 | 1.5 | 2 | 1 | 43.4 | 43.2 | 43.6 | 43.4 | 0.01 | 0.04 | 0.01 | 0.04 | 0.12 | 0.09 | 0.12 | 0.09 | 500 |
| 4 | 5 | 200 | 500 | 0 | 0.2 | 1.5 | 2 | 2 | 36.8 | 36.8 | 37.0 | 36.8 | 0.01 | 0.05 | 0.01 | 0.05 | 0.12 | 0.09 | 0.12 | 0.09 | 500 |
| 4 | 5 | 200 | 500 | 0 | 0.2 | 2.5 | 0.5 | 1 | 40.0 | 39.8 | 40.2 | 39.8 | 0.00 | 0.05 | 0.00 | 0.05 | 0.12 | 0.09 | 0.12 | 0.09 | 500 |
| 4 | 5 | 200 | 500 | 0 | 0.2 | 2.5 | 0.5 | 2 | 41.4 | 41.4 | 41.6 | 41.2 | 0.00 | 0.05 | 0.00 | 0.05 | 0.12 | 0.09 | 0.12 | 0.09 | 500 |
| 4 | 5 | 200 | 500 | 0 | 0.5 | 1.5 | 2 | 1 | 98.6 | 98.6 | 98.6 | 98.6 | 0.00 | 0.12 | 0.01 | 0.12 | 0.12 | 0.10 | 0.12 | 0.10 | 500 |
| 4 | 5 | 200 | 500 | 0 | 0.5 | 1.5 | 2 | 2 | 99.4 | 99.4 | 99.4 | 99.4 | 0.00 | 0.11 | 0.00 | 0.11 | 0.11 | 0.09 | 0.11 | 0.09 | 500 |
| 4 | 5 | 200 | 500 | 0 | 0.5 | 2.5 | 0.5 | 1 | 98.6 | 98.6 | 98.6 | 98.6 | 0.00 | 0.14 | 0.01 | 0.14 | 0.12 | 0.09 | 0.12 | 0.09 | 500 |
| 4 | 5 | 200 | 500 | 0 | 0.5 | 2.5 | 0.5 | 2 | 99.0 | 99.0 | 99.0 | 99.0 | 0.00 | 0.13 | 0.00 | 0.13 | 0.12 | 0.09 | 0.12 | 0.09 | 500 |
| 4 | 5 | 200 | 500 | 0.5 | 0 | 1.5 | 2 | 1 | 5.2 | 5.0 | 5.0 | 4.8 | 0.00 | 0.00 | 0.00 | 0.00 | 0.12 | 0.09 | 0.12 | 0.09 | 500 |
| 4 | 5 | 200 | 500 | 0.5 | 0 | 1.5 | 2 | 2 | 5.0 | 5.0 | 5.0 | 5.0 | 0.00 | 0.00 | 0.00 | 0.00 | 0.12 | 0.09 | 0.12 | 0.09 | 500 |
| 4 | 5 | 200 | 500 | 0.5 | 0 | 2.5 | 0.5 | 1 | 5.2 | 5.0 | 5.4 | 5.0 | 0.00 | 0.00 | 0.00 | 0.00 | 0.12 | 0.09 | 0.12 | 0.09 | 500 |
| 4 | 5 | 200 | 500 | 0.5 | 0 | 2.5 | 0.5 | 2 | 5.8 | 5.8 | 5.8 | 5.8 | 0.00 | 0.00 | 0.00 | 0.00 | 0.12 | 0.09 | 0.12 | 0.09 | 500 |
| 4 | 5 | 200 | 500 | 0.5 | 0.2 | 1.5 | 2 | 1 | 36.0 | 36.0 | 36.8 | 36.0 | 0.01 | 0.06 | 0.01 | 0.06 | 0.11 | 0.09 | 0.12 | 0.09 | 500 |
| 4 | 5 | 200 | 500 | 0.5 | 0.2 | 1.5 | 2 | 2 | 42.4 | 42.0 | 42.6 | 42.0 | 0.01 | 0.04 | 0.01 | 0.04 | 0.12 | 0.09 | 0.12 | 0.09 | 500 |
| 4 | 5 | 200 | 500 | 0.5 | 0.2 | 2.5 | 0.5 | 1 | 39.4 | 39.2 | 39.2 | 39.0 | 0.00 | 0.05 | 0.00 | 0.06 | 0.12 | 0.09 | 0.12 | 0.09 | 500 |
| 4 | 5 | 200 | 500 | 0.5 | 0.2 | 2.5 | 0.5 | 2 | 42.0 | 42.0 | 41.8 | 41.8 | 0.01 | 0.05 | 0.01 | 0.05 | 0.12 | 0.09 | 0.12 | 0.09 | 500 |
| 4 | 5 | 200 | 500 | 0.5 | 0.5 | 1.5 | 2 | 1 | 99.0 | 99.0 | 99.0 | 99.0 | 0.00 | 0.12 | 0.00 | 0.12 | 0.12 | 0.10 | 0.12 | 0.10 | 500 |
| 4 | 5 | 200 | 500 | 0.5 | 0.5 | 1.5 | 2 | 2 | 98.6 | 98.6 | 98.6 | 98.6 | 0.01 | 0.12 | 0.00 | 0.12 | 0.12 | 0.09 | 0.12 | 0.09 | 500 |
| 4 | 5 | 200 | 500 | 0.5 | 0.5 | 2.5 | 0.5 | 1 | 98.6 | 98.6 | 98.6 | 98.6 | 0.01 | 0.13 | 0.01 | 0.13 | 0.13 | 0.10 | 0.13 | 0.10 | 500 |
| 4 | 5 | 200 | 500 | 0.5 | 0.5 | 2.5 | 0.5 | 2 | 98.2 | 98.2 | 98.2 | 98.2 | 0.01 | 0.13 | 0.01 | 0.13 | 0.12 | 0.09 | 0.12 | 0.09 | 500 |
| 4 | 5 | 200 | 500 | 2 | 0 | 1.5 | 2 | 1 | 7.4 | 7.4 | 7.2 | 7.2 | 0.00 | 0.00 | 0.00 | 0.00 | 0.13 | 0.08 | 0.13 | 0.08 | 500 |
| 4 | 5 | 200 | 500 | 2 | 0 | 1.5 | 2 | 2 | 7.4 | 7.4 | 7.4 | 7.0 | 0.00 | 0.00 | 0.00 | 0.00 | 0.13 | 0.08 | 0.13 | 0.08 | 500 |
| 4 | 5 | 200 | 500 | 2 | 0 | 2.5 | 0.5 | 1 | 5.8 | 5.8 | 5.8 | 5.8 | 0.00 | 0.00 | 0.00 | 0.00 | 0.13 | 0.08 | 0.13 | 0.08 | 500 |
| 4 | 5 | 200 | 500 | 2 | 0 | 2.5 | 0.5 | 2 | 5.0 | 5.0 | 4.8 | 4.6 | 0.01 | 0.00 | 0.01 | 0.00 | 0.13 | 0.08 | 0.13 | 0.08 | 500 |
| 4 | 5 | 200 | 500 | 2 | 0.2 | 1.5 | 2 | 1 | 36.8 | 37.0 | 37.0 | 36.6 | 0.01 | 0.07 | 0.01 | 0.07 | 0.13 | 0.08 | 0.13 | 0.08 | 500 |
| 4 | 5 | 200 | 500 | 2 | 0.2 | 1.5 | 2 | 2 | 34.0 | 33.8 | 34.0 | 34.0 | 0.00 | 0.07 | 0.00 | 0.07 | 0.13 | 0.08 | 0.13 | 0.08 | 500 |
| 4 | 5 | 200 | 500 | 2 | 0.2 | 2.5 | 0.5 | 1 | 40.2 | 40.0 | 40.2 | 39.8 | 0.01 | 0.07 | 0.01 | 0.06 | 0.13 | 0.09 | 0.14 | 0.09 | 500 |
| 4 | 5 | 200 | 500 | 2 | 0.2 | 2.5 | 0.5 | 2 | 32.8 | 32.8 | 33.2 | 32.6 | 0.01 | 0.08 | 0.01 | 0.08 | 0.12 | 0.08 | 0.12 | 0.08 | 500 |
| 4 | 5 | 200 | 500 | 2 | 0.5 | 1.5 | 2 | 1 | 95.6 | 95.6 | 95.6 | 95.6 | 0.00 | 0.18 | 0.01 | 0.18 | 0.14 | 0.09 | 0.14 | 0.09 | 500 |
| 4 | 5 | 200 | 500 | 2 | 0.5 | 1.5 | 2 | 2 | 98.2 | 98.2 | 98.2 | 98.0 | 0.00 | 0.18 | 0.00 | 0.18 | 0.13 | 0.09 | 0.13 | 0.09 | 500 |
| 4 | 5 | 200 | 500 | 2 | 0.5 | 2.5 | 0.5 | 1 | 97.8 | 97.6 | 97.8 | 97.8 | 0.01 | 0.18 | 0.00 | 0.18 | 0.12 | 0.09 | 0.12 | 0.09 | 500 |
| 4 | 5 | 200 | 500 | 2 | 0.5 | 2.5 | 0.5 | 2 | 97.4 | 97.4 | 97.6 | 97.6 | 0.00 | 0.18 | 0.00 | 0.17 | 0.13 | 0.09 | 0.13 | 0.09 | 500 |
| 4 | 5 | 500 | 100 | 0 | 0 | 1.5 | 2 | 1 | 6.6 | 6.4 | 6.4 | 6.4 | 0.00 | 0.00 | 0.00 | 0.00 | 0.07 | 0.06 | 0.08 | 0.06 | 487 |
| 4 | 5 | 500 | 100 | 0 | 0 | 1.5 | 2 | 2 | 4.6 | 4.6 | 4.8 | 4.8 | 0.01 | 0.00 | 0.01 | 0.00 | 0.07 | 0.06 | 0.07 | 0.06 | 500 |
| 4 | 5 | 500 | 100 | 0 | 0 | 2.5 | 0.5 | 1 | 5.7 | 5.6 | 5.6 | 5.6 | 0.00 | 0.00 | 0.00 | 0.00 | 0.08 | 0.05 | 0.08 | 0.06 | 496 |
| 4 | 5 | 500 | 100 | 0 | 0 | 2.5 | 0.5 | 2 | 5.0 | 5.0 | 5.0 | 5.0 | 0.00 | 0.00 | 0.00 | 0.00 | 0.07 | 0.05 | 0.08 | 0.06 | 499 |
| 4 | 5 | 500 | 100 | 0 | 0.2 | 1.5 | 2 | 1 | 78.7 | 78.7 | 78.7 | 78.7 | 0.00 | 0.05 | 0.00 | 0.04 | 0.07 | 0.06 | 0.08 | 0.06 | 475 |
| 4 | 5 | 500 | 100 | 0 | 0.2 | 1.5 | 2 | 2 | 76.8 | 76.8 | 77.0 | 77.0 | 0.00 | 0.05 | 0.00 | 0.04 | 0.08 | 0.06 | 0.08 | 0.06 | 500 |
| 4 | 5 | 500 | 100 | 0 | 0.2 | 2.5 | 0.5 | 1 | 77.9 | 76.6 | 77.6 | 77.4 | 0.00 | 0.05 | 0.01 | 0.05 | 0.07 | 0.05 | 0.07 | 0.06 | 497 |
| 4 | 5 | 500 | 100 | 0 | 0.2 | 2.5 | 0.5 | 2 | 78.4 | 78.4 | 78.6 | 78.4 | 0.00 | 0.05 | 0.01 | 0.05 | 0.07 | 0.05 | 0.07 | 0.06 | 500 |
| 4 | 5 | 500 | 100 | 0 | 0.5 | 1.5 | 2 | 1 | 100.0 | 100.0 | 100.0 | 100.0 | 0.00 | 0.11 | 0.01 | 0.10 | 0.08 | 0.06 | 0.09 | 0.07 | 485 |
| 4 | 5 | 500 | 100 | 0 | 0.5 | 1.5 | 2 | 2 | 100.0 | 100.0 | 100.0 | 100.0 | 0.00 | 0.12 | 0.01 | 0.11 | 0.08 | 0.06 | 0.09 | 0.08 | 500 |
| 4 | 5 | 500 | 100 | 0 | 0.5 | 2.5 | 0.5 | 1 | 100.0 | 100.0 | 100.0 | 100.0 | 0.01 | 0.13 | 0.02 | 0.11 | 0.07 | 0.06 | 0.09 | 0.07 | 492 |
| 4 | 5 | 500 | 100 | 0 | 0.5 | 2.5 | 0.5 | 2 | 100.0 | 100.0 | 100.0 | 100.0 | 0.00 | 0.14 | 0.01 | 0.13 | 0.07 | 0.06 | 0.08 | 0.07 | 500 |
| 4 | 5 | 500 | 100 | 0.5 | 0 | 1.5 | 2 | 1 | 3.8 | 3.6 | 3.8 | 3.6 | 0.00 | 0.00 | 0.00 | 0.00 | 0.07 | 0.05 | 0.07 | 0.05 | 479 |
| 4 | 5 | 500 | 100 | 0.5 | 0 | 1.5 | 2 | 2 | 4.6 | 4.6 | 4.6 | 4.6 | 0.00 | 0.00 | 0.00 | 0.00 | 0.07 | 0.05 | 0.08 | 0.06 | 500 |
| 4 | 5 | 500 | 100 | 0.5 | 0 | 2.5 | 0.5 | 1 | 6.0 | 5.8 | 5.8 | 5.8 | 0.00 | 0.00 | 0.00 | 0.00 | 0.07 | 0.05 | 0.08 | 0.05 | 497 |
| 4 | 5 | 500 | 100 | 0.5 | 0 | 2.5 | 0.5 | 2 | 5.4 | 5.4 | 5.8 | 5.8 | 0.00 | 0.00 | 0.00 | 0.00 | 0.07 | 0.05 | 0.08 | 0.06 | 499 |
| 4 | 5 | 500 | 100 | 0.5 | 0.2 | 1.5 | 2 | 1 | 76.6 | 76.4 | 76.6 | 76.4 | 0.00 | 0.05 | 0.00 | 0.05 | 0.08 | 0.06 | 0.08 | 0.06 | 479 |
| 4 | 5 | 500 | 100 | 0.5 | 0.2 | 1.5 | 2 | 2 | 74.0 | 74.0 | 74.0 | 73.8 | 0.00 | 0.05 | 0.00 | 0.05 | 0.08 | 0.06 | 0.08 | 0.06 | 500 |
| 4 | 5 | 500 | 100 | 0.5 | 0.2 | 2.5 | 0.5 | 1 | 76.3 | 75.0 | 75.0 | 75.0 | 0.00 | 0.06 | 0.00 | 0.06 | 0.07 | 0.05 | 0.08 | 0.06 | 493 |
| 4 | 5 | 500 | 100 | 0.5 | 0.2 | 2.5 | 0.5 | 2 | 77.4 | 77.4 | 77.2 | 77.2 | 0.00 | 0.06 | 0.00 | 0.05 | 0.07 | 0.05 | 0.08 | 0.06 | 500 |
| 4 | 5 | 500 | 100 | 0.5 | 0.5 | 1.5 | 2 | 1 | 100.0 | 100.0 | 100.0 | 100.0 | 0.00 | 0.12 | 0.01 | 0.11 | 0.07 | 0.06 | 0.09 | 0.07 | 481 |
| 4 | 5 | 500 | 100 | 0.5 | 0.5 | 1.5 | 2 | 2 | 100.0 | 100.0 | 100.0 | 100.0 | 0.00 | 0.12 | 0.01 | 0.11 | 0.07 | 0.06 | 0.08 | 0.07 | 500 |
| 4 | 5 | 500 | 100 | 0.5 | 0.5 | 2.5 | 0.5 | 1 | 100.0 | 100.0 | 100.0 | 100.0 | 0.00 | 0.14 | 0.01 | 0.12 | 0.08 | 0.06 | 0.09 | 0.07 | 496 |
| 4 | 5 | 500 | 100 | 0.5 | 0.5 | 2.5 | 0.5 | 2 | 100.0 | 100.0 | 100.0 | 100.0 | 0.00 | 0.14 | 0.01 | 0.13 | 0.08 | 0.06 | 0.09 | 0.08 | 500 |
| 4 | 5 | 500 | 100 | 2 | 0 | 1.5 | 2 | 1 | 5.6 | 5.4 | 5.4 | 5.4 | 0.00 | 0.00 | 0.00 | 0.00 | 0.08 | 0.05 | 0.08 | 0.05 | 485 |
| 4 | 5 | 500 | 100 | 2 | 0 | 1.5 | 2 | 2 | 4.6 | 4.6 | 4.8 | 4.4 | 0.00 | 0.00 | 0.00 | 0.00 | 0.08 | 0.05 | 0.08 | 0.05 | 500 |
| 4 | 5 | 500 | 100 | 2 | 0 | 2.5 | 0.5 | 1 | 5.0 | 5.0 | 5.0 | 5.0 | 0.01 | 0.00 | 0.01 | 0.00 | 0.08 | 0.05 | 0.08 | 0.06 | 498 |
| 4 | 5 | 500 | 100 | 2 | 0.2 | 1.5 | 2 | 1 | 70.8 | 70.8 | 70.8 | 70.6 | 0.00 | 0.07 | 0.01 | 0.06 | 0.08 | 0.05 | 0.09 | 0.06 | 500 |
| 4 | 5 | 500 | 100 | 2 | 0.2 | 1.5 | 2 | 2 | 70.6 | 70.6 | 70.4 | 70.4 | 0.00 | 0.07 | 0.01 | 0.07 | 0.08 | 0.05 | 0.09 | 0.06 | 500 |
| 4 | 5 | 500 | 100 | 2 | 0.2 | 2.5 | 0.5 | 1 | 72.3 | 71.0 | 71.0 | 70.8 | 0.00 | 0.07 | 0.01 | 0.06 | 0.08 | 0.05 | 0.09 | 0.06 | 491 |
| 4 | 5 | 500 | 100 | 2 | 0.2 | 2.5 | 0.5 | 2 | 70.6 | 70.6 | 70.4 | 70.4 | 0.00 | 0.07 | 0.01 | 0.07 | 0.08 | 0.05 | 0.09 | 0.06 | 500 |
| 4 | 5 | 500 | 100 | 2 | 0.5 | 1.5 | 2 | 1 | 100.0 | 100.0 | 100.0 | 100.0 | 0.00 | 0.18 | 0.02 | 0.17 | 0.08 | 0.05 | 0.10 | 0.07 | 483 |
| 4 | 5 | 500 | 100 | 2 | 0.5 | 1.5 | 2 | 2 | 100.0 | 100.0 | 100.0 | 100.0 | 0.00 | 0.18 | 0.02 | 0.17 | 0.08 | 0.06 | 0.10 | 0.07 | 498 |
| 4 | 5 | 500 | 100 | 2 | 0.5 | 2.5 | 0.5 | 1 | 100.0 | 100.0 | 100.0 | 100.0 | 0.01 | 0.17 | 0.02 | 0.16 | 0.08 | 0.05 | 0.09 | 0.07 | 500 |
| 4 | 5 | 500 | 100 | 2 | 0.5 | 2.5 | 0.5 | 1 | 100.0 | 100.0 | 100.0 | 100.0 | 0.01 | 0.17 | 0.02 | 0.15 | 0.08 | 0.06 | 0.10 | 0.07 | 497 |
| 4 | 5 | 500 | 100 | 2 | 0.5 | 2.5 | 0.5 | 2 | 100.0 | 100.0 | 100.0 | 100.0 | 0.01 | 0.17 | 0.02 | 0.16 | 0.08 | 0.05 | 0.09 | 0.07 | 500 |
| 4 | 5 | 500 | 250 | 0 | 0 | 1.5 | 2 | 1 | 5.2 | 5.2 | 5.2 | 5.2 | 0.00 | 0.00 | 0.00 | 0.00 | 0.07 | 0.05 | 0.07 | 0.05 | 500 |
| 4 | 5 | 500 | 250 | 0 | 0 | 1.5 | 2 | 2 | 5.2 | 5.2 | 5.4 | 5.4 | 0.00 | 0.00 | 0.00 | 0.00 | 0.07 | 0.06 | 0.08 | 0.06 | 500 |
| 4 | 5 | 500 | 250 | 0 | 0 | 2.5 | 0.5 | 1 | 5.8 | 5.8 | 5.8 | 5.8 | 0.00 | 0.00 | 0.00 | 0.00 | 0.08 | 0.06 | 0.08 | 0.06 | 500 |
| 4 | 5 | 500 | 250 | 0 | 0 | 2.5 | 0.5 | 2 | 6.6 | 6.6 | 6.8 | 6.6 | 0.01 | 0.01 | 0.01 | 0.01 | 0.08 | 0.06 | 0.08 | 0.06 | 500 |
| 4 | 5 | 500 | 250 | 0 | 0.2 | 1.5 | 2 | 1 | 76.6 | 76.6 | 76.8 | 76.6 | 0.00 | 0.05 | 0.00 | 0.05 | 0.07 | 0.06 | 0.07 | 0.06 | 500 |
| 4 | 5 | 500 | 250 | 0 | 0.2 | 1.5 | 2 | 2 | 77.2 | 77.2 | 77.2 | 77.2 | 0.00 | 0.05 | 0.00 | 0.05 | 0.07 | 0.05 | 0.07 | 0.06 | 500 |
| 4 | 5 | 500 | 250 | 0 | 0.2 | 2.5 | 0.5 | 1 | 76.8 | 76.6 | 76.6 | 76.4 | 0.00 | 0.06 | 0.00 | 0.05 | 0.07 | 0.05 | 0.08 | 0.06 | 500 |
| 4 | 5 | 500 | 250 | 0 | 0.2 | 2.5 | 0.5 | 2 | 76.8 | 76.8 | 76.6 | 76.6 | 0.00 | 0.06 | 0.00 | 0.05 | 0.07 | 0.05 | 0.07 | 0.05 | 500 |
| 4 | 5 | 500 | 250 | 0 | 0.5 | 1.5 | 2 | 1 | 100.0 | 100.0 | 100.0 | 100.0 | 0.00 | 0.12 | 0.00 | 0.11 | 0.07 | 0.06 | 0.08 | 0.06 | 500 |
| 4 | 5 | 500 | 250 | 0 | 0.5 | 1.5 | 2 | 2 | 100.0 | 100.0 | 100.0 | 100.0 | 0.00 | 0.12 | 0.00 | 0.11 | 0.07 | 0.06 | 0.08 | 0.06 | 500 |
| 4 | 5 | 500 | 250 | 0 | 0.5 | 2.5 | 0.5 | 1 | 100.0 | 100.0 | 100.0 | 100.0 | 0.01 | 0.13 | 0.01 | 0.13 | 0.08 | 0.06 | 0.08 | 0.06 | 500 |
| 4 | 5 | 500 | 250 | 0 | 0.5 | 2.5 | 0.5 | 2 | 100.0 | 100.0 | 100.0 | 100.0 | 0.01 | 0.13 | 0.01 | 0.13 | 0.08 | 0.06 | 0.08 | 0.06 | 500 |
| 4 | 5 | 500 | 250 | 0.5 | 0 | 1.5 | 2 | 1 | 4.6 | 4.6 | 4.6 | 4.6 | 0.00 | 0.00 | 0.00 | 0.00 | 0.07 | 0.06 | 0.07 | 0.06 | 500 |
| 4 | 5 | 500 | 250 | 0.5 | 0 | 1.5 | 2 | 2 | 5.2 | 5.2 | 5.4 | 5.2 | 0.00 | 0.00 | 0.00 | 0.00 | 0.08 | 0.06 | 0.08 | 0.06 | 500 |
| 4 | 5 | 500 | 250 | 0.5 | 0 | 2.5 | 0.5 | 1 | 3.4 | 3.4 | 3.4 | 3.4 | 0.00 | 0.00 | 0.00 | 0.00 | 0.07 | 0.05 | 0.07 | 0.05 | 500 |
| 4 | 5 | 500 | 250 | 0.5 | 0 | 2.5 | 0.5 | 2 | 4.4 | 4.4 | 4.4 | 4.4 | 0.00 | 0.00 | 0.00 | 0.00 | 0.07 | 0.05 | 0.07 | 0.05 | 500 |
| 4 | 5 | 500 | 250 | 0.5 | 0.2 | 1.5 | 2 | 1 | 75.6 | 75.6 | 75.8 | 75.8 | 0.00 | 0.05 | 0.00 | 0.05 | 0.08 | 0.06 | 0.08 | 0.06 | 500 |
| 4 | 5 | 500 | 250 | 0.5 | 0.2 | 1.5 | 2 | 2 | 77.6 | 77.6 | 77.6 | 77.6 | 0.00 | 0.05 | 0.01 | 0.05 | 0.07 | 0.06 | 0.08 | 0.06 | 500 |
| 4 | 5 | 500 | 250 | 0.5 | 0.2 | 2.5 | 0.5 | 1 | 75.0 | 75.0 | 75.4 | 74.8 | 0.00 | 0.06 | 0.00 | 0.06 | 0.08 | 0.06 | 0.08 | 0.06 | 500 |
| 4 | 5 | 500 | 250 | 0.5 | 0.2 | 2.5 | 0.5 | 2 | 77.8 | 77.8 | 77.8 | 77.8 | 0.00 | 0.05 | 0.01 | 0.05 | 0.08 | 0.05 | 0.08 | 0.06 | 500 |
| 4 | 5 | 500 | 250 | 0.5 | 0.5 | 1.5 | 2 | 1 | 100.0 | 100.0 | 100.0 | 100.0 | 0.00 | 0.12 | 0.01 | 0.11 | 0.07 | 0.06 | 0.07 | 0.06 | 500 |
| 4 | 5 | 500 | 250 | 0.5 | 0.5 | 1.5 | 2 | 2 | 100.0 | 100.0 | 100.0 | 100.0 | 0.00 | 0.12 | 0.00 | 0.12 | 0.07 | 0.06 | 0.08 | 0.06 | 500 |
| 4 | 5 | 500 | 250 | 0.5 | 0.5 | 2.5 | 0.5 | 1 | 100.0 | 100.0 | 100.0 | 100.0 | 0.00 | 0.13 | 0.01 | 0.13 | 0.08 | 0.06 | 0.08 | 0.06 | 500 |
| 4 | 5 | 500 | 250 | 0.5 | 0.5 | 2.5 | 0.5 | 2 | 100.0 | 100.0 | 100.0 | 100.0 | 0.01 | 0.13 | 0.01 | 0.13 | 0.07 | 0.06 | 0.08 | 0.06 | 500 |
| 4 | 5 | 500 | 250 | 2 | 0 | 1.5 | 2 | 1 | 4.2 | 4.2 | 4.2 | 4.2 | 0.00 | 0.00 | 0.00 | 0.00 | 0.08 | 0.05 | 0.08 | 0.05 | 500 |
| 4 | 5 | 500 | 250 | 2 | 0 | 1.5 | 2 | 2 | 4.2 | 4.2 | 4.2 | 4.2 | 0.00 | 0.00 | 0.00 | 0.00 | 0.08 | 0.05 | 0.08 | 0.05 | 500 |
| 4 | 5 | 500 | 250 | 2 | 0 | 2.5 | 0.5 | 1 | 3.2 | 3.2 | 3.0 | 3.0 | 0.00 | 0.00 | 0.00 | 0.00 | 0.07 | 0.05 | 0.07 | 0.05 | 500 |
| 4 | 5 | 500 | 250 | 2 | 0 | 2.5 | 0.5 | 2 | 3.2 | 2.8 | 3.2 | 2.8 | 0.00 | 0.00 | 0.00 | 0.00 | 0.07 | 0.05 | 0.07 | 0.05 | 500 |
| 4 | 5 | 500 | 250 | 2 | 0.2 | 1.5 | 2 | 1 | 69.8 | 69.8 | 69.4 | 69.4 | 0.00 | 0.08 | 0.00 | 0.07 | 0.08 | 0.05 | 0.08 | 0.05 | 500 |
| 4 | 5 | 500 | 250 | 2 | 0.2 | 1.5 | 2 | 2 | 70.8 | 70.8 | 70.8 | 70.6 | 0.00 | 0.08 | 0.00 | 0.07 | 0.08 | 0.05 | 0.08 | 0.05 | 500 |
| 4 | 5 | 500 | 250 | 2 | 0.2 | 2.5 | 0.5 | 1 | 73.2 | 73.2 | 73.6 | 73.2 | 0.01 | 0.07 | 0.01 | 0.06 | 0.08 | 0.05 | 0.08 | 0.06 | 500 |
| 4 | 5 | 500 | 250 | 2 | 0.2 | 2.5 | 0.5 | 2 | 72.8 | 72.6 | 72.8 | 72.8 | 0.00 | 0.07 | 0.00 | 0.07 | 0.08 | 0.05 | 0.08 | 0.05 | 500 |
| 4 | 5 | 500 | 250 | 2 | 0.5 | 1.5 | 2 | 1 | 100.0 | 100.0 | 100.0 | 100.0 | 0.00 | 0.18 | 0.01 | 0.18 | 0.09 | 0.06 | 0.09 | 0.07 | 500 |
| 4 | 5 | 500 | 250 | 2 | 0.5 | 1.5 | 2 | 2 | 100.0 | 100.0 | 100.0 | 100.0 | 0.01 | 0.19 | 0.00 | 0.18 | 0.08 | 0.06 | 0.09 | 0.06 | 500 |
| 4 | 5 | 500 | 250 | 2 | 0.5 | 2.5 | 0.5 | 1 | 100.0 | 100.0 | 100.0 | 100.0 | 0.00 | 0.17 | 0.01 | 0.17 | 0.08 | 0.06 | 0.08 | 0.06 | 500 |
| 4 | 5 | 500 | 250 | 2 | 0.5 | 2.5 | 0.5 | 2 | 100.0 | 100.0 | 100.0 | 100.0 | 0.00 | 0.18 | 0.00 | 0.17 | 0.08 | 0.06 | 0.08 | 0.06 | 500 |
| 4 | 5 | 500 | 500 | 0 | 0 | 1.5 | 2 | 1 | 5.8 | 5.8 | 6.0 | 6.0 | 0.00 | 0.00 | 0.00 | 0.00 | 0.08 | 0.06 | 0.08 | 0.06 | 500 |
| 4 | 5 | 500 | 500 | 0 | 0 | 1.5 | 2 | 2 | 6.0 | 5.8 | 6.0 | 6.0 | 0.00 | 0.00 | 0.00 | 0.00 | 0.07 | 0.05 | 0.07 | 0.05 | 500 |
| 4 | 5 | 500 | 500 | 0 | 0 | 2.5 | 0.5 | 1 | 4.6 | 4.4 | 4.4 | 4.4 | 0.00 | 0.00 | 0.00 | 0.00 | 0.07 | 0.05 | 0.07 | 0.05 | 500 |
| 4 | 5 | 500 | 500 | 0 | 0 | 2.5 | 0.5 | 2 | 4.8 | 4.8 | 4.8 | 4.8 | 0.01 | 0.00 | 0.01 | 0.00 | 0.07 | 0.05 | 0.07 | 0.05 | 500 |
| 4 | 5 | 500 | 500 | 0 | 0.2 | 1.5 | 2 | 1 | 79.4 | 79.4 | 79.2 | 79.2 | 0.00 | 0.05 | 0.00 | 0.05 | 0.07 | 0.06 | 0.07 | 0.06 | 500 |
| 4 | 5 | 500 | 500 | 0 | 0.2 | 1.5 | 2 | 2 | 80.0 | 80.0 | 80.2 | 80.2 | 0.00 | 0.05 | 0.00 | 0.05 | 0.07 | 0.05 | 0.07 | 0.05 | 500 |
| 4 | 5 | 500 | 500 | 0 | 0.2 | 2.5 | 0.5 | 1 | 78.4 | 78.4 | 78.2 | 78.2 | 0.01 | 0.05 | 0.01 | 0.05 | 0.08 | 0.06 | 0.08 | 0.06 | 500 |
| 4 | 5 | 500 | 500 | 0 | 0.2 | 2.5 | 0.5 | 2 | 75.4 | 75.2 | 75.8 | 75.6 | 0.00 | 0.06 | 0.00 | 0.06 | 0.07 | 0.05 | 0.07 | 0.05 | 500 |
| 4 | 5 | 500 | 500 | 0 | 0.5 | 1.5 | 2 | 1 | 100.0 | 100.0 | 100.0 | 100.0 | 0.00 | 0.12 | 0.00 | 0.12 | 0.07 | 0.06 | 0.07 | 0.06 | 500 |
| 4 | 5 | 500 | 500 | 0 | 0.5 | 1.5 | 2 | 2 | 100.0 | 100.0 | 100.0 | 100.0 | 0.00 | 0.12 | 0.00 | 0.11 | 0.08 | 0.06 | 0.08 | 0.06 | 500 |
| 4 | 5 | 500 | 500 | 0 | 0.5 | 2.5 | 0.5 | 1 | 100.0 | 100.0 | 100.0 | 100.0 | 0.00 | 0.13 | 0.00 | 0.13 | 0.08 | 0.06 | 0.08 | 0.06 | 500 |
| 4 | 5 | 500 | 500 | 0 | 0.5 | 2.5 | 0.5 | 2 | 100.0 | 100.0 | 100.0 | 100.0 | 0.00 | 0.14 | 0.00 | 0.13 | 0.08 | 0.06 | 0.08 | 0.06 | 500 |
| 4 | 5 | 500 | 500 | 0.5 | 0 | 1.5 | 2 | 1 | 3.8 | 3.8 | 3.8 | 3.8 | 0.00 | 0.00 | 0.00 | 0.00 | 0.07 | 0.05 | 0.07 | 0.05 | 500 |
| 4 | 5 | 500 | 500 | 0.5 | 0 | 1.5 | 2 | 2 | 4.8 | 4.8 | 4.8 | 4.8 | 0.00 | 0.00 | 0.00 | 0.00 | 0.07 | 0.06 | 0.07 | 0.06 | 500 |
| 4 | 5 | 500 | 500 | 0.5 | 0 | 2.5 | 0.5 | 1 | 5.6 | 5.6 | 5.6 | 5.4 | 0.00 | 0.00 | 0.00 | 0.00 | 0.08 | 0.05 | 0.08 | 0.06 | 500 |
| 4 | 5 | 500 | 500 | 0.5 | 0 | 2.5 | 0.5 | 2 | 5.6 | 5.6 | 5.6 | 5.6 | 0.00 | 0.00 | 0.00 | 0.00 | 0.08 | 0.05 | 0.08 | 0.05 | 500 |
| 4 | 5 | 500 | 500 | 0.5 | 0.2 | 1.5 | 2 | 1 | 77.0 | 77.0 | 77.0 | 76.8 | 0.00 | 0.05 | 0.00 | 0.05 | 0.07 | 0.05 | 0.07 | 0.06 | 500 |
| 4 | 5 | 500 | 500 | 0.5 | 0.2 | 1.5 | 2 | 2 | 75.2 | 75.2 | 75.2 | 75.2 | 0.00 | 0.05 | 0.00 | 0.05 | 0.08 | 0.06 | 0.08 | 0.06 | 500 |
| 4 | 5 | 500 | 500 | 0.5 | 0.2 | 2.5 | 0.5 | 1 | 74.4 | 74.4 | 74.6 | 74.2 | 0.00 | 0.06 | 0.00 | 0.06 | 0.08 | 0.05 | 0.08 | 0.06 | 500 |
| 4 | 5 | 500 | 500 | 0.5 | 0.2 | 2.5 | 0.5 | 2 | 79.4 | 79.4 | 79.4 | 79.4 | 0.00 | 0.06 | 0.00 | 0.06 | 0.07 | 0.05 | 0.07 | 0.05 | 500 |
| 4 | 5 | 500 | 500 | 0.5 | 0.5 | 1.5 | 2 | 1 | 100.0 | 100.0 | 100.0 | 100.0 | 0.00 | 0.12 | 0.01 | 0.12 | 0.08 | 0.06 | 0.08 | 0.06 | 500 |
| 4 | 5 | 500 | 500 | 0.5 | 0.5 | 1.5 | 2 | 2 | 100.0 | 100.0 | 100.0 | 100.0 | 0.00 | 0.12 | 0.00 | 0.12 | 0.08 | 0.06 | 0.08 | 0.06 | 500 |
| 4 | 5 | 500 | 500 | 0.5 | 0.5 | 2.5 | 0.5 | 1 | 100.0 | 100.0 | 100.0 | 100.0 | 0.00 | 0.13 | 0.00 | 0.13 | 0.08 | 0.06 | 0.08 | 0.06 | 500 |
| 4 | 5 | 500 | 500 | 0.5 | 0.5 | 2.5 | 0.5 | 2 | 100.0 | 100.0 | 100.0 | 100.0 | 0.00 | 0.14 | 0.00 | 0.13 | 0.08 | 0.06 | 0.08 | 0.06 | 500 |
| 4 | 5 | 500 | 500 | 2 | 0 | 1.5 | 2 | 1 | 3.4 | 3.4 | 3.4 | 3.4 | 0.00 | 0.00 | 0.00 | 0.00 | 0.07 | 0.05 | 0.07 | 0.05 | 500 |
| 4 | 5 | 500 | 500 | 2 | 0 | 1.5 | 2 | 2 | 5.4 | 5.4 | 5.4 | 5.4 | 0.00 | 0.00 | 0.00 | 0.00 | 0.08 | 0.05 | 0.08 | 0.05 | 500 |
| 4 | 5 | 500 | 500 | 2 | 0 | 2.5 | 0.5 | 1 | 6.4 | 6.4 | 6.4 | 6.4 | 0.01 | 0.00 | 0.01 | 0.00 | 0.08 | 0.05 | 0.08 | 0.05 | 500 |
| 4 | 5 | 500 | 500 | 2 | 0 | 2.5 | 0.5 | 2 | 5.8 | 5.8 | 6.2 | 6.2 | 0.00 | 0.00 | 0.00 | 0.00 | 0.08 | 0.05 | 0.08 | 0.05 | 500 |
| 4 | 5 | 500 | 500 | 2 | 0.2 | 1.5 | 2 | 1 | 74.6 | 74.6 | 74.8 | 74.8 | 0.01 | 0.07 | 0.01 | 0.07 | 0.08 | 0.05 | 0.08 | 0.05 | 500 |
| 4 | 5 | 500 | 500 | 2 | 0.2 | 1.5 | 2 | 2 | 69.4 | 69.4 | 69.4 | 69.4 | 0.00 | 0.08 | 0.00 | 0.08 | 0.08 | 0.05 | 0.08 | 0.05 | 500 |
| 4 | 5 | 500 | 500 | 2 | 0.2 | 2.5 | 0.5 | 1 | 73.4 | 73.4 | 73.4 | 73.2 | 0.01 | 0.07 | 0.01 | 0.07 | 0.08 | 0.05 | 0.08 | 0.05 | 500 |
| 4 | 5 | 500 | 500 | 2 | 0.2 | 2.5 | 0.5 | 2 | 71.4 | 71.4 | 71.6 | 71.4 | 0.00 | 0.07 | 0.00 | 0.07 | 0.08 | 0.05 | 0.08 | 0.05 | 500 |
| 4 | 5 | 500 | 500 | 2 | 0.5 | 1.5 | 2 | 1 | 100.0 | 100.0 | 100.0 | 100.0 | 0.00 | 0.18 | 0.00 | 0.18 | 0.08 | 0.06 | 0.09 | 0.06 | 500 |
| 4 | 5 | 500 | 500 | 2 | 0.5 | 1.5 | 2 | 2 | 100.0 | 100.0 | 100.0 | 100.0 | 0.00 | 0.18 | 0.00 | 0.18 | 0.08 | 0.05 | 0.08 | 0.06 | 500 |
| 4 | 5 | 500 | 500 | 2 | 0.5 | 2.5 | 0.5 | 1 | 100.0 | 100.0 | 100.0 | 100.0 | 0.00 | 0.17 | 0.00 | 0.17 | 0.08 | 0.06 | 0.09 | 0.06 | 500 |
| 4 | 5 | 500 | 500 | 2 | 0.5 | 2.5 | 0.5 | 2 | 100.0 | 100.0 | 100.0 | 100.0 | 0.00 | 0.17 | 0.00 | 0.17 | 0.08 | 0.06 | 0.08 | 0.06 | 500 |
| 7 | 3 | 50 | 100 | 0 | 0 | 1.5 | 2 | 1 | 4.0 | 3.8 | 4.2 | 3.8 | 0.02 | 0.01 | 0.02 | 0.01 | 0.24 | 0.17 | 0.23 | 0.17 | 500 |
| 7 | 3 | 50 | 100 | 0 | 0 | 1.5 | 2 | 2 | 4.0 | 3.8 | 4.2 | 3.8 | 0.02 | 0.01 | 0.02 | 0.01 | 0.24 | 0.17 | 0.23 | 0.17 | 500 |
| 7 | 3 | 50 | 100 | 0 | 0 | 2.5 | 0.5 | 1 | 4.6 | 4.6 | 4.6 | 4.6 | 0.02 | 0.01 | 0.02 | 0.01 | 0.23 | 0.16 | 0.22 | 0.16 | 500 |
| 7 | 3 | 50 | 100 | 0 | 0 | 2.5 | 0.5 | 2 | 6.2 | 5.6 | 6.4 | 5.8 | 0.01 | 0.00 | 0.01 | 0.00 | 0.24 | 0.17 | 0.24 | 0.17 | 500 |
| 7 | 3 | 50 | 100 | 0 | 0.2 | 1.5 | 2 | 1 | 11.4 | 10.6 | 11.4 | 10.8 | 0.01 | 0.06 | 0.01 | 0.06 | 0.24 | 0.18 | 0.24 | 0.18 | 500 |
| 7 | 3 | 50 | 100 | 0 | 0.2 | 1.5 | 2 | 2 | 14.0 | 13.6 | 14.0 | 13.6 | 0.01 | 0.06 | 0.01 | 0.06 | 0.24 | 0.18 | 0.25 | 0.18 | 500 |
| 7 | 3 | 50 | 100 | 0 | 0.2 | 2.5 | 0.5 | 1 | 13.0 | 12.2 | 13.6 | 12.2 | 0.01 | 0.07 | 0.01 | 0.06 | 0.24 | 0.18 | 0.25 | 0.18 | 500 |
| 7 | 3 | 50 | 100 | 0 | 0.2 | 2.5 | 0.5 | 2 | 15.0 | 14.8 | 15.2 | 14.8 | 0.00 | 0.05 | 0.00 | 0.05 | 0.25 | 0.18 | 0.25 | 0.18 | 500 |
| 7 | 3 | 50 | 100 | 0 | 0.5 | 1.5 | 2 | 1 | 54.0 | 53.4 | 54.6 | 53.6 | 0.01 | 0.12 | 0.00 | 0.13 | 0.24 | 0.18 | 0.23 | 0.18 | 500 |
| 7 | 3 | 50 | 100 | 0 | 0.5 | 1.5 | 2 | 2 | 56.2 | 55.8 | 56.4 | 55.6 | 0.02 | 0.12 | 0.01 | 0.12 | 0.24 | 0.18 | 0.23 | 0.18 | 500 |
| 7 | 3 | 50 | 100 | 0 | 0.5 | 2.5 | 0.5 | 1 | 57.2 | 55.0 | 57.6 | 55.0 | 0.01 | 0.13 | 0.01 | 0.13 | 0.24 | 0.19 | 0.24 | 0.18 | 500 |
| 7 | 3 | 50 | 100 | 0 | 0.5 | 2.5 | 0.5 | 2 | 57.4 | 56.6 | 58.0 | 56.4 | 0.02 | 0.12 | 0.02 | 0.12 | 0.24 | 0.18 | 0.24 | 0.18 | 500 |
| 7 | 3 | 50 | 100 | 0.5 | 0 | 1.5 | 2 | 1 | 5.6 | 4.8 | 5.6 | 4.8 | 0.01 | 0.01 | 0.01 | 0.01 | 0.23 | 0.17 | 0.23 | 0.17 | 500 |
| 7 | 3 | 50 | 100 | 0.5 | 0 | 1.5 | 2 | 2 | 5.6 | 4.8 | 5.6 | 4.8 | 0.01 | 0.01 | 0.01 | 0.01 | 0.23 | 0.17 | 0.23 | 0.17 | 500 |
| 7 | 3 | 50 | 100 | 0.5 | 0 | 2.5 | 0.5 | 1 | 4.4 | 4.2 | 4.4 | 4.2 | 0.00 | 0.00 | 0.00 | 0.00 | 0.24 | 0.17 | 0.24 | 0.17 | 500 |
| 7 | 3 | 50 | 100 | 0.5 | 0 | 2.5 | 0.5 | 2 | 5.2 | 5.2 | 5.4 | 5.2 | 0.00 | 0.00 | 0.00 | 0.00 | 0.24 | 0.17 | 0.24 | 0.17 | 500 |
| 7 | 3 | 50 | 100 | 0.5 | 0.2 | 1.5 | 2 | 1 | 11.2 | 10.2 | 11.8 | 10.2 | 0.01 | 0.06 | 0.01 | 0.06 | 0.23 | 0.17 | 0.23 | 0.17 | 500 |
| 7 | 3 | 50 | 100 | 0.5 | 0.2 | 1.5 | 2 | 2 | 15.8 | 14.0 | 16.2 | 14.2 | 0.01 | 0.05 | 0.01 | 0.05 | 0.24 | 0.18 | 0.25 | 0.18 | 500 |
| 7 | 3 | 50 | 100 | 0.5 | 0.2 | 2.5 | 0.5 | 1 | 15.2 | 14.8 | 15.2 | 14.8 | 0.01 | 0.05 | 0.01 | 0.05 | 0.25 | 0.18 | 0.25 | 0.18 | 500 |
| 7 | 3 | 50 | 100 | 0.5 | 0.2 | 2.5 | 0.5 | 2 | 15.4 | 15.0 | 15.4 | 14.8 | 0.01 | 0.05 | 0.01 | 0.05 | 0.25 | 0.18 | 0.25 | 0.18 | 500 |
| 7 | 3 | 50 | 100 | 0.5 | 0.5 | 1.5 | 2 | 1 | 58.4 | 57.2 | 59.2 | 57.2 | 0.01 | 0.12 | 0.01 | 0.13 | 0.23 | 0.18 | 0.23 | 0.18 | 500 |
| 7 | 3 | 50 | 100 | 0.5 | 0.5 | 1.5 | 2 | 2 | 55.8 | 54.8 | 55.8 | 54.8 | 0.01 | 0.14 | 0.01 | 0.14 | 0.24 | 0.18 | 0.24 | 0.18 | 500 |
| 7 | 3 | 50 | 100 | 0.5 | 0.5 | 2.5 | 0.5 | 1 | 58.8 | 57.4 | 59.0 | 57.2 | 0.02 | 0.13 | 0.02 | 0.13 | 0.25 | 0.19 | 0.25 | 0.19 | 500 |
| 7 | 3 | 50 | 100 | 0.5 | 0.5 | 2.5 | 0.5 | 2 | 53.8 | 52.6 | 53.8 | 52.6 | 0.00 | 0.13 | 0.01 | 0.13 | 0.25 | 0.19 | 0.25 | 0.19 | 500 |
| 7 | 3 | 50 | 100 | 2 | 0 | 1.5 | 2 | 1 | 3.8 | 3.4 | 3.8 | 3.6 | 0.01 | 0.01 | 0.01 | 0.01 | 0.26 | 0.15 | 0.26 | 0.15 | 500 |
| 7 | 3 | 50 | 100 | 2 | 0 | 1.5 | 2 | 2 | 3.8 | 3.4 | 3.8 | 3.6 | 0.01 | 0.01 | 0.01 | 0.01 | 0.26 | 0.15 | 0.26 | 0.15 | 500 |
| 7 | 3 | 50 | 100 | 2 | 0 | 2.5 | 0.5 | 1 | 5.0 | 4.8 | 5.0 | 4.8 | 0.00 | 0.00 | 0.00 | 0.00 | 0.26 | 0.16 | 0.26 | 0.16 | 500 |
| 7 | 3 | 50 | 100 | 2 | 0 | 2.5 | 0.5 | 2 | 5.0 | 4.8 | 5.0 | 4.8 | 0.01 | 0.00 | 0.01 | 0.00 | 0.27 | 0.17 | 0.27 | 0.17 | 500 |
| 7 | 3 | 50 | 100 | 2 | 0.2 | 1.5 | 2 | 1 | 11.4 | 11.2 | 12.0 | 11.2 | 0.00 | 0.08 | 0.00 | 0.08 | 0.26 | 0.16 | 0.27 | 0.16 | 500 |
| 7 | 3 | 50 | 100 | 2 | 0.2 | 1.5 | 2 | 2 | 11.6 | 11.0 | 11.8 | 10.8 | 0.00 | 0.08 | 0.00 | 0.08 | 0.26 | 0.16 | 0.26 | 0.16 | 500 |
| 7 | 3 | 50 | 100 | 2 | 0.2 | 2.5 | 0.5 | 1 | 15.4 | 14.0 | 15.4 | 14.0 | 0.00 | 0.08 | 0.00 | 0.08 | 0.28 | 0.17 | 0.28 | 0.17 | 500 |
| 7 | 3 | 50 | 100 | 2 | 0.2 | 2.5 | 0.5 | 2 | 10.2 | 9.4 | 10.4 | 9.4 | 0.00 | 0.08 | 0.00 | 0.08 | 0.25 | 0.15 | 0.25 | 0.15 | 500 |
| 7 | 3 | 50 | 100 | 2 | 0.5 | 1.5 | 2 | 1 | 52.2 | 51.2 | 52.4 | 51.2 | 0.03 | 0.18 | 0.03 | 0.18 | 0.26 | 0.17 | 0.26 | 0.17 | 500 |
| 7 | 3 | 50 | 100 | 2 | 0.5 | 1.5 | 2 | 2 | 46.4 | 44.8 | 47.4 | 44.8 | 0.00 | 0.20 | 0.00 | 0.19 | 0.27 | 0.17 | 0.28 | 0.18 | 500 |
| 7 | 3 | 50 | 100 | 2 | 0.5 | 2.5 | 0.5 | 1 | 48.2 | 47.2 | 48.4 | 47.2 | 0.01 | 0.19 | 0.01 | 0.19 | 0.27 | 0.18 | 0.27 | 0.18 | 500 |
| 7 | 3 | 50 | 100 | 2 | 0.5 | 2.5 | 0.5 | 2 | 46.4 | 44.8 | 46.6 | 45.0 | 0.00 | 0.19 | 0.01 | 0.19 | 0.26 | 0.17 | 0.26 | 0.17 | 500 |
| 7 | 3 | 50 | 250 | 0 | 0 | 1.5 | 2 | 1 | 7.2 | 6.4 | 7.2 | 6.4 | 0.01 | 0.01 | 0.01 | 0.01 | 0.25 | 0.18 | 0.25 | 0.18 | 500 |
| 7 | 3 | 50 | 250 | 0 | 0 | 1.5 | 2 | 2 | 7.8 | 6.8 | 7.8 | 6.8 | 0.01 | 0.01 | 0.01 | 0.01 | 0.25 | 0.18 | 0.25 | 0.18 | 500 |
| 7 | 3 | 50 | 250 | 0 | 0 | 2.5 | 0.5 | 1 | 5.2 | 4.6 | 5.2 | 4.6 | 0.02 | 0.01 | 0.02 | 0.01 | 0.24 | 0.17 | 0.24 | 0.17 | 500 |
| 7 | 3 | 50 | 250 | 0 | 0 | 2.5 | 0.5 | 2 | 4.0 | 4.0 | 4.2 | 4.0 | 0.02 | 0.02 | 0.02 | 0.01 | 0.23 | 0.16 | 0.23 | 0.16 | 500 |
| 7 | 3 | 50 | 250 | 0 | 0.2 | 1.5 | 2 | 1 | 11.4 | 10.8 | 11.8 | 10.8 | 0.01 | 0.04 | 0.01 | 0.05 | 0.22 | 0.17 | 0.22 | 0.16 | 500 |
| 7 | 3 | 50 | 250 | 0 | 0.2 | 1.5 | 2 | 2 | 11.4 | 11.2 | 11.4 | 11.2 | 0.00 | 0.06 | 0.00 | 0.06 | 0.23 | 0.17 | 0.22 | 0.16 | 500 |
| 7 | 3 | 50 | 250 | 0 | 0.2 | 2.5 | 0.5 | 1 | 14.0 | 13.4 | 14.2 | 13.2 | 0.00 | 0.06 | 0.00 | 0.06 | 0.24 | 0.18 | 0.24 | 0.17 | 500 |
| 7 | 3 | 50 | 250 | 0 | 0.2 | 2.5 | 0.5 | 2 | 16.8 | 16.6 | 16.8 | 16.6 | 0.01 | 0.05 | 0.01 | 0.05 | 0.26 | 0.19 | 0.26 | 0.19 | 500 |
| 7 | 3 | 50 | 250 | 0 | 0.5 | 1.5 | 2 | 1 | 57.8 | 57.0 | 58.4 | 56.8 | 0.02 | 0.11 | 0.01 | 0.12 | 0.24 | 0.18 | 0.23 | 0.18 | 500 |
| 7 | 3 | 50 | 250 | 0 | 0.5 | 1.5 | 2 | 2 | 59.6 | 58.6 | 59.8 | 58.6 | 0.01 | 0.12 | 0.00 | 0.13 | 0.23 | 0.18 | 0.23 | 0.18 | 500 |
| 7 | 3 | 50 | 250 | 0 | 0.5 | 2.5 | 0.5 | 1 | 54.0 | 53.2 | 54.4 | 53.4 | 0.01 | 0.14 | 0.01 | 0.15 | 0.23 | 0.18 | 0.23 | 0.18 | 500 |
| 7 | 3 | 50 | 250 | 0 | 0.5 | 2.5 | 0.5 | 2 | 55.4 | 54.0 | 55.8 | 53.8 | 0.00 | 0.13 | 0.00 | 0.14 | 0.25 | 0.19 | 0.24 | 0.18 | 500 |
| 7 | 3 | 50 | 250 | 0.5 | 0 | 1.5 | 2 | 1 | 6.0 | 5.6 | 6.0 | 5.6 | 0.01 | 0.00 | 0.01 | 0.00 | 0.24 | 0.18 | 0.24 | 0.17 | 500 |
| 7 | 3 | 50 | 250 | 0.5 | 0 | 1.5 | 2 | 2 | 6.0 | 5.6 | 6.0 | 5.6 | 0.01 | 0.00 | 0.01 | 0.00 | 0.24 | 0.18 | 0.24 | 0.17 | 500 |
| 7 | 3 | 50 | 250 | 0.5 | 0 | 2.5 | 0.5 | 1 | 5.0 | 5.0 | 5.2 | 5.0 | 0.00 | 0.00 | 0.00 | 0.00 | 0.25 | 0.18 | 0.24 | 0.17 | 500 |
| 7 | 3 | 50 | 250 | 0.5 | 0 | 2.5 | 0.5 | 2 | 4.6 | 4.2 | 4.6 | 4.2 | 0.01 | 0.00 | 0.01 | 0.01 | 0.24 | 0.17 | 0.24 | 0.17 | 500 |
| 7 | 3 | 50 | 250 | 0.5 | 0.2 | 1.5 | 2 | 1 | 12.6 | 12.2 | 12.6 | 12.2 | 0.00 | 0.06 | 0.00 | 0.06 | 0.24 | 0.18 | 0.24 | 0.17 | 500 |
| 7 | 3 | 50 | 250 | 0.5 | 0.2 | 1.5 | 2 | 2 | 14.8 | 14.2 | 15.0 | 14.2 | 0.00 | 0.05 | 0.00 | 0.05 | 0.23 | 0.17 | 0.23 | 0.17 | 500 |
| 7 | 3 | 50 | 250 | 0.5 | 0.2 | 2.5 | 0.5 | 1 | 16.0 | 14.8 | 16.2 | 14.8 | 0.01 | 0.05 | 0.01 | 0.05 | 0.25 | 0.18 | 0.25 | 0.18 | 500 |
| 7 | 3 | 50 | 250 | 0.5 | 0.2 | 2.5 | 0.5 | 2 | 13.6 | 12.8 | 13.8 | 12.6 | 0.00 | 0.05 | 0.00 | 0.06 | 0.24 | 0.17 | 0.24 | 0.17 | 500 |
| 7 | 3 | 50 | 250 | 0.5 | 0.5 | 1.5 | 2 | 1 | 53.8 | 53.4 | 54.4 | 53.4 | 0.00 | 0.13 | 0.00 | 0.13 | 0.24 | 0.18 | 0.23 | 0.18 | 500 |
| 7 | 3 | 50 | 250 | 0.5 | 0.5 | 1.5 | 2 | 2 | 52.6 | 52.2 | 52.8 | 52.2 | 0.01 | 0.14 | 0.01 | 0.14 | 0.25 | 0.19 | 0.24 | 0.19 | 500 |
| 7 | 3 | 50 | 250 | 0.5 | 0.5 | 2.5 | 0.5 | 1 | 56.0 | 55.0 | 56.0 | 55.0 | 0.01 | 0.13 | 0.00 | 0.14 | 0.24 | 0.18 | 0.24 | 0.18 | 500 |
| 7 | 3 | 50 | 250 | 0.5 | 0.5 | 2.5 | 0.5 | 2 | 51.2 | 50.8 | 51.6 | 51.0 | 0.03 | 0.16 | 0.03 | 0.16 | 0.26 | 0.19 | 0.26 | 0.19 | 500 |
| 7 | 3 | 50 | 250 | 2 | 0 | 1.5 | 2 | 1 | 5.6 | 5.0 | 5.6 | 5.0 | 0.02 | 0.01 | 0.02 | 0.01 | 0.27 | 0.16 | 0.27 | 0.16 | 500 |
| 7 | 3 | 50 | 250 | 2 | 0 | 1.5 | 2 | 2 | 5.6 | 5.0 | 5.6 | 5.0 | 0.02 | 0.01 | 0.02 | 0.01 | 0.27 | 0.16 | 0.27 | 0.16 | 500 |
| 7 | 3 | 50 | 250 | 2 | 0 | 2.5 | 0.5 | 1 | 5.6 | 5.0 | 5.8 | 5.2 | 0.00 | 0.00 | 0.00 | 0.00 | 0.27 | 0.16 | 0.27 | 0.16 | 500 |
| 7 | 3 | 50 | 250 | 2 | 0 | 2.5 | 0.5 | 2 | 3.8 | 3.4 | 3.8 | 3.6 | 0.00 | 0.00 | 0.00 | 0.00 | 0.26 | 0.16 | 0.26 | 0.16 | 500 |
| 7 | 3 | 50 | 250 | 2 | 0.2 | 1.5 | 2 | 1 | 11.0 | 10.4 | 11.0 | 10.4 | 0.01 | 0.09 | 0.01 | 0.09 | 0.26 | 0.15 | 0.26 | 0.15 | 500 |
| 7 | 3 | 50 | 250 | 2 | 0.2 | 1.5 | 2 | 2 | 12.6 | 12.4 | 13.0 | 12.4 | 0.00 | 0.09 | 0.00 | 0.08 | 0.27 | 0.16 | 0.27 | 0.16 | 500 |
| 7 | 3 | 50 | 250 | 2 | 0.2 | 2.5 | 0.5 | 1 | 11.6 | 11.4 | 12.4 | 11.2 | 0.00 | 0.08 | 0.00 | 0.08 | 0.25 | 0.16 | 0.25 | 0.16 | 500 |
| 7 | 3 | 50 | 250 | 2 | 0.2 | 2.5 | 0.5 | 2 | 13.6 | 12.8 | 13.6 | 12.8 | 0.02 | 0.07 | 0.01 | 0.07 | 0.27 | 0.17 | 0.27 | 0.17 | 500 |
| 7 | 3 | 50 | 250 | 2 | 0.5 | 1.5 | 2 | 1 | 49.0 | 47.8 | 49.2 | 47.8 | 0.00 | 0.20 | 0.00 | 0.20 | 0.27 | 0.17 | 0.27 | 0.17 | 500 |
| 7 | 3 | 50 | 250 | 2 | 0.5 | 1.5 | 2 | 2 | 50.8 | 48.8 | 51.4 | 48.6 | 0.03 | 0.18 | 0.03 | 0.18 | 0.25 | 0.17 | 0.25 | 0.16 | 500 |
| 7 | 3 | 50 | 250 | 2 | 0.5 | 2.5 | 0.5 | 1 | 48.6 | 47.0 | 48.6 | 47.0 | 0.01 | 0.18 | 0.00 | 0.19 | 0.28 | 0.18 | 0.27 | 0.18 | 500 |
| 7 | 3 | 50 | 250 | 2 | 0.5 | 2.5 | 0.5 | 2 | 48.0 | 47.2 | 48.6 | 47.2 | 0.00 | 0.19 | 0.01 | 0.20 | 0.27 | 0.18 | 0.27 | 0.18 | 500 |
| 7 | 3 | 50 | 500 | 0 | 0 | 1.5 | 2 | 1 | 6.4 | 6.4 | 6.4 | 6.4 | 0.00 | 0.00 | 0.00 | 0.00 | 0.25 | 0.18 | 0.24 | 0.18 | 500 |
| 7 | 3 | 50 | 500 | 0 | 0 | 1.5 | 2 | 2 | 4.6 | 4.4 | 4.6 | 4.4 | 0.00 | 0.00 | 0.00 | 0.00 | 0.24 | 0.18 | 0.24 | 0.17 | 500 |
| 7 | 3 | 50 | 500 | 0 | 0 | 2.5 | 0.5 | 1 | 5.6 | 5.2 | 5.6 | 5.2 | 0.01 | 0.00 | 0.01 | 0.01 | 0.24 | 0.17 | 0.24 | 0.17 | 500 |
| 7 | 3 | 50 | 500 | 0 | 0 | 2.5 | 0.5 | 2 | 4.8 | 4.8 | 5.0 | 4.8 | 0.00 | 0.00 | 0.00 | 0.00 | 0.24 | 0.17 | 0.24 | 0.17 | 500 |
| 7 | 3 | 50 | 500 | 0 | 0.2 | 1.5 | 2 | 1 | 12.8 | 12.6 | 13.0 | 12.6 | 0.00 | 0.05 | 0.00 | 0.06 | 0.24 | 0.18 | 0.24 | 0.18 | 500 |
| 7 | 3 | 50 | 500 | 0 | 0.2 | 1.5 | 2 | 2 | 15.2 | 15.0 | 15.2 | 15.0 | 0.00 | 0.05 | 0.00 | 0.05 | 0.24 | 0.18 | 0.23 | 0.17 | 500 |
| 7 | 3 | 50 | 500 | 0 | 0.2 | 2.5 | 0.5 | 1 | 12.8 | 11.8 | 12.8 | 12.0 | 0.00 | 0.05 | 0.00 | 0.06 | 0.24 | 0.18 | 0.24 | 0.17 | 500 |
| 7 | 3 | 50 | 500 | 0 | 0.2 | 2.5 | 0.5 | 2 | 14.2 | 13.4 | 14.2 | 13.4 | 0.01 | 0.06 | 0.01 | 0.06 | 0.25 | 0.18 | 0.25 | 0.18 | 500 |
| 7 | 3 | 50 | 500 | 0 | 0.5 | 1.5 | 2 | 1 | 56.6 | 55.4 | 58.0 | 55.4 | 0.00 | 0.13 | 0.00 | 0.13 | 0.25 | 0.19 | 0.24 | 0.19 | 500 |
| 7 | 3 | 50 | 500 | 0 | 0.5 | 1.5 | 2 | 2 | 55.8 | 55.2 | 56.0 | 54.8 | 0.00 | 0.13 | 0.00 | 0.13 | 0.23 | 0.18 | 0.23 | 0.17 | 500 |
| 7 | 3 | 50 | 500 | 0 | 0.5 | 2.5 | 0.5 | 1 | 52.8 | 51.8 | 53.2 | 51.8 | 0.00 | 0.14 | 0.01 | 0.14 | 0.24 | 0.18 | 0.23 | 0.18 | 500 |
| 7 | 3 | 50 | 500 | 0 | 0.5 | 2.5 | 0.5 | 2 | 51.4 | 50.4 | 52.0 | 50.6 | 0.02 | 0.15 | 0.03 | 0.16 | 0.25 | 0.19 | 0.25 | 0.19 | 500 |
| 7 | 3 | 50 | 500 | 0.5 | 0 | 1.5 | 2 | 1 | 4.4 | 4.4 | 4.6 | 4.4 | 0.02 | 0.01 | 0.01 | 0.01 | 0.23 | 0.16 | 0.23 | 0.16 | 500 |
| 7 | 3 | 50 | 500 | 0.5 | 0 | 1.5 | 2 | 2 | 5.4 | 5.2 | 5.4 | 5.4 | 0.02 | 0.01 | 0.02 | 0.01 | 0.25 | 0.18 | 0.24 | 0.18 | 500 |
| 7 | 3 | 50 | 500 | 0.5 | 0 | 2.5 | 0.5 | 1 | 5.6 | 5.2 | 5.8 | 5.4 | 0.01 | 0.01 | 0.01 | 0.01 | 0.24 | 0.17 | 0.24 | 0.17 | 500 |
| 7 | 3 | 50 | 500 | 0.5 | 0 | 2.5 | 0.5 | 2 | 5.0 | 4.6 | 5.2 | 4.6 | 0.01 | 0.01 | 0.01 | 0.01 | 0.24 | 0.17 | 0.23 | 0.17 | 500 |
| 7 | 3 | 50 | 500 | 0.5 | 0.2 | 1.5 | 2 | 1 | 11.4 | 11.0 | 12.0 | 10.8 | 0.01 | 0.06 | 0.01 | 0.06 | 0.23 | 0.17 | 0.23 | 0.17 | 500 |
| 7 | 3 | 50 | 500 | 0.5 | 0.2 | 1.5 | 2 | 2 | 15.6 | 14.8 | 15.8 | 15.2 | 0.02 | 0.04 | 0.02 | 0.04 | 0.24 | 0.18 | 0.24 | 0.17 | 500 |
| 7 | 3 | 50 | 500 | 0.5 | 0.2 | 2.5 | 0.5 | 1 | 16.4 | 16.0 | 16.6 | 16.0 | 0.01 | 0.05 | 0.01 | 0.05 | 0.26 | 0.18 | 0.25 | 0.18 | 500 |
| 7 | 3 | 50 | 500 | 0.5 | 0.2 | 2.5 | 0.5 | 2 | 14.0 | 13.4 | 14.2 | 13.2 | 0.01 | 0.05 | 0.01 | 0.05 | 0.24 | 0.17 | 0.24 | 0.17 | 500 |
| 7 | 3 | 50 | 500 | 0.5 | 0.5 | 1.5 | 2 | 1 | 57.4 | 56.4 | 57.4 | 56.4 | 0.02 | 0.11 | 0.02 | 0.12 | 0.23 | 0.18 | 0.23 | 0.17 | 500 |
| 7 | 3 | 50 | 500 | 0.5 | 0.5 | 1.5 | 2 | 2 | 57.0 | 55.2 | 57.4 | 55.6 | 0.02 | 0.12 | 0.01 | 0.13 | 0.24 | 0.19 | 0.24 | 0.19 | 500 |
| 7 | 3 | 50 | 500 | 0.5 | 0.5 | 2.5 | 0.5 | 1 | 59.6 | 58.8 | 60.0 | 58.8 | 0.02 | 0.12 | 0.02 | 0.13 | 0.24 | 0.19 | 0.24 | 0.18 | 500 |
| 7 | 3 | 50 | 500 | 0.5 | 0.5 | 2.5 | 0.5 | 2 | 59.4 | 57.6 | 59.4 | 58.2 | 0.02 | 0.12 | 0.01 | 0.13 | 0.23 | 0.18 | 0.23 | 0.17 | 500 |
| 7 | 3 | 50 | 500 | 2 | 0 | 1.5 | 2 | 1 | 6.2 | 5.8 | 6.8 | 5.8 | 0.01 | 0.01 | 0.01 | 0.01 | 0.27 | 0.16 | 0.27 | 0.16 | 500 |
| 7 | 3 | 50 | 500 | 2 | 0 | 1.5 | 2 | 2 | 6.4 | 6.0 | 6.4 | 6.0 | 0.02 | 0.01 | 0.02 | 0.01 | 0.27 | 0.16 | 0.27 | 0.16 | 500 |
| 7 | 3 | 50 | 500 | 2 | 0 | 2.5 | 0.5 | 1 | 6.4 | 6.4 | 6.6 | 6.4 | 0.00 | 0.00 | 0.00 | 0.00 | 0.27 | 0.16 | 0.27 | 0.16 | 500 |
| 7 | 3 | 50 | 500 | 2 | 0 | 2.5 | 0.5 | 2 | 6.2 | 6.0 | 6.4 | 6.0 | 0.01 | 0.00 | 0.01 | 0.00 | 0.27 | 0.16 | 0.27 | 0.16 | 500 |
| 7 | 3 | 50 | 500 | 2 | 0.2 | 1.5 | 2 | 1 | 10.6 | 10.4 | 11.0 | 10.2 | 0.00 | 0.08 | 0.00 | 0.08 | 0.26 | 0.16 | 0.26 | 0.16 | 500 |
| 7 | 3 | 50 | 500 | 2 | 0.2 | 1.5 | 2 | 2 | 11.6 | 11.0 | 12.2 | 10.6 | 0.01 | 0.07 | 0.01 | 0.07 | 0.27 | 0.16 | 0.26 | 0.16 | 500 |
| 7 | 3 | 50 | 500 | 2 | 0.2 | 2.5 | 0.5 | 1 | 14.2 | 14.0 | 14.4 | 14.0 | 0.01 | 0.08 | 0.00 | 0.08 | 0.27 | 0.17 | 0.27 | 0.17 | 500 |
| 7 | 3 | 50 | 500 | 2 | 0.2 | 2.5 | 0.5 | 2 | 12.6 | 12.4 | 13.0 | 12.4 | 0.01 | 0.08 | 0.01 | 0.08 | 0.26 | 0.16 | 0.25 | 0.16 | 500 |
| 7 | 3 | 50 | 500 | 2 | 0.5 | 1.5 | 2 | 1 | 45.6 | 45.0 | 46.4 | 44.8 | 0.01 | 0.21 | 0.02 | 0.21 | 0.26 | 0.17 | 0.26 | 0.17 | 500 |
| 7 | 3 | 50 | 500 | 2 | 0.5 | 1.5 | 2 | 2 | 46.8 | 45.6 | 46.8 | 45.6 | 0.00 | 0.20 | 0.00 | 0.20 | 0.26 | 0.17 | 0.26 | 0.16 | 500 |
| 7 | 3 | 50 | 500 | 2 | 0.5 | 2.5 | 0.5 | 1 | 47.2 | 45.8 | 47.6 | 46.2 | 0.01 | 0.20 | 0.01 | 0.20 | 0.27 | 0.18 | 0.27 | 0.17 | 500 |
| 7 | 3 | 50 | 500 | 2 | 0.5 | 2.5 | 0.5 | 2 | 46.0 | 44.8 | 46.2 | 44.4 | 0.01 | 0.20 | 0.02 | 0.21 | 0.26 | 0.17 | 0.26 | 0.16 | 500 |
| 7 | 3 | 100 | 100 | 0 | 0 | 1.5 | 2 | 1 | 7.2 | 7.2 | 7.2 | 7.2 | 0.01 | 0.01 | 0.01 | 0.01 | 0.17 | 0.13 | 0.17 | 0.13 | 500 |
| 7 | 3 | 100 | 100 | 0 | 0 | 1.5 | 2 | 2 | 8.0 | 7.8 | 8.0 | 7.8 | 0.01 | 0.01 | 0.01 | 0.01 | 0.17 | 0.13 | 0.17 | 0.13 | 500 |
| 7 | 3 | 100 | 100 | 0 | 0 | 2.5 | 0.5 | 1 | 4.8 | 4.8 | 4.8 | 4.8 | 0.01 | 0.01 | 0.01 | 0.01 | 0.18 | 0.13 | 0.18 | 0.13 | 500 |
| 7 | 3 | 100 | 100 | 0 | 0 | 2.5 | 0.5 | 2 | 5.6 | 5.4 | 5.6 | 5.4 | 0.00 | 0.00 | 0.00 | 0.00 | 0.17 | 0.12 | 0.17 | 0.12 | 500 |
| 7 | 3 | 100 | 100 | 0 | 0.2 | 1.5 | 2 | 1 | 28.2 | 27.6 | 28.0 | 27.8 | 0.01 | 0.05 | 0.01 | 0.05 | 0.18 | 0.13 | 0.18 | 0.14 | 500 |
| 7 | 3 | 100 | 100 | 0 | 0.2 | 1.5 | 2 | 2 | 24.6 | 24.4 | 24.6 | 24.4 | 0.01 | 0.05 | 0.01 | 0.05 | 0.17 | 0.13 | 0.17 | 0.13 | 500 |
| 7 | 3 | 100 | 100 | 0 | 0.2 | 2.5 | 0.5 | 1 | 24.2 | 23.4 | 24.4 | 23.4 | 0.01 | 0.05 | 0.01 | 0.05 | 0.17 | 0.12 | 0.17 | 0.13 | 500 |
| 7 | 3 | 100 | 100 | 0 | 0.2 | 2.5 | 0.5 | 2 | 25.0 | 24.8 | 25.2 | 24.8 | 0.00 | 0.05 | 0.01 | 0.05 | 0.17 | 0.12 | 0.17 | 0.13 | 500 |
| 7 | 3 | 100 | 100 | 0 | 0.5 | 1.5 | 2 | 1 | 84.0 | 83.4 | 83.8 | 83.2 | 0.00 | 0.13 | 0.01 | 0.12 | 0.17 | 0.13 | 0.17 | 0.13 | 500 |
| 7 | 3 | 100 | 100 | 0 | 0.5 | 1.5 | 2 | 2 | 85.8 | 85.8 | 85.8 | 85.8 | 0.02 | 0.12 | 0.02 | 0.11 | 0.17 | 0.13 | 0.17 | 0.14 | 500 |
| 7 | 3 | 100 | 100 | 0 | 0.5 | 2.5 | 0.5 | 1 | 83.0 | 82.8 | 83.0 | 82.8 | 0.00 | 0.13 | 0.01 | 0.13 | 0.17 | 0.13 | 0.18 | 0.14 | 500 |
| 7 | 3 | 100 | 100 | 0 | 0.5 | 2.5 | 0.5 | 2 | 84.0 | 83.8 | 84.2 | 83.8 | 0.00 | 0.13 | 0.01 | 0.13 | 0.16 | 0.13 | 0.17 | 0.13 | 500 |
| 7 | 3 | 100 | 100 | 0.5 | 0 | 1.5 | 2 | 1 | 5.6 | 5.6 | 5.8 | 5.6 | 0.00 | 0.00 | 0.00 | 0.00 | 0.17 | 0.12 | 0.17 | 0.13 | 500 |
| 7 | 3 | 100 | 100 | 0.5 | 0 | 1.5 | 2 | 2 | 5.6 | 5.6 | 5.8 | 5.6 | 0.00 | 0.00 | 0.00 | 0.00 | 0.17 | 0.12 | 0.17 | 0.13 | 500 |
| 7 | 3 | 100 | 100 | 0.5 | 0 | 2.5 | 0.5 | 1 | 6.4 | 6.4 | 6.4 | 6.4 | 0.00 | 0.00 | 0.00 | 0.00 | 0.17 | 0.12 | 0.17 | 0.12 | 500 |
| 7 | 3 | 100 | 100 | 0.5 | 0 | 2.5 | 0.5 | 2 | 8.2 | 8.2 | 8.4 | 8.2 | 0.01 | 0.01 | 0.01 | 0.00 | 0.18 | 0.13 | 0.18 | 0.13 | 500 |
| 7 | 3 | 100 | 100 | 0.5 | 0.2 | 1.5 | 2 | 1 | 22.6 | 22.2 | 22.8 | 21.8 | 0.00 | 0.06 | 0.00 | 0.05 | 0.17 | 0.12 | 0.17 | 0.13 | 500 |
| 7 | 3 | 100 | 100 | 0.5 | 0.2 | 1.5 | 2 | 2 | 24.6 | 24.6 | 24.8 | 24.6 | 0.01 | 0.05 | 0.01 | 0.05 | 0.17 | 0.12 | 0.17 | 0.12 | 500 |
| 7 | 3 | 100 | 100 | 0.5 | 0.2 | 2.5 | 0.5 | 1 | 23.6 | 23.6 | 23.6 | 23.4 | 0.00 | 0.06 | 0.00 | 0.05 | 0.17 | 0.12 | 0.17 | 0.12 | 500 |
| 7 | 3 | 100 | 100 | 0.5 | 0.2 | 2.5 | 0.5 | 2 | 22.2 | 22.0 | 22.8 | 22.2 | 0.00 | 0.06 | 0.00 | 0.06 | 0.17 | 0.12 | 0.17 | 0.12 | 500 |
| 7 | 3 | 100 | 100 | 0.5 | 0.5 | 1.5 | 2 | 1 | 85.0 | 85.0 | 85.2 | 84.8 | 0.01 | 0.13 | 0.01 | 0.12 | 0.17 | 0.13 | 0.17 | 0.13 | 500 |
| 7 | 3 | 100 | 100 | 0.5 | 0.5 | 1.5 | 2 | 2 | 87.8 | 87.4 | 88.0 | 87.6 | 0.02 | 0.12 | 0.02 | 0.12 | 0.17 | 0.13 | 0.17 | 0.13 | 500 |
| 7 | 3 | 100 | 100 | 0.5 | 0.5 | 2.5 | 0.5 | 1 | 86.4 | 86.2 | 86.4 | 86.0 | 0.02 | 0.13 | 0.02 | 0.12 | 0.17 | 0.13 | 0.17 | 0.13 | 500 |
| 7 | 3 | 100 | 100 | 0.5 | 0.5 | 2.5 | 0.5 | 2 | 84.8 | 84.2 | 84.8 | 84.2 | 0.00 | 0.14 | 0.00 | 0.14 | 0.17 | 0.13 | 0.17 | 0.13 | 500 |
| 7 | 3 | 100 | 100 | 2 | 0 | 1.5 | 2 | 1 | 4.4 | 4.4 | 4.4 | 4.4 | 0.00 | 0.00 | 0.00 | 0.00 | 0.18 | 0.11 | 0.18 | 0.11 | 500 |
| 7 | 3 | 100 | 100 | 2 | 0 | 1.5 | 2 | 2 | 4.4 | 4.4 | 4.4 | 4.4 | 0.00 | 0.00 | 0.00 | 0.00 | 0.18 | 0.11 | 0.18 | 0.11 | 500 |
| 7 | 3 | 100 | 100 | 2 | 0 | 2.5 | 0.5 | 1 | 6.6 | 6.6 | 6.6 | 6.6 | 0.00 | 0.00 | 0.00 | 0.00 | 0.19 | 0.11 | 0.19 | 0.11 | 500 |
| 7 | 3 | 100 | 100 | 2 | 0 | 2.5 | 0.5 | 2 | 5.4 | 5.4 | 5.4 | 5.2 | 0.01 | 0.00 | 0.01 | 0.00 | 0.19 | 0.11 | 0.19 | 0.11 | 500 |
| 7 | 3 | 100 | 100 | 2 | 0.2 | 1.5 | 2 | 1 | 19.0 | 18.6 | 19.2 | 18.8 | 0.00 | 0.08 | 0.00 | 0.08 | 0.19 | 0.11 | 0.19 | 0.11 | 500 |
| 7 | 3 | 100 | 100 | 2 | 0.2 | 1.5 | 2 | 2 | 22.0 | 21.6 | 22.0 | 21.8 | 0.02 | 0.07 | 0.02 | 0.07 | 0.19 | 0.11 | 0.19 | 0.11 | 500 |
| 7 | 3 | 100 | 100 | 2 | 0.2 | 2.5 | 0.5 | 1 | 16.2 | 16.2 | 16.2 | 16.2 | 0.02 | 0.09 | 0.02 | 0.09 | 0.18 | 0.11 | 0.18 | 0.11 | 500 |
| 7 | 3 | 100 | 100 | 2 | 0.2 | 2.5 | 0.5 | 2 | 20.2 | 19.8 | 20.0 | 19.6 | 0.00 | 0.08 | 0.01 | 0.08 | 0.19 | 0.11 | 0.19 | 0.12 | 500 |
| 7 | 3 | 100 | 100 | 2 | 0.5 | 1.5 | 2 | 1 | 77.6 | 77.4 | 77.8 | 77.2 | 0.00 | 0.20 | 0.01 | 0.19 | 0.18 | 0.12 | 0.18 | 0.12 | 500 |
| 7 | 3 | 100 | 100 | 2 | 0.5 | 1.5 | 2 | 2 | 75.4 | 75.4 | 75.6 | 75.0 | 0.00 | 0.20 | 0.00 | 0.20 | 0.19 | 0.12 | 0.19 | 0.12 | 500 |
| 7 | 3 | 100 | 100 | 2 | 0.5 | 2.5 | 0.5 | 1 | 75.2 | 75.2 | 75.4 | 75.2 | 0.01 | 0.20 | 0.01 | 0.20 | 0.19 | 0.12 | 0.19 | 0.12 | 500 |
| 7 | 3 | 100 | 100 | 2 | 0.5 | 2.5 | 0.5 | 2 | 77.4 | 77.0 | 77.4 | 77.2 | 0.00 | 0.19 | 0.00 | 0.19 | 0.19 | 0.12 | 0.19 | 0.12 | 500 |
| 7 | 3 | 100 | 250 | 0 | 0 | 1.5 | 2 | 1 | 4.6 | 4.2 | 4.8 | 4.0 | 0.01 | 0.00 | 0.01 | 0.00 | 0.16 | 0.12 | 0.17 | 0.12 | 500 |
| 7 | 3 | 100 | 250 | 0 | 0 | 1.5 | 2 | 2 | 4.6 | 4.2 | 4.8 | 4.0 | 0.01 | 0.00 | 0.01 | 0.00 | 0.16 | 0.12 | 0.17 | 0.12 | 500 |
| 7 | 3 | 100 | 250 | 0 | 0 | 2.5 | 0.5 | 1 | 5.8 | 5.6 | 6.0 | 5.8 | 0.00 | 0.00 | 0.00 | 0.00 | 0.17 | 0.12 | 0.17 | 0.12 | 500 |
| 7 | 3 | 100 | 250 | 0 | 0 | 2.5 | 0.5 | 2 | 5.8 | 5.6 | 5.8 | 5.6 | 0.02 | 0.01 | 0.02 | 0.01 | 0.17 | 0.12 | 0.17 | 0.12 | 500 |
| 7 | 3 | 100 | 250 | 0 | 0.2 | 1.5 | 2 | 1 | 23.4 | 22.6 | 23.6 | 22.8 | 0.00 | 0.05 | 0.00 | 0.05 | 0.17 | 0.13 | 0.17 | 0.13 | 500 |
| 7 | 3 | 100 | 250 | 0 | 0.2 | 1.5 | 2 | 2 | 22.6 | 22.6 | 22.6 | 22.6 | 0.00 | 0.05 | 0.00 | 0.05 | 0.17 | 0.13 | 0.17 | 0.13 | 500 |
| 7 | 3 | 100 | 250 | 0 | 0.2 | 2.5 | 0.5 | 1 | 20.0 | 19.6 | 20.4 | 19.6 | 0.01 | 0.06 | 0.01 | 0.06 | 0.17 | 0.12 | 0.17 | 0.12 | 500 |
| 7 | 3 | 100 | 250 | 0 | 0.2 | 2.5 | 0.5 | 2 | 24.2 | 23.4 | 24.2 | 23.6 | 0.01 | 0.05 | 0.01 | 0.05 | 0.16 | 0.12 | 0.16 | 0.12 | 500 |
| 7 | 3 | 100 | 250 | 0 | 0.5 | 1.5 | 2 | 1 | 86.0 | 85.4 | 86.2 | 85.4 | 0.01 | 0.12 | 0.01 | 0.12 | 0.17 | 0.13 | 0.17 | 0.13 | 500 |
| 7 | 3 | 100 | 250 | 0 | 0.5 | 1.5 | 2 | 2 | 82.6 | 82.4 | 83.0 | 82.6 | 0.01 | 0.14 | 0.01 | 0.14 | 0.17 | 0.14 | 0.17 | 0.13 | 500 |
| 7 | 3 | 100 | 250 | 0 | 0.5 | 2.5 | 0.5 | 1 | 84.2 | 83.6 | 84.2 | 83.4 | 0.00 | 0.13 | 0.00 | 0.14 | 0.17 | 0.13 | 0.17 | 0.13 | 500 |
| 7 | 3 | 100 | 250 | 0 | 0.5 | 2.5 | 0.5 | 2 | 83.8 | 83.0 | 83.8 | 83.0 | 0.00 | 0.13 | 0.00 | 0.13 | 0.18 | 0.13 | 0.18 | 0.13 | 500 |
| 7 | 3 | 100 | 250 | 0.5 | 0 | 1.5 | 2 | 1 | 5.4 | 5.4 | 5.6 | 5.4 | 0.00 | 0.00 | 0.00 | 0.00 | 0.18 | 0.13 | 0.18 | 0.13 | 500 |
| 7 | 3 | 100 | 250 | 0.5 | 0 | 1.5 | 2 | 2 | 5.4 | 5.4 | 5.6 | 5.4 | 0.00 | 0.00 | 0.00 | 0.00 | 0.18 | 0.13 | 0.18 | 0.13 | 500 |
| 7 | 3 | 100 | 250 | 0.5 | 0 | 2.5 | 0.5 | 1 | 5.6 | 5.4 | 5.6 | 5.4 | 0.00 | 0.00 | 0.00 | 0.00 | 0.17 | 0.12 | 0.18 | 0.12 | 500 |
| 7 | 3 | 100 | 250 | 0.5 | 0 | 2.5 | 0.5 | 2 | 3.0 | 3.0 | 3.0 | 3.0 | 0.01 | 0.01 | 0.01 | 0.01 | 0.17 | 0.12 | 0.17 | 0.12 | 500 |
| 7 | 3 | 100 | 250 | 0.5 | 0.2 | 1.5 | 2 | 1 | 20.8 | 20.6 | 21.4 | 20.6 | 0.00 | 0.05 | 0.00 | 0.05 | 0.16 | 0.12 | 0.16 | 0.12 | 500 |
| 7 | 3 | 100 | 250 | 0.5 | 0.2 | 1.5 | 2 | 2 | 24.0 | 23.0 | 24.2 | 23.0 | 0.00 | 0.05 | 0.00 | 0.05 | 0.17 | 0.12 | 0.17 | 0.12 | 500 |
| 7 | 3 | 100 | 250 | 0.5 | 0.2 | 2.5 | 0.5 | 1 | 20.4 | 20.0 | 20.4 | 20.2 | 0.00 | 0.06 | 0.00 | 0.06 | 0.17 | 0.12 | 0.16 | 0.12 | 500 |
| 7 | 3 | 100 | 250 | 0.5 | 0.2 | 2.5 | 0.5 | 2 | 25.8 | 25.2 | 25.8 | 25.2 | 0.02 | 0.05 | 0.02 | 0.05 | 0.17 | 0.12 | 0.17 | 0.12 | 500 |
| 7 | 3 | 100 | 250 | 0.5 | 0.5 | 1.5 | 2 | 1 | 85.2 | 85.0 | 85.2 | 84.8 | 0.01 | 0.13 | 0.00 | 0.13 | 0.17 | 0.13 | 0.17 | 0.13 | 500 |
| 7 | 3 | 100 | 250 | 0.5 | 0.5 | 1.5 | 2 | 2 | 86.0 | 86.0 | 86.0 | 85.8 | 0.01 | 0.13 | 0.01 | 0.13 | 0.16 | 0.13 | 0.17 | 0.13 | 500 |
| 7 | 3 | 100 | 250 | 0.5 | 0.5 | 2.5 | 0.5 | 1 | 84.2 | 84.2 | 84.4 | 84.2 | 0.00 | 0.14 | 0.00 | 0.14 | 0.16 | 0.12 | 0.16 | 0.12 | 500 |
| 7 | 3 | 100 | 250 | 0.5 | 0.5 | 2.5 | 0.5 | 2 | 86.6 | 86.4 | 86.6 | 86.4 | 0.01 | 0.13 | 0.01 | 0.13 | 0.17 | 0.13 | 0.17 | 0.13 | 500 |
| 7 | 3 | 100 | 250 | 2 | 0 | 1.5 | 2 | 1 | 5.4 | 5.2 | 5.4 | 5.2 | 0.01 | 0.00 | 0.01 | 0.00 | 0.19 | 0.11 | 0.19 | 0.11 | 500 |
| 7 | 3 | 100 | 250 | 2 | 0 | 1.5 | 2 | 2 | 5.4 | 5.2 | 5.4 | 5.2 | 0.01 | 0.00 | 0.01 | 0.00 | 0.19 | 0.11 | 0.19 | 0.11 | 500 |
| 7 | 3 | 100 | 250 | 2 | 0 | 2.5 | 0.5 | 1 | 4.0 | 4.0 | 4.0 | 4.0 | 0.00 | 0.00 | 0.00 | 0.00 | 0.18 | 0.11 | 0.18 | 0.11 | 500 |
| 7 | 3 | 100 | 250 | 2 | 0 | 2.5 | 0.5 | 2 | 5.6 | 5.2 | 5.4 | 5.2 | 0.01 | 0.01 | 0.01 | 0.00 | 0.18 | 0.11 | 0.18 | 0.11 | 500 |
| 7 | 3 | 100 | 250 | 2 | 0.2 | 1.5 | 2 | 1 | 20.2 | 19.8 | 20.2 | 19.6 | 0.00 | 0.08 | 0.00 | 0.08 | 0.19 | 0.11 | 0.19 | 0.11 | 500 |
| 7 | 3 | 100 | 250 | 2 | 0.2 | 1.5 | 2 | 2 | 19.4 | 19.2 | 19.6 | 19.2 | 0.00 | 0.08 | 0.00 | 0.08 | 0.19 | 0.11 | 0.19 | 0.11 | 500 |
| 7 | 3 | 100 | 250 | 2 | 0.2 | 2.5 | 0.5 | 1 | 17.8 | 17.0 | 17.8 | 17.0 | 0.02 | 0.09 | 0.02 | 0.09 | 0.19 | 0.12 | 0.19 | 0.11 | 500 |
| 7 | 3 | 100 | 250 | 2 | 0.2 | 2.5 | 0.5 | 2 | 20.6 | 19.6 | 20.6 | 19.8 | 0.01 | 0.07 | 0.01 | 0.07 | 0.18 | 0.11 | 0.18 | 0.11 | 500 |
| 7 | 3 | 100 | 250 | 2 | 0.5 | 1.5 | 2 | 1 | 77.8 | 77.8 | 78.0 | 78.0 | 0.01 | 0.19 | 0.01 | 0.19 | 0.19 | 0.12 | 0.18 | 0.12 | 500 |
| 7 | 3 | 100 | 250 | 2 | 0.5 | 1.5 | 2 | 2 | 80.0 | 79.4 | 80.0 | 79.4 | 0.02 | 0.19 | 0.02 | 0.19 | 0.18 | 0.12 | 0.18 | 0.12 | 500 |
| 7 | 3 | 100 | 250 | 2 | 0.5 | 2.5 | 0.5 | 1 | 77.2 | 77.2 | 77.2 | 77.2 | 0.00 | 0.19 | 0.00 | 0.19 | 0.18 | 0.12 | 0.18 | 0.12 | 500 |
| 7 | 3 | 100 | 250 | 2 | 0.5 | 2.5 | 0.5 | 2 | 81.6 | 81.0 | 81.6 | 81.0 | 0.01 | 0.19 | 0.01 | 0.19 | 0.18 | 0.12 | 0.18 | 0.12 | 500 |
| 7 | 3 | 100 | 500 | 0 | 0 | 1.5 | 2 | 1 | 6.0 | 6.0 | 6.0 | 6.0 | 0.00 | 0.00 | 0.00 | 0.00 | 0.17 | 0.13 | 0.17 | 0.12 | 500 |
| 7 | 3 | 100 | 500 | 0 | 0 | 1.5 | 2 | 2 | 5.4 | 5.2 | 5.4 | 5.2 | 0.01 | 0.00 | 0.01 | 0.00 | 0.17 | 0.12 | 0.17 | 0.12 | 500 |
| 7 | 3 | 100 | 500 | 0 | 0 | 2.5 | 0.5 | 1 | 6.0 | 6.0 | 6.0 | 6.0 | 0.00 | 0.00 | 0.00 | 0.00 | 0.17 | 0.12 | 0.17 | 0.12 | 500 |
| 7 | 3 | 100 | 500 | 0 | 0 | 2.5 | 0.5 | 2 | 5.2 | 5.2 | 5.2 | 5.2 | 0.01 | 0.01 | 0.01 | 0.01 | 0.16 | 0.12 | 0.16 | 0.12 | 500 |
| 7 | 3 | 100 | 500 | 0 | 0.2 | 1.5 | 2 | 1 | 19.6 | 19.6 | 19.6 | 19.6 | 0.01 | 0.06 | 0.01 | 0.06 | 0.17 | 0.12 | 0.17 | 0.12 | 500 |
| 7 | 3 | 100 | 500 | 0 | 0.2 | 1.5 | 2 | 2 | 26.0 | 26.0 | 26.0 | 26.0 | 0.01 | 0.04 | 0.01 | 0.04 | 0.17 | 0.12 | 0.16 | 0.12 | 500 |
| 7 | 3 | 100 | 500 | 0 | 0.2 | 2.5 | 0.5 | 1 | 21.2 | 21.2 | 21.2 | 21.2 | 0.00 | 0.05 | 0.00 | 0.05 | 0.17 | 0.12 | 0.17 | 0.12 | 500 |
| 7 | 3 | 100 | 500 | 0 | 0.2 | 2.5 | 0.5 | 2 | 21.4 | 21.4 | 21.4 | 21.4 | 0.00 | 0.06 | 0.00 | 0.06 | 0.17 | 0.12 | 0.17 | 0.12 | 500 |
| 7 | 3 | 100 | 500 | 0 | 0.5 | 1.5 | 2 | 1 | 84.2 | 83.6 | 84.0 | 83.4 | 0.00 | 0.13 | 0.00 | 0.13 | 0.17 | 0.13 | 0.17 | 0.13 | 500 |
| 7 | 3 | 100 | 500 | 0 | 0.5 | 1.5 | 2 | 2 | 84.6 | 84.2 | 84.6 | 84.4 | 0.00 | 0.13 | 0.00 | 0.13 | 0.17 | 0.13 | 0.17 | 0.13 | 500 |
| 7 | 3 | 100 | 500 | 0 | 0.5 | 2.5 | 0.5 | 1 | 87.8 | 87.6 | 88.0 | 87.6 | 0.02 | 0.12 | 0.02 | 0.12 | 0.17 | 0.13 | 0.16 | 0.13 | 500 |
| 7 | 3 | 100 | 500 | 0 | 0.5 | 2.5 | 0.5 | 2 | 83.8 | 83.4 | 83.8 | 83.4 | 0.01 | 0.14 | 0.01 | 0.14 | 0.17 | 0.13 | 0.17 | 0.13 | 500 |
| 7 | 3 | 100 | 500 | 0.5 | 0 | 1.5 | 2 | 1 | 6.0 | 5.8 | 6.0 | 5.8 | 0.01 | 0.01 | 0.01 | 0.01 | 0.17 | 0.12 | 0.17 | 0.12 | 500 |
| 7 | 3 | 100 | 500 | 0.5 | 0 | 1.5 | 2 | 2 | 5.6 | 5.4 | 5.8 | 5.4 | 0.01 | 0.01 | 0.01 | 0.01 | 0.17 | 0.12 | 0.17 | 0.12 | 500 |
| 7 | 3 | 100 | 500 | 0.5 | 0 | 2.5 | 0.5 | 1 | 6.2 | 6.0 | 6.2 | 6.0 | 0.01 | 0.01 | 0.01 | 0.01 | 0.17 | 0.12 | 0.17 | 0.12 | 500 |
| 7 | 3 | 100 | 500 | 0.5 | 0 | 2.5 | 0.5 | 2 | 2.6 | 2.2 | 2.8 | 2.4 | 0.00 | 0.00 | 0.00 | 0.00 | 0.15 | 0.11 | 0.15 | 0.11 | 500 |
| 7 | 3 | 100 | 500 | 0.5 | 0.2 | 1.5 | 2 | 1 | 21.0 | 20.8 | 21.0 | 20.8 | 0.01 | 0.06 | 0.01 | 0.06 | 0.17 | 0.12 | 0.17 | 0.12 | 500 |
| 7 | 3 | 100 | 500 | 0.5 | 0.2 | 1.5 | 2 | 2 | 18.8 | 18.6 | 18.8 | 18.6 | 0.01 | 0.06 | 0.01 | 0.06 | 0.15 | 0.11 | 0.15 | 0.11 | 500 |
| 7 | 3 | 100 | 500 | 0.5 | 0.2 | 2.5 | 0.5 | 1 | 19.6 | 19.2 | 19.6 | 19.2 | 0.01 | 0.06 | 0.01 | 0.06 | 0.17 | 0.12 | 0.17 | 0.12 | 500 |
| 7 | 3 | 100 | 500 | 0.5 | 0.2 | 2.5 | 0.5 | 2 | 22.6 | 22.4 | 22.8 | 22.4 | 0.00 | 0.06 | 0.00 | 0.06 | 0.17 | 0.12 | 0.17 | 0.12 | 500 |
| 7 | 3 | 100 | 500 | 0.5 | 0.5 | 1.5 | 2 | 1 | 84.8 | 84.8 | 85.0 | 84.6 | 0.00 | 0.13 | 0.00 | 0.13 | 0.17 | 0.13 | 0.17 | 0.13 | 500 |
| 7 | 3 | 100 | 500 | 0.5 | 0.5 | 1.5 | 2 | 2 | 87.0 | 86.8 | 87.0 | 86.8 | 0.01 | 0.13 | 0.01 | 0.13 | 0.17 | 0.13 | 0.17 | 0.13 | 500 |
| 7 | 3 | 100 | 500 | 0.5 | 0.5 | 2.5 | 0.5 | 1 | 82.6 | 82.4 | 82.6 | 82.4 | 0.00 | 0.14 | 0.00 | 0.14 | 0.18 | 0.13 | 0.17 | 0.13 | 500 |
| 7 | 3 | 100 | 500 | 0.5 | 0.5 | 2.5 | 0.5 | 2 | 83.0 | 83.0 | 83.4 | 83.0 | 0.00 | 0.14 | 0.00 | 0.14 | 0.17 | 0.13 | 0.17 | 0.13 | 500 |
| 7 | 3 | 100 | 500 | 2 | 0 | 1.5 | 2 | 1 | 5.0 | 5.0 | 5.0 | 5.0 | 0.00 | 0.00 | 0.00 | 0.00 | 0.18 | 0.11 | 0.18 | 0.11 | 500 |
| 7 | 3 | 100 | 500 | 2 | 0 | 1.5 | 2 | 2 | 2.6 | 2.4 | 2.8 | 2.4 | 0.01 | 0.01 | 0.01 | 0.01 | 0.17 | 0.10 | 0.17 | 0.10 | 500 |
| 7 | 3 | 100 | 500 | 2 | 0 | 2.5 | 0.5 | 1 | 6.6 | 6.4 | 6.6 | 6.4 | 0.01 | 0.01 | 0.01 | 0.01 | 0.19 | 0.11 | 0.19 | 0.11 | 500 |
| 7 | 3 | 100 | 500 | 2 | 0 | 2.5 | 0.5 | 2 | 6.0 | 5.6 | 6.0 | 5.6 | 0.00 | 0.00 | 0.00 | 0.00 | 0.18 | 0.11 | 0.18 | 0.11 | 500 |
| 7 | 3 | 100 | 500 | 2 | 0.2 | 1.5 | 2 | 1 | 18.0 | 17.6 | 18.0 | 17.6 | 0.00 | 0.08 | 0.00 | 0.08 | 0.18 | 0.11 | 0.18 | 0.11 | 500 |
| 7 | 3 | 100 | 500 | 2 | 0.2 | 1.5 | 2 | 2 | 12.0 | 11.8 | 12.2 | 12.0 | 0.03 | 0.10 | 0.03 | 0.10 | 0.18 | 0.11 | 0.18 | 0.11 | 500 |
| 7 | 3 | 100 | 500 | 2 | 0.2 | 2.5 | 0.5 | 1 | 19.8 | 19.4 | 19.8 | 19.4 | 0.00 | 0.08 | 0.00 | 0.08 | 0.18 | 0.11 | 0.18 | 0.11 | 500 |
| 7 | 3 | 100 | 500 | 2 | 0.2 | 2.5 | 0.5 | 2 | 16.4 | 16.2 | 16.6 | 16.2 | 0.00 | 0.08 | 0.00 | 0.08 | 0.18 | 0.11 | 0.18 | 0.11 | 500 |
| 7 | 3 | 100 | 500 | 2 | 0.5 | 1.5 | 2 | 1 | 77.8 | 77.6 | 78.0 | 77.6 | 0.01 | 0.19 | 0.01 | 0.19 | 0.19 | 0.13 | 0.19 | 0.13 | 500 |
| 7 | 3 | 100 | 500 | 2 | 0.5 | 1.5 | 2 | 2 | 78.0 | 77.2 | 78.4 | 77.2 | 0.01 | 0.19 | 0.00 | 0.20 | 0.19 | 0.12 | 0.19 | 0.12 | 500 |
| 7 | 3 | 100 | 500 | 2 | 0.5 | 2.5 | 0.5 | 1 | 76.2 | 75.6 | 76.2 | 75.6 | 0.00 | 0.19 | 0.00 | 0.19 | 0.19 | 0.12 | 0.18 | 0.12 | 500 |
| 7 | 3 | 100 | 500 | 2 | 0.5 | 2.5 | 0.5 | 2 | 76.4 | 75.4 | 76.4 | 75.4 | 0.00 | 0.19 | 0.00 | 0.20 | 0.19 | 0.12 | 0.19 | 0.12 | 500 |
| 7 | 3 | 200 | 100 | 0 | 0 | 1.5 | 2 | 1 | 5.2 | 5.0 | 5.4 | 5.2 | 0.00 | 0.00 | 0.00 | 0.00 | 0.12 | 0.09 | 0.12 | 0.09 | 500 |
| 7 | 3 | 200 | 100 | 0 | 0 | 1.5 | 2 | 2 | 5.2 | 5.0 | 5.4 | 5.2 | 0.00 | 0.00 | 0.00 | 0.00 | 0.12 | 0.09 | 0.12 | 0.09 | 500 |
| 7 | 3 | 200 | 100 | 0 | 0 | 2.5 | 0.5 | 1 | 5.0 | 4.8 | 5.0 | 4.8 | 0.00 | 0.00 | 0.00 | 0.00 | 0.12 | 0.09 | 0.12 | 0.09 | 500 |
| 7 | 3 | 200 | 100 | 0 | 0 | 2.5 | 0.5 | 2 | 3.4 | 3.4 | 3.4 | 3.4 | 0.00 | 0.00 | 0.00 | 0.00 | 0.12 | 0.08 | 0.12 | 0.08 | 500 |
| 7 | 3 | 200 | 100 | 0 | 0.2 | 1.5 | 2 | 1 | 39.6 | 39.2 | 39.4 | 39.2 | 0.00 | 0.05 | 0.00 | 0.05 | 0.12 | 0.08 | 0.12 | 0.09 | 500 |
| 7 | 3 | 200 | 100 | 0 | 0.2 | 1.5 | 2 | 2 | 42.6 | 42.2 | 42.6 | 42.4 | 0.01 | 0.05 | 0.01 | 0.04 | 0.13 | 0.09 | 0.13 | 0.10 | 500 |
| 7 | 3 | 200 | 100 | 0 | 0.2 | 2.5 | 0.5 | 1 | 39.8 | 39.0 | 39.8 | 39.2 | 0.00 | 0.06 | 0.00 | 0.06 | 0.12 | 0.09 | 0.12 | 0.09 | 500 |
| 7 | 3 | 200 | 100 | 0 | 0.2 | 2.5 | 0.5 | 2 | 42.0 | 41.8 | 41.8 | 41.8 | 0.00 | 0.05 | 0.01 | 0.05 | 0.12 | 0.09 | 0.12 | 0.09 | 500 |
| 7 | 3 | 200 | 100 | 0 | 0.5 | 1.5 | 2 | 1 | 99.2 | 99.0 | 99.2 | 99.2 | 0.00 | 0.13 | 0.01 | 0.12 | 0.12 | 0.09 | 0.12 | 0.09 | 500 |
| 7 | 3 | 200 | 100 | 0 | 0.5 | 1.5 | 2 | 2 | 98.8 | 98.8 | 98.8 | 98.6 | 0.01 | 0.12 | 0.02 | 0.12 | 0.12 | 0.10 | 0.13 | 0.10 | 500 |
| 7 | 3 | 200 | 100 | 0 | 0.5 | 2.5 | 0.5 | 1 | 98.6 | 98.6 | 98.8 | 98.6 | 0.00 | 0.13 | 0.01 | 0.13 | 0.12 | 0.09 | 0.12 | 0.09 | 500 |
| 7 | 3 | 200 | 100 | 0 | 0.5 | 2.5 | 0.5 | 2 | 99.6 | 99.4 | 99.6 | 99.4 | 0.00 | 0.14 | 0.00 | 0.13 | 0.12 | 0.10 | 0.13 | 0.10 | 500 |
| 7 | 3 | 200 | 100 | 0.5 | 0 | 1.5 | 1 | 1 | 5.8 | 5.8 | 5.8 | 5.8 | 0.00 | 0.00 | 0.00 | 0.00 | 0.12 | 0.09 | 0.12 | 0.09 | 500 |
| 7 | 3 | 200 | 100 | 0.5 | 0 | 1.5 | 2 | 1 | 5.8 | 5.8 | 5.8 | 5.8 | 0.00 | 0.00 | 0.00 | 0.00 | 0.12 | 0.09 | 0.12 | 0.09 | 500 |
| 7 | 3 | 200 | 100 | 0.5 | 0 | 2.5 | 0.5 | 1 | 6.2 | 5.8 | 6.2 | 5.8 | 0.01 | 0.00 | 0.01 | 0.00 | 0.12 | 0.08 | 0.12 | 0.09 | 500 |
| 7 | 3 | 200 | 100 | 0.5 | 0 | 2.5 | 0.5 | 2 | 4.2 | 4.0 | 4.4 | 4.0 | 0.00 | 0.00 | 0.00 | 0.00 | 0.12 | 0.08 | 0.12 | 0.09 | 500 |
| 7 | 3 | 200 | 100 | 0.5 | 0.2 | 1.5 | 2 | 1 | 41.2 | 41.0 | 41.4 | 40.8 | 0.00 | 0.06 | 0.00 | 0.05 | 0.12 | 0.09 | 0.12 | 0.09 | 500 |
| 7 | 3 | 200 | 100 | 0.5 | 0.2 | 1.5 | 2 | 2 | 44.8 | 44.0 | 45.0 | 44.6 | 0.01 | 0.05 | 0.01 | 0.05 | 0.12 | 0.09 | 0.12 | 0.09 | 500 |
| 7 | 3 | 200 | 100 | 0.5 | 0.2 | 2.5 | 0.5 | 1 | 38.2 | 38.0 | 38.2 | 38.2 | 0.00 | 0.06 | 0.00 | 0.06 | 0.11 | 0.08 | 0.11 | 0.08 | 500 |
| 7 | 3 | 200 | 100 | 0.5 | 0.2 | 2.5 | 0.5 | 2 | 39.2 | 39.0 | 39.2 | 39.0 | 0.00 | 0.06 | 0.01 | 0.05 | 0.12 | 0.09 | 0.12 | 0.09 | 500 |
| 7 | 3 | 200 | 100 | 0.5 | 0.5 | 1.5 | 2 | 1 | 99.4 | 99.4 | 99.4 | 99.4 | 0.02 | 0.12 | 0.02 | 0.11 | 0.12 | 0.09 | 0.13 | 0.10 | 500 |
| 7 | 3 | 200 | 100 | 0.5 | 0.5 | 1.5 | 2 | 2 | 98.0 | 98.0 | 98.0 | 98.0 | 0.00 | 0.14 | 0.01 | 0.13 | 0.12 | 0.09 | 0.12 | 0.10 | 500 |
| 7 | 3 | 200 | 100 | 0.5 | 0.5 | 2.5 | 0.5 | 1 | 98.0 | 98.0 | 98.0 | 98.0 | 0.01 | 0.15 | 0.00 | 0.14 | 0.12 | 0.09 | 0.12 | 0.09 | 500 |
| 7 | 3 | 200 | 100 | 0.5 | 0.5 | 2.5 | 0.5 | 2 | 97.8 | 97.6 | 98.0 | 97.6 | 0.00 | 0.14 | 0.00 | 0.14 | 0.13 | 0.10 | 0.13 | 0.10 | 500 |
| 7 | 3 | 200 | 100 | 2 | 0 | 1.5 | 2 | 1 | 6.2 | 5.8 | 6.2 | 5.8 | 0.00 | 0.00 | 0.00 | 0.00 | 0.13 | 0.08 | 0.14 | 0.08 | 500 |
| 7 | 3 | 200 | 100 | 2 | 0 | 1.5 | 2 | 2 | 6.2 | 5.8 | 6.2 | 5.8 | 0.00 | 0.00 | 0.00 | 0.00 | 0.13 | 0.08 | 0.14 | 0.08 | 500 |
| 7 | 3 | 200 | 100 | 2 | 0 | 2.5 | 0.5 | 1 | 6.0 | 6.0 | 6.0 | 6.0 | 0.01 | 0.01 | 0.01 | 0.01 | 0.14 | 0.08 | 0.14 | 0.08 | 500 |
| 7 | 3 | 200 | 100 | 2 | 0 | 2.5 | 0.5 | 2 | 6.8 | 6.8 | 6.8 | 6.8 | 0.00 | 0.00 | 0.00 | 0.00 | 0.13 | 0.08 | 0.13 | 0.08 | 500 |
| 7 | 3 | 200 | 100 | 2 | 0.2 | 1.5 | 2 | 1 | 34.2 | 34.4 | 34.6 | 34.4 | 0.00 | 0.08 | 0.00 | 0.08 | 0.13 | 0.08 | 0.14 | 0.08 | 500 |
| 7 | 3 | 200 | 100 | 2 | 0.2 | 1.5 | 2 | 2 | 34.0 | 33.4 | 34.6 | 33.4 | 0.01 | 0.08 | 0.01 | 0.07 | 0.13 | 0.08 | 0.13 | 0.08 | 500 |
| 7 | 3 | 200 | 100 | 2 | 0.2 | 2.5 | 0.5 | 1 | 30.8 | 30.8 | 31.0 | 30.8 | 0.01 | 0.08 | 0.00 | 0.08 | 0.12 | 0.07 | 0.12 | 0.08 | 500 |
| 7 | 3 | 200 | 100 | 2 | 0.2 | 2.5 | 0.5 | 2 | 34.4 | 34.2 | 34.6 | 34.2 | 0.00 | 0.08 | 0.01 | 0.08 | 0.12 | 0.08 | 0.12 | 0.08 | 500 |
| 7 | 3 | 200 | 100 | 2 | 0.5 | 1.5 | 2 | 1 | 97.4 | 97.4 | 97.4 | 97.4 | 0.00 | 0.20 | 0.01 | 0.19 | 0.13 | 0.09 | 0.14 | 0.09 | 500 |
| 7 | 3 | 200 | 100 | 2 | 0.5 | 1.5 | 2 | 2 | 97.2 | 97.2 | 97.2 | 97.2 | 0.01 | 0.19 | 0.02 | 0.18 | 0.13 | 0.08 | 0.13 | 0.09 | 500 |
| 7 | 3 | 200 | 100 | 2 | 0.5 | 2.5 | 0.5 | 1 | 97.8 | 97.8 | 97.8 | 97.8 | 0.00 | 0.19 | 0.01 | 0.19 | 0.13 | 0.09 | 0.13 | 0.09 | 500 |
| 7 | 3 | 200 | 100 | 2 | 0.5 | 2.5 | 0.5 | 2 | 96.6 | 96.6 | 96.6 | 96.6 | 0.01 | 0.20 | 0.00 | 0.19 | 0.14 | 0.09 | 0.15 | 0.10 | 500 |
| 7 | 3 | 200 | 250 | 0 | 0 | 1.5 | 2 | 1 | 7.6 | 7.2 | 7.4 | 7.2 | 0.01 | 0.01 | 0.01 | 0.01 | 0.12 | 0.09 | 0.12 | 0.09 | 500 |
| 7 | 3 | 200 | 250 | 0 | 0 | 1.5 | 2 | 2 | 7.6 | 7.2 | 7.4 | 7.2 | 0.01 | 0.01 | 0.01 | 0.01 | 0.12 | 0.09 | 0.12 | 0.09 | 500 |
| 7 | 3 | 200 | 250 | 0 | 0 | 2.5 | 0.5 | 1 | 5.8 | 5.8 | 5.8 | 5.8 | 0.00 | 0.00 | 0.00 | 0.00 | 0.12 | 0.09 | 0.12 | 0.09 | 500 |
| 7 | 3 | 200 | 250 | 0 | 0 | 2.5 | 0.5 | 2 | 4.0 | 4.0 | 4.0 | 4.0 | 0.01 | 0.01 | 0.01 | 0.01 | 0.11 | 0.08 | 0.11 | 0.08 | 500 |
| 7 | 3 | 200 | 250 | 0 | 0.2 | 1.5 | 2 | 1 | 40.8 | 40.8 | 40.8 | 40.8 | 0.00 | 0.05 | 0.00 | 0.05 | 0.12 | 0.09 | 0.12 | 0.09 | 500 |
| 7 | 3 | 200 | 250 | 0 | 0.2 | 1.5 | 2 | 2 | 36.6 | 36.2 | 36.4 | 36.2 | 0.00 | 0.05 | 0.00 | 0.05 | 0.11 | 0.08 | 0.12 | 0.09 | 500 |
| 7 | 3 | 200 | 250 | 0 | 0.2 | 2.5 | 0.5 | 1 | 37.8 | 37.8 | 38.0 | 37.8 | 0.00 | 0.06 | 0.00 | 0.06 | 0.12 | 0.09 | 0.12 | 0.09 | 500 |
| 7 | 3 | 200 | 250 | 0 | 0.2 | 2.5 | 0.5 | 2 | 39.6 | 39.4 | 39.6 | 39.0 | 0.00 | 0.06 | 0.00 | 0.05 | 0.12 | 0.08 | 0.12 | 0.08 | 500 |
| 7 | 3 | 200 | 250 | 0 | 0.5 | 1.5 | 2 | 1 | 99.2 | 99.0 | 99.2 | 99.2 | 0.00 | 0.13 | 0.00 | 0.13 | 0.12 | 0.09 | 0.12 | 0.09 | 500 |
| 7 | 3 | 200 | 250 | 0 | 0.5 | 1.5 | 2 | 2 | 99.0 | 99.0 | 99.0 | 99.0 | 0.00 | 0.13 | 0.00 | 0.13 | 0.12 | 0.09 | 0.12 | 0.10 | 500 |
| 7 | 3 | 200 | 250 | 0 | 0.5 | 2.5 | 0.5 | 1 | 98.4 | 98.4 | 98.4 | 98.4 | 0.00 | 0.13 | 0.00 | 0.13 | 0.12 | 0.09 | 0.12 | 0.09 | 500 |
| 7 | 3 | 200 | 250 | 0 | 0.5 | 2.5 | 0.5 | 2 | 97.8 | 97.8 | 97.8 | 97.8 | 0.00 | 0.14 | 0.00 | 0.13 | 0.12 | 0.09 | 0.12 | 0.09 | 500 |
| 7 | 3 | 200 | 250 | 0.5 | 0 | 1.5 | 2 | 1 | 5.4 | 5.2 | 5.6 | 5.2 | 0.00 | 0.00 | 0.00 | 0.00 | 0.12 | 0.09 | 0.12 | 0.09 | 500 |
| 7 | 3 | 200 | 250 | 0.5 | 0 | 1.5 | 2 | 2 | 5.4 | 5.2 | 5.6 | 5.2 | 0.00 | 0.00 | 0.00 | 0.00 | 0.12 | 0.09 | 0.12 | 0.09 | 500 |
| 7 | 3 | 200 | 250 | 0.5 | 0 | 2.5 | 0.5 | 1 | 5.0 | 5.0 | 5.0 | 5.0 | 0.00 | 0.00 | 0.00 | 0.00 | 0.12 | 0.08 | 0.12 | 0.08 | 500 |
| 7 | 3 | 200 | 250 | 0.5 | 0 | 2.5 | 0.5 | 2 | 6.2 | 6.2 | 6.2 | 6.2 | 0.00 | 0.00 | 0.00 | 0.00 | 0.12 | 0.09 | 0.12 | 0.09 | 500 |
| 7 | 3 | 200 | 250 | 0.5 | 0.2 | 1.5 | 2 | 1 | 40.0 | 40.0 | 40.2 | 40.0 | 0.00 | 0.06 | 0.00 | 0.06 | 0.12 | 0.08 | 0.12 | 0.08 | 500 |
| 7 | 3 | 200 | 250 | 0.5 | 0.2 | 1.5 | 2 | 2 | 37.2 | 37.2 | 37.2 | 37.2 | 0.01 | 0.06 | 0.01 | 0.06 | 0.11 | 0.08 | 0.11 | 0.08 | 500 |
| 7 | 3 | 200 | 250 | 0.5 | 0.2 | 2.5 | 0.5 | 1 | 38.8 | 38.6 | 38.8 | 38.6 | 0.00 | 0.06 | 0.00 | 0.06 | 0.12 | 0.08 | 0.12 | 0.08 | 500 |
| 7 | 3 | 200 | 250 | 0.5 | 0.2 | 2.5 | 0.5 | 2 | 39.6 | 39.6 | 39.6 | 39.6 | 0.00 | 0.06 | 0.00 | 0.06 | 0.12 | 0.09 | 0.12 | 0.09 | 500 |
| 7 | 3 | 200 | 250 | 0.5 | 0.5 | 1.5 | 2 | 1 | 99.6 | 99.6 | 99.6 | 99.6 | 0.00 | 0.13 | 0.00 | 0.13 | 0.12 | 0.09 | 0.12 | 0.09 | 500 |
| 7 | 3 | 200 | 250 | 0.5 | 0.5 | 1.5 | 2 | 2 | 99.4 | 99.4 | 99.4 | 99.4 | 0.00 | 0.13 | 0.01 | 0.13 | 0.12 | 0.09 | 0.12 | 0.09 | 500 |
| 7 | 3 | 200 | 250 | 0.5 | 0.5 | 2.5 | 0.5 | 1 | 98.8 | 98.8 | 98.8 | 98.8 | 0.00 | 0.14 | 0.00 | 0.14 | 0.12 | 0.09 | 0.12 | 0.09 | 500 |
| 7 | 3 | 200 | 250 | 0.5 | 0.5 | 2.5 | 0.5 | 2 | 98.6 | 98.6 | 98.6 | 98.6 | 0.00 | 0.14 | 0.00 | 0.14 | 0.12 | 0.09 | 0.12 | 0.09 | 500 |
| 7 | 3 | 200 | 250 | 2 | 0 | 1.5 | 2 | 1 | 5.2 | 5.2 | 5.2 | 5.2 | 0.00 | 0.00 | 0.00 | 0.00 | 0.14 | 0.08 | 0.14 | 0.08 | 500 |
| 7 | 3 | 200 | 250 | 2 | 0 | 1.5 | 2 | 2 | 5.2 | 5.2 | 5.2 | 5.2 | 0.00 | 0.00 | 0.00 | 0.00 | 0.14 | 0.08 | 0.14 | 0.08 | 500 |
| 7 | 3 | 200 | 250 | 2 | 0 | 2.5 | 0.5 | 1 | 6.4 | 6.4 | 6.6 | 6.4 | 0.00 | 0.00 | 0.00 | 0.00 | 0.13 | 0.08 | 0.13 | 0.08 | 500 |
| 7 | 3 | 200 | 250 | 2 | 0 | 2.5 | 0.5 | 2 | 3.0 | 2.8 | 3.2 | 2.8 | 0.00 | 0.00 | 0.00 | 0.00 | 0.12 | 0.08 | 0.12 | 0.08 | 500 |
| 7 | 3 | 200 | 250 | 2 | 0.2 | 1.5 | 2 | 1 | 36.6 | 36.6 | 36.6 | 36.4 | 0.01 | 0.08 | 0.01 | 0.08 | 0.13 | 0.08 | 0.13 | 0.08 | 500 |
| 7 | 3 | 200 | 250 | 2 | 0.2 | 1.5 | 2 | 2 | 33.8 | 33.6 | 33.8 | 33.8 | 0.00 | 0.08 | 0.00 | 0.08 | 0.13 | 0.08 | 0.13 | 0.08 | 500 |
| 7 | 3 | 200 | 250 | 2 | 0.2 | 2.5 | 0.5 | 1 | 35.6 | 35.0 | 35.6 | 35.2 | 0.01 | 0.08 | 0.01 | 0.08 | 0.13 | 0.08 | 0.13 | 0.08 | 500 |
| 7 | 3 | 200 | 250 | 2 | 0.2 | 2.5 | 0.5 | 2 | 37.8 | 37.0 | 38.0 | 37.2 | 0.01 | 0.07 | 0.01 | 0.07 | 0.13 | 0.08 | 0.13 | 0.08 | 500 |
| 7 | 3 | 200 | 250 | 2 | 0.5 | 1.5 | 2 | 1 | 96.4 | 96.4 | 96.4 | 96.4 | 0.00 | 0.20 | 0.00 | 0.20 | 0.14 | 0.09 | 0.14 | 0.09 | 500 |
| 7 | 3 | 200 | 250 | 2 | 0.5 | 1.5 | 2 | 2 | 97.6 | 97.6 | 97.6 | 97.6 | 0.00 | 0.20 | 0.00 | 0.20 | 0.13 | 0.08 | 0.13 | 0.08 | 500 |
| 7 | 3 | 200 | 250 | 2 | 0.5 | 2.5 | 0.5 | 1 | 98.0 | 97.8 | 98.0 | 97.8 | 0.00 | 0.19 | 0.01 | 0.19 | 0.12 | 0.08 | 0.12 | 0.08 | 500 |
| 7 | 3 | 200 | 250 | 2 | 0.5 | 2.5 | 0.5 | 2 | 97.8 | 97.8 | 97.8 | 97.8 | 0.01 | 0.19 | 0.01 | 0.18 | 0.13 | 0.08 | 0.13 | 0.08 | 500 |
| 7 | 3 | 200 | 500 | 0 | 0 | 1.5 | 2 | 1 | 4.6 | 4.6 | 4.6 | 4.6 | 0.00 | 0.00 | 0.00 | 0.00 | 0.12 | 0.09 | 0.12 | 0.09 | 500 |
| 7 | 3 | 200 | 500 | 0 | 0 | 1.5 | 2 | 2 | 5.4 | 5.4 | 5.6 | 5.4 | 0.01 | 0.01 | 0.01 | 0.01 | 0.12 | 0.09 | 0.12 | 0.09 | 500 |
| 7 | 3 | 200 | 500 | 0 | 0 | 2.5 | 0.5 | 1 | 4.6 | 4.6 | 4.6 | 4.6 | 0.01 | 0.01 | 0.01 | 0.01 | 0.11 | 0.08 | 0.11 | 0.08 | 500 |
| 7 | 3 | 200 | 500 | 0 | 0 | 2.5 | 0.5 | 2 | 5.4 | 5.4 | 5.4 | 5.4 | 0.01 | 0.01 | 0.01 | 0.01 | 0.12 | 0.08 | 0.12 | 0.08 | 500 |
| 7 | 3 | 200 | 500 | 0 | 0.2 | 1.5 | 2 | 1 | 44.4 | 44.2 | 44.4 | 44.2 | 0.01 | 0.05 | 0.01 | 0.05 | 0.12 | 0.09 | 0.12 | 0.09 | 500 |
| 7 | 3 | 200 | 500 | 0 | 0.2 | 1.5 | 2 | 2 | 38.0 | 37.6 | 38.2 | 37.6 | 0.00 | 0.05 | 0.00 | 0.05 | 0.11 | 0.08 | 0.11 | 0.08 | 500 |
| 7 | 3 | 200 | 500 | 0 | 0.2 | 2.5 | 0.5 | 1 | 42.0 | 41.6 | 42.2 | 41.6 | 0.00 | 0.06 | 0.00 | 0.06 | 0.13 | 0.09 | 0.13 | 0.09 | 500 |
| 7 | 3 | 200 | 500 | 0 | 0.2 | 2.5 | 0.5 | 2 | 37.6 | 37.2 | 37.6 | 37.4 | 0.00 | 0.06 | 0.00 | 0.06 | 0.12 | 0.09 | 0.12 | 0.09 | 500 |
| 7 | 3 | 200 | 500 | 0 | 0.5 | 1.5 | 2 | 1 | 99.2 | 99.2 | 99.2 | 99.2 | 0.01 | 0.12 | 0.01 | 0.12 | 0.12 | 0.09 | 0.12 | 0.09 | 500 |
| 7 | 3 | 200 | 500 | 0 | 0.5 | 1.5 | 2 | 2 | 99.0 | 99.0 | 99.0 | 99.0 | 0.00 | 0.13 | 0.00 | 0.13 | 0.12 | 0.09 | 0.12 | 0.09 | 500 |
| 7 | 3 | 200 | 500 | 0 | 0.5 | 2.5 | 0.5 | 1 | 99.4 | 99.4 | 99.4 | 99.4 | 0.00 | 0.13 | 0.00 | 0.13 | 0.12 | 0.09 | 0.12 | 0.09 | 500 |
| 7 | 3 | 200 | 500 | 0 | 0.5 | 2.5 | 0.5 | 2 | 99.2 | 99.2 | 99.2 | 99.2 | 0.01 | 0.13 | 0.01 | 0.13 | 0.12 | 0.09 | 0.12 | 0.09 | 500 |
| 7 | 3 | 200 | 500 | 0.5 | 0 | 1.5 | 2 | 1 | 5.8 | 5.4 | 6.0 | 5.4 | 0.00 | 0.00 | 0.00 | 0.00 | 0.12 | 0.09 | 0.12 | 0.09 | 500 |
| 7 | 3 | 200 | 500 | 0.5 | 0 | 1.5 | 2 | 2 | 5.2 | 5.2 | 5.2 | 5.2 | 0.00 | 0.00 | 0.00 | 0.00 | 0.12 | 0.08 | 0.12 | 0.08 | 500 |
| 7 | 3 | 200 | 500 | 0.5 | 0 | 2.5 | 0.5 | 1 | 6.4 | 6.4 | 6.6 | 6.4 | 0.00 | 0.00 | 0.00 | 0.00 | 0.12 | 0.09 | 0.12 | 0.09 | 500 |
| 7 | 3 | 200 | 500 | 0.5 | 0 | 2.5 | 0.5 | 2 | 6.0 | 6.0 | 6.0 | 6.0 | 0.01 | 0.00 | 0.01 | 0.00 | 0.12 | 0.09 | 0.12 | 0.09 | 500 |
| 7 | 3 | 200 | 500 | 0.5 | 0.2 | 1.5 | 2 | 1 | 38.2 | 38.0 | 38.2 | 38.2 | 0.01 | 0.06 | 0.01 | 0.06 | 0.12 | 0.09 | 0.12 | 0.09 | 500 |
| 7 | 3 | 200 | 500 | 0.5 | 0.2 | 1.5 | 2 | 2 | 37.0 | 36.8 | 37.2 | 36.6 | 0.01 | 0.06 | 0.01 | 0.06 | 0.13 | 0.09 | 0.13 | 0.09 | 500 |
| 7 | 3 | 200 | 500 | 0.5 | 0.2 | 2.5 | 0.5 | 1 | 40.4 | 40.2 | 40.4 | 40.4 | 0.00 | 0.05 | 0.00 | 0.06 | 0.12 | 0.09 | 0.12 | 0.09 | 500 |
| 7 | 3 | 200 | 500 | 0.5 | 0.2 | 2.5 | 0.5 | 2 | 39.4 | 39.2 | 39.6 | 39.2 | 0.00 | 0.06 | 0.00 | 0.06 | 0.11 | 0.08 | 0.11 | 0.08 | 500 |
| 7 | 3 | 200 | 500 | 0.5 | 0.5 | 1.5 | 2 | 1 | 99.0 | 99.0 | 99.0 | 99.0 | 0.00 | 0.13 | 0.00 | 0.13 | 0.11 | 0.09 | 0.11 | 0.09 | 500 |
| 7 | 3 | 200 | 500 | 0.5 | 0.5 | 1.5 | 2 | 2 | 98.6 | 98.6 | 98.6 | 98.6 | 0.00 | 0.13 | 0.00 | 0.13 | 0.12 | 0.10 | 0.12 | 0.09 | 500 |
| 7 | 3 | 200 | 500 | 0.5 | 0.5 | 2.5 | 0.5 | 1 | 98.4 | 98.4 | 98.4 | 98.4 | 0.00 | 0.14 | 0.00 | 0.14 | 0.12 | 0.09 | 0.12 | 0.09 | 500 |
| 7 | 3 | 200 | 500 | 0.5 | 0.5 | 2.5 | 0.5 | 2 | 98.6 | 98.6 | 98.6 | 98.6 | 0.02 | 0.13 | 0.02 | 0.13 | 0.12 | 0.09 | 0.12 | 0.09 | 500 |
| 7 | 3 | 200 | 500 | 2 | 0 | 1.5 | 2 | 1 | 4.6 | 4.4 | 4.6 | 4.4 | 0.01 | 0.00 | 0.01 | 0.00 | 0.13 | 0.08 | 0.13 | 0.08 | 500 |
| 7 | 3 | 200 | 500 | 2 | 0 | 1.5 | 2 | 2 | 4.2 | 4.2 | 4.2 | 4.0 | 0.00 | 0.00 | 0.00 | 0.00 | 0.13 | 0.07 | 0.13 | 0.07 | 500 |
| 7 | 3 | 200 | 500 | 2 | 0 | 2.5 | 0.5 | 1 | 5.0 | 4.8 | 5.0 | 4.8 | 0.01 | 0.00 | 0.01 | 0.00 | 0.13 | 0.08 | 0.13 | 0.08 | 500 |
| 7 | 3 | 200 | 500 | 2 | 0 | 2.5 | 0.5 | 2 | 4.8 | 4.6 | 4.8 | 4.6 | 0.01 | 0.00 | 0.01 | 0.00 | 0.13 | 0.08 | 0.13 | 0.08 | 500 |
| 7 | 3 | 200 | 500 | 2 | 0.2 | 1.5 | 2 | 1 | 36.4 | 36.0 | 36.4 | 36.2 | 0.01 | 0.08 | 0.01 | 0.08 | 0.13 | 0.08 | 0.13 | 0.08 | 500 |
| 7 | 3 | 200 | 500 | 2 | 0.2 | 1.5 | 2 | 2 | 34.6 | 34.0 | 34.6 | 34.0 | 0.00 | 0.08 | 0.00 | 0.08 | 0.14 | 0.08 | 0.14 | 0.08 | 500 |
| 7 | 3 | 200 | 500 | 2 | 0.2 | 2.5 | 0.5 | 1 | 36.0 | 35.4 | 36.0 | 35.4 | 0.00 | 0.08 | 0.00 | 0.08 | 0.13 | 0.08 | 0.13 | 0.08 | 500 |
| 7 | 3 | 200 | 500 | 2 | 0.2 | 2.5 | 0.5 | 2 | 35.2 | 35.0 | 35.4 | 35.0 | 0.01 | 0.08 | 0.01 | 0.08 | 0.13 | 0.08 | 0.13 | 0.08 | 500 |
| 7 | 3 | 200 | 500 | 2 | 0.5 | 1.5 | 2 | 1 | 97.2 | 97.2 | 97.2 | 97.2 | 0.00 | 0.20 | 0.00 | 0.20 | 0.13 | 0.09 | 0.13 | 0.08 | 500 |
| 7 | 3 | 200 | 500 | 2 | 0.5 | 1.5 | 2 | 2 | 97.2 | 97.2 | 97.2 | 97.2 | 0.00 | 0.20 | 0.01 | 0.20 | 0.13 | 0.08 | 0.13 | 0.08 | 500 |
| 7 | 3 | 200 | 500 | 2 | 0.5 | 2.5 | 0.5 | 1 | 96.4 | 96.2 | 96.4 | 96.2 | 0.01 | 0.20 | 0.01 | 0.20 | 0.13 | 0.09 | 0.13 | 0.09 | 500 |
| 7 | 3 | 200 | 500 | 2 | 0.5 | 2.5 | 0.5 | 2 | 97.2 | 97.2 | 97.2 | 97.2 | 0.01 | 0.19 | 0.01 | 0.19 | 0.13 | 0.09 | 0.13 | 0.09 | 500 |
| 7 | 3 | 500 | 100 | 0 | 0 | 1.5 | 2 | 1 | 4.6 | 4.6 | 4.8 | 4.6 | 0.00 | 0.00 | 0.00 | 0.00 | 0.07 | 0.05 | 0.07 | 0.05 | 500 |
| 7 | 3 | 500 | 100 | 0 | 0 | 1.5 | 2 | 2 | 4.6 | 4.6 | 4.8 | 4.6 | 0.00 | 0.00 | 0.00 | 0.00 | 0.07 | 0.05 | 0.07 | 0.05 | 500 |
| 7 | 3 | 500 | 100 | 0 | 0 | 2.5 | 0.5 | 1 | 4.8 | 4.8 | 4.8 | 4.8 | 0.00 | 0.00 | 0.00 | 0.00 | 0.08 | 0.05 | 0.08 | 0.05 | 500 |
| 7 | 3 | 500 | 100 | 0 | 0 | 2.5 | 0.5 | 2 | 4.8 | 4.8 | 5.0 | 4.8 | 0.00 | 0.00 | 0.00 | 0.00 | 0.08 | 0.05 | 0.08 | 0.06 | 500 |
| 7 | 3 | 500 | 100 | 0 | 0.2 | 1.5 | 2 | 1 | 76.2 | 76.0 | 76.2 | 76.2 | 0.00 | 0.05 | 0.00 | 0.05 | 0.08 | 0.06 | 0.08 | 0.06 | 500 |
| 7 | 3 | 500 | 100 | 0 | 0.2 | 1.5 | 2 | 2 | 77.4 | 77.0 | 77.2 | 77.2 | 0.00 | 0.05 | 0.00 | 0.05 | 0.08 | 0.06 | 0.08 | 0.06 | 500 |
| 7 | 3 | 500 | 100 | 0 | 0.2 | 2.5 | 0.5 | 1 | 77.8 | 77.4 | 77.6 | 77.4 | 0.00 | 0.06 | 0.00 | 0.05 | 0.07 | 0.05 | 0.07 | 0.05 | 500 |
| 7 | 3 | 500 | 100 | 0 | 0.2 | 2.5 | 0.5 | 2 | 81.4 | 81.0 | 81.4 | 81.0 | 0.01 | 0.05 | 0.01 | 0.05 | 0.07 | 0.05 | 0.07 | 0.05 | 500 |
| 7 | 3 | 500 | 100 | 0 | 0.5 | 1.5 | 2 | 1 | 100.0 | 100.0 | 100.0 | 100.0 | 0.00 | 0.13 | 0.01 | 0.12 | 0.07 | 0.06 | 0.08 | 0.06 | 500 |
| 7 | 3 | 500 | 100 | 0 | 0.5 | 1.5 | 2 | 2 | 100.0 | 100.0 | 100.0 | 100.0 | 0.00 | 0.13 | 0.01 | 0.12 | 0.08 | 0.06 | 0.08 | 0.07 | 500 |
| 7 | 3 | 500 | 100 | 0 | 0.5 | 2.5 | 0.5 | 1 | 100.0 | 100.0 | 100.0 | 100.0 | 0.00 | 0.13 | 0.01 | 0.13 | 0.07 | 0.05 | 0.08 | 0.06 | 500 |
| 7 | 3 | 500 | 100 | 0 | 0.5 | 2.5 | 0.5 | 2 | 100.0 | 100.0 | 100.0 | 100.0 | 0.00 | 0.14 | 0.01 | 0.13 | 0.08 | 0.06 | 0.08 | 0.07 | 500 |
| 7 | 3 | 500 | 100 | 0.5 | 0 | 1.5 | 2 | 1 | 6.8 | 6.8 | 7.0 | 7.0 | 0.00 | 0.00 | 0.00 | 0.00 | 0.08 | 0.06 | 0.08 | 0.06 | 500 |
| 7 | 3 | 500 | 100 | 0.5 | 0 | 1.5 | 2 | 2 | 6.8 | 6.8 | 7.0 | 7.0 | 0.00 | 0.00 | 0.00 | 0.00 | 0.08 | 0.06 | 0.08 | 0.06 | 500 |
| 7 | 3 | 500 | 100 | 0.5 | 0 | 2.5 | 0.5 | 1 | 4.2 | 4.0 | 4.2 | 4.0 | 0.00 | 0.00 | 0.00 | 0.00 | 0.07 | 0.05 | 0.07 | 0.05 | 500 |
| 7 | 3 | 500 | 100 | 0.5 | 0 | 2.5 | 0.5 | 2 | 5.6 | 5.6 | 5.6 | 5.6 | 0.00 | 0.00 | 0.00 | 0.00 | 0.08 | 0.05 | 0.08 | 0.05 | 500 |
| 7 | 3 | 500 | 100 | 0.5 | 0.2 | 1.5 | 2 | 1 | 77.0 | 77.0 | 77.0 | 76.8 | 0.00 | 0.06 | 0.00 | 0.05 | 0.07 | 0.05 | 0.07 | 0.05 | 500 |
| 7 | 3 | 500 | 100 | 0.5 | 0.2 | 1.5 | 2 | 2 | 75.8 | 75.6 | 75.8 | 75.6 | 0.00 | 0.06 | 0.00 | 0.05 | 0.08 | 0.06 | 0.08 | 0.06 | 500 |
| 7 | 3 | 500 | 100 | 0.5 | 0.2 | 2.5 | 0.5 | 1 | 75.2 | 75.2 | 75.2 | 75.2 | 0.00 | 0.06 | 0.00 | 0.05 | 0.08 | 0.05 | 0.08 | 0.06 | 500 |
| 7 | 3 | 500 | 100 | 0.5 | 0.2 | 2.5 | 0.5 | 2 | 75.6 | 75.2 | 75.6 | 75.2 | 0.00 | 0.06 | 0.00 | 0.06 | 0.08 | 0.05 | 0.08 | 0.06 | 500 |
| 7 | 3 | 500 | 100 | 0.5 | 0.5 | 1.5 | 2 | 1 | 100.0 | 100.0 | 100.0 | 100.0 | 0.00 | 0.13 | 0.01 | 0.13 | 0.08 | 0.06 | 0.08 | 0.06 | 500 |
| 7 | 3 | 500 | 100 | 0.5 | 0.5 | 1.5 | 2 | 2 | 100.0 | 100.0 | 100.0 | 100.0 | 0.00 | 0.13 | 0.01 | 0.12 | 0.08 | 0.06 | 0.08 | 0.07 | 500 |
| 7 | 3 | 500 | 100 | 0.5 | 0.5 | 2.5 | 0.5 | 1 | 100.0 | 100.0 | 100.0 | 100.0 | 0.00 | 0.14 | 0.01 | 0.13 | 0.07 | 0.06 | 0.08 | 0.06 | 500 |
| 7 | 3 | 500 | 100 | 0.5 | 0.5 | 2.5 | 0.5 | 2 | 100.0 | 100.0 | 100.0 | 100.0 | 0.00 | 0.14 | 0.00 | 0.14 | 0.07 | 0.05 | 0.08 | 0.06 | 500 |
| 7 | 3 | 500 | 100 | 2 | 0 | 1.5 | 2 | 1 | 4.4 | 4.4 | 4.4 | 4.4 | 0.00 | 0.00 | 0.00 | 0.00 | 0.08 | 0.05 | 0.08 | 0.05 | 500 |
| 7 | 3 | 500 | 100 | 2 | 0 | 1.5 | 2 | 2 | 4.4 | 4.4 | 4.4 | 4.4 | 0.00 | 0.00 | 0.00 | 0.00 | 0.08 | 0.05 | 0.08 | 0.05 | 500 |
| 7 | 3 | 500 | 100 | 2 | 0 | 2.5 | 0.5 | 1 | 5.6 | 5.6 | 5.6 | 5.6 | 0.00 | 0.00 | 0.00 | 0.00 | 0.08 | 0.05 | 0.08 | 0.05 | 500 |
| 7 | 3 | 500 | 100 | 2 | 0 | 2.5 | 0.5 | 2 | 4.2 | 4.2 | 4.2 | 4.2 | 0.00 | 0.00 | 0.00 | 0.00 | 0.08 | 0.05 | 0.08 | 0.05 | 500 |
| 7 | 3 | 500 | 100 | 2 | 0.2 | 1.5 | 2 | 1 | 70.6 | 70.6 | 70.0 | 70.0 | 0.00 | 0.08 | 0.01 | 0.08 | 0.08 | 0.05 | 0.08 | 0.05 | 500 |
| 7 | 3 | 500 | 100 | 2 | 0.2 | 1.5 | 2 | 2 | 67.4 | 67.2 | 67.4 | 67.2 | 0.00 | 0.08 | 0.00 | 0.08 | 0.08 | 0.05 | 0.09 | 0.05 | 500 |
| 7 | 3 | 500 | 100 | 2 | 0.2 | 2.5 | 0.5 | 1 | 68.4 | 68.4 | 68.8 | 68.8 | 0.00 | 0.08 | 0.00 | 0.08 | 0.08 | 0.05 | 0.09 | 0.05 | 500 |
| 7 | 3 | 500 | 100 | 2 | 0.2 | 2.5 | 0.5 | 2 | 67.4 | 67.4 | 67.4 | 67.4 | 0.00 | 0.08 | 0.00 | 0.08 | 0.08 | 0.05 | 0.08 | 0.05 | 500 |
| 7 | 3 | 500 | 100 | 2 | 0.5 | 1.5 | 2 | 1 | 100.0 | 100.0 | 100.0 | 100.0 | 0.00 | 0.20 | 0.01 | 0.19 | 0.08 | 0.05 | 0.08 | 0.06 | 500 |
| 7 | 3 | 500 | 100 | 2 | 0.5 | 1.5 | 2 | 2 | 100.0 | 100.0 | 100.0 | 100.0 | 0.00 | 0.20 | 0.01 | 0.19 | 0.08 | 0.05 | 0.08 | 0.06 | 500 |
| 7 | 3 | 500 | 100 | 2 | 0.5 | 2.5 | 0.5 | 1 | 100.0 | 100.0 | 100.0 | 100.0 | 0.00 | 0.19 | 0.01 | 0.19 | 0.08 | 0.05 | 0.09 | 0.06 | 500 |
| 7 | 3 | 500 | 100 | 2 | 0.5 | 2.5 | 0.5 | 2 | 100.0 | 100.0 | 100.0 | 100.0 | 0.01 | 0.19 | 0.01 | 0.18 | 0.08 | 0.05 | 0.08 | 0.06 | 500 |
| 7 | 3 | 500 | 250 | 0 | 0 | 1.5 | 2 | 1 | 5.2 | 5.2 | 5.2 | 5.2 | 0.00 | 0.00 | 0.00 | 0.00 | 0.07 | 0.05 | 0.07 | 0.05 | 500 |
| 7 | 3 | 500 | 250 | 0 | 0 | 1.5 | 2 | 2 | 5.2 | 5.2 | 5.2 | 5.2 | 0.00 | 0.00 | 0.00 | 0.00 | 0.07 | 0.05 | 0.07 | 0.05 | 500 |
| 7 | 3 | 500 | 250 | 0 | 0 | 2.5 | 0.5 | 1 | 6.6 | 6.6 | 6.6 | 6.6 | 0.00 | 0.00 | 0.00 | 0.00 | 0.08 | 0.06 | 0.08 | 0.06 | 500 |
| 7 | 3 | 500 | 250 | 0 | 0 | 2.5 | 0.5 | 2 | 5.2 | 5.2 | 5.2 | 5.2 | 0.00 | 0.00 | 0.00 | 0.00 | 0.07 | 0.05 | 0.07 | 0.05 | 500 |
| 7 | 3 | 500 | 250 | 0 | 0.2 | 1.5 | 2 | 1 | 76.8 | 76.6 | 77.0 | 76.8 | 0.00 | 0.05 | 0.00 | 0.05 | 0.07 | 0.05 | 0.07 | 0.05 | 500 |
| 7 | 3 | 500 | 250 | 0 | 0.2 | 1.5 | 2 | 2 | 77.4 | 76.8 | 77.2 | 77.0 | 0.00 | 0.05 | 0.00 | 0.05 | 0.07 | 0.05 | 0.07 | 0.05 | 500 |
| 7 | 3 | 500 | 250 | 0 | 0.2 | 2.5 | 0.5 | 1 | 75.4 | 75.2 | 75.4 | 75.2 | 0.00 | 0.06 | 0.00 | 0.05 | 0.07 | 0.05 | 0.07 | 0.05 | 500 |
| 7 | 3 | 500 | 250 | 0 | 0.2 | 2.5 | 0.5 | 2 | 75.8 | 75.6 | 75.8 | 75.6 | 0.00 | 0.06 | 0.00 | 0.06 | 0.07 | 0.05 | 0.07 | 0.05 | 500 |
| 7 | 3 | 500 | 250 | 0 | 0.5 | 1.5 | 2 | 1 | 100.0 | 100.0 | 100.0 | 100.0 | 0.01 | 0.13 | 0.01 | 0.12 | 0.08 | 0.06 | 0.08 | 0.06 | 500 |
| 7 | 3 | 500 | 250 | 0 | 0.5 | 1.5 | 2 | 2 | 100.0 | 100.0 | 100.0 | 100.0 | 0.00 | 0.13 | 0.00 | 0.13 | 0.08 | 0.06 | 0.08 | 0.06 | 500 |
| 7 | 3 | 500 | 250 | 0 | 0.5 | 2.5 | 0.5 | 1 | 100.0 | 100.0 | 100.0 | 100.0 | 0.00 | 0.14 | 0.00 | 0.14 | 0.08 | 0.06 | 0.08 | 0.06 | 500 |
| 7 | 3 | 500 | 250 | 0 | 0.5 | 2.5 | 0.5 | 2 | 100.0 | 100.0 | 100.0 | 100.0 | 0.00 | 0.14 | 0.00 | 0.13 | 0.08 | 0.06 | 0.08 | 0.06 | 500 |
| 7 | 3 | 500 | 250 | 0.5 | 0 | 1.5 | 2 | 1 | 5.2 | 5.2 | 5.2 | 5.2 | 0.01 | 0.00 | 0.01 | 0.00 | 0.07 | 0.05 | 0.07 | 0.05 | 500 |
| 7 | 3 | 500 | 250 | 0.5 | 0 | 1.5 | 2 | 2 | 5.2 | 5.2 | 5.2 | 5.2 | 0.01 | 0.00 | 0.01 | 0.00 | 0.07 | 0.05 | 0.07 | 0.05 | 500 |
| 7 | 3 | 500 | 250 | 0.5 | 0 | 2.5 | 0.5 | 1 | 6.2 | 5.8 | 6.2 | 5.8 | 0.00 | 0.00 | 0.00 | 0.00 | 0.08 | 0.05 | 0.08 | 0.06 | 500 |
| 7 | 3 | 500 | 250 | 0.5 | 0 | 2.5 | 0.5 | 2 | 6.2 | 5.8 | 6.2 | 5.8 | 0.00 | 0.00 | 0.00 | 0.00 | 0.08 | 0.05 | 0.08 | 0.06 | 500 |
| 7 | 3 | 500 | 250 | 0.5 | 0.2 | 1.5 | 2 | 1 | 75.0 | 75.0 | 75.0 | 75.0 | 0.00 | 0.05 | 0.00 | 0.05 | 0.08 | 0.06 | 0.08 | 0.06 | 500 |
| 7 | 3 | 500 | 250 | 0.5 | 0.2 | 1.5 | 2 | 2 | 74.8 | 74.4 | 75.2 | 74.2 | 0.00 | 0.06 | 0.00 | 0.06 | 0.07 | 0.05 | 0.07 | 0.05 | 500 |
| 7 | 3 | 500 | 250 | 0.5 | 0.2 | 2.5 | 0.5 | 1 | 79.0 | 79.0 | 79.2 | 79.0 | 0.00 | 0.06 | 0.01 | 0.05 | 0.07 | 0.05 | 0.07 | 0.05 | 500 |
| 7 | 3 | 500 | 250 | 0.5 | 0.2 | 2.5 | 0.5 | 2 | 73.8 | 73.8 | 73.8 | 73.8 | 0.00 | 0.06 | 0.00 | 0.06 | 0.08 | 0.05 | 0.08 | 0.06 | 500 |
| 7 | 3 | 500 | 250 | 0.5 | 0.5 | 1.5 | 2 | 1 | 100.0 | 100.0 | 100.0 | 100.0 | 0.01 | 0.14 | 0.00 | 0.14 | 0.08 | 0.06 | 0.08 | 0.06 | 500 |
| 7 | 3 | 500 | 250 | 0.5 | 0.5 | 1.5 | 2 | 2 | 100.0 | 100.0 | 100.0 | 100.0 | 0.00 | 0.13 | 0.00 | 0.13 | 0.08 | 0.06 | 0.08 | 0.06 | 500 |
| 7 | 3 | 500 | 250 | 0.5 | 0.5 | 2.5 | 0.5 | 1 | 100.0 | 100.0 | 100.0 | 100.0 | 0.00 | 0.14 | 0.01 | 0.13 | 0.08 | 0.06 | 0.08 | 0.06 | 500 |
| 7 | 3 | 500 | 250 | 0.5 | 0.5 | 2.5 | 0.5 | 2 | 100.0 | 100.0 | 100.0 | 100.0 | 0.00 | 0.14 | 0.01 | 0.14 | 0.08 | 0.06 | 0.08 | 0.06 | 500 |
| 7 | 3 | 500 | 250 | 2 | 0 | 1.5 | 2 | 1 | 3.6 | 3.6 | 3.6 | 3.6 | 0.01 | 0.00 | 0.01 | 0.00 | 0.08 | 0.05 | 0.08 | 0.05 | 500 |
| 7 | 3 | 500 | 250 | 2 | 0 | 1.5 | 2 | 2 | 3.6 | 3.6 | 3.6 | 3.6 | 0.01 | 0.00 | 0.01 | 0.00 | 0.08 | 0.05 | 0.08 | 0.05 | 500 |
| 7 | 3 | 500 | 250 | 2 | 0 | 2.5 | 0.5 | 1 | 5.4 | 5.4 | 5.4 | 5.4 | 0.00 | 0.00 | 0.00 | 0.00 | 0.08 | 0.05 | 0.08 | 0.05 | 500 |
| 7 | 3 | 500 | 250 | 2 | 0 | 2.5 | 0.5 | 2 | 6.0 | 6.0 | 6.0 | 6.0 | 0.00 | 0.00 | 0.00 | 0.00 | 0.08 | 0.05 | 0.08 | 0.05 | 500 |
| 7 | 3 | 500 | 250 | 2 | 0.2 | 1.5 | 2 | 1 | 67.8 | 67.8 | 67.8 | 67.8 | 0.00 | 0.08 | 0.00 | 0.08 | 0.08 | 0.05 | 0.09 | 0.05 | 500 |
| 7 | 3 | 500 | 250 | 2 | 0.2 | 1.5 | 2 | 2 | 64.8 | 64.8 | 64.8 | 64.8 | 0.01 | 0.09 | 0.00 | 0.08 | 0.08 | 0.05 | 0.08 | 0.05 | 500 |
| 7 | 3 | 500 | 250 | 2 | 0.2 | 2.5 | 0.5 | 1 | 65.6 | 65.6 | 65.6 | 65.6 | 0.00 | 0.08 | 0.00 | 0.08 | 0.09 | 0.05 | 0.09 | 0.05 | 500 |
| 7 | 3 | 500 | 250 | 2 | 0.2 | 2.5 | 0.5 | 2 | 66.2 | 66.2 | 66.2 | 66.2 | 0.00 | 0.08 | 0.00 | 0.08 | 0.08 | 0.05 | 0.08 | 0.05 | 500 |
| 7 | 3 | 500 | 250 | 2 | 0.5 | 1.5 | 2 | 1 | 100.0 | 100.0 | 100.0 | 100.0 | 0.01 | 0.20 | 0.00 | 0.20 | 0.08 | 0.05 | 0.08 | 0.06 | 500 |
| 7 | 3 | 500 | 250 | 2 | 0.5 | 1.5 | 2 | 2 | 100.0 | 100.0 | 100.0 | 100.0 | 0.00 | 0.20 | 0.00 | 0.20 | 0.08 | 0.05 | 0.08 | 0.05 | 500 |
| 7 | 3 | 500 | 250 | 2 | 0.5 | 2.5 | 0.5 | 1 | 100.0 | 100.0 | 100.0 | 100.0 | 0.01 | 0.19 | 0.01 | 0.19 | 0.08 | 0.06 | 0.08 | 0.05 | 500 |
| 7 | 3 | 500 | 250 | 2 | 0.5 | 2.5 | 0.5 | 2 | 100.0 | 100.0 | 100.0 | 100.0 | 0.00 | 0.19 | 0.00 | 0.19 | 0.08 | 0.05 | 0.08 | 0.05 | 500 |
| 7 | 3 | 500 | 500 | 0 | 0 | 1.5 | 2 | 1 | 3.4 | 3.4 | 3.4 | 3.4 | 0.00 | 0.00 | 0.00 | 0.00 | 0.07 | 0.05 | 0.07 | 0.05 | 500 |
| 7 | 3 | 500 | 500 | 0 | 0 | 1.5 | 2 | 2 | 5.2 | 5.2 | 5.4 | 5.2 | 0.00 | 0.00 | 0.00 | 0.00 | 0.07 | 0.05 | 0.07 | 0.05 | 500 |
| 7 | 3 | 500 | 500 | 0 | 0 | 2.5 | 0.5 | 1 | 5.4 | 5.4 | 5.4 | 5.4 | 0.01 | 0.01 | 0.01 | 0.01 | 0.08 | 0.05 | 0.08 | 0.05 | 500 |
| 7 | 3 | 500 | 500 | 0 | 0 | 2.5 | 0.5 | 2 | 5.4 | 5.4 | 5.4 | 5.4 | 0.00 | 0.00 | 0.00 | 0.00 | 0.08 | 0.06 | 0.08 | 0.06 | 500 |
| 7 | 3 | 500 | 500 | 0 | 0.2 | 1.5 | 2 | 1 | 78.4 | 78.4 | 78.4 | 78.4 | 0.00 | 0.05 | 0.00 | 0.05 | 0.07 | 0.05 | 0.07 | 0.05 | 500 |
| 7 | 3 | 500 | 500 | 0 | 0.2 | 1.5 | 2 | 2 | 73.8 | 73.6 | 73.8 | 73.6 | 0.00 | 0.06 | 0.00 | 0.06 | 0.07 | 0.05 | 0.07 | 0.05 | 500 |
| 7 | 3 | 500 | 500 | 0 | 0.2 | 2.5 | 0.5 | 1 | 74.8 | 74.8 | 74.8 | 74.8 | 0.00 | 0.06 | 0.00 | 0.06 | 0.08 | 0.06 | 0.08 | 0.06 | 500 |
| 7 | 3 | 500 | 500 | 0 | 0.2 | 2.5 | 0.5 | 2 | 76.2 | 76.0 | 76.2 | 76.0 | 0.00 | 0.06 | 0.00 | 0.06 | 0.08 | 0.06 | 0.08 | 0.06 | 500 |
| 7 | 3 | 500 | 500 | 0 | 0.5 | 1.5 | 2 | 1 | 100.0 | 100.0 | 100.0 | 100.0 | 0.00 | 0.13 | 0.00 | 0.13 | 0.08 | 0.06 | 0.08 | 0.06 | 500 |
| 7 | 3 | 500 | 500 | 0 | 0.5 | 1.5 | 2 | 2 | 100.0 | 100.0 | 100.0 | 100.0 | 0.00 | 0.13 | 0.00 | 0.13 | 0.07 | 0.06 | 0.07 | 0.06 | 500 |
| 7 | 3 | 500 | 500 | 0 | 0.5 | 2.5 | 0.5 | 1 | 100.0 | 100.0 | 100.0 | 100.0 | 0.00 | 0.14 | 0.00 | 0.14 | 0.08 | 0.06 | 0.08 | 0.06 | 500 |
| 7 | 3 | 500 | 500 | 0 | 0.5 | 2.5 | 0.5 | 2 | 100.0 | 100.0 | 100.0 | 100.0 | 0.00 | 0.14 | 0.00 | 0.14 | 0.08 | 0.06 | 0.08 | 0.06 | 500 |
| 7 | 3 | 500 | 500 | 0.5 | 0 | 1.5 | 2 | 1 | 5.8 | 5.8 | 5.8 | 5.8 | 0.00 | 0.00 | 0.00 | 0.00 | 0.08 | 0.05 | 0.08 | 0.05 | 500 |
| 7 | 3 | 500 | 500 | 0.5 | 0 | 1.5 | 2 | 2 | 4.8 | 4.8 | 4.8 | 4.8 | 0.00 | 0.00 | 0.00 | 0.00 | 0.07 | 0.05 | 0.07 | 0.05 | 500 |
| 7 | 3 | 500 | 500 | 0.5 | 0 | 2.5 | 0.5 | 1 | 4.0 | 4.0 | 4.0 | 4.0 | 0.00 | 0.00 | 0.00 | 0.00 | 0.07 | 0.05 | 0.07 | 0.05 | 500 |
| 7 | 3 | 500 | 500 | 0.5 | 0 | 2.5 | 0.5 | 2 | 4.6 | 4.6 | 4.6 | 4.6 | 0.00 | 0.00 | 0.00 | 0.00 | 0.08 | 0.05 | 0.08 | 0.05 | 500 |
| 7 | 3 | 500 | 500 | 0.5 | 0.2 | 1.5 | 2 | 1 | 74.8 | 74.8 | 74.8 | 74.6 | 0.00 | 0.06 | 0.00 | 0.06 | 0.08 | 0.06 | 0.08 | 0.06 | 500 |
| 7 | 3 | 500 | 500 | 0.5 | 0.2 | 1.5 | 2 | 2 | 75.6 | 75.6 | 75.6 | 75.6 | 0.00 | 0.05 | 0.00 | 0.05 | 0.07 | 0.05 | 0.07 | 0.05 | 500 |
| 7 | 3 | 500 | 500 | 0.5 | 0.2 | 2.5 | 0.5 | 1 | 78.8 | 78.6 | 78.8 | 78.8 | 0.01 | 0.05 | 0.01 | 0.05 | 0.07 | 0.05 | 0.07 | 0.05 | 500 |
| 7 | 3 | 500 | 500 | 0.5 | 0.2 | 2.5 | 0.5 | 2 | 74.6 | 74.4 | 74.8 | 74.2 | 0.00 | 0.06 | 0.00 | 0.06 | 0.08 | 0.05 | 0.08 | 0.05 | 500 |
| 7 | 3 | 500 | 500 | 0.5 | 0.5 | 1.5 | 2 | 1 | 100.0 | 100.0 | 100.0 | 100.0 | 0.01 | 0.13 | 0.01 | 0.13 | 0.07 | 0.06 | 0.07 | 0.06 | 500 |
| 7 | 3 | 500 | 500 | 0.5 | 0.5 | 1.5 | 2 | 2 | 100.0 | 100.0 | 100.0 | 100.0 | 0.00 | 0.13 | 0.00 | 0.13 | 0.07 | 0.06 | 0.07 | 0.06 | 500 |
| 7 | 3 | 500 | 500 | 0.5 | 0.5 | 2.5 | 0.5 | 1 | 100.0 | 100.0 | 100.0 | 100.0 | 0.00 | 0.14 | 0.00 | 0.14 | 0.07 | 0.05 | 0.07 | 0.06 | 500 |
| 7 | 3 | 500 | 500 | 0.5 | 0.5 | 2.5 | 0.5 | 2 | 100.0 | 100.0 | 100.0 | 100.0 | 0.00 | 0.14 | 0.00 | 0.14 | 0.08 | 0.06 | 0.08 | 0.06 | 500 |
| 7 | 3 | 500 | 500 | 2 | 0 | 1.5 | 2 | 1 | 6.6 | 6.2 | 6.6 | 6.4 | 0.00 | 0.00 | 0.00 | 0.00 | 0.08 | 0.05 | 0.09 | 0.05 | 500 |
| 7 | 3 | 500 | 500 | 2 | 0 | 1.5 | 2 | 2 | 3.4 | 3.4 | 3.4 | 3.4 | 0.00 | 0.00 | 0.00 | 0.00 | 0.08 | 0.05 | 0.08 | 0.05 | 500 |
| 7 | 3 | 500 | 500 | 2 | 0 | 2.5 | 0.5 | 1 | 3.8 | 3.8 | 3.8 | 3.8 | 0.01 | 0.00 | 0.01 | 0.00 | 0.08 | 0.05 | 0.08 | 0.05 | 500 |
| 7 | 3 | 500 | 500 | 2 | 0 | 2.5 | 0.5 | 2 | 6.4 | 6.4 | 6.4 | 6.4 | 0.00 | 0.00 | 0.00 | 0.00 | 0.08 | 0.05 | 0.08 | 0.05 | 500 |
| 7 | 3 | 500 | 500 | 2 | 0.2 | 1.5 | 2 | 1 | 63.0 | 62.8 | 63.0 | 62.8 | 0.01 | 0.09 | 0.01 | 0.09 | 0.09 | 0.05 | 0.09 | 0.05 | 500 |
| 7 | 3 | 500 | 500 | 2 | 0.2 | 1.5 | 2 | 2 | 71.8 | 71.6 | 72.0 | 72.0 | 0.01 | 0.08 | 0.01 | 0.08 | 0.08 | 0.05 | 0.08 | 0.05 | 500 |
| 7 | 3 | 500 | 500 | 2 | 0.2 | 2.5 | 0.5 | 1 | 70.4 | 70.4 | 70.4 | 70.4 | 0.01 | 0.08 | 0.01 | 0.08 | 0.08 | 0.05 | 0.08 | 0.05 | 500 |
| 7 | 3 | 500 | 500 | 2 | 0.2 | 2.5 | 0.5 | 2 | 65.2 | 64.8 | 65.0 | 64.8 | 0.01 | 0.08 | 0.01 | 0.08 | 0.08 | 0.05 | 0.08 | 0.05 | 500 |
| 7 | 3 | 500 | 500 | 2 | 0.5 | 1.5 | 2 | 1 | 100.0 | 100.0 | 100.0 | 100.0 | 0.00 | 0.20 | 0.00 | 0.20 | 0.08 | 0.05 | 0.08 | 0.05 | 500 |
| 7 | 3 | 500 | 500 | 2 | 0.5 | 1.5 | 2 | 2 | 100.0 | 100.0 | 100.0 | 100.0 | 0.00 | 0.20 | 0.01 | 0.20 | 0.08 | 0.05 | 0.08 | 0.05 | 500 |
| 7 | 3 | 500 | 500 | 2 | 0.5 | 2.5 | 0.5 | 1 | 100.0 | 100.0 | 100.0 | 100.0 | 0.00 | 0.19 | 0.00 | 0.19 | 0.08 | 0.06 | 0.08 | 0.06 | 500 |
| 7 | 3 | 500 | 500 | 2 | 0.5 | 2.5 | 0.5 | 2 | 100.0 | 100.0 | 100.0 | 100.0 | 0.00 | 0.19 | 0.00 | 0.19 | 0.08 | 0.06 | 0.08 | 0.06 | 500 |
| 7 | 5 | 50 | 100 | 0 | 0 | 1.5 | 2 | 1 | 6.3 | 5.6 | 6.2 | 6.0 | 0.01 | 0.01 | 0.01 | 0.01 | 0.23 | 0.19 | 0.23 | 0.19 | 477 |
| 7 | 5 | 50 | 100 | 0 | 0 | 1.5 | 2 | 2 | 4.6 | 4.4 | 4.8 | 4.4 | 0.01 | 0.01 | 0.01 | 0.01 | 0.22 | 0.18 | 0.22 | 0.19 | 500 |
| 7 | 5 | 50 | 100 | 0 | 0 | 2.5 | 0.5 | 1 | 5.5 | 5.2 | 5.8 | 5.2 | 0.01 | 0.00 | 0.01 | 0.01 | 0.23 | 0.19 | 0.23 | 0.19 | 493 |
| 7 | 5 | 50 | 100 | 0 | 0 | 2.5 | 0.5 | 2 | 5.8 | 4.8 | 5.6 | 5.0 | 0.01 | 0.01 | 0.01 | 0.01 | 0.23 | 0.19 | 0.23 | 0.19 | 500 |
| 7 | 5 | 50 | 100 | 0 | 0.2 | 1.5 | 2 | 1 | 15.9 | 14.4 | 15.9 | 14.4 | 0.00 | 0.03 | 0.00 | 0.03 | 0.22 | 0.19 | 0.22 | 0.19 | 472 |
| 7 | 5 | 50 | 100 | 0 | 0.2 | 1.5 | 2 | 2 | 15.0 | 14.8 | 15.2 | 14.6 | 0.01 | 0.02 | 0.01 | 0.02 | 0.22 | 0.19 | 0.22 | 0.19 | 500 |
| 7 | 5 | 50 | 100 | 0 | 0.2 | 2.5 | 0.5 | 1 | 14.8 | 13.8 | 15.0 | 13.6 | 0.00 | 0.03 | 0.00 | 0.03 | 0.23 | 0.19 | 0.23 | 0.19 | 486 |
| 7 | 5 | 50 | 100 | 0 | 0.2 | 2.5 | 0.5 | 2 | 14.8 | 13.6 | 15.4 | 13.4 | 0.00 | 0.04 | 0.00 | 0.04 | 0.22 | 0.18 | 0.22 | 0.18 | 500 |
| 7 | 5 | 50 | 100 | 0 | 0.5 | 1.5 | 2 | 1 | 68.9 | 68.9 | 68.9 | 68.9 | 0.02 | 0.06 | 0.02 | 0.06 | 0.22 | 0.19 | 0.22 | 0.19 | 483 |
| 7 | 5 | 50 | 100 | 0 | 0.5 | 1.5 | 2 | 2 | 65.6 | 64.8 | 65.2 | 64.2 | 0.02 | 0.06 | 0.02 | 0.05 | 0.21 | 0.19 | 0.22 | 0.19 | 500 |
| 7 | 5 | 50 | 100 | 0 | 0.5 | 2.5 | 0.5 | 1 | 58.8 | 58.8 | 58.8 | 58.8 | 0.00 | 0.08 | 0.00 | 0.09 | 0.24 | 0.20 | 0.23 | 0.20 | 488 |
| 7 | 5 | 50 | 100 | 0 | 0.5 | 2.5 | 0.5 | 2 | 58.9 | 58.4 | 59.2 | 58.6 | 0.01 | 0.09 | 0.00 | 0.09 | 0.23 | 0.19 | 0.23 | 0.19 | 499 |
| 7 | 5 | 50 | 100 | 0.5 | 0 | 1.5 | 2 | 1 | 5.0 | 4.8 | 4.8 | 4.8 | 0.01 | 0.01 | 0.01 | 0.01 | 0.22 | 0.19 | 0.22 | 0.19 | 476 |
| 7 | 5 | 50 | 100 | 0.5 | 0 | 1.5 | 2 | 2 | 4.8 | 4.6 | 4.8 | 4.4 | 0.00 | 0.00 | 0.00 | 0.00 | 0.23 | 0.20 | 0.23 | 0.20 | 498 |
| 7 | 5 | 50 | 100 | 0.5 | 0 | 2.5 | 0.5 | 1 | 6.3 | 6.0 | 6.2 | 5.6 | 0.00 | 0.00 | 0.00 | 0.00 | 0.23 | 0.19 | 0.24 | 0.20 | 494 |
| 7 | 5 | 50 | 100 | 0.5 | 0 | 2.5 | 0.5 | 2 | 6.4 | 6.0 | 6.6 | 6.0 | 0.00 | 0.00 | 0.00 | 0.00 | 0.22 | 0.18 | 0.23 | 0.19 | 500 |
| 7 | 5 | 50 | 100 | 0.5 | 0.2 | 1.5 | 2 | 1 | 15.8 | 14.4 | 15.2 | 14.2 | 0.01 | 0.04 | 0.01 | 0.04 | 0.23 | 0.19 | 0.23 | 0.19 | 480 |
| 7 | 5 | 50 | 100 | 0.5 | 0.2 | 1.5 | 2 | 2 | 15.4 | 15.0 | 15.4 | 15.0 | 0.00 | 0.03 | 0.00 | 0.03 | 0.22 | 0.19 | 0.22 | 0.19 | 499 |
| 7 | 5 | 50 | 100 | 0.5 | 0.2 | 2.5 | 0.5 | 1 | 18.3 | 18.0 | 18.4 | 17.8 | 0.01 | 0.03 | 0.01 | 0.03 | 0.25 | 0.20 | 0.25 | 0.20 | 493 |
| 7 | 5 | 50 | 100 | 0.5 | 0.2 | 2.5 | 0.5 | 2 | 16.0 | 15.6 | 16.2 | 15.6 | 0.01 | 0.03 | 0.01 | 0.03 | 0.23 | 0.19 | 0.23 | 0.19 | 500 |
| 7 | 5 | 50 | 100 | 0.5 | 0.5 | 1.5 | 2 | 1 | 62.6 | 61.2 | 62.6 | 61.2 | 0.02 | 0.06 | 0.02 | 0.05 | 0.23 | 0.20 | 0.23 | 0.20 | 478 |
| 7 | 5 | 50 | 100 | 0.5 | 0.5 | 1.5 | 2 | 2 | 61.4 | 60.8 | 61.4 | 60.6 | 0.00 | 0.07 | 0.01 | 0.07 | 0.23 | 0.20 | 0.23 | 0.20 | 500 |
| 7 | 5 | 50 | 100 | 0.5 | 0.5 | 2.5 | 0.5 | 1 | 60.2 | 58.0 | 60.4 | 58.4 | 0.01 | 0.08 | 0.01 | 0.08 | 0.23 | 0.19 | 0.23 | 0.19 | 490 |
| 7 | 5 | 50 | 100 | 0.5 | 0.5 | 2.5 | 0.5 | 2 | 60.4 | 58.8 | 60.2 | 58.8 | 0.00 | 0.08 | 0.01 | 0.08 | 0.23 | 0.20 | 0.23 | 0.20 | 500 |
| 7 | 5 | 50 | 100 | 2 | 0 | 1.5 | 2 | 1 | 4.5 | 4.2 | 4.8 | 4.4 | 0.00 | 0.00 | 0.00 | 0.00 | 0.23 | 0.17 | 0.24 | 0.18 | 487 |
| 7 | 5 | 50 | 100 | 2 | 0 | 1.5 | 2 | 2 | 5.6 | 5.6 | 5.8 | 5.6 | 0.01 | 0.01 | 0.01 | 0.01 | 0.24 | 0.17 | 0.24 | 0.18 | 499 |
| 7 | 5 | 50 | 100 | 2 | 0 | 2.5 | 0.5 | 1 | 5.5 | 5.0 | 5.4 | 5.2 | 0.01 | 0.01 | 0.01 | 0.01 | 0.24 | 0.18 | 0.25 | 0.19 | 489 |
| 7 | 5 | 50 | 100 | 2 | 0 | 2.5 | 0.5 | 2 | 6.2 | 6.0 | 6.4 | 6.0 | 0.01 | 0.01 | 0.01 | 0.01 | 0.24 | 0.18 | 0.25 | 0.19 | 500 |
| 7 | 5 | 50 | 100 | 2 | 0.2 | 1.5 | 2 | 1 | 15.9 | 14.4 | 15.6 | 14.0 | 0.01 | 0.05 | 0.01 | 0.04 | 0.24 | 0.18 | 0.25 | 0.19 | 477 |
| 7 | 5 | 50 | 100 | 2 | 0.2 | 1.5 | 2 | 2 | 17.8 | 17.0 | 18.6 | 17.2 | 0.03 | 0.03 | 0.03 | 0.03 | 0.25 | 0.18 | 0.25 | 0.19 | 500 |
| 7 | 5 | 50 | 100 | 2 | 0.2 | 2.5 | 0.5 | 1 | 15.6 | 14.6 | 15.6 | 15.0 | 0.01 | 0.04 | 0.02 | 0.03 | 0.24 | 0.18 | 0.25 | 0.19 | 494 |
| 7 | 5 | 50 | 100 | 2 | 0.2 | 2.5 | 0.5 | 2 | 16.4 | 15.8 | 16.2 | 16.0 | 0.01 | 0.04 | 0.02 | 0.03 | 0.24 | 0.18 | 0.25 | 0.19 | 499 |
| 7 | 5 | 50 | 100 | 2 | 0.5 | 1.5 | 2 | 1 | 56.3 | 56.3 | 56.3 | 56.3 | 0.01 | 0.12 | 0.03 | 0.10 | 0.24 | 0.18 | 0.25 | 0.19 | 476 |
| 7 | 5 | 50 | 100 | 2 | 0.5 | 1.5 | 2 | 2 | 54.8 | 53.6 | 55.6 | 53.4 | 0.01 | 0.13 | 0.02 | 0.11 | 0.24 | 0.18 | 0.24 | 0.19 | 500 |
| 7 | 5 | 50 | 100 | 2 | 0.5 | 2.5 | 0.5 | 1 | 60.1 | 60.1 | 60.1 | 60.1 | 0.01 | 0.11 | 0.03 | 0.09 | 0.25 | 0.20 | 0.25 | 0.20 | 494 |
| 7 | 5 | 50 | 100 | 2 | 0.5 | 2.5 | 0.5 | 2 | 59.7 | 58.2 | 60.4 | 58.2 | 0.02 | 0.10 | 0.04 | 0.08 | 0.25 | 0.20 | 0.25 | 0.20 | 499 |
| 7 | 5 | 50 | 250 | 0 | 0 | 1.5 | 2 | 1 | 5.6 | 5.4 | 5.6 | 5.2 | 0.01 | 0.01 | 0.01 | 0.01 | 0.23 | 0.19 | 0.22 | 0.19 | 500 |
| 7 | 5 | 50 | 250 | 0 | 0 | 1.5 | 2 | 2 | 6.6 | 5.8 | 6.6 | 6.2 | 0.01 | 0.01 | 0.01 | 0.00 | 0.22 | 0.19 | 0.22 | 0.19 | 500 |
| 7 | 5 | 50 | 250 | 0 | 0 | 2.5 | 0.5 | 1 | 4.4 | 4.2 | 4.4 | 4.0 | 0.02 | 0.01 | 0.02 | 0.01 | 0.22 | 0.18 | 0.22 | 0.18 | 500 |
| 7 | 5 | 50 | 250 | 0 | 0 | 2.5 | 0.5 | 2 | 6.8 | 6.0 | 7.2 | 6.2 | 0.01 | 0.01 | 0.01 | 0.01 | 0.23 | 0.19 | 0.23 | 0.19 | 500 |
| 7 | 5 | 50 | 250 | 0 | 0.2 | 1.5 | 2 | 1 | 14.2 | 13.4 | 14.8 | 13.6 | 0.00 | 0.03 | 0.00 | 0.03 | 0.22 | 0.18 | 0.21 | 0.18 | 500 |
| 7 | 5 | 50 | 250 | 0 | 0.2 | 1.5 | 2 | 2 | 15.0 | 13.6 | 15.4 | 13.6 | 0.01 | 0.02 | 0.00 | 0.03 | 0.22 | 0.19 | 0.22 | 0.19 | 500 |
| 7 | 5 | 50 | 250 | 0 | 0.2 | 2.5 | 0.5 | 1 | 14.0 | 13.2 | 14.2 | 13.2 | 0.00 | 0.03 | 0.00 | 0.04 | 0.22 | 0.18 | 0.21 | 0.18 | 500 |
| 7 | 5 | 50 | 250 | 0 | 0.2 | 2.5 | 0.5 | 2 | 13.6 | 13.0 | 14.4 | 13.2 | 0.01 | 0.04 | 0.01 | 0.04 | 0.22 | 0.18 | 0.22 | 0.18 | 500 |
| 7 | 5 | 50 | 250 | 0 | 0.5 | 1.5 | 2 | 1 | 64.0 | 63.4 | 64.4 | 63.4 | 0.00 | 0.07 | 0.00 | 0.07 | 0.22 | 0.19 | 0.22 | 0.19 | 500 |
| 7 | 5 | 50 | 250 | 0 | 0.5 | 1.5 | 2 | 2 | 63.8 | 63.0 | 64.2 | 63.2 | 0.02 | 0.06 | 0.01 | 0.07 | 0.22 | 0.19 | 0.22 | 0.19 | 500 |
| 7 | 5 | 50 | 250 | 0 | 0.5 | 2.5 | 0.5 | 1 | 58.6 | 57.4 | 58.6 | 57.0 | 0.01 | 0.09 | 0.01 | 0.09 | 0.23 | 0.20 | 0.23 | 0.19 | 500 |
| 7 | 5 | 50 | 250 | 0 | 0.5 | 2.5 | 0.5 | 2 | 61.2 | 59.6 | 61.2 | 59.2 | 0.00 | 0.08 | 0.00 | 0.09 | 0.23 | 0.19 | 0.22 | 0.19 | 500 |
| 7 | 5 | 50 | 250 | 0.5 | 0 | 1.5 | 2 | 1 | 6.2 | 6.0 | 6.0 | 6.0 | 0.01 | 0.01 | 0.01 | 0.00 | 0.23 | 0.20 | 0.23 | 0.19 | 500 |
| 7 | 5 | 50 | 250 | 0.5 | 0 | 1.5 | 2 | 2 | 5.2 | 5.2 | 5.4 | 5.0 | 0.01 | 0.01 | 0.01 | 0.01 | 0.22 | 0.19 | 0.22 | 0.18 | 500 |
| 7 | 5 | 50 | 250 | 0.5 | 0 | 2.5 | 0.5 | 1 | 5.2 | 4.8 | 5.8 | 5.0 | 0.00 | 0.00 | 0.00 | 0.00 | 0.23 | 0.19 | 0.23 | 0.19 | 500 |
| 7 | 5 | 50 | 250 | 0.5 | 0 | 2.5 | 0.5 | 2 | 6.0 | 5.4 | 6.0 | 5.4 | 0.01 | 0.01 | 0.01 | 0.00 | 0.23 | 0.19 | 0.23 | 0.19 | 500 |
| 7 | 5 | 50 | 250 | 0.5 | 0.2 | 1.5 | 2 | 1 | 14.2 | 13.6 | 14.0 | 13.2 | 0.01 | 0.04 | 0.01 | 0.04 | 0.24 | 0.20 | 0.24 | 0.20 | 500 |
| 7 | 5 | 50 | 250 | 0.5 | 0.2 | 1.5 | 2 | 2 | 16.2 | 15.4 | 16.6 | 15.6 | 0.01 | 0.03 | 0.01 | 0.03 | 0.22 | 0.19 | 0.22 | 0.19 | 500 |
| 7 | 5 | 50 | 250 | 0.5 | 0.2 | 2.5 | 0.5 | 1 | 17.2 | 16.6 | 17.0 | 16.6 | 0.01 | 0.03 | 0.01 | 0.03 | 0.24 | 0.20 | 0.24 | 0.19 | 500 |
| 7 | 5 | 50 | 250 | 0.5 | 0.2 | 2.5 | 0.5 | 2 | 14.8 | 14.0 | 15.2 | 14.0 | 0.00 | 0.03 | 0.00 | 0.04 | 0.22 | 0.18 | 0.22 | 0.18 | 500 |
| 7 | 5 | 50 | 250 | 0.5 | 0.5 | 1.5 | 2 | 1 | 60.4 | 59.2 | 60.6 | 59.2 | 0.00 | 0.07 | 0.00 | 0.07 | 0.23 | 0.20 | 0.23 | 0.20 | 500 |
| 7 | 5 | 50 | 250 | 0.5 | 0.5 | 1.5 | 2 | 2 | 60.8 | 60.2 | 60.6 | 60.4 | 0.01 | 0.07 | 0.00 | 0.08 | 0.24 | 0.21 | 0.24 | 0.20 | 500 |
| 7 | 5 | 50 | 250 | 0.5 | 0.5 | 2.5 | 0.5 | 1 | 65.8 | 63.8 | 66.6 | 64.2 | 0.03 | 0.07 | 0.02 | 0.07 | 0.22 | 0.19 | 0.22 | 0.19 | 500 |
| 7 | 5 | 50 | 250 | 0.5 | 0.5 | 2.5 | 0.5 | 2 | 62.6 | 61.2 | 63.4 | 60.8 | 0.01 | 0.08 | 0.00 | 0.09 | 0.22 | 0.19 | 0.22 | 0.18 | 500 |
| 7 | 5 | 50 | 250 | 2 | 0 | 1.5 | 2 | 1 | 4.2 | 4.0 | 4.2 | 3.8 | 0.00 | 0.00 | 0.00 | 0.00 | 0.24 | 0.18 | 0.24 | 0.18 | 500 |
| 7 | 5 | 50 | 250 | 2 | 0 | 1.5 | 2 | 2 | 5.8 | 5.8 | 5.8 | 5.6 | 0.00 | 0.00 | 0.00 | 0.00 | 0.24 | 0.18 | 0.24 | 0.18 | 500 |
| 7 | 5 | 50 | 250 | 2 | 0 | 2.5 | 0.5 | 1 | 3.6 | 3.0 | 4.0 | 2.8 | 0.01 | 0.01 | 0.01 | 0.01 | 0.23 | 0.17 | 0.23 | 0.17 | 500 |
| 7 | 5 | 50 | 250 | 2 | 0 | 2.5 | 0.5 | 2 | 6.0 | 5.4 | 6.4 | 5.2 | 0.02 | 0.01 | 0.02 | 0.01 | 0.24 | 0.18 | 0.24 | 0.18 | 500 |
| 7 | 5 | 50 | 250 | 2 | 0.2 | 1.5 | 2 | 1 | 12.6 | 12.4 | 13.2 | 12.6 | 0.00 | 0.06 | 0.00 | 0.05 | 0.24 | 0.18 | 0.24 | 0.18 | 500 |
| 7 | 5 | 50 | 250 | 2 | 0.2 | 1.5 | 2 | 2 | 13.0 | 12.2 | 13.2 | 12.4 | 0.00 | 0.05 | 0.00 | 0.05 | 0.24 | 0.18 | 0.24 | 0.18 | 500 |
| 7 | 5 | 50 | 250 | 2 | 0.2 | 2.5 | 0.5 | 1 | 13.8 | 13.0 | 14.4 | 13.0 | 0.00 | 0.05 | 0.01 | 0.04 | 0.24 | 0.18 | 0.24 | 0.19 | 500 |
| 7 | 5 | 50 | 250 | 2 | 0.2 | 2.5 | 0.5 | 2 | 15.8 | 14.8 | 16.0 | 15.0 | 0.01 | 0.04 | 0.01 | 0.04 | 0.23 | 0.18 | 0.23 | 0.18 | 500 |
| 7 | 5 | 50 | 250 | 2 | 0.5 | 1.5 | 2 | 1 | 54.4 | 53.4 | 55.0 | 53.4 | 0.02 | 0.12 | 0.02 | 0.11 | 0.25 | 0.19 | 0.25 | 0.19 | 500 |
| 7 | 5 | 50 | 250 | 2 | 0.5 | 1.5 | 2 | 2 | 57.0 | 55.2 | 58.2 | 55.6 | 0.00 | 0.13 | 0.01 | 0.12 | 0.24 | 0.18 | 0.24 | 0.18 | 500 |
| 7 | 5 | 50 | 250 | 2 | 0.5 | 2.5 | 0.5 | 1 | 57.2 | 56.8 | 57.4 | 57.0 | 0.01 | 0.12 | 0.00 | 0.12 | 0.24 | 0.19 | 0.24 | 0.19 | 500 |
| 7 | 5 | 50 | 250 | 2 | 0.5 | 2.5 | 0.5 | 2 | 56.4 | 54.8 | 57.0 | 55.2 | 0.01 | 0.11 | 0.01 | 0.11 | 0.23 | 0.19 | 0.23 | 0.18 | 500 |
| 7 | 5 | 50 | 500 | 0 | 0 | 1.5 | 2 | 1 | 5.6 | 5.4 | 5.6 | 5.4 | 0.01 | 0.00 | 0.00 | 0.00 | 0.23 | 0.20 | 0.23 | 0.19 | 500 |
| 7 | 5 | 50 | 500 | 0 | 0 | 1.5 | 2 | 2 | 5.6 | 5.4 | 6.0 | 5.4 | 0.01 | 0.01 | 0.01 | 0.01 | 0.22 | 0.19 | 0.22 | 0.19 | 500 |
| 7 | 5 | 50 | 500 | 0 | 0 | 2.5 | 0.5 | 1 | 5.6 | 5.2 | 5.6 | 5.4 | 0.01 | 0.01 | 0.01 | 0.00 | 0.23 | 0.19 | 0.23 | 0.19 | 500 |
| 7 | 5 | 50 | 500 | 0 | 0 | 2.5 | 0.5 | 2 | 6.6 | 6.2 | 6.6 | 6.4 | 0.01 | 0.01 | 0.01 | 0.01 | 0.23 | 0.19 | 0.23 | 0.19 | 500 |
| 7 | 5 | 50 | 500 | 0 | 0.2 | 1.5 | 2 | 1 | 17.6 | 16.8 | 17.6 | 16.8 | 0.01 | 0.02 | 0.01 | 0.03 | 0.23 | 0.20 | 0.23 | 0.19 | 500 |
| 7 | 5 | 50 | 500 | 0 | 0.2 | 1.5 | 2 | 2 | 11.6 | 11.2 | 12.2 | 11.0 | 0.01 | 0.04 | 0.01 | 0.04 | 0.21 | 0.18 | 0.20 | 0.17 | 500 |
| 7 | 5 | 50 | 500 | 0 | 0.2 | 2.5 | 0.5 | 1 | 15.8 | 15.0 | 16.2 | 15.2 | 0.00 | 0.03 | 0.00 | 0.03 | 0.23 | 0.19 | 0.23 | 0.19 | 500 |
| 7 | 5 | 50 | 500 | 0 | 0.2 | 2.5 | 0.5 | 2 | 15.8 | 15.8 | 16.0 | 15.8 | 0.01 | 0.03 | 0.01 | 0.03 | 0.23 | 0.19 | 0.22 | 0.18 | 500 |
| 7 | 5 | 50 | 500 | 0 | 0.5 | 1.5 | 2 | 1 | 63.4 | 61.4 | 63.6 | 61.8 | 0.02 | 0.05 | 0.01 | 0.06 | 0.23 | 0.20 | 0.22 | 0.19 | 500 |
| 7 | 5 | 50 | 500 | 0 | 0.5 | 1.5 | 2 | 2 | 61.8 | 59.8 | 61.8 | 60.2 | 0.00 | 0.08 | 0.01 | 0.08 | 0.22 | 0.19 | 0.21 | 0.19 | 500 |
| 7 | 5 | 50 | 500 | 0 | 0.5 | 2.5 | 0.5 | 1 | 63.6 | 62.0 | 63.4 | 62.6 | 0.01 | 0.07 | 0.00 | 0.08 | 0.23 | 0.20 | 0.22 | 0.19 | 500 |
| 7 | 5 | 50 | 500 | 0 | 0.5 | 2.5 | 0.5 | 2 | 62.6 | 61.8 | 62.8 | 61.8 | 0.01 | 0.08 | 0.00 | 0.09 | 0.23 | 0.20 | 0.22 | 0.19 | 500 |
| 7 | 5 | 50 | 500 | 0.5 | 0 | 1.5 | 2 | 1 | 6.0 | 5.8 | 6.0 | 5.8 | 0.02 | 0.02 | 0.02 | 0.02 | 0.23 | 0.19 | 0.22 | 0.18 | 500 |
| 7 | 5 | 50 | 500 | 0.5 | 0 | 1.5 | 2 | 2 | 5.4 | 5.0 | 5.4 | 5.2 | 0.01 | 0.01 | 0.01 | 0.01 | 0.22 | 0.18 | 0.21 | 0.18 | 500 |
| 7 | 5 | 50 | 500 | 0.5 | 0 | 2.5 | 0.5 | 1 | 6.4 | 6.0 | 6.6 | 6.2 | 0.02 | 0.01 | 0.02 | 0.01 | 0.23 | 0.19 | 0.23 | 0.19 | 500 |
| 7 | 5 | 50 | 500 | 0.5 | 0 | 2.5 | 0.5 | 2 | 6.4 | 6.2 | 6.4 | 6.0 | 0.01 | 0.01 | 0.01 | 0.01 | 0.24 | 0.19 | 0.23 | 0.19 | 500 |
| 7 | 5 | 50 | 500 | 0.5 | 0.2 | 1.5 | 2 | 1 | 15.4 | 14.6 | 15.4 | 14.6 | 0.00 | 0.03 | 0.00 | 0.04 | 0.22 | 0.19 | 0.22 | 0.18 | 500 |
| 7 | 5 | 50 | 500 | 0.5 | 0.2 | 1.5 | 2 | 2 | 16.2 | 16.0 | 16.6 | 15.6 | 0.00 | 0.03 | 0.00 | 0.03 | 0.23 | 0.20 | 0.23 | 0.19 | 500 |
| 7 | 5 | 50 | 500 | 0.5 | 0.2 | 2.5 | 0.5 | 1 | 16.6 | 15.6 | 17.0 | 15.8 | 0.00 | 0.04 | 0.00 | 0.04 | 0.24 | 0.20 | 0.23 | 0.19 | 500 |
| 7 | 5 | 50 | 500 | 0.5 | 0.2 | 2.5 | 0.5 | 2 | 13.6 | 13.2 | 13.6 | 13.4 | 0.00 | 0.04 | 0.01 | 0.04 | 0.22 | 0.18 | 0.22 | 0.18 | 500 |
| 7 | 5 | 50 | 500 | 0.5 | 0.5 | 1.5 | 2 | 1 | 61.6 | 60.6 | 62.6 | 60.4 | 0.01 | 0.07 | 0.00 | 0.08 | 0.23 | 0.20 | 0.22 | 0.19 | 500 |
| 7 | 5 | 50 | 500 | 0.5 | 0.5 | 1.5 | 2 | 2 | 66.4 | 65.0 | 66.6 | 65.0 | 0.01 | 0.07 | 0.00 | 0.07 | 0.24 | 0.21 | 0.23 | 0.20 | 500 |
| 7 | 5 | 50 | 500 | 0.5 | 0.5 | 2.5 | 0.5 | 1 | 64.2 | 63.6 | 64.6 | 63.2 | 0.02 | 0.07 | 0.01 | 0.08 | 0.23 | 0.20 | 0.23 | 0.19 | 500 |
| 7 | 5 | 50 | 500 | 0.5 | 0.5 | 2.5 | 0.5 | 2 | 65.2 | 64.2 | 65.4 | 64.0 | 0.02 | 0.07 | 0.02 | 0.07 | 0.23 | 0.20 | 0.23 | 0.19 | 500 |
| 7 | 5 | 50 | 500 | 2 | 0 | 1.5 | 2 | 1 | 4.6 | 4.2 | 4.6 | 4.2 | 0.02 | 0.01 | 0.02 | 0.01 | 0.24 | 0.17 | 0.24 | 0.17 | 500 |
| 7 | 5 | 50 | 500 | 2 | 0 | 1.5 | 2 | 2 | 4.8 | 4.4 | 5.6 | 4.2 | 0.01 | 0.00 | 0.01 | 0.00 | 0.24 | 0.17 | 0.24 | 0.17 | 500 |
| 7 | 5 | 50 | 500 | 2 | 0 | 2.5 | 0.5 | 1 | 6.4 | 6.4 | 6.4 | 6.4 | 0.01 | 0.01 | 0.01 | 0.01 | 0.24 | 0.18 | 0.24 | 0.18 | 500 |
| 7 | 5 | 50 | 500 | 2 | 0 | 2.5 | 0.5 | 2 | 5.0 | 4.6 | 5.2 | 4.6 | 0.00 | 0.00 | 0.00 | 0.00 | 0.23 | 0.17 | 0.23 | 0.17 | 500 |
| 7 | 5 | 50 | 500 | 2 | 0.2 | 1.5 | 2 | 1 | 16.2 | 15.0 | 16.6 | 15.2 | 0.00 | 0.06 | 0.00 | 0.06 | 0.24 | 0.18 | 0.24 | 0.18 | 500 |
| 7 | 5 | 50 | 500 | 2 | 0.2 | 1.5 | 2 | 2 | 14.8 | 14.2 | 15.0 | 14.6 | 0.01 | 0.05 | 0.01 | 0.04 | 0.25 | 0.19 | 0.25 | 0.19 | 500 |
| 7 | 5 | 50 | 500 | 2 | 0.2 | 2.5 | 0.5 | 1 | 11.2 | 10.6 | 11.6 | 11.0 | 0.00 | 0.05 | 0.00 | 0.05 | 0.23 | 0.17 | 0.23 | 0.17 | 500 |
| 7 | 5 | 50 | 500 | 2 | 0.2 | 2.5 | 0.5 | 2 | 15.8 | 15.4 | 16.6 | 15.4 | 0.02 | 0.04 | 0.02 | 0.03 | 0.24 | 0.18 | 0.24 | 0.18 | 500 |
| 7 | 5 | 50 | 500 | 2 | 0.5 | 1.5 | 2 | 1 | 59.0 | 58.0 | 59.2 | 57.6 | 0.02 | 0.11 | 0.02 | 0.11 | 0.24 | 0.19 | 0.24 | 0.19 | 500 |
| 7 | 5 | 50 | 500 | 2 | 0.5 | 1.5 | 2 | 2 | 54.0 | 52.8 | 54.2 | 52.6 | 0.00 | 0.13 | 0.00 | 0.13 | 0.25 | 0.19 | 0.25 | 0.19 | 500 |
| 7 | 5 | 50 | 500 | 2 | 0.5 | 2.5 | 0.5 | 1 | 59.0 | 57.2 | 59.2 | 57.0 | 0.02 | 0.10 | 0.02 | 0.10 | 0.24 | 0.20 | 0.24 | 0.20 | 500 |
| 7 | 5 | 50 | 500 | 2 | 0.5 | 2.5 | 0.5 | 2 | 57.0 | 55.8 | 57.2 | 55.8 | 0.01 | 0.11 | 0.01 | 0.11 | 0.24 | 0.19 | 0.24 | 0.19 | 500 |
| 7 | 5 | 100 | 100 | 0 | 0 | 1.5 | 2 | 1 | 5.4 | 5.2 | 5.2 | 5.0 | 0.00 | 0.00 | 0.00 | 0.00 | 0.15 | 0.13 | 0.15 | 0.13 | 480 |
| 7 | 5 | 100 | 100 | 0 | 0 | 1.5 | 2 | 2 | 7.2 | 7.2 | 7.2 | 7.0 | 0.01 | 0.01 | 0.01 | 0.01 | 0.16 | 0.13 | 0.16 | 0.14 | 500 |
| 7 | 5 | 100 | 100 | 0 | 0 | 2.5 | 0.5 | 1 | 3.3 | 3.0 | 3.2 | 3.0 | 0.00 | 0.00 | 0.00 | 0.00 | 0.15 | 0.13 | 0.16 | 0.13 | 489 |
| 7 | 5 | 100 | 100 | 0 | 0 | 2.5 | 0.5 | 2 | 3.6 | 3.6 | 3.6 | 3.6 | 0.00 | 0.00 | 0.01 | 0.00 | 0.15 | 0.12 | 0.15 | 0.13 | 500 |
| 7 | 5 | 100 | 100 | 0 | 0.2 | 1.5 | 2 | 1 | 26.2 | 24.8 | 25.0 | 24.6 | 0.00 | 0.03 | 0.00 | 0.03 | 0.16 | 0.14 | 0.16 | 0.14 | 478 |
| 7 | 5 | 100 | 100 | 0 | 0.2 | 1.5 | 2 | 2 | 23.1 | 22.8 | 23.8 | 23.0 | 0.00 | 0.03 | 0.00 | 0.03 | 0.15 | 0.13 | 0.15 | 0.13 | 499 |
| 7 | 5 | 100 | 100 | 0 | 0.2 | 2.5 | 0.5 | 1 | 27.9 | 26.4 | 27.4 | 26.6 | 0.00 | 0.03 | 0.00 | 0.03 | 0.17 | 0.14 | 0.17 | 0.14 | 491 |
| 7 | 5 | 100 | 100 | 0 | 0.2 | 2.5 | 0.5 | 2 | 26.6 | 26.0 | 27.0 | 26.2 | 0.00 | 0.03 | 0.01 | 0.03 | 0.16 | 0.13 | 0.16 | 0.14 | 500 |
| 7 | 5 | 100 | 100 | 0 | 0.5 | 1.5 | 2 | 1 | 91.5 | 91.5 | 91.5 | 91.5 | 0.02 | 0.06 | 0.02 | 0.05 | 0.16 | 0.14 | 0.16 | 0.14 | 480 |
| 7 | 5 | 100 | 100 | 0 | 0.5 | 1.5 | 2 | 2 | 91.0 | 90.4 | 90.4 | 90.2 | 0.01 | 0.07 | 0.01 | 0.07 | 0.15 | 0.13 | 0.16 | 0.14 | 498 |
| 7 | 5 | 100 | 100 | 0 | 0.5 | 2.5 | 0.5 | 1 | 89.6 | 88.0 | 88.0 | 88.0 | 0.00 | 0.08 | 0.01 | 0.08 | 0.16 | 0.14 | 0.16 | 0.14 | 492 |
| 7 | 5 | 100 | 100 | 0 | 0.5 | 2.5 | 0.5 | 2 | 87.6 | 87.2 | 87.6 | 87.2 | 0.00 | 0.08 | 0.01 | 0.08 | 0.16 | 0.14 | 0.17 | 0.14 | 500 |
| 7 | 5 | 100 | 100 | 0.5 | 0 | 1.5 | 2 | 1 | 6.2 | 5.6 | 5.8 | 5.6 | 0.00 | 0.00 | 0.00 | 0.00 | 0.16 | 0.13 | 0.16 | 0.14 | 482 |
| 7 | 5 | 100 | 100 | 0.5 | 0 | 1.5 | 2 | 2 | 5.6 | 5.6 | 6.0 | 5.8 | 0.01 | 0.01 | 0.01 | 0.01 | 0.16 | 0.13 | 0.16 | 0.13 | 500 |
| 7 | 5 | 100 | 100 | 0.5 | 0 | 2.5 | 0.5 | 1 | 5.5 | 5.4 | 5.4 | 5.2 | 0.00 | 0.00 | 0.00 | 0.00 | 0.16 | 0.13 | 0.16 | 0.13 | 495 |
| 7 | 5 | 100 | 100 | 0.5 | 0 | 2.5 | 0.5 | 2 | 5.8 | 5.8 | 6.0 | 5.8 | 0.00 | 0.00 | 0.00 | 0.00 | 0.16 | 0.13 | 0.17 | 0.14 | 500 |
| 7 | 5 | 100 | 100 | 0.5 | 0.2 | 1.5 | 2 | 1 | 27.8 | 27.8 | 27.8 | 27.8 | 0.00 | 0.03 | 0.01 | 0.03 | 0.16 | 0.14 | 0.17 | 0.14 | 478 |
| 7 | 5 | 100 | 100 | 0.5 | 0.2 | 1.5 | 2 | 2 | 25.0 | 24.6 | 24.8 | 24.6 | 0.00 | 0.03 | 0.00 | 0.03 | 0.15 | 0.13 | 0.15 | 0.13 | 500 |
| 7 | 5 | 100 | 100 | 0.5 | 0.2 | 2.5 | 0.5 | 1 | 23.5 | 23.0 | 23.0 | 22.8 | 0.00 | 0.04 | 0.00 | 0.03 | 0.15 | 0.13 | 0.16 | 0.13 | 494 |
| 7 | 5 | 100 | 100 | 0.5 | 0.2 | 2.5 | 0.5 | 2 | 22.4 | 22.2 | 23.0 | 22.4 | 0.01 | 0.04 | 0.01 | 0.04 | 0.16 | 0.13 | 0.16 | 0.13 | 500 |
| 7 | 5 | 100 | 100 | 0.5 | 0.5 | 1.5 | 2 | 1 | 92.7 | 92.7 | 92.7 | 92.7 | 0.02 | 0.06 | 0.03 | 0.05 | 0.15 | 0.13 | 0.16 | 0.14 | 478 |
| 7 | 5 | 100 | 100 | 0.5 | 0.5 | 1.5 | 2 | 2 | 88.6 | 87.6 | 87.8 | 87.6 | 0.00 | 0.08 | 0.01 | 0.07 | 0.16 | 0.14 | 0.16 | 0.14 | 498 |
| 7 | 5 | 100 | 100 | 0.5 | 0.5 | 2.5 | 0.5 | 1 | 90.1 | 90.1 | 90.1 | 90.1 | 0.02 | 0.07 | 0.02 | 0.07 | 0.16 | 0.13 | 0.16 | 0.14 | 493 |
| 7 | 5 | 100 | 100 | 0.5 | 0.5 | 2.5 | 0.5 | 2 | 88.6 | 88.4 | 88.8 | 87.8 | 0.00 | 0.08 | 0.01 | 0.08 | 0.16 | 0.14 | 0.16 | 0.14 | 500 |
| 7 | 5 | 100 | 100 | 2 | 0 | 1.5 | 2 | 1 | 6.1 | 5.4 | 5.8 | 5.4 | 0.01 | 0.01 | 0.01 | 0.01 | 0.17 | 0.13 | 0.18 | 0.13 | 475 |
| 7 | 5 | 100 | 100 | 2 | 0 | 1.5 | 2 | 2 | 4.4 | 4.4 | 4.4 | 4.2 | 0.01 | 0.01 | 0.01 | 0.01 | 0.17 | 0.12 | 0.17 | 0.13 | 500 |
| 7 | 5 | 100 | 100 | 2 | 0 | 2.5 | 0.5 | 1 | 3.8 | 3.6 | 3.8 | 3.6 | 0.00 | 0.00 | 0.00 | 0.00 | 0.16 | 0.12 | 0.17 | 0.13 | 496 |
| 7 | 5 | 100 | 100 | 2 | 0 | 2.5 | 0.5 | 2 | 3.4 | 3.4 | 3.4 | 3.4 | 0.00 | 0.00 | 0.00 | 0.00 | 0.16 | 0.12 | 0.16 | 0.12 | 500 |
| 7 | 5 | 100 | 100 | 2 | 0.2 | 1.5 | 2 | 1 | 21.9 | 20.6 | 21.4 | 21.0 | 0.00 | 0.05 | 0.01 | 0.05 | 0.18 | 0.13 | 0.18 | 0.14 | 475 |
| 7 | 5 | 100 | 100 | 2 | 0.2 | 1.5 | 2 | 2 | 19.8 | 19.6 | 20.4 | 19.8 | 0.00 | 0.05 | 0.01 | 0.05 | 0.17 | 0.12 | 0.17 | 0.13 | 500 |
| 7 | 5 | 100 | 100 | 2 | 0.2 | 2.5 | 0.5 | 1 | 25.2 | 24.8 | 24.8 | 24.4 | 0.01 | 0.04 | 0.02 | 0.03 | 0.17 | 0.13 | 0.17 | 0.13 | 493 |
| 7 | 5 | 100 | 100 | 2 | 0.2 | 2.5 | 0.5 | 2 | 25.2 | 24.4 | 25.4 | 24.2 | 0.01 | 0.04 | 0.02 | 0.04 | 0.17 | 0.13 | 0.17 | 0.13 | 500 |
| 7 | 5 | 100 | 100 | 2 | 0.5 | 1.5 | 2 | 1 | 83.4 | 80.0 | 80.4 | 80.0 | 0.00 | 0.13 | 0.01 | 0.12 | 0.17 | 0.13 | 0.18 | 0.14 | 481 |
| 7 | 5 | 100 | 100 | 2 | 0.5 | 1.5 | 2 | 2 | 86.0 | 85.6 | 86.2 | 86.0 | 0.01 | 0.12 | 0.02 | 0.11 | 0.17 | 0.13 | 0.18 | 0.14 | 500 |
| 7 | 5 | 100 | 100 | 2 | 0.5 | 2.5 | 0.5 | 1 | 86.5 | 86.5 | 86.5 | 86.5 | 0.01 | 0.11 | 0.03 | 0.09 | 0.17 | 0.14 | 0.18 | 0.14 | 489 |
| 7 | 5 | 100 | 100 | 2 | 0.5 | 2.5 | 0.5 | 2 | 86.8 | 86.4 | 87.0 | 86.4 | 0.00 | 0.12 | 0.02 | 0.10 | 0.16 | 0.13 | 0.17 | 0.14 | 500 |
| 7 | 5 | 100 | 250 | 0 | 0 | 1.5 | 2 | 1 | 4.8 | 4.8 | 5.2 | 4.6 | 0.01 | 0.00 | 0.01 | 0.00 | 0.16 | 0.13 | 0.15 | 0.13 | 499 |
| 7 | 5 | 100 | 250 | 0 | 0 | 1.5 | 2 | 2 | 5.4 | 4.8 | 5.6 | 5.0 | 0.01 | 0.01 | 0.01 | 0.01 | 0.16 | 0.13 | 0.16 | 0.13 | 500 |
| 7 | 5 | 100 | 250 | 0 | 0 | 2.5 | 0.5 | 1 | 5.4 | 5.4 | 5.4 | 5.2 | 0.00 | 0.00 | 0.00 | 0.00 | 0.15 | 0.13 | 0.15 | 0.13 | 500 |
| 7 | 5 | 100 | 250 | 0 | 0 | 2.5 | 0.5 | 2 | 4.6 | 4.6 | 4.6 | 4.6 | 0.00 | 0.00 | 0.00 | 0.00 | 0.16 | 0.13 | 0.16 | 0.13 | 500 |
| 7 | 5 | 100 | 250 | 0 | 0.2 | 1.5 | 2 | 1 | 26.8 | 25.8 | 27.0 | 26.2 | 0.01 | 0.02 | 0.01 | 0.02 | 0.16 | 0.13 | 0.16 | 0.13 | 500 |
| 7 | 5 | 100 | 250 | 0 | 0.2 | 1.5 | 2 | 2 | 28.2 | 27.6 | 28.2 | 27.6 | 0.00 | 0.03 | 0.00 | 0.03 | 0.16 | 0.14 | 0.16 | 0.14 | 500 |
| 7 | 5 | 100 | 250 | 0 | 0.2 | 2.5 | 0.5 | 1 | 23.6 | 23.6 | 23.6 | 23.2 | 0.00 | 0.04 | 0.00 | 0.04 | 0.16 | 0.13 | 0.16 | 0.13 | 500 |
| 7 | 5 | 100 | 250 | 0 | 0.2 | 2.5 | 0.5 | 2 | 24.4 | 24.2 | 24.2 | 24.0 | 0.01 | 0.03 | 0.01 | 0.03 | 0.15 | 0.12 | 0.15 | 0.12 | 500 |
| 7 | 5 | 100 | 250 | 0 | 0.5 | 1.5 | 2 | 1 | 91.0 | 90.8 | 91.0 | 90.8 | 0.00 | 0.07 | 0.00 | 0.07 | 0.15 | 0.14 | 0.15 | 0.13 | 500 |
| 7 | 5 | 100 | 250 | 0 | 0.5 | 1.5 | 2 | 2 | 91.6 | 91.4 | 91.4 | 91.0 | 0.01 | 0.06 | 0.01 | 0.06 | 0.16 | 0.14 | 0.16 | 0.14 | 500 |
| 7 | 5 | 100 | 250 | 0 | 0.5 | 2.5 | 0.5 | 1 | 87.2 | 87.2 | 87.4 | 87.2 | 0.00 | 0.09 | 0.00 | 0.09 | 0.16 | 0.13 | 0.16 | 0.13 | 500 |
| 7 | 5 | 100 | 250 | 0 | 0.5 | 2.5 | 0.5 | 2 | 89.6 | 89.2 | 89.6 | 89.2 | 0.01 | 0.08 | 0.01 | 0.08 | 0.16 | 0.14 | 0.16 | 0.14 | 500 |
| 7 | 5 | 100 | 250 | 0.5 | 0 | 1.5 | 2 | 1 | 3.8 | 3.8 | 3.8 | 3.6 | 0.00 | 0.00 | 0.00 | 0.00 | 0.15 | 0.13 | 0.15 | 0.13 | 499 |
| 7 | 5 | 100 | 250 | 0.5 | 0 | 1.5 | 2 | 2 | 3.8 | 3.8 | 3.8 | 3.8 | 0.01 | 0.01 | 0.01 | 0.01 | 0.15 | 0.13 | 0.15 | 0.12 | 500 |
| 7 | 5 | 100 | 250 | 0.5 | 0 | 2.5 | 0.5 | 1 | 5.8 | 5.8 | 6.0 | 5.6 | 0.00 | 0.00 | 0.00 | 0.00 | 0.16 | 0.13 | 0.16 | 0.13 | 500 |
| 7 | 5 | 100 | 250 | 0.5 | 0 | 2.5 | 0.5 | 2 | 5.6 | 5.6 | 6.0 | 5.4 | 0.00 | 0.00 | 0.00 | 0.00 | 0.16 | 0.13 | 0.16 | 0.13 | 500 |
| 7 | 5 | 100 | 250 | 0.5 | 0.2 | 1.5 | 2 | 1 | 25.6 | 25.4 | 25.8 | 25.0 | 0.00 | 0.03 | 0.00 | 0.03 | 0.16 | 0.13 | 0.16 | 0.13 | 500 |
| 7 | 5 | 100 | 250 | 0.5 | 0.2 | 1.5 | 2 | 2 | 24.0 | 23.4 | 24.4 | 23.4 | 0.01 | 0.02 | 0.01 | 0.02 | 0.16 | 0.13 | 0.16 | 0.13 | 500 |
| 7 | 5 | 100 | 250 | 0.5 | 0.2 | 2.5 | 0.5 | 1 | 25.0 | 24.2 | 25.2 | 24.0 | 0.01 | 0.04 | 0.01 | 0.04 | 0.16 | 0.13 | 0.16 | 0.13 | 500 |
| 7 | 5 | 100 | 250 | 0.5 | 0.2 | 2.5 | 0.5 | 2 | 27.4 | 26.8 | 27.4 | 26.6 | 0.01 | 0.03 | 0.00 | 0.03 | 0.16 | 0.13 | 0.16 | 0.13 | 500 |
| 7 | 5 | 100 | 250 | 0.5 | 0.5 | 1.5 | 2 | 1 | 88.6 | 88.6 | 89.2 | 88.8 | 0.00 | 0.07 | 0.00 | 0.08 | 0.16 | 0.14 | 0.16 | 0.14 | 500 |
| 7 | 5 | 100 | 250 | 0.5 | 0.5 | 1.5 | 2 | 2 | 88.0 | 87.8 | 88.0 | 87.8 | 0.00 | 0.08 | 0.00 | 0.07 | 0.16 | 0.14 | 0.16 | 0.14 | 500 |
| 7 | 5 | 100 | 250 | 0.5 | 0.5 | 2.5 | 0.5 | 1 | 89.6 | 89.0 | 89.6 | 89.0 | 0.00 | 0.09 | 0.00 | 0.09 | 0.15 | 0.13 | 0.15 | 0.13 | 500 |
| 7 | 5 | 100 | 250 | 0.5 | 0.5 | 2.5 | 0.5 | 2 | 89.6 | 89.0 | 89.6 | 89.2 | 0.01 | 0.10 | 0.01 | 0.10 | 0.16 | 0.13 | 0.15 | 0.13 | 500 |
| 7 | 5 | 100 | 250 | 2 | 0 | 1.5 | 2 | 1 | 4.6 | 4.6 | 4.8 | 4.8 | 0.00 | 0.00 | 0.00 | 0.00 | 0.16 | 0.12 | 0.16 | 0.12 | 500 |
| 7 | 5 | 100 | 250 | 2 | 0 | 1.5 | 2 | 2 | 4.6 | 4.4 | 5.0 | 4.4 | 0.01 | 0.00 | 0.01 | 0.00 | 0.16 | 0.12 | 0.17 | 0.12 | 500 |
| 7 | 5 | 100 | 250 | 2 | 0 | 2.5 | 0.5 | 1 | 7.2 | 7.2 | 7.4 | 7.2 | 0.00 | 0.00 | 0.00 | 0.00 | 0.17 | 0.13 | 0.18 | 0.14 | 500 |
| 7 | 5 | 100 | 250 | 2 | 0 | 2.5 | 0.5 | 2 | 5.2 | 5.2 | 5.2 | 4.8 | 0.01 | 0.01 | 0.01 | 0.01 | 0.16 | 0.12 | 0.17 | 0.13 | 500 |
| 7 | 5 | 100 | 250 | 2 | 0.2 | 1.5 | 2 | 1 | 23.6 | 23.2 | 23.6 | 23.2 | 0.01 | 0.05 | 0.01 | 0.05 | 0.16 | 0.12 | 0.16 | 0.12 | 500 |
| 7 | 5 | 100 | 250 | 2 | 0.2 | 1.5 | 2 | 2 | 24.2 | 23.4 | 24.0 | 23.0 | 0.01 | 0.04 | 0.01 | 0.04 | 0.17 | 0.12 | 0.17 | 0.12 | 500 |
| 7 | 5 | 100 | 250 | 2 | 0.2 | 2.5 | 0.5 | 1 | 26.2 | 25.6 | 26.4 | 25.8 | 0.00 | 0.05 | 0.01 | 0.04 | 0.17 | 0.13 | 0.17 | 0.13 | 500 |
| 7 | 5 | 100 | 250 | 2 | 0.2 | 2.5 | 0.5 | 2 | 22.8 | 22.0 | 22.6 | 22.2 | 0.00 | 0.05 | 0.00 | 0.05 | 0.16 | 0.12 | 0.17 | 0.13 | 500 |
| 7 | 5 | 100 | 250 | 2 | 0.5 | 1.5 | 2 | 1 | 85.2 | 85.0 | 85.4 | 85.0 | 0.01 | 0.12 | 0.02 | 0.12 | 0.17 | 0.13 | 0.17 | 0.13 | 500 |
| 7 | 5 | 100 | 250 | 2 | 0.5 | 1.5 | 2 | 2 | 85.8 | 85.6 | 86.4 | 85.8 | 0.00 | 0.13 | 0.01 | 0.12 | 0.16 | 0.13 | 0.17 | 0.13 | 500 |
| 7 | 5 | 100 | 250 | 2 | 0.5 | 2.5 | 0.5 | 1 | 86.8 | 86.2 | 86.6 | 86.4 | 0.01 | 0.11 | 0.02 | 0.10 | 0.16 | 0.13 | 0.16 | 0.13 | 500 |
| 7 | 5 | 100 | 250 | 2 | 0.5 | 2.5 | 0.5 | 2 | 88.0 | 88.0 | 88.0 | 88.0 | 0.00 | 0.12 | 0.00 | 0.11 | 0.16 | 0.13 | 0.16 | 0.13 | 500 |
| 7 | 5 | 100 | 500 | 0 | 0 | 1.5 | 2 | 1 | 6.4 | 5.8 | 6.0 | 5.8 | 0.02 | 0.01 | 0.02 | 0.01 | 0.16 | 0.14 | 0.16 | 0.14 | 500 |
| 7 | 5 | 100 | 500 | 0 | 0 | 1.5 | 2 | 2 | 3.4 | 3.2 | 3.4 | 3.0 | 0.01 | 0.01 | 0.01 | 0.01 | 0.15 | 0.13 | 0.15 | 0.13 | 500 |
| 7 | 5 | 100 | 500 | 0 | 0 | 2.5 | 0.5 | 1 | 4.0 | 3.8 | 4.2 | 3.6 | 0.01 | 0.01 | 0.01 | 0.01 | 0.15 | 0.12 | 0.15 | 0.12 | 500 |
| 7 | 5 | 100 | 500 | 0 | 0 | 2.5 | 0.5 | 2 | 5.2 | 4.4 | 5.2 | 4.6 | 0.00 | 0.00 | 0.00 | 0.00 | 0.16 | 0.13 | 0.16 | 0.13 | 500 |
| 7 | 5 | 100 | 500 | 0 | 0.2 | 1.5 | 2 | 1 | 26.2 | 25.0 | 26.4 | 24.8 | 0.00 | 0.03 | 0.00 | 0.03 | 0.16 | 0.13 | 0.16 | 0.13 | 500 |
| 7 | 5 | 100 | 500 | 0 | 0.2 | 1.5 | 2 | 2 | 26.6 | 26.6 | 26.8 | 26.6 | 0.00 | 0.03 | 0.00 | 0.03 | 0.16 | 0.14 | 0.16 | 0.14 | 500 |
| 7 | 5 | 100 | 500 | 0 | 0.2 | 2.5 | 0.5 | 1 | 26.2 | 25.0 | 26.4 | 25.6 | 0.01 | 0.03 | 0.01 | 0.03 | 0.16 | 0.13 | 0.16 | 0.13 | 500 |
| 7 | 5 | 100 | 500 | 0 | 0.2 | 2.5 | 0.5 | 2 | 26.6 | 26.0 | 26.8 | 25.8 | 0.00 | 0.03 | 0.00 | 0.04 | 0.17 | 0.14 | 0.17 | 0.14 | 500 |
| 7 | 5 | 100 | 500 | 0 | 0.5 | 1.5 | 2 | 1 | 91.0 | 90.6 | 91.0 | 90.6 | 0.02 | 0.05 | 0.02 | 0.06 | 0.16 | 0.14 | 0.16 | 0.14 | 500 |
| 7 | 5 | 100 | 500 | 0 | 0.5 | 1.5 | 2 | 2 | 85.4 | 85.2 | 85.8 | 85.0 | 0.01 | 0.08 | 0.01 | 0.09 | 0.16 | 0.14 | 0.16 | 0.14 | 500 |
| 7 | 5 | 100 | 500 | 0 | 0.5 | 2.5 | 0.5 | 1 | 88.2 | 88.0 | 88.4 | 88.2 | 0.00 | 0.08 | 0.00 | 0.08 | 0.16 | 0.14 | 0.16 | 0.14 | 500 |
| 7 | 5 | 100 | 500 | 0 | 0.5 | 2.5 | 0.5 | 2 | 89.0 | 89.0 | 89.0 | 89.0 | 0.00 | 0.09 | 0.00 | 0.09 | 0.16 | 0.13 | 0.16 | 0.13 | 500 |
| 7 | 5 | 100 | 500 | 0.5 | 0 | 1.5 | 2 | 1 | 4.8 | 4.8 | 5.0 | 4.4 | 0.00 | 0.00 | 0.00 | 0.00 | 0.16 | 0.13 | 0.16 | 0.13 | 500 |
| 7 | 5 | 100 | 500 | 0.5 | 0 | 1.5 | 2 | 2 | 3.8 | 3.8 | 3.8 | 3.6 | 0.00 | 0.00 | 0.00 | 0.00 | 0.15 | 0.13 | 0.15 | 0.13 | 500 |
| 7 | 5 | 100 | 500 | 0.5 | 0 | 2.5 | 0.5 | 1 | 5.0 | 4.8 | 5.4 | 4.6 | 0.00 | 0.00 | 0.00 | 0.00 | 0.16 | 0.13 | 0.16 | 0.13 | 500 |
| 7 | 5 | 100 | 500 | 0.5 | 0 | 2.5 | 0.5 | 2 | 4.8 | 4.6 | 5.0 | 4.6 | 0.01 | 0.01 | 0.01 | 0.01 | 0.16 | 0.13 | 0.15 | 0.13 | 500 |
| 7 | 5 | 100 | 500 | 0.5 | 0.2 | 1.5 | 2 | 1 | 27.8 | 27.2 | 28.2 | 27.4 | 0.01 | 0.02 | 0.01 | 0.02 | 0.16 | 0.13 | 0.15 | 0.13 | 500 |
| 7 | 5 | 100 | 500 | 0.5 | 0.2 | 1.5 | 2 | 2 | 25.6 | 24.8 | 25.8 | 25.0 | 0.00 | 0.03 | 0.00 | 0.03 | 0.15 | 0.13 | 0.15 | 0.13 | 500 |
| 7 | 5 | 100 | 500 | 0.5 | 0.2 | 2.5 | 0.5 | 1 | 21.4 | 20.6 | 21.8 | 20.8 | 0.01 | 0.04 | 0.01 | 0.05 | 0.15 | 0.13 | 0.15 | 0.13 | 500 |
| 7 | 5 | 100 | 500 | 0.5 | 0.2 | 2.5 | 0.5 | 2 | 25.2 | 24.8 | 25.8 | 24.8 | 0.01 | 0.03 | 0.01 | 0.03 | 0.15 | 0.12 | 0.15 | 0.12 | 500 |
| 7 | 5 | 100 | 500 | 0.5 | 0.5 | 1.5 | 2 | 1 | 89.0 | 88.8 | 89.6 | 89.2 | 0.00 | 0.07 | 0.00 | 0.07 | 0.16 | 0.14 | 0.15 | 0.13 | 500 |
| 7 | 5 | 100 | 500 | 0.5 | 0.5 | 1.5 | 2 | 2 | 88.0 | 87.6 | 88.2 | 87.4 | 0.00 | 0.08 | 0.01 | 0.08 | 0.16 | 0.14 | 0.16 | 0.14 | 500 |
| 7 | 5 | 100 | 500 | 0.5 | 0.5 | 2.5 | 0.5 | 1 | 88.6 | 88.4 | 88.8 | 88.2 | 0.00 | 0.09 | 0.00 | 0.09 | 0.16 | 0.14 | 0.16 | 0.14 | 500 |
| 7 | 5 | 100 | 500 | 0.5 | 0.5 | 2.5 | 0.5 | 2 | 90.6 | 90.2 | 90.6 | 90.4 | 0.01 | 0.08 | 0.01 | 0.08 | 0.16 | 0.13 | 0.16 | 0.13 | 500 |
| 7 | 5 | 100 | 500 | 2 | 0 | 1.5 | 2 | 1 | 5.8 | 5.8 | 6.0 | 5.4 | 0.00 | 0.00 | 0.00 | 0.00 | 0.17 | 0.12 | 0.17 | 0.12 | 500 |
| 7 | 5 | 100 | 500 | 2 | 0 | 1.5 | 2 | 2 | 5.8 | 5.8 | 6.0 | 5.8 | 0.00 | 0.00 | 0.00 | 0.00 | 0.17 | 0.12 | 0.17 | 0.12 | 500 |
| 7 | 5 | 100 | 500 | 2 | 0 | 2.5 | 0.5 | 1 | 6.2 | 6.2 | 6.4 | 6.2 | 0.01 | 0.01 | 0.01 | 0.01 | 0.17 | 0.13 | 0.17 | 0.13 | 500 |
| 7 | 5 | 100 | 500 | 2 | 0 | 2.5 | 0.5 | 2 | 4.8 | 4.6 | 4.8 | 4.6 | 0.00 | 0.00 | 0.00 | 0.00 | 0.16 | 0.12 | 0.16 | 0.12 | 500 |
| 7 | 5 | 100 | 500 | 2 | 0.2 | 1.5 | 2 | 1 | 24.0 | 23.4 | 24.6 | 23.8 | 0.00 | 0.05 | 0.00 | 0.05 | 0.16 | 0.12 | 0.16 | 0.12 | 500 |
| 7 | 5 | 100 | 500 | 2 | 0.2 | 1.5 | 2 | 2 | 21.8 | 21.4 | 22.0 | 21.4 | 0.00 | 0.05 | 0.00 | 0.05 | 0.17 | 0.13 | 0.17 | 0.13 | 500 |
| 7 | 5 | 100 | 500 | 2 | 0.2 | 2.5 | 0.5 | 1 | 25.0 | 24.6 | 25.0 | 24.4 | 0.00 | 0.05 | 0.00 | 0.05 | 0.17 | 0.13 | 0.17 | 0.13 | 500 |
| 7 | 5 | 100 | 500 | 2 | 0.2 | 2.5 | 0.5 | 2 | 22.8 | 22.4 | 22.8 | 22.4 | 0.01 | 0.05 | 0.01 | 0.04 | 0.16 | 0.12 | 0.16 | 0.12 | 500 |
| 7 | 5 | 100 | 500 | 2 | 0.5 | 1.5 | 2 | 1 | 85.0 | 84.8 | 85.2 | 84.8 | 0.01 | 0.12 | 0.01 | 0.12 | 0.17 | 0.13 | 0.18 | 0.13 | 500 |
| 7 | 5 | 100 | 500 | 2 | 0.5 | 1.5 | 2 | 2 | 84.2 | 84.2 | 84.4 | 84.2 | 0.00 | 0.13 | 0.00 | 0.13 | 0.18 | 0.14 | 0.18 | 0.14 | 500 |
| 7 | 5 | 100 | 500 | 2 | 0.5 | 2.5 | 0.5 | 1 | 88.4 | 88.2 | 88.4 | 88.2 | 0.02 | 0.10 | 0.03 | 0.10 | 0.17 | 0.14 | 0.17 | 0.14 | 500 |
| 7 | 5 | 100 | 500 | 2 | 0.5 | 2.5 | 0.5 | 2 | 87.0 | 86.4 | 87.2 | 86.4 | 0.00 | 0.12 | 0.01 | 0.11 | 0.16 | 0.13 | 0.16 | 0.13 | 500 |
| 7 | 5 | 200 | 100 | 0 | 0 | 1.5 | 2 | 1 | 4.6 | 4.4 | 4.6 | 4.4 | 0.00 | 0.00 | 0.00 | 0.00 | 0.11 | 0.09 | 0.11 | 0.09 | 482 |
| 7 | 5 | 200 | 100 | 0 | 0 | 1.5 | 2 | 2 | 4.8 | 4.8 | 4.8 | 4.8 | 0.00 | 0.00 | 0.00 | 0.00 | 0.11 | 0.09 | 0.11 | 0.10 | 500 |
| 7 | 5 | 200 | 100 | 0 | 0 | 2.5 | 0.5 | 1 | 4.1 | 4.0 | 4.2 | 3.8 | 0.00 | 0.00 | 0.00 | 0.00 | 0.11 | 0.09 | 0.11 | 0.09 | 489 |
| 7 | 5 | 200 | 100 | 0 | 0 | 2.5 | 0.5 | 2 | 3.6 | 3.6 | 3.6 | 3.6 | 0.00 | 0.00 | 0.00 | 0.00 | 0.11 | 0.09 | 0.11 | 0.09 | 500 |
| 7 | 5 | 200 | 100 | 0 | 0.2 | 1.5 | 2 | 1 | 47.1 | 47.1 | 47.1 | 47.1 | 0.00 | 0.03 | 0.01 | 0.02 | 0.11 | 0.10 | 0.12 | 0.10 | 486 |
| 7 | 5 | 200 | 100 | 0 | 0.2 | 1.5 | 2 | 2 | 47.0 | 47.0 | 46.8 | 46.4 | 0.01 | 0.03 | 0.01 | 0.02 | 0.11 | 0.09 | 0.11 | 0.09 | 500 |
| 7 | 5 | 200 | 100 | 0 | 0.2 | 2.5 | 0.5 | 1 | 44.0 | 43.8 | 43.8 | 43.8 | 0.01 | 0.03 | 0.01 | 0.03 | 0.11 | 0.09 | 0.11 | 0.09 | 498 |
| 7 | 5 | 200 | 100 | 0 | 0.2 | 2.5 | 0.5 | 2 | 44.4 | 43.8 | 45.0 | 44.8 | 0.00 | 0.03 | 0.01 | 0.03 | 0.11 | 0.09 | 0.11 | 0.09 | 500 |
| 7 | 5 | 200 | 100 | 0 | 0.5 | 1.5 | 2 | 1 | 99.8 | 99.8 | 99.8 | 99.8 | 0.01 | 0.07 | 0.02 | 0.06 | 0.11 | 0.09 | 0.11 | 0.10 | 479 |
| 7 | 5 | 200 | 100 | 0 | 0.5 | 1.5 | 2 | 2 | 99.2 | 99.2 | 99.2 | 99.2 | 0.00 | 0.07 | 0.01 | 0.06 | 0.10 | 0.09 | 0.11 | 0.10 | 500 |
| 7 | 5 | 200 | 100 | 0 | 0.5 | 2.5 | 0.5 | 1 | 99.0 | 98.2 | 98.2 | 98.2 | 0.00 | 0.08 | 0.01 | 0.08 | 0.12 | 0.10 | 0.12 | 0.11 | 496 |
| 7 | 5 | 200 | 100 | 0 | 0.5 | 2.5 | 0.5 | 2 | 99.6 | 99.6 | 99.6 | 99.6 | 0.01 | 0.08 | 0.02 | 0.07 | 0.12 | 0.10 | 0.12 | 0.11 | 500 |
| 7 | 5 | 200 | 100 | 0.5 | 0 | 1.5 | 2 | 1 | 5.9 | 5.6 | 5.6 | 5.6 | 0.00 | 0.00 | 0.00 | 0.00 | 0.11 | 0.09 | 0.11 | 0.09 | 477 |
| 7 | 5 | 200 | 100 | 0.5 | 0 | 1.5 | 2 | 2 | 5.4 | 5.4 | 5.6 | 5.6 | 0.00 | 0.00 | 0.00 | 0.00 | 0.11 | 0.09 | 0.11 | 0.09 | 500 |
| 7 | 5 | 200 | 100 | 0.5 | 0 | 2.5 | 0.5 | 1 | 4.7 | 4.6 | 4.6 | 4.6 | 0.00 | 0.00 | 0.00 | 0.00 | 0.11 | 0.09 | 0.11 | 0.09 | 489 |
| 7 | 5 | 200 | 100 | 0.5 | 0 | 2.5 | 0.5 | 2 | 5.4 | 5.4 | 5.4 | 5.4 | 0.01 | 0.01 | 0.01 | 0.00 | 0.11 | 0.09 | 0.11 | 0.09 | 500 |
| 7 | 5 | 200 | 100 | 0.5 | 0.2 | 1.5 | 2 | 1 | 47.0 | 47.0 | 47.0 | 47.0 | 0.00 | 0.03 | 0.01 | 0.03 | 0.12 | 0.10 | 0.12 | 0.10 | 477 |
| 7 | 5 | 200 | 100 | 0.5 | 0.2 | 1.5 | 2 | 2 | 40.4 | 40.4 | 41.2 | 40.6 | 0.01 | 0.04 | 0.01 | 0.04 | 0.11 | 0.09 | 0.11 | 0.09 | 500 |
| 7 | 5 | 200 | 100 | 0.5 | 0.2 | 2.5 | 0.5 | 1 | 48.9 | 48.2 | 48.8 | 48.6 | 0.01 | 0.03 | 0.01 | 0.02 | 0.12 | 0.10 | 0.12 | 0.10 | 497 |
| 7 | 5 | 200 | 100 | 0.5 | 0.2 | 2.5 | 0.5 | 2 | 41.6 | 41.4 | 41.4 | 41.2 | 0.01 | 0.04 | 0.00 | 0.04 | 0.12 | 0.10 | 0.12 | 0.10 | 500 |
| 7 | 5 | 200 | 100 | 0.5 | 0.5 | 1.5 | 2 | 1 | 100.0 | 95.2 | 95.2 | 95.2 | 0.01 | 0.07 | 0.01 | 0.07 | 0.11 | 0.09 | 0.11 | 0.10 | 476 |
| 7 | 5 | 200 | 100 | 0.5 | 0.5 | 1.5 | 2 | 2 | 99.6 | 99.4 | 99.4 | 99.4 | 0.01 | 0.07 | 0.02 | 0.06 | 0.11 | 0.10 | 0.12 | 0.11 | 499 |
| 7 | 5 | 200 | 100 | 0.5 | 0.5 | 2.5 | 0.5 | 1 | 99.6 | 99.6 | 99.6 | 99.6 | 0.01 | 0.08 | 0.02 | 0.07 | 0.11 | 0.09 | 0.11 | 0.10 | 492 |
| 7 | 5 | 200 | 100 | 0.5 | 0.5 | 2.5 | 0.5 | 2 | 99.4 | 99.4 | 99.4 | 99.4 | 0.00 | 0.09 | 0.01 | 0.08 | 0.10 | 0.09 | 0.11 | 0.10 | 500 |
| 7 | 5 | 200 | 100 | 2 | 0 | 1.5 | 2 | 1 | 5.8 | 5.4 | 5.6 | 5.6 | 0.01 | 0.01 | 0.01 | 0.01 | 0.12 | 0.09 | 0.12 | 0.09 | 482 |
| 7 | 5 | 200 | 100 | 2 | 0 | 1.5 | 2 | 2 | 3.4 | 3.4 | 3.0 | 3.0 | 0.00 | 0.00 | 0.00 | 0.00 | 0.11 | 0.08 | 0.11 | 0.08 | 500 |
| 7 | 5 | 200 | 100 | 2 | 0 | 2.5 | 0.5 | 1 | 4.9 | 4.6 | 4.6 | 4.6 | 0.00 | 0.00 | 0.00 | 0.00 | 0.11 | 0.09 | 0.12 | 0.09 | 494 |
| 7 | 5 | 200 | 100 | 2 | 0 | 2.5 | 0.5 | 2 | 5.6 | 5.6 | 5.4 | 5.4 | 0.00 | 0.00 | 0.00 | 0.00 | 0.12 | 0.09 | 0.12 | 0.09 | 500 |
| 7 | 5 | 200 | 100 | 2 | 0.2 | 1.5 | 2 | 1 | 39.1 | 39.1 | 39.1 | 39.1 | 0.00 | 0.05 | 0.01 | 0.05 | 0.12 | 0.08 | 0.12 | 0.09 | 481 |
| 7 | 5 | 200 | 100 | 2 | 0.2 | 1.5 | 2 | 2 | 41.0 | 40.8 | 41.2 | 40.0 | 0.00 | 0.06 | 0.00 | 0.05 | 0.12 | 0.08 | 0.12 | 0.09 | 500 |
| 7 | 5 | 200 | 100 | 2 | 0.2 | 2.5 | 0.5 | 1 | 39.1 | 38.2 | 38.2 | 38.0 | 0.00 | 0.05 | 0.01 | 0.04 | 0.12 | 0.09 | 0.12 | 0.09 | 494 |
| 7 | 5 | 200 | 100 | 2 | 0.2 | 2.5 | 0.5 | 2 | 41.2 | 41.2 | 41.4 | 41.2 | 0.01 | 0.05 | 0.00 | 0.05 | 0.11 | 0.09 | 0.12 | 0.09 | 500 |
| 7 | 5 | 200 | 100 | 2 | 0.5 | 1.5 | 2 | 1 | 99.4 | 99.4 | 99.4 | 99.4 | 0.01 | 0.12 | 0.03 | 0.11 | 0.11 | 0.09 | 0.13 | 0.10 | 476 |
| 7 | 5 | 200 | 100 | 2 | 0.5 | 1.5 | 2 | 2 | 99.4 | 99.4 | 99.4 | 99.4 | 0.01 | 0.13 | 0.02 | 0.11 | 0.12 | 0.10 | 0.13 | 0.10 | 500 |
| 7 | 5 | 200 | 100 | 2 | 0.5 | 2.5 | 0.5 | 1 | 98.8 | 98.8 | 98.8 | 98.8 | 0.00 | 0.12 | 0.02 | 0.10 | 0.11 | 0.09 | 0.12 | 0.10 | 492 |
| 7 | 5 | 200 | 100 | 2 | 0.5 | 2.5 | 0.5 | 2 | 99.4 | 99.4 | 99.4 | 99.4 | 0.00 | 0.12 | 0.02 | 0.10 | 0.11 | 0.09 | 0.12 | 0.09 | 500 |
| 7 | 5 | 200 | 250 | 0 | 0 | 1.5 | 2 | 1 | 5.2 | 5.2 | 5.2 | 5.2 | 0.01 | 0.00 | 0.01 | 0.00 | 0.11 | 0.09 | 0.11 | 0.09 | 500 |
| 7 | 5 | 200 | 250 | 0 | 0 | 1.5 | 2 | 2 | 6.4 | 6.4 | 6.4 | 6.4 | 0.01 | 0.01 | 0.01 | 0.01 | 0.11 | 0.10 | 0.11 | 0.10 | 500 |
| 7 | 5 | 200 | 250 | 0 | 0 | 2.5 | 0.5 | 1 | 6.8 | 6.8 | 6.8 | 6.8 | 0.00 | 0.00 | 0.00 | 0.00 | 0.12 | 0.10 | 0.12 | 0.10 | 500 |
| 7 | 5 | 200 | 250 | 0 | 0 | 2.5 | 0.5 | 2 | 6.2 | 6.2 | 6.2 | 6.2 | 0.00 | 0.00 | 0.00 | 0.00 | 0.11 | 0.09 | 0.11 | 0.09 | 500 |
| 7 | 5 | 200 | 250 | 0 | 0.2 | 1.5 | 2 | 1 | 42.6 | 42.6 | 42.8 | 42.6 | 0.00 | 0.03 | 0.00 | 0.03 | 0.11 | 0.09 | 0.11 | 0.09 | 500 |
| 7 | 5 | 200 | 250 | 0 | 0.2 | 1.5 | 2 | 2 | 43.6 | 43.6 | 44.0 | 43.4 | 0.00 | 0.03 | 0.00 | 0.03 | 0.11 | 0.10 | 0.11 | 0.09 | 500 |
| 7 | 5 | 200 | 250 | 0 | 0.2 | 2.5 | 0.5 | 1 | 43.0 | 42.4 | 43.2 | 42.0 | 0.00 | 0.03 | 0.00 | 0.03 | 0.11 | 0.09 | 0.11 | 0.09 | 500 |
| 7 | 5 | 200 | 250 | 0 | 0.2 | 2.5 | 0.5 | 2 | 44.4 | 43.8 | 44.6 | 44.2 | 0.00 | 0.03 | 0.00 | 0.03 | 0.11 | 0.09 | 0.11 | 0.09 | 500 |
| 7 | 5 | 200 | 250 | 0 | 0.5 | 1.5 | 2 | 1 | 98.8 | 98.8 | 98.8 | 98.8 | 0.01 | 0.07 | 0.01 | 0.07 | 0.11 | 0.10 | 0.11 | 0.10 | 500 |
| 7 | 5 | 200 | 250 | 0 | 0.5 | 1.5 | 2 | 2 | 100.0 | 100.0 | 100.0 | 100.0 | 0.00 | 0.07 | 0.00 | 0.07 | 0.11 | 0.09 | 0.11 | 0.09 | 500 |
| 7 | 5 | 200 | 250 | 0 | 0.5 | 2.5 | 0.5 | 1 | 100.0 | 99.8 | 100.0 | 99.8 | 0.00 | 0.09 | 0.00 | 0.08 | 0.11 | 0.10 | 0.11 | 0.10 | 500 |
| 7 | 5 | 200 | 250 | 0 | 0.5 | 2.5 | 0.5 | 2 | 99.4 | 99.4 | 99.4 | 99.4 | 0.00 | 0.09 | 0.00 | 0.09 | 0.11 | 0.10 | 0.11 | 0.10 | 500 |
| 7 | 5 | 200 | 250 | 0.5 | 0 | 1.5 | 2 | 1 | 5.2 | 5.0 | 5.2 | 5.0 | 0.01 | 0.01 | 0.01 | 0.01 | 0.11 | 0.09 | 0.11 | 0.09 | 500 |
| 7 | 5 | 200 | 250 | 0.5 | 0 | 1.5 | 2 | 2 | 4.8 | 4.6 | 5.0 | 4.6 | 0.00 | 0.00 | 0.00 | 0.00 | 0.11 | 0.09 | 0.11 | 0.09 | 500 |
| 7 | 5 | 200 | 250 | 0.5 | 0 | 2.5 | 0.5 | 1 | 6.2 | 6.0 | 6.0 | 6.0 | 0.01 | 0.01 | 0.01 | 0.01 | 0.11 | 0.09 | 0.11 | 0.09 | 500 |
| 7 | 5 | 200 | 250 | 0.5 | 0 | 2.5 | 0.5 | 2 | 4.4 | 4.2 | 4.4 | 4.4 | 0.01 | 0.01 | 0.01 | 0.01 | 0.11 | 0.09 | 0.11 | 0.09 | 500 |
| 7 | 5 | 200 | 250 | 0.5 | 0.2 | 1.5 | 2 | 1 | 46.0 | 45.6 | 46.0 | 46.0 | 0.00 | 0.03 | 0.00 | 0.03 | 0.11 | 0.10 | 0.11 | 0.10 | 500 |
| 7 | 5 | 200 | 250 | 0.5 | 0.2 | 1.5 | 2 | 2 | 40.0 | 39.6 | 39.6 | 39.0 | 0.01 | 0.04 | 0.01 | 0.04 | 0.11 | 0.10 | 0.11 | 0.10 | 500 |
| 7 | 5 | 200 | 250 | 0.5 | 0.2 | 2.5 | 0.5 | 1 | 42.2 | 42.2 | 42.8 | 42.4 | 0.00 | 0.04 | 0.00 | 0.03 | 0.11 | 0.09 | 0.11 | 0.09 | 500 |
| 7 | 5 | 200 | 250 | 0.5 | 0.2 | 2.5 | 0.5 | 2 | 41.0 | 40.8 | 40.8 | 40.2 | 0.00 | 0.04 | 0.00 | 0.04 | 0.11 | 0.09 | 0.11 | 0.09 | 500 |
| 7 | 5 | 200 | 250 | 0.5 | 0.5 | 1.5 | 2 | 1 | 99.6 | 99.6 | 99.6 | 99.6 | 0.01 | 0.07 | 0.01 | 0.07 | 0.11 | 0.09 | 0.11 | 0.09 | 500 |
| 7 | 5 | 200 | 250 | 0.5 | 0.5 | 1.5 | 2 | 2 | 99.4 | 99.4 | 99.6 | 99.6 | 0.00 | 0.08 | 0.00 | 0.07 | 0.11 | 0.10 | 0.11 | 0.10 | 500 |
| 7 | 5 | 200 | 250 | 0.5 | 0.5 | 2.5 | 0.5 | 1 | 99.4 | 99.4 | 99.4 | 99.4 | 0.01 | 0.09 | 0.00 | 0.09 | 0.12 | 0.10 | 0.12 | 0.10 | 500 |
| 7 | 5 | 200 | 250 | 0.5 | 0.5 | 2.5 | 0.5 | 2 | 99.8 | 99.8 | 99.8 | 99.8 | 0.00 | 0.08 | 0.00 | 0.08 | 0.12 | 0.10 | 0.12 | 0.10 | 500 |
| 7 | 5 | 200 | 250 | 2 | 0 | 1.5 | 2 | 1 | 6.4 | 6.4 | 6.4 | 6.4 | 0.01 | 0.01 | 0.01 | 0.01 | 0.12 | 0.09 | 0.13 | 0.09 | 500 |
| 7 | 5 | 200 | 250 | 2 | 0 | 1.5 | 2 | 2 | 4.2 | 4.2 | 4.2 | 4.2 | 0.00 | 0.00 | 0.00 | 0.00 | 0.11 | 0.08 | 0.11 | 0.08 | 500 |
| 7 | 5 | 200 | 250 | 2 | 0 | 2.5 | 0.5 | 1 | 3.2 | 3.0 | 3.2 | 3.2 | 0.01 | 0.01 | 0.01 | 0.01 | 0.11 | 0.08 | 0.11 | 0.08 | 500 |
| 7 | 5 | 200 | 250 | 2 | 0 | 2.5 | 0.5 | 2 | 4.8 | 4.8 | 5.0 | 5.0 | 0.01 | 0.00 | 0.01 | 0.00 | 0.12 | 0.09 | 0.12 | 0.09 | 500 |
| 7 | 5 | 200 | 250 | 2 | 0.2 | 1.5 | 2 | 1 | 41.2 | 40.6 | 41.2 | 40.6 | 0.00 | 0.05 | 0.01 | 0.05 | 0.12 | 0.09 | 0.12 | 0.09 | 500 |
| 7 | 5 | 200 | 250 | 2 | 0.2 | 1.5 | 2 | 2 | 40.0 | 39.6 | 39.4 | 39.2 | 0.00 | 0.06 | 0.00 | 0.05 | 0.12 | 0.09 | 0.12 | 0.09 | 500 |
| 7 | 5 | 200 | 250 | 2 | 0.2 | 2.5 | 0.5 | 1 | 44.6 | 44.0 | 44.4 | 43.6 | 0.01 | 0.04 | 0.01 | 0.04 | 0.12 | 0.09 | 0.12 | 0.09 | 500 |
| 7 | 5 | 200 | 250 | 2 | 0.2 | 2.5 | 0.5 | 2 | 44.4 | 44.0 | 44.6 | 44.2 | 0.01 | 0.04 | 0.01 | 0.04 | 0.11 | 0.09 | 0.12 | 0.09 | 500 |
| 7 | 5 | 200 | 250 | 2 | 0.5 | 1.5 | 2 | 1 | 99.0 | 99.0 | 99.0 | 99.0 | 0.00 | 0.13 | 0.01 | 0.12 | 0.12 | 0.09 | 0.12 | 0.09 | 500 |
| 7 | 5 | 200 | 250 | 2 | 0.5 | 1.5 | 2 | 2 | 98.6 | 98.6 | 98.6 | 98.6 | 0.00 | 0.13 | 0.01 | 0.13 | 0.12 | 0.09 | 0.12 | 0.09 | 500 |
| 7 | 5 | 200 | 250 | 2 | 0.5 | 2.5 | 0.5 | 1 | 99.2 | 99.2 | 99.2 | 99.2 | 0.00 | 0.12 | 0.01 | 0.11 | 0.11 | 0.09 | 0.11 | 0.09 | 500 |
| 7 | 5 | 200 | 250 | 2 | 0.5 | 2.5 | 0.5 | 2 | 98.6 | 98.6 | 98.8 | 98.6 | 0.00 | 0.12 | 0.01 | 0.11 | 0.12 | 0.10 | 0.12 | 0.10 | 500 |
| 7 | 5 | 200 | 500 | 0 | 0 | 1.5 | 2 | 1 | 4.2 | 4.0 | 4.2 | 4.0 | 0.00 | 0.00 | 0.00 | 0.00 | 0.11 | 0.09 | 0.11 | 0.09 | 500 |
| 7 | 5 | 200 | 500 | 0 | 0 | 1.5 | 2 | 2 | 5.4 | 5.4 | 5.6 | 5.6 | 0.00 | 0.00 | 0.00 | 0.00 | 0.11 | 0.09 | 0.11 | 0.09 | 500 |
| 7 | 5 | 200 | 500 | 0 | 0 | 2.5 | 0.5 | 1 | 4.8 | 4.8 | 4.8 | 4.8 | 0.00 | 0.00 | 0.00 | 0.00 | 0.11 | 0.09 | 0.11 | 0.09 | 500 |
| 7 | 5 | 200 | 500 | 0 | 0 | 2.5 | 0.5 | 2 | 5.6 | 5.6 | 5.6 | 5.4 | 0.00 | 0.00 | 0.00 | 0.00 | 0.12 | 0.09 | 0.11 | 0.09 | 500 |
| 7 | 5 | 200 | 500 | 0 | 0.2 | 1.5 | 2 | 1 | 43.8 | 43.6 | 44.2 | 44.2 | 0.00 | 0.03 | 0.00 | 0.03 | 0.11 | 0.10 | 0.11 | 0.10 | 500 |
| 7 | 5 | 200 | 500 | 0 | 0.2 | 1.5 | 2 | 2 | 45.0 | 44.6 | 44.8 | 44.6 | 0.00 | 0.03 | 0.00 | 0.03 | 0.11 | 0.09 | 0.11 | 0.09 | 500 |
| 7 | 5 | 200 | 500 | 0 | 0.2 | 2.5 | 0.5 | 1 | 41.2 | 40.2 | 41.0 | 40.6 | 0.01 | 0.04 | 0.01 | 0.04 | 0.11 | 0.09 | 0.11 | 0.09 | 500 |
| 7 | 5 | 200 | 500 | 0 | 0.2 | 2.5 | 0.5 | 2 | 43.6 | 43.2 | 43.6 | 43.6 | 0.00 | 0.04 | 0.00 | 0.04 | 0.11 | 0.09 | 0.11 | 0.09 | 500 |
| 7 | 5 | 200 | 500 | 0 | 0.5 | 1.5 | 2 | 1 | 99.8 | 99.8 | 99.8 | 99.8 | 0.00 | 0.07 | 0.00 | 0.07 | 0.11 | 0.09 | 0.11 | 0.09 | 500 |
| 7 | 5 | 200 | 500 | 0 | 0.5 | 1.5 | 2 | 2 | 99.4 | 99.4 | 99.4 | 99.4 | 0.00 | 0.08 | 0.00 | 0.07 | 0.11 | 0.10 | 0.11 | 0.10 | 500 |
| 7 | 5 | 200 | 500 | 0 | 0.5 | 2.5 | 0.5 | 1 | 99.6 | 99.6 | 99.6 | 99.6 | 0.00 | 0.09 | 0.01 | 0.09 | 0.12 | 0.10 | 0.12 | 0.10 | 500 |
| 7 | 5 | 200 | 500 | 0 | 0.5 | 2.5 | 0.5 | 2 | 99.2 | 99.2 | 99.2 | 99.2 | 0.01 | 0.08 | 0.01 | 0.08 | 0.11 | 0.10 | 0.11 | 0.10 | 500 |
| 7 | 5 | 200 | 500 | 0.5 | 0 | 1.5 | 2 | 1 | 4.8 | 4.6 | 4.8 | 4.6 | 0.01 | 0.01 | 0.01 | 0.01 | 0.11 | 0.09 | 0.11 | 0.09 | 500 |
| 7 | 5 | 200 | 500 | 0.5 | 0 | 1.5 | 2 | 2 | 7.2 | 7.2 | 7.2 | 7.2 | 0.01 | 0.01 | 0.01 | 0.01 | 0.11 | 0.10 | 0.11 | 0.10 | 500 |
| 7 | 5 | 200 | 500 | 0.5 | 0 | 2.5 | 0.5 | 1 | 4.2 | 4.2 | 4.2 | 4.2 | 0.00 | 0.00 | 0.00 | 0.00 | 0.11 | 0.09 | 0.11 | 0.09 | 500 |
| 7 | 5 | 200 | 500 | 0.5 | 0 | 2.5 | 0.5 | 2 | 5.0 | 5.0 | 5.4 | 5.2 | 0.01 | 0.01 | 0.01 | 0.01 | 0.11 | 0.09 | 0.11 | 0.09 | 500 |
| 7 | 5 | 200 | 500 | 0.5 | 0.2 | 1.5 | 2 | 1 | 44.0 | 44.0 | 44.2 | 44.0 | 0.00 | 0.03 | 0.00 | 0.03 | 0.11 | 0.09 | 0.11 | 0.09 | 500 |
| 7 | 5 | 200 | 500 | 0.5 | 0.2 | 1.5 | 2 | 2 | 45.2 | 45.0 | 45.0 | 44.6 | 0.00 | 0.03 | 0.00 | 0.03 | 0.11 | 0.09 | 0.11 | 0.09 | 500 |
| 7 | 5 | 200 | 500 | 0.5 | 0.2 | 2.5 | 0.5 | 1 | 45.4 | 45.2 | 45.4 | 45.2 | 0.01 | 0.03 | 0.01 | 0.03 | 0.11 | 0.09 | 0.11 | 0.09 | 500 |
| 7 | 5 | 200 | 500 | 0.5 | 0.2 | 2.5 | 0.5 | 2 | 48.2 | 47.6 | 48.2 | 47.4 | 0.00 | 0.03 | 0.00 | 0.04 | 0.11 | 0.09 | 0.11 | 0.09 | 500 |
| 7 | 5 | 200 | 500 | 0.5 | 0.5 | 1.5 | 2 | 1 | 99.6 | 99.6 | 99.6 | 99.6 | 0.00 | 0.08 | 0.00 | 0.08 | 0.11 | 0.10 | 0.11 | 0.10 | 500 |
| 7 | 5 | 200 | 500 | 0.5 | 0.5 | 1.5 | 2 | 2 | 99.0 | 99.0 | 99.0 | 99.0 | 0.00 | 0.08 | 0.01 | 0.08 | 0.11 | 0.10 | 0.11 | 0.10 | 500 |
| 7 | 5 | 200 | 500 | 0.5 | 0.5 | 2.5 | 0.5 | 1 | 99.2 | 99.2 | 99.4 | 99.2 | 0.01 | 0.08 | 0.00 | 0.08 | 0.11 | 0.09 | 0.11 | 0.10 | 500 |
| 7 | 5 | 200 | 500 | 0.5 | 0.5 | 2.5 | 0.5 | 2 | 99.2 | 99.2 | 99.2 | 99.2 | 0.00 | 0.09 | 0.00 | 0.09 | 0.11 | 0.09 | 0.11 | 0.09 | 500 |
| 7 | 5 | 200 | 500 | 2 | 0 | 1.5 | 2 | 1 | 4.2 | 4.2 | 4.2 | 4.2 | 0.00 | 0.00 | 0.00 | 0.00 | 0.12 | 0.09 | 0.12 | 0.09 | 500 |
| 7 | 5 | 200 | 500 | 2 | 0 | 1.5 | 2 | 2 | 4.4 | 4.4 | 4.4 | 4.4 | 0.00 | 0.00 | 0.00 | 0.00 | 0.12 | 0.09 | 0.12 | 0.09 | 500 |
| 7 | 5 | 200 | 500 | 2 | 0 | 2.5 | 0.5 | 1 | 4.8 | 4.8 | 4.8 | 4.8 | 0.00 | 0.00 | 0.00 | 0.00 | 0.11 | 0.09 | 0.11 | 0.09 | 500 |
| 7 | 5 | 200 | 500 | 2 | 0 | 2.5 | 0.5 | 2 | 7.2 | 7.0 | 7.2 | 7.0 | 0.00 | 0.00 | 0.00 | 0.00 | 0.12 | 0.09 | 0.12 | 0.09 | 500 |
| 7 | 5 | 200 | 500 | 2 | 0.2 | 1.5 | 2 | 1 | 39.6 | 39.4 | 40.2 | 39.6 | 0.00 | 0.05 | 0.00 | 0.05 | 0.12 | 0.09 | 0.12 | 0.09 | 500 |
| 7 | 5 | 200 | 500 | 2 | 0.2 | 1.5 | 2 | 2 | 39.0 | 38.8 | 39.0 | 39.0 | 0.00 | 0.05 | 0.00 | 0.05 | 0.11 | 0.08 | 0.12 | 0.08 | 500 |
| 7 | 5 | 200 | 500 | 2 | 0.2 | 2.5 | 0.5 | 1 | 40.8 | 40.4 | 40.6 | 40.6 | 0.00 | 0.05 | 0.00 | 0.05 | 0.12 | 0.09 | 0.12 | 0.09 | 500 |
| 7 | 5 | 200 | 500 | 2 | 0.2 | 2.5 | 0.5 | 2 | 43.4 | 43.2 | 43.4 | 43.0 | 0.00 | 0.05 | 0.00 | 0.05 | 0.12 | 0.09 | 0.12 | 0.09 | 500 |
| 7 | 5 | 200 | 500 | 2 | 0.5 | 1.5 | 2 | 1 | 98.2 | 98.2 | 98.2 | 98.2 | 0.00 | 0.13 | 0.00 | 0.13 | 0.13 | 0.10 | 0.13 | 0.10 | 500 |
| 7 | 5 | 200 | 500 | 2 | 0.5 | 1.5 | 2 | 2 | 99.4 | 99.4 | 99.4 | 99.4 | 0.01 | 0.13 | 0.01 | 0.13 | 0.12 | 0.09 | 0.12 | 0.09 | 500 |
| 7 | 5 | 200 | 500 | 2 | 0.5 | 2.5 | 0.5 | 1 | 99.4 | 99.4 | 99.4 | 99.4 | 0.00 | 0.12 | 0.01 | 0.11 | 0.11 | 0.09 | 0.11 | 0.09 | 500 |
| 7 | 5 | 200 | 500 | 2 | 0.5 | 2.5 | 0.5 | 2 | 98.8 | 98.8 | 98.8 | 98.8 | 0.00 | 0.12 | 0.01 | 0.11 | 0.12 | 0.10 | 0.12 | 0.10 | 500 |
| 7 | 5 | 500 | 100 | 0 | 0 | 1.5 | 2 | 1 | 5.0 | 4.8 | 4.8 | 4.8 | 0.00 | 0.00 | 0.00 | 0.00 | 0.07 | 0.06 | 0.07 | 0.06 | 481 |
| 7 | 5 | 500 | 100 | 0 | 0 | 1.5 | 2 | 2 | 4.2 | 4.2 | 4.2 | 4.2 | 0.00 | 0.00 | 0.00 | 0.00 | 0.07 | 0.06 | 0.07 | 0.06 | 500 |
| 7 | 5 | 500 | 100 | 0 | 0 | 2.5 | 0.5 | 1 | 4.9 | 4.8 | 5.0 | 5.0 | 0.00 | 0.00 | 0.00 | 0.00 | 0.07 | 0.06 | 0.07 | 0.06 | 492 |
| 7 | 5 | 500 | 100 | 0 | 0 | 2.5 | 0.5 | 2 | 6.6 | 6.6 | 6.6 | 6.4 | 0.00 | 0.00 | 0.00 | 0.00 | 0.07 | 0.06 | 0.07 | 0.06 | 500 |
| 7 | 5 | 500 | 100 | 0 | 0.2 | 1.5 | 2 | 1 | 83.8 | 83.8 | 83.8 | 83.8 | 0.00 | 0.03 | 0.00 | 0.03 | 0.07 | 0.06 | 0.07 | 0.06 | 476 |
| 7 | 5 | 500 | 100 | 0 | 0.2 | 1.5 | 2 | 2 | 82.2 | 82.2 | 82.2 | 82.2 | 0.00 | 0.03 | 0.01 | 0.03 | 0.07 | 0.06 | 0.07 | 0.06 | 500 |
| 7 | 5 | 500 | 100 | 0 | 0.2 | 2.5 | 0.5 | 1 | 79.2 | 79.2 | 79.2 | 79.2 | 0.00 | 0.04 | 0.00 | 0.03 | 0.07 | 0.06 | 0.07 | 0.06 | 495 |
| 7 | 5 | 500 | 100 | 0 | 0.2 | 2.5 | 0.5 | 2 | 83.6 | 83.6 | 84.0 | 83.8 | 0.00 | 0.04 | 0.00 | 0.03 | 0.06 | 0.05 | 0.07 | 0.06 | 500 |
| 7 | 5 | 500 | 100 | 0 | 0.5 | 1.5 | 2 | 1 | 100.0 | 100.0 | 100.0 | 100.0 | 0.00 | 0.07 | 0.01 | 0.06 | 0.07 | 0.06 | 0.08 | 0.07 | 477 |
| 7 | 5 | 500 | 100 | 0 | 0.5 | 1.5 | 2 | 2 | 100.0 | 100.0 | 100.0 | 100.0 | 0.00 | 0.07 | 0.01 | 0.06 | 0.07 | 0.06 | 0.08 | 0.07 | 500 |
| 7 | 5 | 500 | 100 | 0 | 0.5 | 2.5 | 0.5 | 1 | 100.0 | 100.0 | 100.0 | 100.0 | 0.01 | 0.08 | 0.01 | 0.07 | 0.07 | 0.06 | 0.08 | 0.07 | 490 |
| 7 | 5 | 500 | 100 | 0 | 0.5 | 2.5 | 0.5 | 2 | 100.0 | 100.0 | 100.0 | 100.0 | 0.00 | 0.08 | 0.01 | 0.08 | 0.07 | 0.06 | 0.08 | 0.07 | 500 |
| 7 | 5 | 500 | 100 | 0.5 | 0 | 1.5 | 2 | 1 | 4.6 | 4.4 | 4.4 | 4.0 | 0.00 | 0.00 | 0.00 | 0.00 | 0.07 | 0.06 | 0.07 | 0.06 | 475 |
| 7 | 5 | 500 | 100 | 0.5 | 0 | 1.5 | 2 | 2 | 5.2 | 5.2 | 5.2 | 5.2 | 0.00 | 0.00 | 0.00 | 0.00 | 0.07 | 0.06 | 0.07 | 0.06 | 499 |
| 7 | 5 | 500 | 100 | 0.5 | 0 | 2.5 | 0.5 | 1 | 6.5 | 6.4 | 7.2 | 6.4 | 0.00 | 0.00 | 0.00 | 0.00 | 0.07 | 0.06 | 0.07 | 0.06 | 494 |
| 7 | 5 | 500 | 100 | 0.5 | 0 | 2.5 | 0.5 | 2 | 4.4 | 4.2 | 4.4 | 4.2 | 0.01 | 0.00 | 0.01 | 0.00 | 0.07 | 0.05 | 0.07 | 0.06 | 500 |
| 7 | 5 | 500 | 100 | 0.5 | 0.2 | 1.5 | 2 | 1 | 81.6 | 81.1 | 81.6 | 81.1 | 0.00 | 0.03 | 0.00 | 0.03 | 0.07 | 0.06 | 0.08 | 0.06 | 478 |
| 7 | 5 | 500 | 100 | 0.5 | 0.2 | 1.5 | 2 | 2 | 83.0 | 82.8 | 83.0 | 83.0 | 0.00 | 0.03 | 0.00 | 0.03 | 0.07 | 0.06 | 0.07 | 0.06 | 500 |
| 7 | 5 | 500 | 100 | 0.5 | 0.2 | 2.5 | 0.5 | 1 | 81.9 | 80.4 | 80.8 | 80.6 | 0.00 | 0.03 | 0.01 | 0.03 | 0.07 | 0.06 | 0.07 | 0.06 | 491 |
| 7 | 5 | 500 | 100 | 0.5 | 0.2 | 2.5 | 0.5 | 2 | 77.8 | 77.6 | 77.8 | 77.8 | 0.00 | 0.04 | 0.00 | 0.03 | 0.07 | 0.06 | 0.07 | 0.06 | 500 |
| 7 | 5 | 500 | 100 | 0.5 | 0.5 | 1.5 | 2 | 1 | 100.0 | 100.0 | 100.0 | 100.0 | 0.00 | 0.07 | 0.01 | 0.06 | 0.07 | 0.06 | 0.07 | 0.07 | 476 |
| 7 | 5 | 500 | 100 | 0.5 | 0.5 | 1.5 | 2 | 2 | 100.0 | 100.0 | 100.0 | 100.0 | 0.00 | 0.08 | 0.01 | 0.07 | 0.07 | 0.06 | 0.08 | 0.07 | 499 |
| 7 | 5 | 500 | 100 | 0.5 | 0.5 | 2.5 | 0.5 | 1 | 100.0 | 100.0 | 100.0 | 100.0 | 0.00 | 0.09 | 0.01 | 0.08 | 0.07 | 0.06 | 0.08 | 0.07 | 491 |
| 7 | 5 | 500 | 100 | 0.5 | 0.5 | 2.5 | 0.5 | 2 | 100.0 | 100.0 | 100.0 | 100.0 | 0.00 | 0.08 | 0.01 | 0.08 | 0.07 | 0.06 | 0.08 | 0.07 | 500 |
| 7 | 5 | 500 | 100 | 2 | 0 | 1.5 | 2 | 1 | 4.4 | 4.0 | 4.2 | 4.0 | 0.00 | 0.00 | 0.00 | 0.00 | 0.08 | 0.05 | 0.08 | 0.06 | 483 |
| 7 | 5 | 500 | 100 | 2 | 0 | 1.5 | 2 | 2 | 5.4 | 5.4 | 5.4 | 5.4 | 0.00 | 0.00 | 0.00 | 0.00 | 0.08 | 0.05 | 0.08 | 0.06 | 500 |
| 7 | 5 | 500 | 100 | 2 | 0 | 2.5 | 0.5 | 1 | 4.5 | 4.4 | 4.4 | 4.2 | 0.00 | 0.00 | 0.00 | 0.00 | 0.07 | 0.05 | 0.07 | 0.06 | 493 |
| 7 | 5 | 500 | 100 | 2 | 0 | 2.5 | 0.5 | 2 | 4.6 | 4.6 | 4.6 | 4.6 | 0.00 | 0.00 | 0.00 | 0.00 | 0.07 | 0.05 | 0.07 | 0.06 | 500 |
| 7 | 5 | 500 | 100 | 2 | 0.2 | 1.5 | 2 | 1 | 78.2 | 76.0 | 76.4 | 76.4 | 0.00 | 0.05 | 0.01 | 0.05 | 0.07 | 0.05 | 0.08 | 0.06 | 486 |
| 7 | 5 | 500 | 100 | 2 | 0.2 | 1.5 | 2 | 2 | 74.8 | 74.8 | 74.8 | 74.8 | 0.00 | 0.05 | 0.00 | 0.05 | 0.08 | 0.06 | 0.08 | 0.06 | 500 |
| 7 | 5 | 500 | 100 | 2 | 0.2 | 2.5 | 0.5 | 1 | 76.8 | 75.8 | 76.2 | 75.8 | 0.01 | 0.05 | 0.00 | 0.05 | 0.07 | 0.06 | 0.08 | 0.06 | 496 |
| 7 | 5 | 500 | 100 | 2 | 0.2 | 2.5 | 0.5 | 2 | 75.6 | 75.6 | 75.6 | 75.6 | 0.00 | 0.05 | 0.00 | 0.05 | 0.08 | 0.06 | 0.08 | 0.06 | 500 |
| 7 | 5 | 500 | 100 | 2 | 0.5 | 1.5 | 2 | 1 | 100.0 | 100.0 | 100.0 | 100.0 | 0.00 | 0.13 | 0.02 | 0.11 | 0.08 | 0.06 | 0.09 | 0.07 | 481 |
| 7 | 5 | 500 | 100 | 2 | 0.5 | 1.5 | 2 | 2 | 100.0 | 100.0 | 100.0 | 100.0 | 0.00 | 0.13 | 0.02 | 0.12 | 0.08 | 0.06 | 0.08 | 0.07 | 500 |
| 7 | 5 | 500 | 100 | 2 | 0.5 | 2.5 | 0.5 | 1 | 100.0 | 100.0 | 100.0 | 100.0 | 0.00 | 0.12 | 0.01 | 0.10 | 0.08 | 0.06 | 0.08 | 0.07 | 493 |
| 7 | 5 | 500 | 100 | 2 | 0.5 | 2.5 | 0.5 | 2 | 100.0 | 100.0 | 100.0 | 100.0 | 0.01 | 0.11 | 0.02 | 0.10 | 0.08 | 0.06 | 0.08 | 0.07 | 499 |
| 7 | 5 | 500 | 250 | 0 | 0 | 1.5 | 2 | 1 | 6.8 | 6.8 | 6.8 | 6.8 | 0.00 | 0.00 | 0.00 | 0.00 | 0.07 | 0.06 | 0.07 | 0.06 | 500 |
| 7 | 5 | 500 | 250 | 0 | 0 | 1.5 | 2 | 2 | 6.2 | 6.0 | 6.0 | 6.0 | 0.00 | 0.00 | 0.00 | 0.00 | 0.07 | 0.06 | 0.07 | 0.06 | 500 |
| 7 | 5 | 500 | 250 | 0 | 0 | 2.5 | 0.5 | 1 | 5.2 | 5.2 | 5.2 | 5.2 | 0.00 | 0.00 | 0.00 | 0.00 | 0.07 | 0.06 | 0.07 | 0.06 | 500 |
| 7 | 5 | 500 | 250 | 0 | 0 | 2.5 | 0.5 | 2 | 3.8 | 3.8 | 3.8 | 3.8 | 0.00 | 0.00 | 0.00 | 0.00 | 0.07 | 0.06 | 0.07 | 0.06 | 500 |
| 7 | 5 | 500 | 250 | 0 | 0.2 | 1.5 | 2 | 1 | 80.8 | 80.8 | 80.8 | 80.6 | 0.00 | 0.03 | 0.00 | 0.03 | 0.07 | 0.06 | 0.07 | 0.06 | 500 |
| 7 | 5 | 500 | 250 | 0 | 0.2 | 1.5 | 2 | 2 | 84.8 | 84.8 | 84.4 | 84.2 | 0.00 | 0.03 | 0.01 | 0.03 | 0.07 | 0.06 | 0.07 | 0.06 | 500 |
| 7 | 5 | 500 | 250 | 0 | 0.2 | 2.5 | 0.5 | 1 | 81.6 | 81.4 | 81.4 | 81.4 | 0.00 | 0.03 | 0.00 | 0.03 | 0.07 | 0.06 | 0.07 | 0.06 | 500 |
| 7 | 5 | 500 | 250 | 0 | 0.2 | 2.5 | 0.5 | 2 | 80.8 | 80.8 | 80.8 | 80.6 | 0.00 | 0.04 | 0.00 | 0.03 | 0.07 | 0.06 | 0.07 | 0.06 | 500 |
| 7 | 5 | 500 | 250 | 0 | 0.5 | 1.5 | 2 | 1 | 100.0 | 100.0 | 100.0 | 100.0 | 0.00 | 0.07 | 0.01 | 0.07 | 0.07 | 0.06 | 0.07 | 0.06 | 499 |
| 7 | 5 | 500 | 250 | 0 | 0.5 | 1.5 | 2 | 2 | 100.0 | 100.0 | 100.0 | 100.0 | 0.00 | 0.07 | 0.00 | 0.07 | 0.07 | 0.06 | 0.07 | 0.06 | 500 |
| 7 | 5 | 500 | 250 | 0 | 0.5 | 2.5 | 0.5 | 1 | 100.0 | 100.0 | 100.0 | 100.0 | 0.01 | 0.08 | 0.01 | 0.08 | 0.07 | 0.06 | 0.07 | 0.06 | 500 |
| 7 | 5 | 500 | 250 | 0 | 0.5 | 2.5 | 0.5 | 2 | 100.0 | 100.0 | 100.0 | 100.0 | 0.00 | 0.09 | 0.00 | 0.08 | 0.07 | 0.06 | 0.07 | 0.06 | 500 |
| 7 | 5 | 500 | 250 | 0.5 | 0 | 1.5 | 2 | 1 | 3.2 | 3.0 | 3.2 | 3.2 | 0.00 | 0.00 | 0.00 | 0.00 | 0.06 | 0.05 | 0.07 | 0.05 | 500 |
| 7 | 5 | 500 | 250 | 0.5 | 0 | 1.5 | 2 | 2 | 4.0 | 3.8 | 4.2 | 4.0 | 0.00 | 0.00 | 0.00 | 0.00 | 0.07 | 0.06 | 0.07 | 0.06 | 500 |
| 7 | 5 | 500 | 250 | 0.5 | 0 | 2.5 | 0.5 | 1 | 5.0 | 5.0 | 5.0 | 5.0 | 0.01 | 0.01 | 0.01 | 0.01 | 0.07 | 0.06 | 0.07 | 0.06 | 500 |
| 7 | 5 | 500 | 250 | 0.5 | 0 | 2.5 | 0.5 | 2 | 5.0 | 5.0 | 4.8 | 4.8 | 0.00 | 0.00 | 0.00 | 0.00 | 0.07 | 0.06 | 0.07 | 0.06 | 500 |
| 7 | 5 | 500 | 250 | 0.5 | 0.2 | 1.5 | 2 | 1 | 80.8 | 80.8 | 80.6 | 80.6 | 0.00 | 0.03 | 0.00 | 0.03 | 0.07 | 0.06 | 0.07 | 0.06 | 500 |
| 7 | 5 | 500 | 250 | 0.5 | 0.2 | 1.5 | 2 | 2 | 82.4 | 82.2 | 82.6 | 82.2 | 0.00 | 0.03 | 0.00 | 0.03 | 0.07 | 0.06 | 0.07 | 0.06 | 500 |
| 7 | 5 | 500 | 250 | 0.5 | 0.2 | 2.5 | 0.5 | 1 | 82.6 | 82.4 | 82.8 | 82.8 | 0.00 | 0.03 | 0.00 | 0.03 | 0.07 | 0.06 | 0.07 | 0.06 | 500 |
| 7 | 5 | 500 | 250 | 0.5 | 0.2 | 2.5 | 0.5 | 2 | 83.2 | 83.2 | 83.4 | 83.2 | 0.00 | 0.04 | 0.00 | 0.04 | 0.07 | 0.06 | 0.07 | 0.06 | 500 |
| 7 | 5 | 500 | 250 | 0.5 | 0.5 | 1.5 | 2 | 1 | 100.0 | 100.0 | 100.0 | 100.0 | 0.00 | 0.08 | 0.01 | 0.07 | 0.07 | 0.06 | 0.07 | 0.06 | 500 |
| 7 | 5 | 500 | 250 | 0.5 | 0.5 | 1.5 | 2 | 2 | 100.0 | 100.0 | 100.0 | 100.0 | 0.00 | 0.07 | 0.01 | 0.07 | 0.07 | 0.06 | 0.07 | 0.06 | 500 |
| 7 | 5 | 500 | 250 | 0.5 | 0.5 | 2.5 | 0.5 | 1 | 100.0 | 100.0 | 100.0 | 100.0 | 0.00 | 0.09 | 0.00 | 0.09 | 0.07 | 0.06 | 0.07 | 0.06 | 500 |
| 7 | 5 | 500 | 250 | 0.5 | 0.5 | 2.5 | 0.5 | 2 | 100.0 | 100.0 | 100.0 | 100.0 | 0.00 | 0.09 | 0.00 | 0.09 | 0.07 | 0.06 | 0.07 | 0.06 | 500 |
| 7 | 5 | 500 | 250 | 2 | 0 | 1.5 | 2 | 1 | 5.2 | 5.2 | 5.4 | 5.2 | 0.00 | 0.00 | 0.00 | 0.00 | 0.08 | 0.06 | 0.08 | 0.06 | 500 |
| 7 | 5 | 500 | 250 | 2 | 0 | 1.5 | 2 | 2 | 4.0 | 4.0 | 4.0 | 4.0 | 0.00 | 0.00 | 0.00 | 0.00 | 0.07 | 0.05 | 0.07 | 0.05 | 500 |
| 7 | 5 | 500 | 250 | 2 | 0 | 2.5 | 0.5 | 1 | 5.0 | 4.8 | 5.2 | 5.0 | 0.01 | 0.00 | 0.01 | 0.00 | 0.08 | 0.06 | 0.08 | 0.06 | 500 |
| 7 | 5 | 500 | 250 | 2 | 0 | 2.5 | 0.5 | 2 | 7.0 | 6.8 | 7.0 | 6.8 | 0.00 | 0.00 | 0.00 | 0.00 | 0.08 | 0.06 | 0.08 | 0.06 | 500 |
| 7 | 5 | 500 | 250 | 2 | 0.2 | 1.5 | 2 | 1 | 73.6 | 73.6 | 73.4 | 73.2 | 0.01 | 0.06 | 0.01 | 0.06 | 0.07 | 0.05 | 0.08 | 0.06 | 500 |
| 7 | 5 | 500 | 250 | 2 | 0.2 | 1.5 | 2 | 2 | 77.0 | 77.0 | 77.0 | 77.0 | 0.00 | 0.06 | 0.00 | 0.05 | 0.08 | 0.06 | 0.08 | 0.06 | 500 |
| 7 | 5 | 500 | 250 | 2 | 0.2 | 2.5 | 0.5 | 1 | 77.6 | 77.4 | 77.6 | 77.6 | 0.00 | 0.05 | 0.01 | 0.04 | 0.08 | 0.06 | 0.08 | 0.06 | 500 |
| 7 | 5 | 500 | 250 | 2 | 0.2 | 2.5 | 0.5 | 2 | 79.6 | 79.0 | 79.4 | 79.2 | 0.00 | 0.05 | 0.01 | 0.04 | 0.08 | 0.06 | 0.08 | 0.06 | 500 |
| 7 | 5 | 500 | 250 | 2 | 0.5 | 1.5 | 2 | 1 | 100.0 | 100.0 | 100.0 | 100.0 | 0.00 | 0.13 | 0.01 | 0.12 | 0.08 | 0.06 | 0.08 | 0.06 | 500 |
| 7 | 5 | 500 | 250 | 2 | 0.5 | 1.5 | 2 | 2 | 100.0 | 100.0 | 100.0 | 100.0 | 0.00 | 0.13 | 0.00 | 0.13 | 0.08 | 0.06 | 0.08 | 0.06 | 500 |
| 7 | 5 | 500 | 250 | 2 | 0.5 | 2.5 | 0.5 | 1 | 100.0 | 100.0 | 100.0 | 100.0 | 0.00 | 0.12 | 0.01 | 0.11 | 0.08 | 0.06 | 0.08 | 0.07 | 500 |
| 7 | 5 | 500 | 250 | 2 | 0.5 | 2.5 | 0.5 | 2 | 100.0 | 100.0 | 100.0 | 100.0 | 0.00 | 0.12 | 0.00 | 0.12 | 0.07 | 0.06 | 0.08 | 0.06 | 500 |
| 7 | 5 | 500 | 500 | 0 | 0 | 1.5 | 2 | 1 | 6.0 | 5.8 | 6.0 | 6.0 | 0.00 | 0.00 | 0.00 | 0.00 | 0.07 | 0.06 | 0.07 | 0.06 | 500 |
| 7 | 5 | 500 | 500 | 0 | 0 | 1.5 | 2 | 2 | 5.8 | 5.8 | 5.8 | 5.8 | 0.00 | 0.00 | 0.00 | 0.00 | 0.07 | 0.06 | 0.07 | 0.06 | 500 |
| 7 | 5 | 500 | 500 | 0 | 0 | 2.5 | 0.5 | 1 | 6.6 | 6.6 | 6.8 | 6.6 | 0.00 | 0.00 | 0.00 | 0.00 | 0.07 | 0.06 | 0.07 | 0.06 | 500 |
| 7 | 5 | 500 | 500 | 0 | 0 | 2.5 | 0.5 | 2 | 6.6 | 6.6 | 6.8 | 6.6 | 0.00 | 0.00 | 0.00 | 0.00 | 0.07 | 0.06 | 0.07 | 0.06 | 500 |
| 7 | 5 | 500 | 500 | 0 | 0.2 | 1.5 | 2 | 1 | 82.4 | 82.4 | 82.4 | 82.2 | 0.00 | 0.03 | 0.00 | 0.03 | 0.07 | 0.06 | 0.07 | 0.06 | 500 |
| 7 | 5 | 500 | 500 | 0 | 0.2 | 1.5 | 2 | 2 | 82.6 | 82.6 | 82.8 | 82.8 | 0.00 | 0.03 | 0.00 | 0.03 | 0.07 | 0.06 | 0.07 | 0.06 | 500 |
| 7 | 5 | 500 | 500 | 0 | 0.2 | 2.5 | 0.5 | 1 | 82.0 | 82.0 | 82.0 | 82.0 | 0.01 | 0.03 | 0.01 | 0.03 | 0.07 | 0.06 | 0.07 | 0.06 | 500 |
| 7 | 5 | 500 | 500 | 0 | 0.2 | 2.5 | 0.5 | 2 | 81.0 | 80.8 | 81.4 | 81.2 | 0.00 | 0.03 | 0.00 | 0.03 | 0.08 | 0.06 | 0.08 | 0.06 | 500 |
| 7 | 5 | 500 | 500 | 0 | 0.5 | 1.5 | 2 | 1 | 100.0 | 100.0 | 100.0 | 100.0 | 0.00 | 0.07 | 0.00 | 0.07 | 0.07 | 0.06 | 0.07 | 0.06 | 500 |
| 7 | 5 | 500 | 500 | 0 | 0.5 | 1.5 | 2 | 2 | 100.0 | 100.0 | 100.0 | 100.0 | 0.01 | 0.08 | 0.00 | 0.08 | 0.07 | 0.06 | 0.07 | 0.06 | 500 |
| 7 | 5 | 500 | 500 | 0 | 0.5 | 2.5 | 0.5 | 1 | 100.0 | 100.0 | 100.0 | 100.0 | 0.01 | 0.08 | 0.01 | 0.08 | 0.07 | 0.06 | 0.07 | 0.06 | 500 |
| 7 | 5 | 500 | 500 | 0 | 0.5 | 2.5 | 0.5 | 2 | 100.0 | 100.0 | 100.0 | 100.0 | 0.00 | 0.09 | 0.00 | 0.09 | 0.08 | 0.06 | 0.08 | 0.07 | 500 |
| 7 | 5 | 500 | 500 | 0.5 | 0 | 1.5 | 2 | 1 | 4.6 | 4.4 | 4.6 | 4.4 | 0.00 | 0.00 | 0.00 | 0.00 | 0.07 | 0.05 | 0.07 | 0.05 | 500 |
| 7 | 5 | 500 | 500 | 0.5 | 0 | 1.5 | 2 | 2 | 5.4 | 5.4 | 5.4 | 5.4 | 0.00 | 0.00 | 0.00 | 0.00 | 0.07 | 0.06 | 0.07 | 0.06 | 500 |
| 7 | 5 | 500 | 500 | 0.5 | 0 | 2.5 | 0.5 | 1 | 5.4 | 5.2 | 5.4 | 5.2 | 0.00 | 0.00 | 0.00 | 0.00 | 0.07 | 0.06 | 0.07 | 0.06 | 500 |
| 7 | 5 | 500 | 500 | 0.5 | 0 | 2.5 | 0.5 | 2 | 5.4 | 5.2 | 5.4 | 5.2 | 0.00 | 0.00 | 0.00 | 0.00 | 0.07 | 0.06 | 0.07 | 0.06 | 500 |
| 7 | 5 | 500 | 500 | 0.5 | 0.2 | 1.5 | 2 | 1 | 83.6 | 83.4 | 83.6 | 83.4 | 0.00 | 0.03 | 0.00 | 0.03 | 0.07 | 0.06 | 0.07 | 0.06 | 500 |
| 7 | 5 | 500 | 500 | 0.5 | 0.2 | 1.5 | 2 | 2 | 83.6 | 83.4 | 83.8 | 83.8 | 0.00 | 0.03 | 0.00 | 0.03 | 0.07 | 0.06 | 0.07 | 0.06 | 500 |
| 7 | 5 | 500 | 500 | 0.5 | 0.2 | 2.5 | 0.5 | 1 | 78.8 | 78.4 | 78.6 | 78.0 | 0.00 | 0.04 | 0.00 | 0.04 | 0.07 | 0.06 | 0.07 | 0.06 | 500 |
| 7 | 5 | 500 | 500 | 0.5 | 0.2 | 2.5 | 0.5 | 2 | 80.6 | 80.6 | 80.6 | 80.6 | 0.00 | 0.04 | 0.00 | 0.04 | 0.07 | 0.06 | 0.07 | 0.06 | 500 |
| 7 | 5 | 500 | 500 | 0.5 | 0.5 | 1.5 | 2 | 1 | 100.0 | 100.0 | 100.0 | 100.0 | 0.00 | 0.08 | 0.00 | 0.08 | 0.07 | 0.06 | 0.07 | 0.06 | 500 |
| 7 | 5 | 500 | 500 | 0.5 | 0.5 | 1.5 | 2 | 2 | 100.0 | 100.0 | 100.0 | 100.0 | 0.00 | 0.08 | 0.00 | 0.08 | 0.07 | 0.06 | 0.07 | 0.06 | 500 |
| 7 | 5 | 500 | 500 | 0.5 | 0.5 | 2.5 | 0.5 | 1 | 100.0 | 100.0 | 100.0 | 100.0 | 0.00 | 0.09 | 0.00 | 0.09 | 0.07 | 0.06 | 0.07 | 0.06 | 500 |
| 7 | 5 | 500 | 500 | 0.5 | 0.5 | 2.5 | 0.5 | 2 | 100.0 | 100.0 | 100.0 | 100.0 | 0.00 | 0.09 | 0.00 | 0.09 | 0.07 | 0.06 | 0.07 | 0.06 | 500 |
| 7 | 5 | 500 | 500 | 2 | 0 | 1.5 | 2 | 1 | 3.6 | 3.6 | 3.6 | 3.6 | 0.00 | 0.00 | 0.00 | 0.00 | 0.07 | 0.05 | 0.07 | 0.05 | 500 |
| 7 | 5 | 500 | 500 | 2 | 0 | 1.5 | 2 | 2 | 4.2 | 4.2 | 4.4 | 4.2 | 0.00 | 0.00 | 0.00 | 0.00 | 0.07 | 0.05 | 0.07 | 0.05 | 500 |
| 7 | 5 | 500 | 500 | 2 | 0 | 2.5 | 0.5 | 1 | 5.8 | 5.8 | 5.8 | 5.8 | 0.00 | 0.00 | 0.00 | 0.00 | 0.07 | 0.06 | 0.07 | 0.06 | 500 |
| 7 | 5 | 500 | 500 | 2 | 0 | 2.5 | 0.5 | 2 | 5.8 | 5.8 | 5.8 | 5.8 | 0.00 | 0.00 | 0.00 | 0.00 | 0.07 | 0.06 | 0.07 | 0.06 | 500 |
| 7 | 5 | 500 | 500 | 2 | 0.2 | 1.5 | 2 | 1 | 76.8 | 76.6 | 77.2 | 76.8 | 0.00 | 0.05 | 0.00 | 0.05 | 0.08 | 0.06 | 0.08 | 0.06 | 500 |
| 7 | 5 | 500 | 500 | 2 | 0.2 | 1.5 | 2 | 2 | 75.4 | 75.4 | 75.4 | 75.4 | 0.00 | 0.06 | 0.00 | 0.05 | 0.08 | 0.06 | 0.08 | 0.06 | 500 |
| 7 | 5 | 500 | 500 | 2 | 0.2 | 2.5 | 0.5 | 1 | 77.0 | 76.8 | 77.0 | 76.6 | 0.00 | 0.05 | 0.00 | 0.05 | 0.07 | 0.06 | 0.07 | 0.06 | 500 |
| 7 | 5 | 500 | 500 | 2 | 0.2 | 2.5 | 0.5 | 2 | 79.2 | 79.2 | 79.2 | 79.2 | 0.00 | 0.05 | 0.00 | 0.05 | 0.07 | 0.05 | 0.07 | 0.06 | 500 |
| 7 | 5 | 500 | 500 | 2 | 0.5 | 1.5 | 2 | 1 | 100.0 | 100.0 | 100.0 | 100.0 | 0.00 | 0.13 | 0.00 | 0.13 | 0.07 | 0.06 | 0.08 | 0.06 | 500 |
| 7 | 5 | 500 | 500 | 2 | 0.5 | 1.5 | 2 | 2 | 100.0 | 100.0 | 100.0 | 100.0 | 0.00 | 0.13 | 0.00 | 0.13 | 0.08 | 0.06 | 0.08 | 0.06 | 500 |
| 7 | 5 | 500 | 500 | 2 | 0.5 | 2.5 | 0.5 | 1 | 100.0 | 100.0 | 100.0 | 100.0 | 0.01 | 0.12 | 0.01 | 0.11 | 0.07 | 0.05 | 0.07 | 0.06 | 500 |
| 7 | 5 | 500 | 500 | 2 | 0.5 | 2.5 | 0.5 | 2 | 100.0 | 100.0 | 100.0 | 100.0 | 0.00 | 0.12 | 0.00 | 0.12 | 0.08 | 0.06 | 0.08 | 0.06 | 500 |
| 10 | 3 | 50 | 100 | 0 | 0 | 1.5 | 2 | 1 | 5.4 | 5.2 | 5.4 | 5.4 | 0.00 | 0.00 | 0.00 | 0.00 | 0.23 | 0.18 | 0.23 | 0.18 | 500 |
| 10 | 3 | 50 | 100 | 0 | 0 | 1.5 | 2 | 2 | 6.2 | 6.2 | 6.4 | 6.2 | 0.02 | 0.01 | 0.02 | 0.01 | 0.23 | 0.18 | 0.23 | 0.18 | 500 |
| 10 | 3 | 50 | 100 | 0 | 0 | 2.5 | 0.5 | 1 | 5.2 | 4.4 | 5.2 | 4.4 | 0.01 | 0.01 | 0.01 | 0.01 | 0.23 | 0.18 | 0.23 | 0.18 | 500 |
| 10 | 3 | 50 | 100 | 0 | 0 | 2.5 | 0.5 | 2 | 4.0 | 3.8 | 4.0 | 3.8 | 0.01 | 0.01 | 0.01 | 0.01 | 0.23 | 0.18 | 0.23 | 0.18 | 500 |
| 10 | 3 | 50 | 100 | 0 | 0.2 | 1.5 | 2 | 1 | 18.2 | 17.2 | 18.0 | 17.2 | 0.03 | 0.02 | 0.02 | 0.02 | 0.23 | 0.19 | 0.23 | 0.18 | 500 |
| 10 | 3 | 50 | 100 | 0 | 0.2 | 1.5 | 2 | 2 | 15.0 | 14.0 | 15.2 | 14.0 | 0.01 | 0.04 | 0.01 | 0.04 | 0.23 | 0.19 | 0.23 | 0.19 | 500 |
| 10 | 3 | 50 | 100 | 0 | 0.2 | 2.5 | 0.5 | 1 | 13.0 | 11.2 | 13.4 | 11.2 | 0.00 | 0.04 | 0.00 | 0.04 | 0.21 | 0.17 | 0.21 | 0.17 | 500 |
| 10 | 3 | 50 | 100 | 0 | 0.2 | 2.5 | 0.5 | 2 | 15.2 | 14.8 | 15.2 | 14.8 | 0.00 | 0.04 | 0.00 | 0.04 | 0.23 | 0.18 | 0.23 | 0.18 | 500 |
| 10 | 3 | 50 | 100 | 0 | 0.5 | 1.5 | 2 | 1 | 65.0 | 64.0 | 65.0 | 64.0 | 0.03 | 0.08 | 0.03 | 0.07 | 0.23 | 0.20 | 0.23 | 0.20 | 500 |
| 10 | 3 | 50 | 100 | 0 | 0.5 | 1.5 | 2 | 2 | 61.4 | 59.8 | 62.0 | 59.6 | 0.01 | 0.09 | 0.01 | 0.09 | 0.24 | 0.20 | 0.24 | 0.20 | 500 |
| 10 | 3 | 50 | 100 | 0 | 0.5 | 2.5 | 0.5 | 1 | 60.2 | 59.0 | 60.2 | 58.8 | 0.00 | 0.10 | 0.00 | 0.10 | 0.23 | 0.19 | 0.23 | 0.19 | 500 |
| 10 | 3 | 50 | 100 | 0 | 0.5 | 2.5 | 0.5 | 2 | 61.0 | 59.4 | 61.0 | 59.4 | 0.02 | 0.09 | 0.02 | 0.09 | 0.24 | 0.19 | 0.23 | 0.19 | 500 |
| 10 | 3 | 50 | 100 | 0.5 | 0 | 1.5 | 2 | 1 | 5.8 | 5.6 | 5.8 | 5.8 | 0.01 | 0.01 | 0.01 | 0.00 | 0.24 | 0.19 | 0.24 | 0.19 | 500 |
| 10 | 3 | 50 | 100 | 0.5 | 0 | 1.5 | 2 | 2 | 4.6 | 4.2 | 5.0 | 4.2 | 0.00 | 0.00 | 0.00 | 0.00 | 0.22 | 0.18 | 0.22 | 0.18 | 500 |
| 10 | 3 | 50 | 100 | 0.5 | 0 | 2.5 | 0.5 | 1 | 7.0 | 6.4 | 7.0 | 6.4 | 0.00 | 0.00 | 0.00 | 0.00 | 0.24 | 0.19 | 0.24 | 0.19 | 500 |
| 10 | 3 | 50 | 100 | 0.5 | 0 | 2.5 | 0.5 | 2 | 4.4 | 4.2 | 4.4 | 4.2 | 0.01 | 0.00 | 0.00 | 0.00 | 0.22 | 0.17 | 0.22 | 0.17 | 500 |
| 10 | 3 | 50 | 100 | 0.5 | 0.2 | 1.5 | 2 | 1 | 13.2 | 12.8 | 13.2 | 12.8 | 0.01 | 0.05 | 0.01 | 0.05 | 0.23 | 0.18 | 0.23 | 0.18 | 500 |
| 10 | 3 | 50 | 100 | 0.5 | 0.2 | 1.5 | 2 | 2 | 12.8 | 12.0 | 12.8 | 12.0 | 0.00 | 0.04 | 0.00 | 0.04 | 0.21 | 0.17 | 0.22 | 0.17 | 500 |
| 10 | 3 | 50 | 100 | 0.5 | 0.2 | 2.5 | 0.5 | 1 | 14.2 | 13.4 | 14.2 | 13.4 | 0.00 | 0.04 | 0.00 | 0.04 | 0.23 | 0.18 | 0.23 | 0.18 | 500 |
| 10 | 3 | 50 | 100 | 0.5 | 0.2 | 2.5 | 0.5 | 2 | 14.2 | 13.0 | 14.6 | 13.0 | 0.01 | 0.05 | 0.01 | 0.05 | 0.23 | 0.18 | 0.23 | 0.18 | 500 |
| 10 | 3 | 50 | 100 | 0.5 | 0.5 | 1.5 | 2 | 1 | 62.8 | 61.2 | 63.0 | 61.4 | 0.01 | 0.09 | 0.01 | 0.09 | 0.24 | 0.20 | 0.24 | 0.20 | 500 |
| 10 | 3 | 50 | 100 | 0.5 | 0.5 | 1.5 | 2 | 2 | 66.6 | 65.8 | 66.8 | 66.0 | 0.03 | 0.08 | 0.03 | 0.07 | 0.22 | 0.19 | 0.22 | 0.18 | 500 |
| 10 | 3 | 50 | 100 | 0.5 | 0.5 | 2.5 | 0.5 | 1 | 60.2 | 58.6 | 60.8 | 58.8 | 0.01 | 0.10 | 0.01 | 0.10 | 0.23 | 0.19 | 0.23 | 0.19 | 500 |
| 10 | 3 | 50 | 100 | 0.5 | 0.5 | 2.5 | 0.5 | 2 | 56.6 | 55.2 | 57.0 | 55.2 | 0.00 | 0.11 | 0.00 | 0.10 | 0.23 | 0.19 | 0.24 | 0.19 | 500 |
| 10 | 3 | 50 | 100 | 2 | 0 | 1.5 | 2 | 1 | 5.2 | 5.0 | 5.4 | 5.2 | 0.01 | 0.01 | 0.01 | 0.01 | 0.24 | 0.16 | 0.24 | 0.16 | 500 |
| 10 | 3 | 50 | 100 | 2 | 0 | 1.5 | 2 | 2 | 4.2 | 4.0 | 4.2 | 4.0 | 0.01 | 0.01 | 0.01 | 0.01 | 0.25 | 0.17 | 0.25 | 0.17 | 500 |
| 10 | 3 | 50 | 100 | 2 | 0 | 2.5 | 0.5 | 1 | 8.0 | 7.6 | 8.0 | 7.6 | 0.02 | 0.02 | 0.02 | 0.02 | 0.26 | 0.18 | 0.26 | 0.18 | 500 |
| 10 | 3 | 50 | 100 | 2 | 0 | 2.5 | 0.5 | 2 | 4.8 | 4.4 | 5.0 | 4.4 | 0.00 | 0.00 | 0.00 | 0.00 | 0.24 | 0.16 | 0.24 | 0.16 | 500 |
| 10 | 3 | 50 | 100 | 2 | 0.2 | 1.5 | 2 | 1 | 11.4 | 11.2 | 11.4 | 11.2 | 0.00 | 0.07 | 0.00 | 0.06 | 0.25 | 0.17 | 0.25 | 0.17 | 500 |
| 10 | 3 | 50 | 100 | 2 | 0.2 | 1.5 | 2 | 2 | 12.0 | 11.0 | 12.2 | 11.0 | 0.03 | 0.09 | 0.03 | 0.08 | 0.25 | 0.17 | 0.25 | 0.17 | 500 |
| 10 | 3 | 50 | 100 | 2 | 0.2 | 2.5 | 0.5 | 1 | 12.4 | 12.0 | 12.4 | 12.0 | 0.01 | 0.07 | 0.01 | 0.07 | 0.24 | 0.17 | 0.25 | 0.17 | 500 |
| 10 | 3 | 50 | 100 | 2 | 0.2 | 2.5 | 0.5 | 2 | 15.2 | 13.8 | 15.2 | 13.8 | 0.02 | 0.05 | 0.02 | 0.05 | 0.25 | 0.17 | 0.25 | 0.17 | 500 |
| 10 | 3 | 50 | 100 | 2 | 0.5 | 1.5 | 2 | 1 | 51.8 | 49.8 | 51.8 | 50.0 | 0.00 | 0.16 | 0.01 | 0.15 | 0.26 | 0.19 | 0.26 | 0.19 | 500 |
| 10 | 3 | 50 | 100 | 2 | 0.5 | 1.5 | 2 | 2 | 52.4 | 51.8 | 53.4 | 51.6 | 0.01 | 0.15 | 0.02 | 0.15 | 0.26 | 0.18 | 0.26 | 0.18 | 500 |
| 10 | 3 | 50 | 100 | 2 | 0.5 | 2.5 | 0.5 | 1 | 58.8 | 58.0 | 59.4 | 58.0 | 0.02 | 0.14 | 0.03 | 0.14 | 0.24 | 0.17 | 0.24 | 0.17 | 500 |
| 10 | 3 | 50 | 100 | 2 | 0.5 | 2.5 | 0.5 | 2 | 58.2 | 57.0 | 58.2 | 57.0 | 0.03 | 0.13 | 0.03 | 0.13 | 0.26 | 0.19 | 0.26 | 0.19 | 500 |
| 10 | 3 | 50 | 250 | 0 | 0 | 1.5 | 2 | 1 | 4.6 | 4.6 | 4.6 | 4.6 | 0.01 | 0.01 | 0.01 | 0.01 | 0.23 | 0.18 | 0.22 | 0.18 | 500 |
| 10 | 3 | 50 | 250 | 0 | 0 | 1.5 | 2 | 2 | 4.8 | 4.4 | 4.8 | 4.6 | 0.01 | 0.00 | 0.01 | 0.01 | 0.22 | 0.18 | 0.22 | 0.18 | 500 |
| 10 | 3 | 50 | 250 | 0 | 0 | 2.5 | 0.5 | 1 | 6.2 | 5.8 | 6.2 | 5.8 | 0.00 | 0.00 | 0.00 | 0.00 | 0.23 | 0.18 | 0.23 | 0.18 | 500 |
| 10 | 3 | 50 | 250 | 0 | 0 | 2.5 | 0.5 | 2 | 4.4 | 3.8 | 4.4 | 3.8 | 0.02 | 0.01 | 0.02 | 0.01 | 0.22 | 0.17 | 0.22 | 0.17 | 500 |
| 10 | 3 | 50 | 250 | 0 | 0.2 | 1.5 | 2 | 1 | 16.2 | 15.6 | 16.0 | 15.8 | 0.01 | 0.03 | 0.01 | 0.04 | 0.23 | 0.19 | 0.23 | 0.18 | 500 |
| 10 | 3 | 50 | 250 | 0 | 0.2 | 1.5 | 2 | 2 | 15.2 | 14.4 | 15.2 | 14.8 | 0.01 | 0.03 | 0.01 | 0.03 | 0.22 | 0.17 | 0.21 | 0.17 | 500 |
| 10 | 3 | 50 | 250 | 0 | 0.2 | 2.5 | 0.5 | 1 | 13.6 | 12.8 | 13.6 | 13.0 | 0.01 | 0.05 | 0.01 | 0.05 | 0.23 | 0.18 | 0.23 | 0.18 | 500 |
| 10 | 3 | 50 | 250 | 0 | 0.2 | 2.5 | 0.5 | 2 | 13.8 | 13.2 | 14.2 | 13.0 | 0.00 | 0.04 | 0.00 | 0.04 | 0.22 | 0.18 | 0.22 | 0.17 | 500 |
| 10 | 3 | 50 | 250 | 0 | 0.5 | 1.5 | 2 | 1 | 56.8 | 54.6 | 56.8 | 55.0 | 0.01 | 0.10 | 0.01 | 0.11 | 0.22 | 0.18 | 0.21 | 0.18 | 500 |
| 10 | 3 | 50 | 250 | 0 | 0.5 | 1.5 | 2 | 2 | 59.8 | 59.4 | 60.0 | 59.6 | 0.00 | 0.09 | 0.00 | 0.10 | 0.22 | 0.18 | 0.22 | 0.18 | 500 |
| 10 | 3 | 50 | 250 | 0 | 0.5 | 2.5 | 0.5 | 1 | 59.6 | 57.8 | 60.0 | 57.8 | 0.01 | 0.11 | 0.02 | 0.12 | 0.25 | 0.20 | 0.24 | 0.20 | 500 |
| 10 | 3 | 50 | 250 | 0 | 0.5 | 2.5 | 0.5 | 2 | 60.6 | 59.2 | 61.0 | 59.2 | 0.01 | 0.10 | 0.00 | 0.10 | 0.24 | 0.20 | 0.24 | 0.19 | 500 |
| 10 | 3 | 50 | 250 | 0.5 | 0 | 1.5 | 2 | 1 | 5.4 | 5.0 | 5.2 | 5.0 | 0.01 | 0.01 | 0.01 | 0.01 | 0.23 | 0.18 | 0.23 | 0.18 | 500 |
| 10 | 3 | 50 | 250 | 0.5 | 0 | 1.5 | 2 | 2 | 5.4 | 5.2 | 5.4 | 5.2 | 0.01 | 0.01 | 0.01 | 0.01 | 0.24 | 0.19 | 0.24 | 0.19 | 500 |
| 10 | 3 | 50 | 250 | 0.5 | 0 | 2.5 | 0.5 | 1 | 5.2 | 5.0 | 5.2 | 5.0 | 0.01 | 0.01 | 0.01 | 0.01 | 0.24 | 0.18 | 0.23 | 0.18 | 500 |
| 10 | 3 | 50 | 250 | 0.5 | 0 | 2.5 | 0.5 | 2 | 4.4 | 4.0 | 4.4 | 4.0 | 0.01 | 0.01 | 0.01 | 0.00 | 0.23 | 0.18 | 0.22 | 0.17 | 500 |
| 10 | 3 | 50 | 250 | 0.5 | 0.2 | 1.5 | 2 | 1 | 12.6 | 11.8 | 12.6 | 11.8 | 0.01 | 0.05 | 0.01 | 0.05 | 0.22 | 0.18 | 0.22 | 0.18 | 500 |
| 10 | 3 | 50 | 250 | 0.5 | 0.2 | 1.5 | 2 | 2 | 16.0 | 15.4 | 16.4 | 15.2 | 0.00 | 0.04 | 0.01 | 0.05 | 0.24 | 0.19 | 0.24 | 0.19 | 500 |
| 10 | 3 | 50 | 250 | 0.5 | 0.2 | 2.5 | 0.5 | 1 | 16.6 | 16.0 | 16.8 | 16.0 | 0.02 | 0.03 | 0.02 | 0.03 | 0.22 | 0.18 | 0.22 | 0.17 | 500 |
| 10 | 3 | 50 | 250 | 0.5 | 0.2 | 2.5 | 0.5 | 2 | 13.0 | 12.2 | 13.2 | 12.4 | 0.01 | 0.04 | 0.00 | 0.04 | 0.21 | 0.17 | 0.21 | 0.16 | 500 |
| 10 | 3 | 50 | 250 | 0.5 | 0.5 | 1.5 | 2 | 1 | 61.2 | 59.6 | 61.2 | 59.4 | 0.01 | 0.10 | 0.00 | 0.10 | 0.22 | 0.18 | 0.22 | 0.18 | 500 |
| 10 | 3 | 50 | 250 | 0.5 | 0.5 | 1.5 | 2 | 2 | 62.2 | 60.8 | 62.2 | 60.6 | 0.02 | 0.09 | 0.01 | 0.09 | 0.23 | 0.19 | 0.23 | 0.19 | 500 |
| 10 | 3 | 50 | 250 | 0.5 | 0.5 | 2.5 | 0.5 | 1 | 63.2 | 61.4 | 63.4 | 61.4 | 0.02 | 0.09 | 0.01 | 0.10 | 0.24 | 0.19 | 0.24 | 0.19 | 500 |
| 10 | 3 | 50 | 250 | 0.5 | 0.5 | 2.5 | 0.5 | 2 | 60.6 | 59.4 | 60.6 | 59.6 | 0.00 | 0.10 | 0.00 | 0.11 | 0.23 | 0.19 | 0.23 | 0.18 | 500 |
| 10 | 3 | 50 | 250 | 2 | 0 | 1.5 | 2 | 1 | 4.2 | 4.0 | 4.4 | 4.0 | 0.01 | 0.01 | 0.01 | 0.01 | 0.25 | 0.17 | 0.25 | 0.16 | 500 |
| 10 | 3 | 50 | 250 | 2 | 0 | 1.5 | 2 | 2 | 6.8 | 6.2 | 6.8 | 6.4 | 0.01 | 0.00 | 0.01 | 0.00 | 0.26 | 0.18 | 0.26 | 0.18 | 500 |
| 10 | 3 | 50 | 250 | 2 | 0 | 2.5 | 0.5 | 1 | 7.0 | 6.8 | 7.0 | 6.8 | 0.00 | 0.00 | 0.01 | 0.00 | 0.26 | 0.18 | 0.26 | 0.18 | 500 |
| 10 | 3 | 50 | 250 | 2 | 0 | 2.5 | 0.5 | 2 | 5.8 | 5.0 | 5.8 | 4.8 | 0.01 | 0.00 | 0.01 | 0.00 | 0.25 | 0.17 | 0.25 | 0.17 | 500 |
| 10 | 3 | 50 | 250 | 2 | 0.2 | 1.5 | 2 | 1 | 14.6 | 14.0 | 14.6 | 14.0 | 0.01 | 0.07 | 0.01 | 0.07 | 0.26 | 0.17 | 0.26 | 0.17 | 500 |
| 10 | 3 | 50 | 250 | 2 | 0.2 | 1.5 | 2 | 2 | 14.8 | 13.6 | 14.8 | 13.6 | 0.00 | 0.06 | 0.00 | 0.06 | 0.25 | 0.17 | 0.25 | 0.17 | 500 |
| 10 | 3 | 50 | 250 | 2 | 0.2 | 2.5 | 0.5 | 1 | 14.4 | 13.8 | 14.6 | 13.8 | 0.02 | 0.05 | 0.02 | 0.05 | 0.25 | 0.17 | 0.25 | 0.17 | 500 |
| 10 | 3 | 50 | 250 | 2 | 0.2 | 2.5 | 0.5 | 2 | 12.2 | 11.0 | 12.4 | 11.0 | 0.00 | 0.07 | 0.00 | 0.07 | 0.24 | 0.16 | 0.24 | 0.16 | 500 |
| 10 | 3 | 50 | 250 | 2 | 0.5 | 1.5 | 2 | 1 | 55.4 | 53.8 | 55.4 | 54.0 | 0.02 | 0.14 | 0.02 | 0.15 | 0.24 | 0.17 | 0.24 | 0.17 | 500 |
| 10 | 3 | 50 | 250 | 2 | 0.5 | 1.5 | 2 | 2 | 54.0 | 53.2 | 54.2 | 53.4 | 0.03 | 0.14 | 0.02 | 0.14 | 0.27 | 0.19 | 0.26 | 0.19 | 500 |
| 10 | 3 | 50 | 250 | 2 | 0.5 | 2.5 | 0.5 | 1 | 57.6 | 56.0 | 58.2 | 56.0 | 0.01 | 0.15 | 0.00 | 0.15 | 0.25 | 0.18 | 0.25 | 0.18 | 500 |
| 10 | 3 | 50 | 250 | 2 | 0.5 | 2.5 | 0.5 | 2 | 52.2 | 51.4 | 52.4 | 51.4 | 0.00 | 0.15 | 0.00 | 0.15 | 0.25 | 0.18 | 0.25 | 0.18 | 500 |
| 10 | 3 | 50 | 500 | 0 | 0 | 1.5 | 2 | 1 | 7.2 | 6.8 | 7.2 | 6.8 | 0.02 | 0.01 | 0.02 | 0.01 | 0.24 | 0.19 | 0.24 | 0.19 | 500 |
| 10 | 3 | 50 | 500 | 0 | 0 | 1.5 | 2 | 2 | 5.0 | 4.4 | 4.8 | 4.4 | 0.01 | 0.01 | 0.01 | 0.01 | 0.23 | 0.18 | 0.23 | 0.18 | 500 |
| 10 | 3 | 50 | 500 | 0 | 0 | 2.5 | 0.5 | 1 | 6.2 | 5.8 | 6.2 | 5.8 | 0.00 | 0.00 | 0.00 | 0.00 | 0.24 | 0.18 | 0.23 | 0.18 | 500 |
| 10 | 3 | 50 | 500 | 0 | 0 | 2.5 | 0.5 | 2 | 6.4 | 6.0 | 6.6 | 6.2 | 0.03 | 0.02 | 0.03 | 0.02 | 0.23 | 0.18 | 0.23 | 0.18 | 500 |
| 10 | 3 | 50 | 500 | 0 | 0.2 | 1.5 | 2 | 1 | 14.8 | 14.4 | 14.8 | 14.4 | 0.01 | 0.05 | 0.01 | 0.05 | 0.23 | 0.18 | 0.22 | 0.18 | 500 |
| 10 | 3 | 50 | 500 | 0 | 0.2 | 1.5 | 2 | 2 | 14.6 | 14.2 | 14.6 | 14.2 | 0.01 | 0.04 | 0.00 | 0.04 | 0.22 | 0.18 | 0.22 | 0.17 | 500 |
| 10 | 3 | 50 | 500 | 0 | 0.2 | 2.5 | 0.5 | 1 | 15.8 | 15.0 | 16.0 | 15.2 | 0.01 | 0.04 | 0.01 | 0.04 | 0.23 | 0.19 | 0.23 | 0.18 | 500 |
| 10 | 3 | 50 | 500 | 0 | 0.2 | 2.5 | 0.5 | 2 | 15.8 | 15.2 | 16.0 | 15.2 | 0.00 | 0.04 | 0.00 | 0.04 | 0.24 | 0.19 | 0.23 | 0.18 | 500 |
| 10 | 3 | 50 | 500 | 0 | 0.5 | 1.5 | 2 | 1 | 60.8 | 60.4 | 61.0 | 60.4 | 0.02 | 0.08 | 0.01 | 0.09 | 0.24 | 0.20 | 0.24 | 0.20 | 500 |
| 10 | 3 | 50 | 500 | 0 | 0.5 | 1.5 | 2 | 2 | 61.2 | 60.6 | 62.0 | 60.6 | 0.02 | 0.08 | 0.01 | 0.09 | 0.24 | 0.20 | 0.23 | 0.19 | 500 |
| 10 | 3 | 50 | 500 | 0 | 0.5 | 2.5 | 0.5 | 1 | 61.8 | 59.8 | 62.4 | 59.8 | 0.02 | 0.09 | 0.01 | 0.09 | 0.23 | 0.19 | 0.23 | 0.19 | 500 |
| 10 | 3 | 50 | 500 | 0 | 0.5 | 2.5 | 0.5 | 2 | 58.2 | 56.8 | 58.8 | 56.8 | 0.00 | 0.11 | 0.01 | 0.11 | 0.22 | 0.18 | 0.22 | 0.18 | 500 |
| 10 | 3 | 50 | 500 | 0.5 | 0 | 1.5 | 2 | 1 | 6.2 | 6.0 | 6.2 | 6.0 | 0.00 | 0.00 | 0.00 | 0.00 | 0.23 | 0.18 | 0.23 | 0.18 | 500 |
| 10 | 3 | 50 | 500 | 0.5 | 0 | 1.5 | 2 | 2 | 4.8 | 4.4 | 4.8 | 4.4 | 0.00 | 0.00 | 0.00 | 0.00 | 0.22 | 0.17 | 0.22 | 0.17 | 500 |
| 10 | 3 | 50 | 500 | 0.5 | 0 | 2.5 | 0.5 | 1 | 4.8 | 4.8 | 5.0 | 4.8 | 0.02 | 0.01 | 0.02 | 0.01 | 0.23 | 0.18 | 0.22 | 0.17 | 500 |
| 10 | 3 | 50 | 500 | 0.5 | 0 | 2.5 | 0.5 | 2 | 5.2 | 4.6 | 5.6 | 4.6 | 0.00 | 0.00 | 0.00 | 0.00 | 0.23 | 0.18 | 0.22 | 0.17 | 500 |
| 10 | 3 | 50 | 500 | 0.5 | 0.2 | 1.5 | 2 | 1 | 16.0 | 14.8 | 16.2 | 14.8 | 0.00 | 0.04 | 0.00 | 0.05 | 0.23 | 0.19 | 0.23 | 0.18 | 500 |
| 10 | 3 | 50 | 500 | 0.5 | 0.2 | 1.5 | 2 | 2 | 14.6 | 13.8 | 14.8 | 13.6 | 0.01 | 0.04 | 0.01 | 0.04 | 0.22 | 0.18 | 0.22 | 0.17 | 500 |
| 10 | 3 | 50 | 500 | 0.5 | 0.2 | 2.5 | 0.5 | 1 | 14.0 | 13.8 | 14.0 | 13.8 | 0.01 | 0.04 | 0.01 | 0.04 | 0.22 | 0.17 | 0.21 | 0.17 | 500 |
| 10 | 3 | 50 | 500 | 0.5 | 0.2 | 2.5 | 0.5 | 2 | 16.6 | 15.0 | 16.8 | 15.0 | 0.01 | 0.04 | 0.01 | 0.04 | 0.24 | 0.19 | 0.24 | 0.19 | 500 |
| 10 | 3 | 50 | 500 | 0.5 | 0.5 | 1.5 | 2 | 1 | 60.6 | 58.8 | 60.6 | 58.8 | 0.02 | 0.09 | 0.01 | 0.09 | 0.23 | 0.19 | 0.23 | 0.19 | 500 |
| 10 | 3 | 50 | 500 | 0.5 | 0.5 | 1.5 | 2 | 2 | 57.8 | 56.0 | 58.0 | 56.0 | 0.00 | 0.10 | 0.01 | 0.11 | 0.23 | 0.19 | 0.23 | 0.19 | 500 |
| 10 | 3 | 50 | 500 | 0.5 | 0.5 | 2.5 | 0.5 | 1 | 62.4 | 61.6 | 62.4 | 61.6 | 0.01 | 0.10 | 0.00 | 0.10 | 0.23 | 0.19 | 0.22 | 0.18 | 500 |
| 10 | 3 | 50 | 500 | 0.5 | 0.5 | 2.5 | 0.5 | 2 | 58.4 | 57.6 | 58.6 | 57.8 | 0.01 | 0.10 | 0.00 | 0.11 | 0.23 | 0.19 | 0.23 | 0.18 | 500 |
| 10 | 3 | 50 | 500 | 2 | 0 | 1.5 | 2 | 1 | 6.0 | 5.6 | 6.0 | 5.6 | 0.02 | 0.01 | 0.02 | 0.01 | 0.25 | 0.17 | 0.25 | 0.17 | 500 |
| 10 | 3 | 50 | 500 | 2 | 0 | 1.5 | 2 | 2 | 5.2 | 4.8 | 5.2 | 4.8 | 0.01 | 0.00 | 0.01 | 0.00 | 0.25 | 0.16 | 0.24 | 0.16 | 500 |
| 10 | 3 | 50 | 500 | 2 | 0 | 2.5 | 0.5 | 1 | 4.6 | 4.6 | 4.8 | 4.6 | 0.00 | 0.00 | 0.00 | 0.00 | 0.26 | 0.17 | 0.25 | 0.17 | 500 |
| 10 | 3 | 50 | 500 | 2 | 0 | 2.5 | 0.5 | 2 | 5.2 | 4.2 | 5.6 | 4.2 | 0.02 | 0.02 | 0.02 | 0.02 | 0.24 | 0.16 | 0.24 | 0.16 | 500 |
| 10 | 3 | 50 | 500 | 2 | 0.2 | 1.5 | 2 | 1 | 12.4 | 11.6 | 12.4 | 11.6 | 0.02 | 0.08 | 0.02 | 0.08 | 0.25 | 0.17 | 0.25 | 0.17 | 500 |
| 10 | 3 | 50 | 500 | 2 | 0.2 | 1.5 | 2 | 2 | 14.0 | 13.4 | 14.2 | 13.2 | 0.00 | 0.07 | 0.00 | 0.07 | 0.25 | 0.17 | 0.25 | 0.16 | 500 |
| 10 | 3 | 50 | 500 | 2 | 0.2 | 2.5 | 0.5 | 1 | 14.8 | 14.4 | 15.0 | 14.2 | 0.01 | 0.05 | 0.01 | 0.06 | 0.23 | 0.16 | 0.23 | 0.16 | 500 |
| 10 | 3 | 50 | 500 | 2 | 0.2 | 2.5 | 0.5 | 2 | 12.0 | 11.0 | 12.2 | 11.2 | 0.02 | 0.07 | 0.02 | 0.08 | 0.25 | 0.18 | 0.25 | 0.17 | 500 |
| 10 | 3 | 50 | 500 | 2 | 0.5 | 1.5 | 2 | 1 | 54.0 | 53.0 | 54.2 | 53.0 | 0.01 | 0.16 | 0.01 | 0.16 | 0.25 | 0.18 | 0.25 | 0.18 | 500 |
| 10 | 3 | 50 | 500 | 2 | 0.5 | 1.5 | 2 | 2 | 56.2 | 55.4 | 57.0 | 55.4 | 0.02 | 0.14 | 0.02 | 0.15 | 0.26 | 0.19 | 0.26 | 0.18 | 500 |
| 10 | 3 | 50 | 500 | 2 | 0.5 | 2.5 | 0.5 | 1 | 55.6 | 54.2 | 55.6 | 54.2 | 0.01 | 0.15 | 0.00 | 0.15 | 0.25 | 0.18 | 0.25 | 0.18 | 500 |
| 10 | 3 | 50 | 500 | 2 | 0.5 | 2.5 | 0.5 | 2 | 55.0 | 53.8 | 55.2 | 53.6 | 0.02 | 0.14 | 0.01 | 0.15 | 0.26 | 0.19 | 0.26 | 0.19 | 500 |
| 10 | 3 | 100 | 100 | 0 | 0 | 1.5 | 2 | 1 | 4.2 | 4.2 | 4.2 | 4.2 | 0.00 | 0.00 | 0.00 | 0.00 | 0.16 | 0.12 | 0.16 | 0.13 | 500 |
| 10 | 3 | 100 | 100 | 0 | 0 | 1.5 | 2 | 2 | 5.2 | 5.0 | 5.0 | 5.0 | 0.01 | 0.00 | 0.01 | 0.01 | 0.16 | 0.13 | 0.16 | 0.13 | 500 |
| 10 | 3 | 100 | 100 | 0 | 0 | 2.5 | 0.5 | 1 | 4.4 | 4.2 | 4.4 | 4.4 | 0.01 | 0.01 | 0.01 | 0.01 | 0.16 | 0.12 | 0.16 | 0.12 | 500 |
| 10 | 3 | 100 | 100 | 0 | 0 | 2.5 | 0.5 | 2 | 6.0 | 5.8 | 6.0 | 5.8 | 0.00 | 0.00 | 0.00 | 0.00 | 0.16 | 0.12 | 0.16 | 0.13 | 500 |
| 10 | 3 | 100 | 100 | 0 | 0.2 | 1.5 | 2 | 1 | 25.2 | 24.2 | 25.0 | 24.2 | 0.01 | 0.05 | 0.01 | 0.05 | 0.16 | 0.13 | 0.16 | 0.13 | 500 |
| 10 | 3 | 100 | 100 | 0 | 0.2 | 1.5 | 2 | 2 | 26.0 | 25.6 | 26.0 | 25.6 | 0.01 | 0.03 | 0.01 | 0.03 | 0.16 | 0.13 | 0.16 | 0.13 | 500 |
| 10 | 3 | 100 | 100 | 0 | 0.2 | 2.5 | 0.5 | 1 | 22.8 | 22.8 | 23.0 | 22.8 | 0.01 | 0.05 | 0.01 | 0.05 | 0.17 | 0.13 | 0.17 | 0.13 | 500 |
| 10 | 3 | 100 | 100 | 0 | 0.2 | 2.5 | 0.5 | 2 | 21.2 | 20.6 | 21.2 | 20.6 | 0.01 | 0.05 | 0.01 | 0.05 | 0.16 | 0.13 | 0.17 | 0.13 | 500 |
| 10 | 3 | 100 | 100 | 0 | 0.5 | 1.5 | 2 | 1 | 86.6 | 86.2 | 86.6 | 86.2 | 0.00 | 0.10 | 0.00 | 0.10 | 0.16 | 0.14 | 0.16 | 0.14 | 500 |
| 10 | 3 | 100 | 100 | 0 | 0.5 | 1.5 | 2 | 2 | 87.2 | 86.6 | 87.2 | 86.4 | 0.01 | 0.09 | 0.02 | 0.08 | 0.18 | 0.15 | 0.18 | 0.15 | 500 |
| 10 | 3 | 100 | 100 | 0 | 0.5 | 2.5 | 0.5 | 1 | 87.6 | 87.2 | 88.0 | 87.2 | 0.00 | 0.10 | 0.00 | 0.10 | 0.16 | 0.13 | 0.16 | 0.13 | 500 |
| 10 | 3 | 100 | 100 | 0 | 0.5 | 2.5 | 0.5 | 2 | 87.6 | 87.4 | 87.8 | 87.4 | 0.00 | 0.10 | 0.01 | 0.09 | 0.17 | 0.14 | 0.17 | 0.14 | 500 |
| 10 | 3 | 100 | 100 | 0.5 | 0 | 1.5 | 2 | 1 | 7.2 | 7.0 | 7.2 | 7.0 | 0.01 | 0.00 | 0.01 | 0.00 | 0.17 | 0.13 | 0.17 | 0.13 | 500 |
| 10 | 3 | 100 | 100 | 0.5 | 0 | 1.5 | 2 | 2 | 5.2 | 5.2 | 5.2 | 5.2 | 0.00 | 0.00 | 0.00 | 0.00 | 0.16 | 0.13 | 0.16 | 0.13 | 500 |
| 10 | 3 | 100 | 100 | 0.5 | 0 | 2.5 | 0.5 | 1 | 3.8 | 3.8 | 3.8 | 3.8 | 0.01 | 0.01 | 0.01 | 0.01 | 0.15 | 0.12 | 0.15 | 0.12 | 500 |
| 10 | 3 | 100 | 100 | 0.5 | 0 | 2.5 | 0.5 | 2 | 5.4 | 5.4 | 5.6 | 5.4 | 0.01 | 0.00 | 0.01 | 0.00 | 0.17 | 0.13 | 0.17 | 0.13 | 500 |
| 10 | 3 | 100 | 100 | 0.5 | 0.2 | 1.5 | 2 | 1 | 26.8 | 26.0 | 27.0 | 26.0 | 0.01 | 0.04 | 0.01 | 0.04 | 0.17 | 0.13 | 0.17 | 0.13 | 500 |
| 10 | 3 | 100 | 100 | 0.5 | 0.2 | 1.5 | 2 | 2 | 22.6 | 21.6 | 22.6 | 21.6 | 0.01 | 0.05 | 0.00 | 0.05 | 0.16 | 0.13 | 0.16 | 0.13 | 500 |
| 10 | 3 | 100 | 100 | 0.5 | 0.2 | 2.5 | 0.5 | 1 | 25.6 | 25.2 | 25.6 | 25.0 | 0.00 | 0.04 | 0.00 | 0.04 | 0.18 | 0.14 | 0.18 | 0.14 | 500 |
| 10 | 3 | 100 | 100 | 0.5 | 0.2 | 2.5 | 0.5 | 2 | 25.8 | 25.8 | 26.0 | 25.8 | 0.01 | 0.04 | 0.01 | 0.04 | 0.17 | 0.13 | 0.17 | 0.13 | 500 |
| 10 | 3 | 100 | 100 | 0.5 | 0.5 | 1.5 | 2 | 1 | 87.4 | 87.4 | 87.4 | 87.4 | 0.00 | 0.10 | 0.00 | 0.10 | 0.16 | 0.14 | 0.17 | 0.14 | 500 |
| 10 | 3 | 100 | 100 | 0.5 | 0.5 | 1.5 | 2 | 2 | 88.6 | 87.8 | 88.8 | 87.8 | 0.01 | 0.10 | 0.01 | 0.09 | 0.15 | 0.13 | 0.16 | 0.13 | 500 |
| 10 | 3 | 100 | 100 | 0.5 | 0.5 | 2.5 | 0.5 | 1 | 85.8 | 85.4 | 85.6 | 85.2 | 0.00 | 0.11 | 0.00 | 0.11 | 0.16 | 0.13 | 0.16 | 0.13 | 500 |
| 10 | 3 | 100 | 100 | 0.5 | 0.5 | 2.5 | 0.5 | 2 | 89.8 | 89.2 | 89.8 | 89.2 | 0.00 | 0.10 | 0.01 | 0.10 | 0.16 | 0.13 | 0.16 | 0.13 | 500 |
| 10 | 3 | 100 | 100 | 2 | 0 | 1.5 | 2 | 1 | 3.0 | 3.0 | 3.0 | 3.0 | 0.01 | 0.00 | 0.01 | 0.00 | 0.16 | 0.11 | 0.16 | 0.11 | 500 |
| 10 | 3 | 100 | 100 | 2 | 0 | 1.5 | 2 | 2 | 4.6 | 4.4 | 4.6 | 4.4 | 0.02 | 0.01 | 0.02 | 0.01 | 0.17 | 0.12 | 0.18 | 0.12 | 500 |
| 10 | 3 | 100 | 100 | 2 | 0 | 2.5 | 0.5 | 1 | 7.0 | 6.8 | 7.0 | 6.6 | 0.01 | 0.00 | 0.01 | 0.00 | 0.18 | 0.12 | 0.18 | 0.12 | 500 |
| 10 | 3 | 100 | 100 | 2 | 0 | 2.5 | 0.5 | 2 | 7.6 | 7.2 | 7.6 | 7.0 | 0.01 | 0.00 | 0.01 | 0.00 | 0.17 | 0.12 | 0.18 | 0.12 | 500 |
| 10 | 3 | 100 | 100 | 2 | 0.2 | 1.5 | 2 | 1 | 22.0 | 21.6 | 22.0 | 22.0 | 0.00 | 0.07 | 0.00 | 0.07 | 0.18 | 0.12 | 0.18 | 0.12 | 500 |
| 10 | 3 | 100 | 100 | 2 | 0.2 | 1.5 | 2 | 2 | 21.4 | 21.2 | 21.8 | 21.2 | 0.01 | 0.06 | 0.01 | 0.06 | 0.17 | 0.12 | 0.18 | 0.12 | 500 |
| 10 | 3 | 100 | 100 | 2 | 0.2 | 2.5 | 0.5 | 1 | 23.4 | 23.2 | 23.4 | 23.2 | 0.00 | 0.06 | 0.01 | 0.06 | 0.18 | 0.12 | 0.18 | 0.13 | 500 |
| 10 | 3 | 100 | 100 | 2 | 0.2 | 2.5 | 0.5 | 2 | 19.2 | 19.0 | 19.4 | 19.0 | 0.01 | 0.07 | 0.01 | 0.07 | 0.17 | 0.12 | 0.17 | 0.12 | 500 |
| 10 | 3 | 100 | 100 | 2 | 0.5 | 1.5 | 2 | 1 | 82.4 | 82.2 | 82.4 | 82.4 | 0.01 | 0.16 | 0.02 | 0.15 | 0.18 | 0.13 | 0.18 | 0.13 | 500 |
| 10 | 3 | 100 | 100 | 2 | 0.5 | 1.5 | 2 | 2 | 81.0 | 81.0 | 81.4 | 80.8 | 0.00 | 0.16 | 0.01 | 0.15 | 0.18 | 0.12 | 0.18 | 0.13 | 500 |
| 10 | 3 | 100 | 100 | 2 | 0.5 | 2.5 | 0.5 | 1 | 86.4 | 85.8 | 86.6 | 86.0 | 0.01 | 0.15 | 0.02 | 0.14 | 0.17 | 0.12 | 0.17 | 0.12 | 500 |
| 10 | 3 | 100 | 100 | 2 | 0.5 | 2.5 | 0.5 | 2 | 82.8 | 82.2 | 83.0 | 82.0 | 0.01 | 0.15 | 0.01 | 0.15 | 0.18 | 0.13 | 0.18 | 0.13 | 500 |
| 10 | 3 | 100 | 250 | 0 | 0 | 1.5 | 2 | 1 | 5.2 | 5.0 | 5.2 | 4.8 | 0.00 | 0.00 | 0.00 | 0.00 | 0.16 | 0.13 | 0.16 | 0.13 | 500 |
| 10 | 3 | 100 | 250 | 0 | 0 | 1.5 | 2 | 2 | 3.8 | 3.8 | 3.8 | 3.6 | 0.00 | 0.00 | 0.00 | 0.00 | 0.16 | 0.12 | 0.16 | 0.12 | 500 |
| 10 | 3 | 100 | 250 | 0 | 0 | 2.5 | 0.5 | 1 | 6.2 | 6.2 | 6.2 | 6.2 | 0.01 | 0.01 | 0.01 | 0.01 | 0.17 | 0.13 | 0.17 | 0.13 | 500 |
| 10 | 3 | 100 | 250 | 0 | 0 | 2.5 | 0.5 | 2 | 4.2 | 3.8 | 4.4 | 3.8 | 0.00 | 0.00 | 0.00 | 0.00 | 0.16 | 0.12 | 0.16 | 0.12 | 500 |
| 10 | 3 | 100 | 250 | 0 | 0.2 | 1.5 | 2 | 1 | 24.2 | 23.2 | 25.2 | 23.2 | 0.00 | 0.04 | 0.01 | 0.04 | 0.16 | 0.13 | 0.16 | 0.13 | 500 |
| 10 | 3 | 100 | 250 | 0 | 0.2 | 1.5 | 2 | 2 | 24.2 | 24.2 | 24.4 | 24.2 | 0.01 | 0.03 | 0.01 | 0.03 | 0.16 | 0.13 | 0.16 | 0.13 | 500 |
| 10 | 3 | 100 | 250 | 0 | 0.2 | 2.5 | 0.5 | 1 | 24.8 | 23.8 | 24.8 | 23.8 | 0.00 | 0.05 | 0.00 | 0.05 | 0.17 | 0.13 | 0.16 | 0.13 | 500 |
| 10 | 3 | 100 | 250 | 0 | 0.2 | 2.5 | 0.5 | 2 | 23.4 | 23.2 | 23.6 | 23.2 | 0.00 | 0.05 | 0.00 | 0.05 | 0.16 | 0.13 | 0.16 | 0.13 | 500 |
| 10 | 3 | 100 | 250 | 0 | 0.5 | 1.5 | 2 | 1 | 87.6 | 87.4 | 87.6 | 87.4 | 0.00 | 0.10 | 0.00 | 0.10 | 0.16 | 0.13 | 0.16 | 0.13 | 500 |
| 10 | 3 | 100 | 250 | 0 | 0.5 | 1.5 | 2 | 2 | 88.2 | 88.2 | 88.4 | 88.2 | 0.01 | 0.09 | 0.01 | 0.09 | 0.17 | 0.14 | 0.17 | 0.14 | 500 |
| 10 | 3 | 100 | 250 | 0 | 0.5 | 2.5 | 0.5 | 1 | 84.8 | 84.6 | 84.8 | 84.6 | 0.00 | 0.11 | 0.00 | 0.11 | 0.16 | 0.13 | 0.16 | 0.13 | 500 |
| 10 | 3 | 100 | 250 | 0 | 0.5 | 2.5 | 0.5 | 2 | 88.2 | 88.0 | 88.2 | 88.0 | 0.01 | 0.09 | 0.01 | 0.09 | 0.16 | 0.13 | 0.16 | 0.13 | 500 |
| 10 | 3 | 100 | 250 | 0.5 | 0 | 1.5 | 2 | 1 | 4.2 | 3.8 | 4.2 | 3.8 | 0.00 | 0.00 | 0.00 | 0.00 | 0.16 | 0.12 | 0.16 | 0.12 | 500 |
| 10 | 3 | 100 | 250 | 0.5 | 0 | 1.5 | 2 | 2 | 5.2 | 4.8 | 5.0 | 5.0 | 0.01 | 0.00 | 0.01 | 0.00 | 0.16 | 0.13 | 0.16 | 0.13 | 500 |
| 10 | 3 | 100 | 250 | 0.5 | 0 | 2.5 | 0.5 | 1 | 3.2 | 2.8 | 3.2 | 2.8 | 0.00 | 0.00 | 0.00 | 0.00 | 0.15 | 0.12 | 0.15 | 0.12 | 500 |
| 10 | 3 | 100 | 250 | 0.5 | 0 | 2.5 | 0.5 | 2 | 4.6 | 4.6 | 4.8 | 4.6 | 0.00 | 0.00 | 0.00 | 0.00 | 0.16 | 0.12 | 0.16 | 0.12 | 500 |
| 10 | 3 | 100 | 250 | 0.5 | 0.2 | 1.5 | 2 | 1 | 23.8 | 23.0 | 24.6 | 23.2 | 0.00 | 0.04 | 0.00 | 0.04 | 0.15 | 0.12 | 0.15 | 0.12 | 500 |
| 10 | 3 | 100 | 250 | 0.5 | 0.2 | 1.5 | 2 | 2 | 21.0 | 20.2 | 21.4 | 20.0 | 0.01 | 0.05 | 0.01 | 0.05 | 0.16 | 0.13 | 0.16 | 0.13 | 500 |
| 10 | 3 | 100 | 250 | 0.5 | 0.2 | 2.5 | 0.5 | 1 | 27.8 | 26.8 | 28.0 | 26.8 | 0.01 | 0.04 | 0.01 | 0.04 | 0.17 | 0.13 | 0.17 | 0.13 | 500 |
| 10 | 3 | 100 | 250 | 0.5 | 0.2 | 2.5 | 0.5 | 2 | 25.8 | 24.8 | 25.8 | 24.6 | 0.00 | 0.05 | 0.00 | 0.05 | 0.16 | 0.13 | 0.16 | 0.13 | 500 |
| 10 | 3 | 100 | 250 | 0.5 | 0.5 | 1.5 | 2 | 1 | 88.8 | 88.8 | 88.8 | 88.8 | 0.00 | 0.10 | 0.00 | 0.10 | 0.16 | 0.13 | 0.16 | 0.13 | 500 |
| 10 | 3 | 100 | 250 | 0.5 | 0.5 | 1.5 | 2 | 2 | 90.6 | 90.2 | 90.6 | 90.0 | 0.02 | 0.08 | 0.02 | 0.08 | 0.16 | 0.13 | 0.16 | 0.13 | 500 |
| 10 | 3 | 100 | 250 | 0.5 | 0.5 | 2.5 | 0.5 | 1 | 86.0 | 85.6 | 86.0 | 85.6 | 0.00 | 0.10 | 0.01 | 0.10 | 0.17 | 0.14 | 0.17 | 0.14 | 500 |
| 10 | 3 | 100 | 250 | 0.5 | 0.5 | 2.5 | 0.5 | 2 | 88.0 | 88.0 | 88.2 | 88.0 | 0.01 | 0.10 | 0.01 | 0.10 | 0.16 | 0.13 | 0.16 | 0.13 | 500 |
| 10 | 3 | 100 | 250 | 2 | 0 | 1.5 | 2 | 1 | 5.0 | 4.8 | 5.0 | 4.8 | 0.00 | 0.00 | 0.00 | 0.00 | 0.18 | 0.12 | 0.18 | 0.12 | 500 |
| 10 | 3 | 100 | 250 | 2 | 0 | 1.5 | 2 | 2 | 3.8 | 3.4 | 3.8 | 3.4 | 0.01 | 0.01 | 0.01 | 0.01 | 0.18 | 0.12 | 0.18 | 0.12 | 500 |
| 10 | 3 | 100 | 250 | 2 | 0 | 2.5 | 0.5 | 1 | 4.2 | 4.2 | 4.2 | 4.2 | 0.01 | 0.01 | 0.01 | 0.01 | 0.17 | 0.12 | 0.17 | 0.12 | 500 |
| 10 | 3 | 100 | 250 | 2 | 0 | 2.5 | 0.5 | 2 | 5.6 | 5.4 | 5.6 | 5.4 | 0.00 | 0.00 | 0.00 | 0.00 | 0.17 | 0.12 | 0.17 | 0.12 | 500 |
| 10 | 3 | 100 | 250 | 2 | 0.2 | 1.5 | 2 | 1 | 23.8 | 23.4 | 23.8 | 23.4 | 0.01 | 0.06 | 0.01 | 0.06 | 0.17 | 0.12 | 0.17 | 0.12 | 500 |
| 10 | 3 | 100 | 250 | 2 | 0.2 | 1.5 | 2 | 2 | 22.8 | 22.2 | 23.0 | 22.4 | 0.00 | 0.06 | 0.00 | 0.06 | 0.18 | 0.12 | 0.18 | 0.12 | 500 |
| 10 | 3 | 100 | 250 | 2 | 0.2 | 2.5 | 0.5 | 1 | 21.4 | 21.2 | 21.4 | 21.0 | 0.01 | 0.06 | 0.01 | 0.06 | 0.17 | 0.11 | 0.17 | 0.11 | 500 |
| 10 | 3 | 100 | 250 | 2 | 0.2 | 2.5 | 0.5 | 2 | 22.4 | 21.6 | 22.4 | 21.6 | 0.01 | 0.06 | 0.01 | 0.06 | 0.17 | 0.12 | 0.17 | 0.12 | 500 |
| 10 | 3 | 100 | 250 | 2 | 0.5 | 1.5 | 2 | 1 | 82.4 | 81.6 | 82.4 | 81.6 | 0.00 | 0.16 | 0.01 | 0.16 | 0.17 | 0.12 | 0.17 | 0.12 | 500 |
| 10 | 3 | 100 | 250 | 2 | 0.5 | 1.5 | 2 | 2 | 81.0 | 80.8 | 81.0 | 80.8 | 0.00 | 0.16 | 0.00 | 0.16 | 0.18 | 0.13 | 0.18 | 0.13 | 500 |
| 10 | 3 | 100 | 250 | 2 | 0.5 | 2.5 | 0.5 | 1 | 83.4 | 82.6 | 83.4 | 82.4 | 0.00 | 0.16 | 0.00 | 0.16 | 0.17 | 0.12 | 0.17 | 0.12 | 500 |
| 10 | 3 | 100 | 250 | 2 | 0.5 | 2.5 | 0.5 | 2 | 84.0 | 83.4 | 84.0 | 83.4 | 0.01 | 0.15 | 0.01 | 0.15 | 0.18 | 0.13 | 0.18 | 0.13 | 500 |
| 10 | 3 | 100 | 500 | 0 | 0 | 1.5 | 2 | 1 | 5.2 | 4.6 | 5.4 | 4.6 | 0.01 | 0.01 | 0.01 | 0.00 | 0.16 | 0.12 | 0.15 | 0.12 | 500 |
| 10 | 3 | 100 | 500 | 0 | 0 | 1.5 | 2 | 2 | 3.6 | 3.2 | 3.8 | 3.2 | 0.01 | 0.00 | 0.01 | 0.00 | 0.16 | 0.12 | 0.15 | 0.12 | 500 |
| 10 | 3 | 100 | 500 | 0 | 0 | 2.5 | 0.5 | 1 | 4.6 | 4.4 | 4.6 | 4.4 | 0.00 | 0.00 | 0.00 | 0.00 | 0.16 | 0.12 | 0.15 | 0.12 | 500 |
| 10 | 3 | 100 | 500 | 0 | 0 | 2.5 | 0.5 | 2 | 4.0 | 3.8 | 4.2 | 3.6 | 0.00 | 0.00 | 0.00 | 0.00 | 0.16 | 0.12 | 0.16 | 0.12 | 500 |
| 10 | 3 | 100 | 500 | 0 | 0.2 | 1.5 | 2 | 1 | 26.6 | 26.0 | 26.8 | 26.2 | 0.01 | 0.03 | 0.01 | 0.03 | 0.15 | 0.12 | 0.15 | 0.12 | 500 |
| 10 | 3 | 100 | 500 | 0 | 0.2 | 1.5 | 2 | 2 | 24.4 | 24.4 | 24.6 | 24.4 | 0.01 | 0.04 | 0.00 | 0.04 | 0.15 | 0.12 | 0.15 | 0.12 | 500 |
| 10 | 3 | 100 | 500 | 0 | 0.2 | 2.5 | 0.5 | 1 | 25.4 | 25.2 | 25.4 | 25.2 | 0.01 | 0.04 | 0.01 | 0.04 | 0.17 | 0.13 | 0.17 | 0.13 | 500 |
| 10 | 3 | 100 | 500 | 0 | 0.2 | 2.5 | 0.5 | 2 | 25.4 | 24.4 | 25.4 | 24.4 | 0.01 | 0.04 | 0.01 | 0.04 | 0.16 | 0.12 | 0.16 | 0.12 | 500 |
| 10 | 3 | 100 | 500 | 0 | 0.5 | 1.5 | 2 | 1 | 91.2 | 90.8 | 91.2 | 90.8 | 0.01 | 0.09 | 0.01 | 0.09 | 0.15 | 0.12 | 0.15 | 0.12 | 500 |
| 10 | 3 | 100 | 500 | 0 | 0.5 | 1.5 | 2 | 2 | 88.0 | 88.0 | 88.0 | 87.8 | 0.00 | 0.10 | 0.01 | 0.10 | 0.16 | 0.14 | 0.16 | 0.14 | 500 |
| 10 | 3 | 100 | 500 | 0 | 0.5 | 2.5 | 0.5 | 1 | 86.6 | 86.4 | 86.6 | 86.4 | 0.00 | 0.10 | 0.00 | 0.11 | 0.16 | 0.13 | 0.16 | 0.13 | 500 |
| 10 | 3 | 100 | 500 | 0 | 0.5 | 2.5 | 0.5 | 2 | 84.8 | 84.8 | 84.8 | 84.8 | 0.02 | 0.12 | 0.02 | 0.12 | 0.17 | 0.14 | 0.16 | 0.13 | 500 |
| 10 | 3 | 100 | 500 | 0.5 | 0 | 1.5 | 2 | 1 | 5.0 | 4.6 | 5.0 | 4.6 | 0.00 | 0.00 | 0.00 | 0.00 | 0.16 | 0.13 | 0.16 | 0.13 | 500 |
| 10 | 3 | 100 | 500 | 0.5 | 0 | 1.5 | 2 | 2 | 5.8 | 5.4 | 5.8 | 5.2 | 0.01 | 0.01 | 0.01 | 0.01 | 0.16 | 0.12 | 0.16 | 0.12 | 500 |
| 10 | 3 | 100 | 500 | 0.5 | 0 | 2.5 | 0.5 | 1 | 4.4 | 4.4 | 4.4 | 4.4 | 0.00 | 0.00 | 0.00 | 0.00 | 0.16 | 0.12 | 0.16 | 0.12 | 500 |
| 10 | 3 | 100 | 500 | 0.5 | 0 | 2.5 | 0.5 | 2 | 5.0 | 4.8 | 5.0 | 5.0 | 0.00 | 0.00 | 0.00 | 0.00 | 0.16 | 0.13 | 0.16 | 0.13 | 500 |
| 10 | 3 | 100 | 500 | 0.5 | 0.2 | 1.5 | 2 | 1 | 25.2 | 24.8 | 25.2 | 24.8 | 0.00 | 0.04 | 0.00 | 0.04 | 0.17 | 0.13 | 0.16 | 0.13 | 500 |
| 10 | 3 | 100 | 500 | 0.5 | 0.2 | 1.5 | 2 | 2 | 22.0 | 21.8 | 22.0 | 21.8 | 0.00 | 0.04 | 0.00 | 0.04 | 0.16 | 0.13 | 0.16 | 0.12 | 500 |
| 10 | 3 | 100 | 500 | 0.5 | 0.2 | 2.5 | 0.5 | 1 | 26.4 | 26.0 | 26.4 | 26.0 | 0.01 | 0.04 | 0.01 | 0.04 | 0.16 | 0.12 | 0.16 | 0.12 | 500 |
| 10 | 3 | 100 | 500 | 0.5 | 0.2 | 2.5 | 0.5 | 2 | 27.0 | 26.2 | 27.0 | 26.2 | 0.01 | 0.04 | 0.01 | 0.04 | 0.18 | 0.14 | 0.17 | 0.14 | 500 |
| 10 | 3 | 100 | 500 | 0.5 | 0.5 | 1.5 | 2 | 1 | 88.8 | 88.4 | 88.8 | 88.4 | 0.01 | 0.09 | 0.01 | 0.10 | 0.16 | 0.14 | 0.16 | 0.13 | 500 |
| 10 | 3 | 100 | 500 | 0.5 | 0.5 | 1.5 | 2 | 2 | 84.6 | 84.4 | 84.6 | 84.4 | 0.02 | 0.12 | 0.02 | 0.12 | 0.16 | 0.13 | 0.16 | 0.13 | 500 |
| 10 | 3 | 100 | 500 | 0.5 | 0.5 | 2.5 | 0.5 | 1 | 87.8 | 87.4 | 88.0 | 87.4 | 0.00 | 0.11 | 0.01 | 0.11 | 0.16 | 0.13 | 0.16 | 0.13 | 500 |
| 10 | 3 | 100 | 500 | 0.5 | 0.5 | 2.5 | 0.5 | 2 | 87.0 | 86.4 | 87.0 | 86.4 | 0.00 | 0.10 | 0.00 | 0.11 | 0.16 | 0.13 | 0.16 | 0.13 | 500 |
| 10 | 3 | 100 | 500 | 2 | 0 | 1.5 | 2 | 1 | 5.8 | 5.6 | 5.8 | 5.6 | 0.01 | 0.00 | 0.00 | 0.00 | 0.18 | 0.12 | 0.18 | 0.12 | 500 |
| 10 | 3 | 100 | 500 | 2 | 0 | 1.5 | 2 | 2 | 3.4 | 3.4 | 3.4 | 3.4 | 0.01 | 0.01 | 0.01 | 0.01 | 0.17 | 0.12 | 0.17 | 0.11 | 500 |
| 10 | 3 | 100 | 500 | 2 | 0 | 2.5 | 0.5 | 1 | 4.0 | 3.8 | 4.0 | 3.8 | 0.00 | 0.00 | 0.00 | 0.00 | 0.17 | 0.11 | 0.17 | 0.11 | 500 |
| 10 | 3 | 100 | 500 | 2 | 0 | 2.5 | 0.5 | 2 | 4.6 | 4.4 | 5.0 | 4.4 | 0.00 | 0.00 | 0.00 | 0.00 | 0.17 | 0.12 | 0.17 | 0.12 | 500 |
| 10 | 3 | 100 | 500 | 2 | 0.2 | 1.5 | 2 | 1 | 22.2 | 21.6 | 22.4 | 21.6 | 0.01 | 0.06 | 0.01 | 0.06 | 0.17 | 0.12 | 0.17 | 0.12 | 500 |
| 10 | 3 | 100 | 500 | 2 | 0.2 | 1.5 | 2 | 2 | 20.0 | 19.2 | 20.2 | 19.2 | 0.00 | 0.06 | 0.00 | 0.06 | 0.17 | 0.11 | 0.17 | 0.11 | 500 |
| 10 | 3 | 100 | 500 | 2 | 0.2 | 2.5 | 0.5 | 1 | 19.8 | 19.0 | 19.8 | 19.0 | 0.00 | 0.07 | 0.00 | 0.07 | 0.17 | 0.12 | 0.17 | 0.12 | 500 |
| 10 | 3 | 100 | 500 | 2 | 0.2 | 2.5 | 0.5 | 2 | 20.8 | 19.8 | 21.0 | 19.8 | 0.00 | 0.07 | 0.00 | 0.07 | 0.17 | 0.12 | 0.17 | 0.12 | 500 |
| 10 | 3 | 100 | 500 | 2 | 0.5 | 1.5 | 2 | 1 | 80.2 | 79.6 | 80.2 | 79.4 | 0.01 | 0.17 | 0.01 | 0.17 | 0.17 | 0.12 | 0.17 | 0.12 | 500 |
| 10 | 3 | 100 | 500 | 2 | 0.5 | 1.5 | 2 | 2 | 82.2 | 81.6 | 82.4 | 81.6 | 0.01 | 0.16 | 0.01 | 0.16 | 0.18 | 0.13 | 0.18 | 0.13 | 500 |
| 10 | 3 | 100 | 500 | 2 | 0.5 | 2.5 | 0.5 | 1 | 84.2 | 83.2 | 84.2 | 83.2 | 0.01 | 0.15 | 0.01 | 0.15 | 0.17 | 0.12 | 0.17 | 0.12 | 500 |
| 10 | 3 | 100 | 500 | 2 | 0.5 | 2.5 | 0.5 | 2 | 84.4 | 84.2 | 84.4 | 84.2 | 0.01 | 0.15 | 0.01 | 0.15 | 0.16 | 0.12 | 0.17 | 0.12 | 500 |
| 10 | 3 | 200 | 100 | 0 | 0 | 1.5 | 2 | 1 | 4.0 | 4.0 | 4.2 | 3.8 | 0.01 | 0.00 | 0.01 | 0.01 | 0.11 | 0.09 | 0.11 | 0.09 | 500 |
| 10 | 3 | 200 | 100 | 0 | 0 | 1.5 | 2 | 2 | 5.0 | 4.8 | 5.0 | 4.6 | 0.00 | 0.00 | 0.00 | 0.00 | 0.11 | 0.09 | 0.11 | 0.09 | 500 |
| 10 | 3 | 200 | 100 | 0 | 0 | 2.5 | 0.5 | 1 | 3.4 | 3.4 | 3.4 | 3.4 | 0.00 | 0.00 | 0.00 | 0.00 | 0.11 | 0.08 | 0.11 | 0.08 | 500 |
| 10 | 3 | 200 | 100 | 0 | 0 | 2.5 | 0.5 | 2 | 5.6 | 5.6 | 5.6 | 5.6 | 0.00 | 0.00 | 0.00 | 0.00 | 0.11 | 0.09 | 0.12 | 0.09 | 500 |
| 10 | 3 | 200 | 100 | 0 | 0.2 | 1.5 | 2 | 1 | 45.4 | 44.6 | 44.8 | 44.4 | 0.00 | 0.04 | 0.00 | 0.04 | 0.11 | 0.09 | 0.11 | 0.09 | 500 |
| 10 | 3 | 200 | 100 | 0 | 0.2 | 1.5 | 2 | 2 | 44.6 | 44.2 | 44.4 | 44.0 | 0.00 | 0.04 | 0.00 | 0.04 | 0.11 | 0.09 | 0.11 | 0.09 | 500 |
| 10 | 3 | 200 | 100 | 0 | 0.2 | 2.5 | 0.5 | 1 | 42.4 | 42.2 | 42.4 | 42.0 | 0.00 | 0.05 | 0.00 | 0.04 | 0.11 | 0.09 | 0.11 | 0.09 | 500 |
| 10 | 3 | 200 | 100 | 0 | 0.2 | 2.5 | 0.5 | 2 | 47.6 | 47.6 | 47.6 | 47.4 | 0.01 | 0.03 | 0.02 | 0.03 | 0.11 | 0.09 | 0.11 | 0.09 | 500 |
| 10 | 3 | 200 | 100 | 0 | 0.5 | 1.5 | 2 | 1 | 99.6 | 99.6 | 99.6 | 99.6 | 0.00 | 0.10 | 0.00 | 0.10 | 0.11 | 0.09 | 0.11 | 0.10 | 500 |
| 10 | 3 | 200 | 100 | 0 | 0.5 | 1.5 | 2 | 2 | 99.8 | 99.8 | 99.8 | 99.8 | 0.01 | 0.09 | 0.01 | 0.09 | 0.11 | 0.09 | 0.11 | 0.10 | 500 |
| 10 | 3 | 200 | 100 | 0 | 0.5 | 2.5 | 0.5 | 1 | 99.4 | 99.4 | 99.4 | 99.4 | 0.01 | 0.11 | 0.00 | 0.10 | 0.11 | 0.09 | 0.12 | 0.09 | 500 |
| 10 | 3 | 200 | 100 | 0 | 0.5 | 2.5 | 0.5 | 2 | 99.0 | 99.0 | 99.0 | 99.0 | 0.01 | 0.10 | 0.01 | 0.09 | 0.11 | 0.09 | 0.11 | 0.09 | 500 |
| 10 | 3 | 200 | 100 | 0.5 | 0 | 1.5 | 2 | 1 | 7.0 | 7.0 | 7.0 | 7.0 | 0.00 | 0.00 | 0.00 | 0.00 | 0.12 | 0.09 | 0.12 | 0.09 | 500 |
| 10 | 3 | 200 | 100 | 0.5 | 0 | 1.5 | 2 | 2 | 6.2 | 6.2 | 6.2 | 6.0 | 0.01 | 0.01 | 0.01 | 0.01 | 0.12 | 0.09 | 0.12 | 0.10 | 500 |
| 10 | 3 | 200 | 100 | 0.5 | 0 | 2.5 | 0.5 | 1 | 6.6 | 6.4 | 6.8 | 6.4 | 0.00 | 0.00 | 0.00 | 0.00 | 0.12 | 0.09 | 0.12 | 0.09 | 500 |
| 10 | 3 | 200 | 100 | 0.5 | 0 | 2.5 | 0.5 | 2 | 4.6 | 4.4 | 4.6 | 4.4 | 0.01 | 0.01 | 0.01 | 0.01 | 0.11 | 0.08 | 0.11 | 0.09 | 500 |
| 10 | 3 | 200 | 100 | 0.5 | 0.2 | 1.5 | 2 | 1 | 42.0 | 41.8 | 42.0 | 41.8 | 0.00 | 0.04 | 0.00 | 0.04 | 0.11 | 0.09 | 0.11 | 0.09 | 500 |
| 10 | 3 | 200 | 100 | 0.5 | 0.2 | 1.5 | 2 | 2 | 42.6 | 42.4 | 42.6 | 42.6 | 0.00 | 0.04 | 0.00 | 0.04 | 0.12 | 0.09 | 0.12 | 0.09 | 500 |
| 10 | 3 | 200 | 100 | 0.5 | 0.2 | 2.5 | 0.5 | 1 | 46.2 | 46.0 | 46.2 | 46.0 | 0.01 | 0.04 | 0.01 | 0.03 | 0.11 | 0.09 | 0.11 | 0.09 | 500 |
| 10 | 3 | 200 | 100 | 0.5 | 0.2 | 2.5 | 0.5 | 2 | 40.0 | 39.8 | 40.0 | 39.8 | 0.00 | 0.05 | 0.00 | 0.04 | 0.11 | 0.09 | 0.11 | 0.09 | 500 |
| 10 | 3 | 200 | 100 | 0.5 | 0.5 | 1.5 | 2 | 1 | 100.0 | 100.0 | 100.0 | 100.0 | 0.01 | 0.09 | 0.02 | 0.09 | 0.11 | 0.09 | 0.11 | 0.09 | 500 |
| 10 | 3 | 200 | 100 | 0.5 | 0.5 | 1.5 | 2 | 2 | 99.2 | 99.2 | 99.2 | 99.2 | 0.00 | 0.10 | 0.01 | 0.10 | 0.11 | 0.09 | 0.12 | 0.10 | 500 |
| 10 | 3 | 200 | 100 | 0.5 | 0.5 | 2.5 | 0.5 | 1 | 98.8 | 98.8 | 98.8 | 98.8 | 0.00 | 0.11 | 0.00 | 0.10 | 0.11 | 0.09 | 0.11 | 0.09 | 500 |
| 10 | 3 | 200 | 100 | 0.5 | 0.5 | 2.5 | 0.5 | 2 | 99.6 | 99.6 | 99.6 | 99.6 | 0.00 | 0.10 | 0.01 | 0.10 | 0.11 | 0.09 | 0.11 | 0.09 | 500 |
| 10 | 3 | 200 | 100 | 2 | 0 | 1.5 | 2 | 1 | 6.2 | 6.2 | 6.2 | 6.2 | 0.01 | 0.00 | 0.01 | 0.01 | 0.13 | 0.08 | 0.13 | 0.09 | 500 |
| 10 | 3 | 200 | 100 | 2 | 0 | 1.5 | 2 | 2 | 4.4 | 4.2 | 4.4 | 4.4 | 0.01 | 0.01 | 0.01 | 0.01 | 0.12 | 0.08 | 0.12 | 0.08 | 500 |
| 10 | 3 | 200 | 100 | 2 | 0 | 2.5 | 0.5 | 1 | 6.4 | 6.4 | 6.4 | 6.4 | 0.00 | 0.00 | 0.00 | 0.00 | 0.13 | 0.09 | 0.13 | 0.09 | 500 |
| 10 | 3 | 200 | 100 | 2 | 0 | 2.5 | 0.5 | 2 | 5.2 | 5.2 | 5.2 | 5.2 | 0.01 | 0.00 | 0.01 | 0.00 | 0.12 | 0.08 | 0.12 | 0.08 | 500 |
| 10 | 3 | 200 | 100 | 2 | 0.2 | 1.5 | 2 | 1 | 35.6 | 35.4 | 35.6 | 35.6 | 0.00 | 0.06 | 0.01 | 0.06 | 0.12 | 0.08 | 0.12 | 0.08 | 500 |
| 10 | 3 | 200 | 100 | 2 | 0.2 | 1.5 | 2 | 2 | 35.0 | 34.8 | 35.0 | 34.8 | 0.00 | 0.07 | 0.00 | 0.06 | 0.13 | 0.08 | 0.13 | 0.09 | 500 |
| 10 | 3 | 200 | 100 | 2 | 0.2 | 2.5 | 0.5 | 1 | 41.4 | 41.4 | 41.4 | 41.4 | 0.01 | 0.06 | 0.01 | 0.06 | 0.12 | 0.08 | 0.12 | 0.08 | 500 |
| 10 | 3 | 200 | 100 | 2 | 0.2 | 2.5 | 0.5 | 2 | 38.6 | 38.4 | 38.6 | 38.6 | 0.00 | 0.06 | 0.00 | 0.06 | 0.12 | 0.08 | 0.12 | 0.08 | 500 |
| 10 | 3 | 200 | 100 | 2 | 0.5 | 1.5 | 2 | 1 | 97.8 | 97.8 | 97.8 | 97.8 | 0.00 | 0.16 | 0.01 | 0.15 | 0.13 | 0.09 | 0.13 | 0.09 | 500 |
| 10 | 3 | 200 | 100 | 2 | 0.5 | 1.5 | 2 | 2 | 98.6 | 98.6 | 98.6 | 98.6 | 0.00 | 0.16 | 0.01 | 0.15 | 0.12 | 0.09 | 0.13 | 0.09 | 500 |
| 10 | 3 | 200 | 100 | 2 | 0.5 | 2.5 | 0.5 | 1 | 99.0 | 99.0 | 99.0 | 99.0 | 0.00 | 0.16 | 0.00 | 0.15 | 0.12 | 0.09 | 0.12 | 0.09 | 500 |
| 10 | 3 | 200 | 100 | 2 | 0.5 | 2.5 | 0.5 | 2 | 97.6 | 97.6 | 97.6 | 97.6 | 0.00 | 0.16 | 0.01 | 0.15 | 0.12 | 0.09 | 0.12 | 0.09 | 500 |
| 10 | 3 | 200 | 250 | 0 | 0 | 1.5 | 2 | 1 | 4.2 | 4.2 | 4.2 | 4.2 | 0.00 | 0.00 | 0.00 | 0.00 | 0.11 | 0.09 | 0.11 | 0.09 | 500 |
| 10 | 3 | 200 | 250 | 0 | 0 | 1.5 | 2 | 2 | 6.8 | 6.8 | 7.0 | 6.8 | 0.01 | 0.01 | 0.01 | 0.01 | 0.11 | 0.09 | 0.11 | 0.09 | 500 |
| 10 | 3 | 200 | 250 | 0 | 0 | 2.5 | 0.5 | 1 | 6.0 | 6.0 | 6.0 | 6.0 | 0.00 | 0.00 | 0.00 | 0.00 | 0.12 | 0.09 | 0.12 | 0.09 | 500 |
| 10 | 3 | 200 | 250 | 0 | 0 | 2.5 | 0.5 | 2 | 4.4 | 4.4 | 4.4 | 4.4 | 0.00 | 0.00 | 0.00 | 0.00 | 0.11 | 0.09 | 0.11 | 0.09 | 500 |
| 10 | 3 | 200 | 250 | 0 | 0.2 | 1.5 | 2 | 1 | 42.6 | 42.4 | 42.6 | 42.2 | 0.00 | 0.04 | 0.00 | 0.04 | 0.11 | 0.09 | 0.12 | 0.09 | 500 |
| 10 | 3 | 200 | 250 | 0 | 0.2 | 1.5 | 2 | 2 | 44.4 | 44.0 | 44.4 | 44.0 | 0.00 | 0.04 | 0.00 | 0.04 | 0.12 | 0.09 | 0.11 | 0.09 | 500 |
| 10 | 3 | 200 | 250 | 0 | 0.2 | 2.5 | 0.5 | 1 | 43.8 | 43.4 | 43.6 | 43.2 | 0.00 | 0.04 | 0.00 | 0.04 | 0.11 | 0.09 | 0.11 | 0.09 | 500 |
| 10 | 3 | 200 | 250 | 0 | 0.2 | 2.5 | 0.5 | 2 | 43.4 | 43.4 | 43.6 | 43.4 | 0.00 | 0.04 | 0.00 | 0.04 | 0.11 | 0.08 | 0.11 | 0.08 | 500 |
| 10 | 3 | 200 | 250 | 0 | 0.5 | 1.5 | 2 | 1 | 99.0 | 99.0 | 99.0 | 99.0 | 0.00 | 0.10 | 0.00 | 0.09 | 0.12 | 0.10 | 0.12 | 0.10 | 500 |
| 10 | 3 | 200 | 250 | 0 | 0.5 | 1.5 | 2 | 2 | 99.8 | 99.8 | 99.8 | 99.8 | 0.01 | 0.09 | 0.01 | 0.09 | 0.11 | 0.09 | 0.11 | 0.10 | 500 |
| 10 | 3 | 200 | 250 | 0 | 0.5 | 2.5 | 0.5 | 1 | 99.2 | 99.2 | 99.2 | 99.2 | 0.00 | 0.10 | 0.00 | 0.10 | 0.11 | 0.09 | 0.11 | 0.09 | 500 |
| 10 | 3 | 200 | 250 | 0 | 0.5 | 2.5 | 0.5 | 2 | 99.2 | 99.2 | 99.2 | 99.2 | 0.00 | 0.11 | 0.00 | 0.10 | 0.11 | 0.09 | 0.11 | 0.09 | 500 |
| 10 | 3 | 200 | 250 | 0.5 | 0 | 1.5 | 2 | 1 | 5.2 | 5.2 | 5.2 | 5.2 | 0.00 | 0.00 | 0.00 | 0.00 | 0.11 | 0.09 | 0.11 | 0.09 | 500 |
| 10 | 3 | 200 | 250 | 0.5 | 0 | 1.5 | 2 | 2 | 5.6 | 5.4 | 5.6 | 5.4 | 0.00 | 0.00 | 0.00 | 0.00 | 0.11 | 0.09 | 0.11 | 0.09 | 500 |
| 10 | 3 | 200 | 250 | 0.5 | 0 | 2.5 | 0.5 | 1 | 4.2 | 4.2 | 4.2 | 4.2 | 0.00 | 0.00 | 0.00 | 0.00 | 0.11 | 0.09 | 0.11 | 0.09 | 500 |
| 10 | 3 | 200 | 250 | 0.5 | 0 | 2.5 | 0.5 | 2 | 6.2 | 6.2 | 6.2 | 6.2 | 0.01 | 0.01 | 0.01 | 0.01 | 0.11 | 0.09 | 0.11 | 0.09 | 500 |
| 10 | 3 | 200 | 250 | 0.5 | 0.2 | 1.5 | 2 | 1 | 42.0 | 41.6 | 42.2 | 41.6 | 0.00 | 0.04 | 0.00 | 0.04 | 0.11 | 0.09 | 0.11 | 0.09 | 500 |
| 10 | 3 | 200 | 250 | 0.5 | 0.2 | 1.5 | 2 | 2 | 45.0 | 44.4 | 45.0 | 44.4 | 0.00 | 0.04 | 0.00 | 0.04 | 0.12 | 0.09 | 0.12 | 0.09 | 500 |
| 10 | 3 | 200 | 250 | 0.5 | 0.2 | 2.5 | 0.5 | 1 | 41.8 | 41.4 | 42.0 | 41.4 | 0.00 | 0.05 | 0.00 | 0.05 | 0.11 | 0.09 | 0.11 | 0.09 | 500 |
| 10 | 3 | 200 | 250 | 0.5 | 0.2 | 2.5 | 0.5 | 2 | 45.0 | 44.4 | 45.0 | 44.4 | 0.00 | 0.04 | 0.00 | 0.04 | 0.12 | 0.09 | 0.12 | 0.09 | 500 |
| 10 | 3 | 200 | 250 | 0.5 | 0.5 | 1.5 | 2 | 1 | 99.0 | 99.0 | 99.0 | 99.0 | 0.01 | 0.10 | 0.01 | 0.10 | 0.11 | 0.09 | 0.11 | 0.09 | 500 |
| 10 | 3 | 200 | 250 | 0.5 | 0.5 | 1.5 | 2 | 2 | 99.6 | 99.6 | 99.6 | 99.6 | 0.00 | 0.10 | 0.01 | 0.10 | 0.11 | 0.09 | 0.11 | 0.09 | 500 |
| 10 | 3 | 200 | 250 | 0.5 | 0.5 | 2.5 | 0.5 | 1 | 99.2 | 99.2 | 99.2 | 99.2 | 0.00 | 0.11 | 0.00 | 0.11 | 0.12 | 0.10 | 0.12 | 0.10 | 500 |
| 10 | 3 | 200 | 250 | 0.5 | 0.5 | 2.5 | 0.5 | 2 | 99.6 | 99.6 | 99.6 | 99.6 | 0.01 | 0.11 | 0.00 | 0.11 | 0.11 | 0.09 | 0.11 | 0.09 | 500 |
| 10 | 3 | 200 | 250 | 2 | 0 | 1.5 | 2 | 1 | 4.0 | 3.6 | 4.0 | 3.6 | 0.01 | 0.01 | 0.01 | 0.01 | 0.12 | 0.08 | 0.12 | 0.08 | 500 |
| 10 | 3 | 200 | 250 | 2 | 0 | 1.5 | 2 | 2 | 4.6 | 4.4 | 4.6 | 4.6 | 0.01 | 0.01 | 0.01 | 0.01 | 0.12 | 0.08 | 0.12 | 0.08 | 500 |
| 10 | 3 | 200 | 250 | 2 | 0 | 2.5 | 0.5 | 1 | 5.4 | 5.2 | 5.4 | 5.2 | 0.00 | 0.00 | 0.00 | 0.00 | 0.13 | 0.09 | 0.13 | 0.09 | 500 |
| 10 | 3 | 200 | 250 | 2 | 0 | 2.5 | 0.5 | 2 | 5.6 | 5.4 | 5.6 | 5.2 | 0.00 | 0.00 | 0.00 | 0.00 | 0.12 | 0.08 | 0.13 | 0.08 | 500 |
| 10 | 3 | 200 | 250 | 2 | 0.2 | 1.5 | 2 | 1 | 39.2 | 39.0 | 39.2 | 39.0 | 0.00 | 0.07 | 0.00 | 0.07 | 0.13 | 0.08 | 0.13 | 0.09 | 500 |
| 10 | 3 | 200 | 250 | 2 | 0.2 | 1.5 | 2 | 2 | 41.6 | 41.4 | 41.6 | 41.4 | 0.02 | 0.06 | 0.02 | 0.06 | 0.13 | 0.09 | 0.13 | 0.09 | 500 |
| 10 | 3 | 200 | 250 | 2 | 0.2 | 2.5 | 0.5 | 1 | 34.2 | 33.8 | 34.2 | 33.8 | 0.01 | 0.07 | 0.01 | 0.07 | 0.13 | 0.09 | 0.13 | 0.09 | 500 |
| 10 | 3 | 200 | 250 | 2 | 0.2 | 2.5 | 0.5 | 2 | 37.4 | 37.0 | 37.4 | 37.0 | 0.00 | 0.07 | 0.00 | 0.07 | 0.12 | 0.08 | 0.12 | 0.09 | 500 |
| 10 | 3 | 200 | 250 | 2 | 0.5 | 1.5 | 2 | 1 | 98.6 | 98.6 | 98.6 | 98.6 | 0.01 | 0.16 | 0.01 | 0.16 | 0.12 | 0.09 | 0.13 | 0.09 | 500 |
| 10 | 3 | 200 | 250 | 2 | 0.5 | 1.5 | 2 | 2 | 98.8 | 98.6 | 98.8 | 98.6 | 0.01 | 0.17 | 0.01 | 0.17 | 0.12 | 0.09 | 0.12 | 0.09 | 500 |
| 10 | 3 | 200 | 250 | 2 | 0.5 | 2.5 | 0.5 | 1 | 99.0 | 99.0 | 99.0 | 99.0 | 0.01 | 0.15 | 0.01 | 0.15 | 0.12 | 0.09 | 0.12 | 0.08 | 500 |
| 10 | 3 | 200 | 250 | 2 | 0.5 | 2.5 | 0.5 | 2 | 98.4 | 98.4 | 98.4 | 98.4 | 0.01 | 0.15 | 0.01 | 0.15 | 0.12 | 0.09 | 0.12 | 0.09 | 500 |
| 10 | 3 | 200 | 500 | 0 | 0 | 1.5 | 2 | 1 | 4.8 | 4.8 | 4.8 | 4.8 | 0.00 | 0.00 | 0.00 | 0.00 | 0.11 | 0.09 | 0.11 | 0.09 | 500 |
| 10 | 3 | 200 | 500 | 0 | 0 | 1.5 | 2 | 2 | 5.2 | 5.2 | 5.2 | 5.2 | 0.01 | 0.01 | 0.01 | 0.01 | 0.12 | 0.09 | 0.12 | 0.09 | 500 |
| 10 | 3 | 200 | 500 | 0 | 0 | 2.5 | 0.5 | 1 | 3.2 | 3.2 | 3.4 | 3.0 | 0.01 | 0.01 | 0.01 | 0.01 | 0.11 | 0.09 | 0.11 | 0.09 | 500 |
| 10 | 3 | 200 | 500 | 0 | 0 | 2.5 | 0.5 | 2 | 7.0 | 7.0 | 7.0 | 7.0 | 0.00 | 0.00 | 0.00 | 0.00 | 0.12 | 0.09 | 0.12 | 0.09 | 500 |
| 10 | 3 | 200 | 500 | 0 | 0.2 | 1.5 | 2 | 1 | 44.4 | 44.2 | 44.2 | 44.2 | 0.00 | 0.04 | 0.00 | 0.04 | 0.12 | 0.09 | 0.12 | 0.09 | 500 |
| 10 | 3 | 200 | 500 | 0 | 0.2 | 1.5 | 2 | 2 | 41.4 | 41.2 | 41.4 | 41.2 | 0.00 | 0.04 | 0.00 | 0.04 | 0.12 | 0.10 | 0.12 | 0.10 | 500 |
| 10 | 3 | 200 | 500 | 0 | 0.2 | 2.5 | 0.5 | 1 | 44.4 | 44.0 | 44.6 | 44.2 | 0.01 | 0.04 | 0.01 | 0.04 | 0.12 | 0.09 | 0.12 | 0.09 | 500 |
| 10 | 3 | 200 | 500 | 0 | 0.2 | 2.5 | 0.5 | 2 | 43.6 | 43.6 | 43.6 | 43.6 | 0.00 | 0.04 | 0.00 | 0.04 | 0.12 | 0.10 | 0.12 | 0.10 | 500 |
| 10 | 3 | 200 | 500 | 0 | 0.5 | 1.5 | 2 | 1 | 99.4 | 99.4 | 99.4 | 99.4 | 0.00 | 0.10 | 0.00 | 0.10 | 0.11 | 0.09 | 0.11 | 0.09 | 500 |
| 10 | 3 | 200 | 500 | 0 | 0.5 | 1.5 | 2 | 2 | 98.6 | 98.6 | 98.6 | 98.6 | 0.00 | 0.10 | 0.00 | 0.10 | 0.11 | 0.09 | 0.11 | 0.09 | 500 |
| 10 | 3 | 200 | 500 | 0 | 0.5 | 2.5 | 0.5 | 1 | 99.6 | 99.6 | 99.6 | 99.6 | 0.01 | 0.10 | 0.00 | 0.10 | 0.11 | 0.09 | 0.11 | 0.09 | 500 |
| 10 | 3 | 200 | 500 | 0 | 0.5 | 2.5 | 0.5 | 2 | 99.2 | 99.2 | 99.2 | 99.2 | 0.00 | 0.11 | 0.00 | 0.11 | 0.11 | 0.09 | 0.11 | 0.09 | 500 |
| 10 | 3 | 200 | 500 | 0.5 | 0 | 1.5 | 2 | 1 | 3.8 | 3.6 | 3.8 | 3.8 | 0.01 | 0.00 | 0.01 | 0.00 | 0.11 | 0.08 | 0.11 | 0.09 | 500 |
| 10 | 3 | 200 | 500 | 0.5 | 0 | 1.5 | 2 | 2 | 5.8 | 5.8 | 5.8 | 5.8 | 0.00 | 0.00 | 0.00 | 0.00 | 0.12 | 0.09 | 0.12 | 0.09 | 500 |
| 10 | 3 | 200 | 500 | 0.5 | 0 | 2.5 | 0.5 | 1 | 3.8 | 3.8 | 3.8 | 3.8 | 0.01 | 0.01 | 0.01 | 0.01 | 0.11 | 0.09 | 0.11 | 0.09 | 500 |
| 10 | 3 | 200 | 500 | 0.5 | 0 | 2.5 | 0.5 | 2 | 5.8 | 5.6 | 5.8 | 5.6 | 0.00 | 0.00 | 0.00 | 0.00 | 0.12 | 0.09 | 0.12 | 0.09 | 500 |
| 10 | 3 | 200 | 500 | 0.5 | 0.2 | 1.5 | 2 | 1 | 44.8 | 44.2 | 44.8 | 44.2 | 0.00 | 0.04 | 0.00 | 0.04 | 0.12 | 0.09 | 0.12 | 0.09 | 500 |
| 10 | 3 | 200 | 500 | 0.5 | 0.2 | 1.5 | 2 | 2 | 45.6 | 45.0 | 45.6 | 45.2 | 0.01 | 0.04 | 0.01 | 0.04 | 0.12 | 0.09 | 0.12 | 0.09 | 500 |
| 10 | 3 | 200 | 500 | 0.5 | 0.2 | 2.5 | 0.5 | 1 | 46.4 | 45.6 | 46.4 | 45.6 | 0.01 | 0.04 | 0.01 | 0.04 | 0.11 | 0.09 | 0.11 | 0.09 | 500 |
| 10 | 3 | 200 | 500 | 0.5 | 0.2 | 2.5 | 0.5 | 2 | 46.2 | 45.6 | 46.2 | 45.6 | 0.01 | 0.04 | 0.01 | 0.04 | 0.11 | 0.09 | 0.11 | 0.09 | 500 |
| 10 | 3 | 200 | 500 | 0.5 | 0.5 | 1.5 | 2 | 1 | 99.0 | 99.0 | 99.0 | 99.0 | 0.01 | 0.11 | 0.01 | 0.11 | 0.11 | 0.09 | 0.11 | 0.09 | 500 |
| 10 | 3 | 200 | 500 | 0.5 | 0.5 | 1.5 | 2 | 2 | 99.4 | 99.4 | 99.4 | 99.4 | 0.00 | 0.10 | 0.00 | 0.10 | 0.11 | 0.09 | 0.11 | 0.09 | 500 |
| 10 | 3 | 200 | 500 | 0.5 | 0.5 | 2.5 | 0.5 | 1 | 99.8 | 99.8 | 99.8 | 99.8 | 0.01 | 0.10 | 0.01 | 0.10 | 0.11 | 0.09 | 0.11 | 0.09 | 500 |
| 10 | 3 | 200 | 500 | 0.5 | 0.5 | 2.5 | 0.5 | 2 | 98.6 | 98.6 | 98.6 | 98.6 | 0.00 | 0.10 | 0.00 | 0.10 | 0.12 | 0.10 | 0.12 | 0.09 | 500 |
| 10 | 3 | 200 | 500 | 2 | 0 | 1.5 | 2 | 1 | 5.4 | 5.4 | 5.4 | 5.4 | 0.01 | 0.00 | 0.01 | 0.00 | 0.12 | 0.08 | 0.12 | 0.08 | 500 |
| 10 | 3 | 200 | 500 | 2 | 0 | 1.5 | 2 | 2 | 4.4 | 4.2 | 4.4 | 4.2 | 0.01 | 0.01 | 0.01 | 0.01 | 0.12 | 0.08 | 0.12 | 0.08 | 500 |
| 10 | 3 | 200 | 500 | 2 | 0 | 2.5 | 0.5 | 1 | 5.4 | 5.2 | 5.4 | 5.4 | 0.00 | 0.00 | 0.00 | 0.00 | 0.12 | 0.08 | 0.12 | 0.08 | 500 |
| 10 | 3 | 200 | 500 | 2 | 0 | 2.5 | 0.5 | 2 | 5.6 | 5.4 | 5.6 | 5.6 | 0.01 | 0.01 | 0.01 | 0.01 | 0.12 | 0.08 | 0.12 | 0.08 | 500 |
| 10 | 3 | 200 | 500 | 2 | 0.2 | 1.5 | 2 | 1 | 34.6 | 33.8 | 34.8 | 33.8 | 0.00 | 0.07 | 0.00 | 0.07 | 0.12 | 0.08 | 0.12 | 0.08 | 500 |
| 10 | 3 | 200 | 500 | 2 | 0.2 | 1.5 | 2 | 2 | 35.2 | 35.2 | 35.2 | 35.2 | 0.00 | 0.07 | 0.00 | 0.07 | 0.12 | 0.08 | 0.12 | 0.08 | 500 |
| 10 | 3 | 200 | 500 | 2 | 0.2 | 2.5 | 0.5 | 1 | 36.2 | 36.2 | 36.2 | 36.2 | 0.01 | 0.07 | 0.01 | 0.07 | 0.12 | 0.08 | 0.12 | 0.08 | 500 |
| 10 | 3 | 200 | 500 | 2 | 0.2 | 2.5 | 0.5 | 2 | 37.4 | 37.0 | 37.4 | 37.2 | 0.01 | 0.07 | 0.01 | 0.07 | 0.12 | 0.08 | 0.12 | 0.08 | 500 |
| 10 | 3 | 200 | 500 | 2 | 0.5 | 1.5 | 2 | 1 | 98.0 | 98.0 | 98.0 | 98.0 | 0.00 | 0.16 | 0.00 | 0.16 | 0.13 | 0.09 | 0.12 | 0.09 | 500 |
| 10 | 3 | 200 | 500 | 2 | 0.5 | 1.5 | 2 | 2 | 97.8 | 97.8 | 97.8 | 97.8 | 0.00 | 0.16 | 0.01 | 0.16 | 0.13 | 0.09 | 0.13 | 0.09 | 500 |
| 10 | 3 | 200 | 500 | 2 | 0.5 | 2.5 | 0.5 | 1 | 98.4 | 98.4 | 98.4 | 98.4 | 0.01 | 0.15 | 0.01 | 0.15 | 0.12 | 0.09 | 0.12 | 0.09 | 500 |
| 10 | 3 | 200 | 500 | 2 | 0.5 | 2.5 | 0.5 | 2 | 99.2 | 99.2 | 99.2 | 99.2 | 0.01 | 0.15 | 0.01 | 0.15 | 0.12 | 0.09 | 0.12 | 0.09 | 500 |
| 10 | 3 | 500 | 100 | 0 | 0 | 1.5 | 2 | 1 | 4.6 | 4.6 | 4.6 | 4.6 | 0.00 | 0.00 | 0.00 | 0.00 | 0.07 | 0.06 | 0.07 | 0.06 | 500 |
| 10 | 3 | 500 | 100 | 0 | 0 | 1.5 | 2 | 2 | 5.0 | 5.0 | 5.0 | 5.0 | 0.00 | 0.00 | 0.00 | 0.00 | 0.07 | 0.06 | 0.08 | 0.06 | 500 |
| 10 | 3 | 500 | 100 | 0 | 0 | 2.5 | 0.5 | 1 | 4.8 | 4.6 | 4.8 | 4.4 | 0.00 | 0.00 | 0.00 | 0.00 | 0.07 | 0.06 | 0.07 | 0.06 | 500 |
| 10 | 3 | 500 | 100 | 0 | 0 | 2.5 | 0.5 | 2 | 6.0 | 6.0 | 6.0 | 6.0 | 0.00 | 0.00 | 0.00 | 0.00 | 0.07 | 0.06 | 0.07 | 0.06 | 500 |
| 10 | 3 | 500 | 100 | 0 | 0.2 | 1.5 | 2 | 1 | 79.6 | 79.4 | 79.6 | 79.6 | 0.00 | 0.04 | 0.00 | 0.04 | 0.07 | 0.05 | 0.07 | 0.06 | 500 |
| 10 | 3 | 500 | 100 | 0 | 0.2 | 1.5 | 2 | 2 | 80.0 | 80.0 | 80.0 | 80.0 | 0.00 | 0.04 | 0.00 | 0.04 | 0.07 | 0.06 | 0.07 | 0.06 | 500 |
| 10 | 3 | 500 | 100 | 0 | 0.2 | 2.5 | 0.5 | 1 | 80.6 | 80.6 | 80.6 | 80.6 | 0.00 | 0.04 | 0.01 | 0.04 | 0.07 | 0.06 | 0.07 | 0.06 | 500 |
| 10 | 3 | 500 | 100 | 0 | 0.2 | 2.5 | 0.5 | 2 | 82.2 | 82.0 | 82.2 | 82.0 | 0.00 | 0.04 | 0.01 | 0.04 | 0.07 | 0.05 | 0.07 | 0.06 | 500 |
| 10 | 3 | 500 | 100 | 0 | 0.5 | 1.5 | 2 | 1 | 100.0 | 100.0 | 100.0 | 100.0 | 0.01 | 0.09 | 0.01 | 0.09 | 0.07 | 0.06 | 0.08 | 0.06 | 500 |
| 10 | 3 | 500 | 100 | 0 | 0.5 | 1.5 | 2 | 2 | 100.0 | 100.0 | 100.0 | 100.0 | 0.00 | 0.10 | 0.01 | 0.09 | 0.08 | 0.06 | 0.08 | 0.07 | 500 |
| 10 | 3 | 500 | 100 | 0 | 0.5 | 2.5 | 0.5 | 1 | 100.0 | 100.0 | 100.0 | 100.0 | 0.00 | 0.10 | 0.01 | 0.10 | 0.07 | 0.06 | 0.07 | 0.06 | 500 |
| 10 | 3 | 500 | 100 | 0 | 0.5 | 2.5 | 0.5 | 2 | 100.0 | 100.0 | 100.0 | 100.0 | 0.00 | 0.10 | 0.01 | 0.09 | 0.08 | 0.06 | 0.08 | 0.07 | 500 |
| 10 | 3 | 500 | 100 | 0.5 | 0 | 1.5 | 2 | 1 | 5.8 | 5.8 | 6.0 | 5.8 | 0.01 | 0.01 | 0.01 | 0.01 | 0.07 | 0.06 | 0.07 | 0.06 | 500 |
| 10 | 3 | 500 | 100 | 0.5 | 0 | 1.5 | 2 | 2 | 5.0 | 5.0 | 5.0 | 5.0 | 0.00 | 0.00 | 0.00 | 0.00 | 0.07 | 0.06 | 0.07 | 0.06 | 500 |
| 10 | 3 | 500 | 100 | 0.5 | 0 | 2.5 | 0.5 | 1 | 7.8 | 7.8 | 7.8 | 7.8 | 0.00 | 0.00 | 0.00 | 0.00 | 0.08 | 0.06 | 0.08 | 0.06 | 500 |
| 10 | 3 | 500 | 100 | 0.5 | 0 | 2.5 | 0.5 | 2 | 3.6 | 3.6 | 3.6 | 3.6 | 0.00 | 0.00 | 0.00 | 0.00 | 0.07 | 0.05 | 0.07 | 0.05 | 500 |
| 10 | 3 | 500 | 100 | 0.5 | 0.2 | 1.5 | 2 | 1 | 78.8 | 78.8 | 78.8 | 78.8 | 0.00 | 0.05 | 0.00 | 0.04 | 0.07 | 0.06 | 0.07 | 0.06 | 500 |
| 10 | 3 | 500 | 100 | 0.5 | 0.2 | 1.5 | 2 | 2 | 78.8 | 78.6 | 78.8 | 78.8 | 0.00 | 0.04 | 0.00 | 0.04 | 0.07 | 0.06 | 0.07 | 0.06 | 500 |
| 10 | 3 | 500 | 100 | 0.5 | 0.2 | 2.5 | 0.5 | 1 | 81.0 | 81.0 | 81.0 | 81.0 | 0.00 | 0.04 | 0.01 | 0.04 | 0.07 | 0.05 | 0.07 | 0.06 | 500 |
| 10 | 3 | 500 | 100 | 0.5 | 0.2 | 2.5 | 0.5 | 2 | 77.2 | 77.0 | 77.2 | 77.0 | 0.00 | 0.05 | 0.00 | 0.04 | 0.07 | 0.06 | 0.08 | 0.06 | 500 |
| 10 | 3 | 500 | 100 | 0.5 | 0.5 | 1.5 | 2 | 1 | 100.0 | 100.0 | 100.0 | 100.0 | 0.00 | 0.10 | 0.01 | 0.09 | 0.07 | 0.06 | 0.07 | 0.06 | 500 |
| 10 | 3 | 500 | 100 | 0.5 | 0.5 | 1.5 | 2 | 2 | 100.0 | 100.0 | 100.0 | 100.0 | 0.01 | 0.10 | 0.01 | 0.09 | 0.07 | 0.06 | 0.08 | 0.06 | 500 |
| 10 | 3 | 500 | 100 | 0.5 | 0.5 | 2.5 | 0.5 | 1 | 100.0 | 100.0 | 100.0 | 100.0 | 0.00 | 0.10 | 0.01 | 0.10 | 0.07 | 0.06 | 0.08 | 0.06 | 500 |
| 10 | 3 | 500 | 100 | 0.5 | 0.5 | 2.5 | 0.5 | 2 | 100.0 | 100.0 | 100.0 | 100.0 | 0.00 | 0.11 | 0.00 | 0.10 | 0.07 | 0.06 | 0.07 | 0.06 | 500 |
| 10 | 3 | 500 | 100 | 2 | 0 | 1.5 | 2 | 1 | 5.0 | 5.0 | 4.8 | 4.8 | 0.00 | 0.00 | 0.00 | 0.00 | 0.08 | 0.05 | 0.08 | 0.05 | 500 |
| 10 | 3 | 500 | 100 | 2 | 0 | 1.5 | 2 | 2 | 5.8 | 5.6 | 5.8 | 5.6 | 0.00 | 0.00 | 0.00 | 0.00 | 0.08 | 0.05 | 0.08 | 0.05 | 500 |
| 10 | 3 | 500 | 100 | 2 | 0 | 2.5 | 0.5 | 1 | 6.6 | 6.4 | 6.8 | 6.4 | 0.00 | 0.00 | 0.00 | 0.00 | 0.08 | 0.05 | 0.08 | 0.06 | 500 |
| 10 | 3 | 500 | 100 | 2 | 0 | 2.5 | 0.5 | 2 | 4.2 | 4.2 | 4.2 | 4.2 | 0.00 | 0.00 | 0.00 | 0.00 | 0.08 | 0.05 | 0.08 | 0.05 | 500 |
| 10 | 3 | 500 | 100 | 2 | 0.2 | 1.5 | 2 | 1 | 72.0 | 71.8 | 72.0 | 71.8 | 0.00 | 0.07 | 0.00 | 0.06 | 0.08 | 0.05 | 0.08 | 0.05 | 500 |
| 10 | 3 | 500 | 100 | 2 | 0.2 | 1.5 | 2 | 2 | 74.4 | 74.2 | 74.2 | 73.8 | 0.00 | 0.06 | 0.01 | 0.06 | 0.08 | 0.05 | 0.08 | 0.06 | 500 |
| 10 | 3 | 500 | 100 | 2 | 0.2 | 2.5 | 0.5 | 1 | 73.0 | 73.0 | 73.0 | 73.0 | 0.00 | 0.07 | 0.00 | 0.06 | 0.08 | 0.05 | 0.08 | 0.05 | 500 |
| 10 | 3 | 500 | 100 | 2 | 0.2 | 2.5 | 0.5 | 2 | 75.6 | 75.2 | 75.6 | 75.2 | 0.00 | 0.06 | 0.01 | 0.06 | 0.07 | 0.05 | 0.08 | 0.05 | 500 |
| 10 | 3 | 500 | 100 | 2 | 0.5 | 1.5 | 2 | 1 | 100.0 | 100.0 | 100.0 | 100.0 | 0.00 | 0.16 | 0.00 | 0.16 | 0.07 | 0.05 | 0.08 | 0.06 | 500 |
| 10 | 3 | 500 | 100 | 2 | 0.5 | 1.5 | 2 | 2 | 100.0 | 100.0 | 100.0 | 100.0 | 0.00 | 0.16 | 0.01 | 0.15 | 0.08 | 0.06 | 0.08 | 0.06 | 500 |
| 10 | 3 | 500 | 100 | 2 | 0.5 | 2.5 | 0.5 | 1 | 100.0 | 100.0 | 100.0 | 100.0 | 0.00 | 0.15 | 0.01 | 0.15 | 0.08 | 0.06 | 0.08 | 0.06 | 500 |
| 10 | 3 | 500 | 100 | 2 | 0.5 | 2.5 | 0.5 | 2 | 100.0 | 100.0 | 100.0 | 100.0 | 0.00 | 0.15 | 0.01 | 0.15 | 0.08 | 0.05 | 0.08 | 0.06 | 500 |
| 10 | 3 | 500 | 250 | 0 | 0 | 1.5 | 2 | 1 | 4.8 | 4.4 | 4.6 | 4.4 | 0.00 | 0.00 | 0.00 | 0.00 | 0.07 | 0.06 | 0.07 | 0.06 | 500 |
| 10 | 3 | 500 | 250 | 0 | 0 | 1.5 | 2 | 2 | 4.6 | 4.6 | 4.6 | 4.6 | 0.00 | 0.00 | 0.00 | 0.00 | 0.07 | 0.05 | 0.07 | 0.05 | 500 |
| 10 | 3 | 500 | 250 | 0 | 0 | 2.5 | 0.5 | 1 | 5.8 | 5.8 | 5.8 | 5.8 | 0.00 | 0.00 | 0.00 | 0.00 | 0.07 | 0.06 | 0.07 | 0.06 | 500 |
| 10 | 3 | 500 | 250 | 0 | 0 | 2.5 | 0.5 | 2 | 4.8 | 4.6 | 4.8 | 4.6 | 0.00 | 0.00 | 0.00 | 0.00 | 0.07 | 0.05 | 0.07 | 0.05 | 500 |
| 10 | 3 | 500 | 250 | 0 | 0.2 | 1.5 | 2 | 1 | 79.4 | 79.2 | 79.4 | 79.2 | 0.00 | 0.04 | 0.00 | 0.04 | 0.07 | 0.06 | 0.07 | 0.06 | 500 |
| 10 | 3 | 500 | 250 | 0 | 0.2 | 1.5 | 2 | 2 | 79.0 | 79.0 | 79.0 | 79.0 | 0.00 | 0.04 | 0.00 | 0.04 | 0.07 | 0.06 | 0.07 | 0.06 | 500 |
| 10 | 3 | 500 | 250 | 0 | 0.2 | 2.5 | 0.5 | 1 | 76.2 | 76.2 | 76.2 | 76.2 | 0.00 | 0.05 | 0.00 | 0.04 | 0.08 | 0.06 | 0.08 | 0.06 | 500 |
| 10 | 3 | 500 | 250 | 0 | 0.2 | 2.5 | 0.5 | 2 | 78.8 | 78.8 | 78.8 | 78.8 | 0.00 | 0.04 | 0.00 | 0.04 | 0.07 | 0.06 | 0.07 | 0.06 | 500 |
| 10 | 3 | 500 | 250 | 0 | 0.5 | 1.5 | 2 | 1 | 100.0 | 100.0 | 100.0 | 100.0 | 0.00 | 0.10 | 0.00 | 0.09 | 0.07 | 0.06 | 0.07 | 0.06 | 500 |
| 10 | 3 | 500 | 250 | 0 | 0.5 | 1.5 | 2 | 2 | 100.0 | 100.0 | 100.0 | 100.0 | 0.00 | 0.10 | 0.00 | 0.10 | 0.07 | 0.06 | 0.07 | 0.06 | 500 |
| 10 | 3 | 500 | 250 | 0 | 0.5 | 2.5 | 0.5 | 1 | 100.0 | 100.0 | 100.0 | 100.0 | 0.00 | 0.10 | 0.00 | 0.10 | 0.07 | 0.06 | 0.07 | 0.06 | 500 |
| 10 | 3 | 500 | 250 | 0 | 0.5 | 2.5 | 0.5 | 2 | 100.0 | 100.0 | 100.0 | 100.0 | 0.00 | 0.10 | 0.00 | 0.10 | 0.07 | 0.06 | 0.07 | 0.06 | 500 |
| 10 | 3 | 500 | 250 | 0.5 | 0 | 1.5 | 2 | 1 | 6.2 | 6.2 | 6.2 | 6.2 | 0.00 | 0.00 | 0.00 | 0.00 | 0.07 | 0.06 | 0.07 | 0.06 | 500 |
| 10 | 3 | 500 | 250 | 0.5 | 0 | 1.5 | 2 | 2 | 5.2 | 5.2 | 5.2 | 5.2 | 0.00 | 0.00 | 0.00 | 0.00 | 0.07 | 0.06 | 0.07 | 0.06 | 500 |
| 10 | 3 | 500 | 250 | 0.5 | 0 | 2.5 | 0.5 | 1 | 4.6 | 4.6 | 4.6 | 4.6 | 0.00 | 0.00 | 0.00 | 0.00 | 0.07 | 0.06 | 0.07 | 0.06 | 500 |
| 10 | 3 | 500 | 250 | 0.5 | 0 | 2.5 | 0.5 | 2 | 5.6 | 5.6 | 5.6 | 5.6 | 0.00 | 0.00 | 0.00 | 0.00 | 0.08 | 0.06 | 0.08 | 0.06 | 500 |
| 10 | 3 | 500 | 250 | 0.5 | 0.2 | 1.5 | 2 | 1 | 78.8 | 78.8 | 78.8 | 78.8 | 0.00 | 0.04 | 0.00 | 0.04 | 0.07 | 0.06 | 0.07 | 0.06 | 500 |
| 10 | 3 | 500 | 250 | 0.5 | 0.2 | 1.5 | 2 | 2 | 80.0 | 79.8 | 79.8 | 79.6 | 0.00 | 0.04 | 0.00 | 0.04 | 0.07 | 0.05 | 0.07 | 0.05 | 500 |
| 10 | 3 | 500 | 250 | 0.5 | 0.2 | 2.5 | 0.5 | 1 | 78.8 | 78.6 | 78.8 | 78.6 | 0.00 | 0.04 | 0.00 | 0.04 | 0.07 | 0.06 | 0.07 | 0.06 | 500 |
| 10 | 3 | 500 | 250 | 0.5 | 0.2 | 2.5 | 0.5 | 2 | 79.8 | 79.6 | 79.8 | 79.6 | 0.00 | 0.05 | 0.00 | 0.05 | 0.07 | 0.06 | 0.07 | 0.06 | 500 |
| 10 | 3 | 500 | 250 | 0.5 | 0.5 | 1.5 | 2 | 1 | 100.0 | 100.0 | 100.0 | 100.0 | 0.00 | 0.10 | 0.00 | 0.10 | 0.07 | 0.06 | 0.08 | 0.06 | 500 |
| 10 | 3 | 500 | 250 | 0.5 | 0.5 | 1.5 | 2 | 2 | 100.0 | 100.0 | 100.0 | 100.0 | 0.00 | 0.10 | 0.00 | 0.10 | 0.08 | 0.06 | 0.08 | 0.06 | 500 |
| 10 | 3 | 500 | 250 | 0.5 | 0.5 | 2.5 | 0.5 | 1 | 100.0 | 100.0 | 100.0 | 100.0 | 0.00 | 0.11 | 0.00 | 0.11 | 0.07 | 0.06 | 0.07 | 0.06 | 500 |
| 10 | 3 | 500 | 250 | 0.5 | 0.5 | 2.5 | 0.5 | 2 | 100.0 | 100.0 | 100.0 | 100.0 | 0.01 | 0.10 | 0.01 | 0.10 | 0.07 | 0.06 | 0.07 | 0.06 | 500 |
| 10 | 3 | 500 | 250 | 2 | 0 | 1.5 | 2 | 1 | 8.2 | 8.2 | 8.2 | 8.2 | 0.00 | 0.00 | 0.00 | 0.00 | 0.08 | 0.06 | 0.08 | 0.06 | 500 |
| 10 | 3 | 500 | 250 | 2 | 0 | 1.5 | 2 | 2 | 6.4 | 6.2 | 6.4 | 6.4 | 0.00 | 0.00 | 0.00 | 0.00 | 0.08 | 0.05 | 0.08 | 0.05 | 500 |
| 10 | 3 | 500 | 250 | 2 | 0 | 2.5 | 0.5 | 1 | 4.6 | 4.6 | 4.8 | 4.6 | 0.00 | 0.00 | 0.00 | 0.00 | 0.08 | 0.05 | 0.08 | 0.05 | 500 |
| 10 | 3 | 500 | 250 | 2 | 0 | 2.5 | 0.5 | 2 | 4.2 | 4.2 | 4.2 | 4.2 | 0.00 | 0.00 | 0.00 | 0.00 | 0.08 | 0.05 | 0.08 | 0.05 | 500 |
| 10 | 3 | 500 | 250 | 2 | 0.2 | 1.5 | 2 | 1 | 76.0 | 75.8 | 75.8 | 75.6 | 0.00 | 0.07 | 0.00 | 0.06 | 0.08 | 0.05 | 0.08 | 0.05 | 500 |
| 10 | 3 | 500 | 250 | 2 | 0.2 | 1.5 | 2 | 2 | 76.2 | 75.6 | 76.2 | 75.6 | 0.01 | 0.06 | 0.01 | 0.06 | 0.08 | 0.05 | 0.08 | 0.05 | 500 |
| 10 | 3 | 500 | 250 | 2 | 0.2 | 2.5 | 0.5 | 1 | 77.2 | 77.0 | 77.2 | 77.0 | 0.00 | 0.06 | 0.01 | 0.06 | 0.07 | 0.05 | 0.08 | 0.05 | 500 |
| 10 | 3 | 500 | 250 | 2 | 0.2 | 2.5 | 0.5 | 2 | 73.4 | 73.4 | 73.4 | 73.4 | 0.00 | 0.06 | 0.00 | 0.06 | 0.08 | 0.05 | 0.08 | 0.05 | 500 |
| 10 | 3 | 500 | 250 | 2 | 0.5 | 1.5 | 2 | 1 | 100.0 | 100.0 | 100.0 | 100.0 | 0.00 | 0.16 | 0.01 | 0.16 | 0.08 | 0.06 | 0.08 | 0.06 | 500 |
| 10 | 3 | 500 | 250 | 2 | 0.5 | 1.5 | 2 | 2 | 100.0 | 100.0 | 100.0 | 100.0 | 0.00 | 0.16 | 0.00 | 0.16 | 0.08 | 0.05 | 0.08 | 0.06 | 500 |
| 10 | 3 | 500 | 250 | 2 | 0.5 | 2.5 | 0.5 | 1 | 100.0 | 100.0 | 100.0 | 100.0 | 0.00 | 0.16 | 0.00 | 0.15 | 0.08 | 0.05 | 0.08 | 0.05 | 500 |
| 10 | 3 | 500 | 250 | 2 | 0.5 | 2.5 | 0.5 | 2 | 100.0 | 100.0 | 100.0 | 100.0 | 0.00 | 0.15 | 0.00 | 0.15 | 0.08 | 0.06 | 0.08 | 0.06 | 500 |
| 10 | 3 | 500 | 500 | 0 | 0 | 1.5 | 2 | 1 | 4.4 | 4.4 | 4.4 | 4.4 | 0.01 | 0.01 | 0.01 | 0.01 | 0.07 | 0.06 | 0.07 | 0.06 | 500 |
| 10 | 3 | 500 | 500 | 0 | 0 | 1.5 | 2 | 2 | 3.6 | 3.6 | 3.6 | 3.6 | 0.00 | 0.00 | 0.00 | 0.00 | 0.07 | 0.05 | 0.07 | 0.05 | 500 |
| 10 | 3 | 500 | 500 | 0 | 0 | 2.5 | 0.5 | 1 | 4.4 | 4.4 | 4.4 | 4.4 | 0.01 | 0.00 | 0.01 | 0.00 | 0.07 | 0.05 | 0.07 | 0.05 | 500 |
| 10 | 3 | 500 | 500 | 0 | 0 | 2.5 | 0.5 | 2 | 4.2 | 4.2 | 4.2 | 4.2 | 0.00 | 0.00 | 0.00 | 0.00 | 0.07 | 0.06 | 0.07 | 0.06 | 500 |
| 10 | 3 | 500 | 500 | 0 | 0.2 | 1.5 | 2 | 1 | 74.2 | 74.0 | 74.2 | 74.0 | 0.01 | 0.05 | 0.01 | 0.05 | 0.07 | 0.06 | 0.07 | 0.06 | 500 |
| 10 | 3 | 500 | 500 | 0 | 0.2 | 1.5 | 2 | 2 | 79.4 | 79.4 | 79.4 | 79.4 | 0.00 | 0.04 | 0.00 | 0.04 | 0.07 | 0.06 | 0.07 | 0.06 | 500 |
| 10 | 3 | 500 | 500 | 0 | 0.2 | 2.5 | 0.5 | 1 | 82.8 | 82.8 | 82.8 | 82.8 | 0.01 | 0.04 | 0.01 | 0.04 | 0.07 | 0.06 | 0.07 | 0.06 | 500 |
| 10 | 3 | 500 | 500 | 0 | 0.2 | 2.5 | 0.5 | 2 | 78.0 | 78.0 | 78.0 | 78.0 | 0.00 | 0.04 | 0.00 | 0.04 | 0.07 | 0.05 | 0.07 | 0.05 | 500 |
| 10 | 3 | 500 | 500 | 0 | 0.5 | 1.5 | 2 | 1 | 100.0 | 100.0 | 100.0 | 100.0 | 0.00 | 0.10 | 0.00 | 0.10 | 0.07 | 0.06 | 0.07 | 0.06 | 500 |
| 10 | 3 | 500 | 500 | 0 | 0.5 | 1.5 | 2 | 2 | 100.0 | 100.0 | 100.0 | 100.0 | 0.00 | 0.10 | 0.00 | 0.10 | 0.07 | 0.06 | 0.07 | 0.06 | 500 |
| 10 | 3 | 500 | 500 | 0 | 0.5 | 2.5 | 0.5 | 1 | 100.0 | 100.0 | 100.0 | 100.0 | 0.00 | 0.11 | 0.00 | 0.11 | 0.07 | 0.06 | 0.07 | 0.06 | 500 |
| 10 | 3 | 500 | 500 | 0 | 0.5 | 2.5 | 0.5 | 2 | 100.0 | 100.0 | 100.0 | 100.0 | 0.01 | 0.10 | 0.01 | 0.10 | 0.07 | 0.06 | 0.07 | 0.06 | 500 |
| 10 | 3 | 500 | 500 | 0.5 | 0 | 1.5 | 2 | 1 | 5.4 | 5.4 | 5.4 | 5.4 | 0.00 | 0.00 | 0.00 | 0.00 | 0.08 | 0.06 | 0.08 | 0.06 | 500 |
| 10 | 3 | 500 | 500 | 0.5 | 0 | 1.5 | 2 | 2 | 3.6 | 3.4 | 3.6 | 3.4 | 0.00 | 0.00 | 0.00 | 0.00 | 0.07 | 0.05 | 0.07 | 0.05 | 500 |
| 10 | 3 | 500 | 500 | 0.5 | 0 | 2.5 | 0.5 | 1 | 5.0 | 4.8 | 5.0 | 4.8 | 0.00 | 0.00 | 0.00 | 0.00 | 0.07 | 0.05 | 0.07 | 0.05 | 500 |
| 10 | 3 | 500 | 500 | 0.5 | 0 | 2.5 | 0.5 | 2 | 4.4 | 4.2 | 4.4 | 4.4 | 0.00 | 0.00 | 0.00 | 0.00 | 0.07 | 0.05 | 0.07 | 0.05 | 500 |
| 10 | 3 | 500 | 500 | 0.5 | 0.2 | 1.5 | 2 | 1 | 83.4 | 83.2 | 83.4 | 83.2 | 0.01 | 0.04 | 0.01 | 0.04 | 0.07 | 0.05 | 0.07 | 0.05 | 500 |
| 10 | 3 | 500 | 500 | 0.5 | 0.2 | 1.5 | 2 | 2 | 78.2 | 78.2 | 78.2 | 78.0 | 0.00 | 0.05 | 0.00 | 0.04 | 0.07 | 0.06 | 0.07 | 0.06 | 500 |
| 10 | 3 | 500 | 500 | 0.5 | 0.2 | 2.5 | 0.5 | 1 | 78.8 | 78.4 | 78.8 | 78.4 | 0.00 | 0.05 | 0.00 | 0.05 | 0.07 | 0.06 | 0.07 | 0.06 | 500 |
| 10 | 3 | 500 | 500 | 0.5 | 0.2 | 2.5 | 0.5 | 2 | 80.0 | 80.0 | 80.0 | 80.0 | 0.00 | 0.04 | 0.00 | 0.04 | 0.07 | 0.06 | 0.07 | 0.06 | 500 |
| 10 | 3 | 500 | 500 | 0.5 | 0.5 | 1.5 | 2 | 1 | 100.0 | 100.0 | 100.0 | 100.0 | 0.00 | 0.10 | 0.00 | 0.10 | 0.07 | 0.06 | 0.07 | 0.06 | 500 |
| 10 | 3 | 500 | 500 | 0.5 | 0.5 | 1.5 | 2 | 2 | 100.0 | 100.0 | 100.0 | 100.0 | 0.01 | 0.11 | 0.01 | 0.11 | 0.07 | 0.06 | 0.07 | 0.06 | 500 |
| 10 | 3 | 500 | 500 | 0.5 | 0.5 | 2.5 | 0.5 | 1 | 100.0 | 100.0 | 100.0 | 100.0 | 0.00 | 0.11 | 0.00 | 0.11 | 0.08 | 0.06 | 0.08 | 0.06 | 500 |
| 10 | 3 | 500 | 500 | 0.5 | 0.5 | 2.5 | 0.5 | 2 | 100.0 | 100.0 | 100.0 | 100.0 | 0.00 | 0.10 | 0.00 | 0.10 | 0.07 | 0.06 | 0.07 | 0.06 | 500 |
| 10 | 3 | 500 | 500 | 2 | 0 | 1.5 | 2 | 1 | 5.2 | 5.2 | 5.2 | 5.2 | 0.01 | 0.00 | 0.01 | 0.00 | 0.08 | 0.05 | 0.08 | 0.05 | 500 |
| 10 | 3 | 500 | 500 | 2 | 0 | 1.5 | 2 | 2 | 4.0 | 4.0 | 4.0 | 4.0 | 0.00 | 0.00 | 0.00 | 0.00 | 0.08 | 0.05 | 0.08 | 0.05 | 500 |
| 10 | 3 | 500 | 500 | 2 | 0 | 2.5 | 0.5 | 1 | 4.4 | 4.2 | 4.4 | 4.2 | 0.00 | 0.00 | 0.00 | 0.00 | 0.07 | 0.05 | 0.07 | 0.05 | 500 |
| 10 | 3 | 500 | 500 | 2 | 0 | 2.5 | 0.5 | 2 | 5.8 | 5.8 | 5.8 | 5.8 | 0.00 | 0.00 | 0.00 | 0.00 | 0.08 | 0.05 | 0.08 | 0.05 | 500 |
| 10 | 3 | 500 | 500 | 2 | 0.2 | 1.5 | 2 | 1 | 75.4 | 75.2 | 75.4 | 75.4 | 0.00 | 0.07 | 0.00 | 0.07 | 0.07 | 0.05 | 0.07 | 0.05 | 500 |
| 10 | 3 | 500 | 500 | 2 | 0.2 | 1.5 | 2 | 2 | 74.6 | 74.6 | 74.6 | 74.6 | 0.00 | 0.07 | 0.00 | 0.07 | 0.08 | 0.05 | 0.08 | 0.05 | 500 |
| 10 | 3 | 500 | 500 | 2 | 0.2 | 2.5 | 0.5 | 1 | 74.0 | 74.0 | 74.0 | 74.0 | 0.01 | 0.06 | 0.01 | 0.06 | 0.08 | 0.05 | 0.08 | 0.05 | 500 |
| 10 | 3 | 500 | 500 | 2 | 0.2 | 2.5 | 0.5 | 2 | 75.2 | 75.2 | 75.4 | 75.2 | 0.00 | 0.06 | 0.00 | 0.06 | 0.08 | 0.05 | 0.08 | 0.05 | 500 |
| 10 | 3 | 500 | 500 | 2 | 0.5 | 1.5 | 2 | 1 | 100.0 | 100.0 | 100.0 | 100.0 | 0.00 | 0.16 | 0.01 | 0.16 | 0.08 | 0.05 | 0.08 | 0.05 | 500 |
| 10 | 3 | 500 | 500 | 2 | 0.5 | 1.5 | 2 | 2 | 100.0 | 100.0 | 100.0 | 100.0 | 0.01 | 0.16 | 0.01 | 0.16 | 0.08 | 0.06 | 0.08 | 0.06 | 500 |
| 10 | 3 | 500 | 500 | 2 | 0.5 | 2.5 | 0.5 | 1 | 100.0 | 100.0 | 100.0 | 100.0 | 0.00 | 0.15 | 0.00 | 0.15 | 0.08 | 0.06 | 0.08 | 0.06 | 500 |
| 10 | 3 | 500 | 500 | 2 | 0.5 | 2.5 | 0.5 | 2 | 100.0 | 100.0 | 100.0 | 100.0 | 0.01 | 0.15 | 0.01 | 0.15 | 0.08 | 0.06 | 0.08 | 0.06 | 500 |
| 10 | 5 | 50 | 100 | 0 | 0 | 1.5 | 2 | 1 | 6.0 | 5.4 | 5.8 | 5.6 | 0.01 | 0.01 | 0.01 | 0.01 | 0.23 | 0.20 | 0.23 | 0.20 | 483 |
| 10 | 5 | 50 | 100 | 0 | 0 | 1.5 | 2 | 2 | 6.5 | 6.0 | 6.6 | 6.0 | 0.00 | 0.00 | 0.00 | 0.00 | 0.23 | 0.20 | 0.22 | 0.20 | 496 |
| 10 | 5 | 50 | 100 | 0 | 0 | 2.5 | 0.5 | 1 | 5.8 | 5.0 | 6.0 | 5.2 | 0.00 | 0.00 | 0.00 | 0.00 | 0.21 | 0.19 | 0.21 | 0.19 | 483 |
| 10 | 5 | 50 | 100 | 0 | 0 | 2.5 | 0.5 | 2 | 5.6 | 5.4 | 5.6 | 5.6 | 0.01 | 0.01 | 0.01 | 0.01 | 0.22 | 0.19 | 0.22 | 0.19 | 500 |
| 10 | 5 | 50 | 100 | 0 | 0.2 | 1.5 | 2 | 1 | 13.4 | 12.0 | 12.6 | 11.4 | 0.01 | 0.04 | 0.01 | 0.04 | 0.20 | 0.18 | 0.20 | 0.18 | 472 |
| 10 | 5 | 50 | 100 | 0 | 0.2 | 1.5 | 2 | 2 | 18.8 | 18.8 | 18.8 | 18.8 | 0.01 | 0.02 | 0.01 | 0.01 | 0.23 | 0.20 | 0.23 | 0.20 | 499 |
| 10 | 5 | 50 | 100 | 0 | 0.2 | 2.5 | 0.5 | 1 | 15.3 | 14.8 | 15.2 | 15.0 | 0.00 | 0.03 | 0.00 | 0.03 | 0.22 | 0.19 | 0.22 | 0.19 | 489 |
| 10 | 5 | 50 | 100 | 0 | 0.2 | 2.5 | 0.5 | 2 | 15.6 | 15.0 | 16.0 | 15.4 | 0.01 | 0.03 | 0.01 | 0.03 | 0.23 | 0.20 | 0.22 | 0.20 | 500 |
| 10 | 5 | 50 | 100 | 0 | 0.5 | 1.5 | 2 | 1 | 67.5 | 67.5 | 67.5 | 67.5 | 0.02 | 0.04 | 0.02 | 0.03 | 0.23 | 0.21 | 0.23 | 0.21 | 471 |
| 10 | 5 | 50 | 100 | 0 | 0.5 | 1.5 | 2 | 2 | 67.2 | 65.8 | 66.8 | 65.6 | 0.00 | 0.05 | 0.01 | 0.05 | 0.21 | 0.19 | 0.21 | 0.19 | 500 |
| 10 | 5 | 50 | 100 | 0 | 0.5 | 2.5 | 0.5 | 1 | 67.3 | 67.3 | 67.3 | 67.3 | 0.03 | 0.04 | 0.03 | 0.04 | 0.22 | 0.19 | 0.22 | 0.19 | 486 |
| 10 | 5 | 50 | 100 | 0 | 0.5 | 2.5 | 0.5 | 2 | 64.8 | 63.8 | 65.6 | 63.6 | 0.00 | 0.06 | 0.00 | 0.06 | 0.23 | 0.20 | 0.23 | 0.20 | 500 |
| 10 | 5 | 50 | 100 | 0.5 | 0 | 1.5 | 2 | 1 | 4.0 | 3.4 | 3.8 | 3.6 | 0.01 | 0.01 | 0.01 | 0.01 | 0.21 | 0.18 | 0.21 | 0.19 | 481 |
| 10 | 5 | 50 | 100 | 0.5 | 0 | 1.5 | 2 | 2 | 5.8 | 5.8 | 5.6 | 5.6 | 0.01 | 0.01 | 0.01 | 0.01 | 0.22 | 0.20 | 0.22 | 0.20 | 500 |
| 10 | 5 | 50 | 100 | 0.5 | 0 | 2.5 | 0.5 | 1 | 5.3 | 5.0 | 5.4 | 5.2 | 0.01 | 0.01 | 0.01 | 0.01 | 0.22 | 0.19 | 0.23 | 0.20 | 493 |
| 10 | 5 | 50 | 100 | 0.5 | 0 | 2.5 | 0.5 | 2 | 5.6 | 5.2 | 5.6 | 5.2 | 0.02 | 0.01 | 0.02 | 0.01 | 0.22 | 0.19 | 0.22 | 0.19 | 500 |
| 10 | 5 | 50 | 100 | 0.5 | 0.2 | 1.5 | 2 | 1 | 14.5 | 13.2 | 14.2 | 13.2 | 0.00 | 0.02 | 0.00 | 0.02 | 0.22 | 0.19 | 0.22 | 0.19 | 483 |
| 10 | 5 | 50 | 100 | 0.5 | 0.2 | 1.5 | 2 | 2 | 18.2 | 17.0 | 18.6 | 16.8 | 0.01 | 0.01 | 0.01 | 0.02 | 0.23 | 0.20 | 0.23 | 0.20 | 499 |
| 10 | 5 | 50 | 100 | 0.5 | 0.2 | 2.5 | 0.5 | 1 | 18.9 | 16.8 | 18.0 | 16.6 | 0.03 | 0.00 | 0.03 | 0.00 | 0.21 | 0.19 | 0.22 | 0.19 | 488 |
| 10 | 5 | 50 | 100 | 0.5 | 0.2 | 2.5 | 0.5 | 2 | 14.6 | 14.0 | 14.8 | 13.8 | 0.00 | 0.03 | 0.00 | 0.03 | 0.21 | 0.18 | 0.21 | 0.18 | 500 |
| 10 | 5 | 50 | 100 | 0.5 | 0.5 | 1.5 | 2 | 1 | 66.0 | 66.0 | 66.0 | 66.0 | 0.00 | 0.05 | 0.01 | 0.05 | 0.21 | 0.19 | 0.21 | 0.19 | 477 |
| 10 | 5 | 50 | 100 | 0.5 | 0.5 | 1.5 | 2 | 2 | 61.6 | 60.6 | 61.8 | 60.6 | 0.00 | 0.06 | 0.00 | 0.06 | 0.22 | 0.20 | 0.22 | 0.20 | 500 |
| 10 | 5 | 50 | 100 | 0.5 | 0.5 | 2.5 | 0.5 | 1 | 64.4 | 61.4 | 63.2 | 61.2 | 0.00 | 0.06 | 0.01 | 0.06 | 0.22 | 0.19 | 0.22 | 0.20 | 491 |
| 10 | 5 | 50 | 100 | 0.5 | 0.5 | 2.5 | 0.5 | 2 | 68.2 | 67.4 | 69.2 | 67.4 | 0.02 | 0.04 | 0.03 | 0.04 | 0.21 | 0.19 | 0.22 | 0.19 | 500 |
| 10 | 5 | 50 | 100 | 2 | 0 | 1.5 | 2 | 1 | 4.0 | 3.4 | 3.6 | 3.4 | 0.00 | 0.00 | 0.00 | 0.00 | 0.22 | 0.17 | 0.23 | 0.18 | 475 |
| 10 | 5 | 50 | 100 | 2 | 0 | 1.5 | 2 | 2 | 5.2 | 5.2 | 5.2 | 5.2 | 0.01 | 0.01 | 0.01 | 0.01 | 0.23 | 0.18 | 0.24 | 0.19 | 500 |
| 10 | 5 | 50 | 100 | 2 | 0 | 2.5 | 0.5 | 1 | 6.1 | 5.0 | 6.2 | 5.4 | 0.01 | 0.01 | 0.01 | 0.01 | 0.23 | 0.18 | 0.24 | 0.19 | 495 |
| 10 | 5 | 50 | 100 | 2 | 0 | 2.5 | 0.5 | 2 | 5.4 | 4.6 | 5.4 | 4.8 | 0.01 | 0.01 | 0.01 | 0.01 | 0.22 | 0.18 | 0.23 | 0.19 | 500 |
| 10 | 5 | 50 | 100 | 2 | 0.2 | 1.5 | 2 | 1 | 14.2 | 12.8 | 14.0 | 12.8 | 0.00 | 0.05 | 0.00 | 0.04 | 0.22 | 0.17 | 0.23 | 0.18 | 473 |
| 10 | 5 | 50 | 100 | 2 | 0.2 | 1.5 | 2 | 2 | 15.0 | 14.2 | 15.2 | 14.2 | 0.01 | 0.05 | 0.00 | 0.04 | 0.23 | 0.18 | 0.24 | 0.19 | 500 |
| 10 | 5 | 50 | 100 | 2 | 0.2 | 2.5 | 0.5 | 1 | 13.7 | 12.8 | 13.4 | 12.8 | 0.00 | 0.04 | 0.01 | 0.03 | 0.22 | 0.18 | 0.23 | 0.19 | 490 |
| 10 | 5 | 50 | 100 | 2 | 0.2 | 2.5 | 0.5 | 2 | 15.4 | 15.0 | 15.6 | 15.0 | 0.01 | 0.03 | 0.02 | 0.02 | 0.22 | 0.18 | 0.23 | 0.19 | 500 |
| 10 | 5 | 50 | 100 | 2 | 0.5 | 1.5 | 2 | 1 | 62.3 | 62.3 | 62.3 | 62.3 | 0.01 | 0.09 | 0.03 | 0.08 | 0.23 | 0.19 | 0.24 | 0.20 | 480 |
| 10 | 5 | 50 | 100 | 2 | 0.5 | 1.5 | 2 | 2 | 56.8 | 55.8 | 57.0 | 55.4 | 0.01 | 0.11 | 0.00 | 0.10 | 0.23 | 0.19 | 0.23 | 0.19 | 500 |
| 10 | 5 | 50 | 100 | 2 | 0.5 | 2.5 | 0.5 | 1 | 60.0 | 58.6 | 59.2 | 58.2 | 0.00 | 0.09 | 0.01 | 0.08 | 0.22 | 0.19 | 0.23 | 0.20 | 492 |
| 10 | 5 | 50 | 100 | 2 | 0.5 | 2.5 | 0.5 | 2 | 62.4 | 60.4 | 63.0 | 60.6 | 0.00 | 0.09 | 0.01 | 0.08 | 0.24 | 0.20 | 0.25 | 0.21 | 500 |
| 10 | 5 | 50 | 250 | 0 | 0 | 1.5 | 2 | 1 | 3.6 | 3.4 | 3.6 | 3.4 | 0.00 | 0.00 | 0.00 | 0.00 | 0.20 | 0.18 | 0.20 | 0.18 | 500 |
| 10 | 5 | 50 | 250 | 0 | 0 | 1.5 | 2 | 2 | 7.4 | 6.8 | 7.4 | 6.8 | 0.00 | 0.00 | 0.00 | 0.00 | 0.24 | 0.21 | 0.23 | 0.21 | 500 |
| 10 | 5 | 50 | 250 | 0 | 0 | 2.5 | 0.5 | 1 | 6.0 | 5.4 | 5.8 | 5.4 | 0.00 | 0.00 | 0.00 | 0.00 | 0.22 | 0.19 | 0.22 | 0.19 | 500 |
| 10 | 5 | 50 | 250 | 0 | 0 | 2.5 | 0.5 | 2 | 4.6 | 4.6 | 4.6 | 4.6 | 0.00 | 0.00 | 0.00 | 0.00 | 0.22 | 0.19 | 0.21 | 0.19 | 500 |
| 10 | 5 | 50 | 250 | 0 | 0.2 | 1.5 | 2 | 1 | 16.0 | 14.4 | 16.2 | 14.2 | 0.00 | 0.02 | 0.00 | 0.03 | 0.22 | 0.19 | 0.21 | 0.19 | 500 |
| 10 | 5 | 50 | 250 | 0 | 0.2 | 1.5 | 2 | 2 | 18.6 | 18.2 | 19.0 | 17.8 | 0.02 | 0.01 | 0.01 | 0.01 | 0.22 | 0.19 | 0.22 | 0.19 | 500 |
| 10 | 5 | 50 | 250 | 0 | 0.2 | 2.5 | 0.5 | 1 | 18.6 | 17.4 | 19.0 | 17.8 | 0.01 | 0.02 | 0.01 | 0.02 | 0.22 | 0.19 | 0.22 | 0.19 | 500 |
| 10 | 5 | 50 | 250 | 0 | 0.2 | 2.5 | 0.5 | 2 | 16.8 | 16.0 | 16.8 | 16.2 | 0.01 | 0.01 | 0.01 | 0.02 | 0.23 | 0.20 | 0.22 | 0.19 | 500 |
| 10 | 5 | 50 | 250 | 0 | 0.5 | 1.5 | 2 | 1 | 64.2 | 63.2 | 64.4 | 63.2 | 0.00 | 0.05 | 0.00 | 0.06 | 0.22 | 0.20 | 0.22 | 0.20 | 500 |
| 10 | 5 | 50 | 250 | 0 | 0.5 | 1.5 | 2 | 2 | 64.4 | 62.8 | 64.2 | 63.2 | 0.01 | 0.05 | 0.00 | 0.05 | 0.22 | 0.20 | 0.22 | 0.20 | 500 |
| 10 | 5 | 50 | 250 | 0 | 0.5 | 2.5 | 0.5 | 1 | 65.0 | 64.0 | 65.6 | 63.8 | 0.01 | 0.06 | 0.00 | 0.06 | 0.22 | 0.19 | 0.21 | 0.19 | 500 |
| 10 | 5 | 50 | 250 | 0 | 0.5 | 2.5 | 0.5 | 2 | 66.6 | 65.4 | 67.4 | 65.4 | 0.01 | 0.06 | 0.00 | 0.06 | 0.21 | 0.18 | 0.20 | 0.18 | 500 |
| 10 | 5 | 50 | 250 | 0.5 | 0 | 1.5 | 2 | 1 | 5.4 | 5.0 | 5.2 | 5.0 | 0.00 | 0.00 | 0.00 | 0.00 | 0.22 | 0.19 | 0.22 | 0.19 | 500 |
| 10 | 5 | 50 | 250 | 0.5 | 0 | 1.5 | 2 | 2 | 5.6 | 5.6 | 6.0 | 5.2 | 0.01 | 0.01 | 0.02 | 0.01 | 0.22 | 0.19 | 0.22 | 0.19 | 500 |
| 10 | 5 | 50 | 250 | 0.5 | 0 | 2.5 | 0.5 | 1 | 4.4 | 3.8 | 4.4 | 3.6 | 0.01 | 0.01 | 0.01 | 0.01 | 0.21 | 0.18 | 0.21 | 0.18 | 500 |
| 10 | 5 | 50 | 250 | 0.5 | 0 | 2.5 | 0.5 | 2 | 6.0 | 5.4 | 5.8 | 4.8 | 0.01 | 0.00 | 0.01 | 0.01 | 0.21 | 0.18 | 0.21 | 0.18 | 500 |
| 10 | 5 | 50 | 250 | 0.5 | 0.2 | 1.5 | 2 | 1 | 16.6 | 16.2 | 17.0 | 16.0 | 0.02 | 0.00 | 0.02 | 0.00 | 0.21 | 0.19 | 0.21 | 0.18 | 500 |
| 10 | 5 | 50 | 250 | 0.5 | 0.2 | 1.5 | 2 | 2 | 13.6 | 12.4 | 14.4 | 12.6 | 0.00 | 0.03 | 0.01 | 0.03 | 0.22 | 0.19 | 0.22 | 0.19 | 500 |
| 10 | 5 | 50 | 250 | 0.5 | 0.2 | 2.5 | 0.5 | 1 | 13.0 | 12.6 | 13.2 | 12.2 | 0.02 | 0.04 | 0.02 | 0.05 | 0.22 | 0.19 | 0.22 | 0.19 | 500 |
| 10 | 5 | 50 | 250 | 0.5 | 0.2 | 2.5 | 0.5 | 2 | 17.4 | 16.6 | 17.8 | 16.2 | 0.01 | 0.01 | 0.01 | 0.02 | 0.22 | 0.19 | 0.22 | 0.19 | 500 |
| 10 | 5 | 50 | 250 | 0.5 | 0.5 | 1.5 | 2 | 1 | 64.8 | 63.6 | 65.2 | 63.8 | 0.00 | 0.06 | 0.00 | 0.06 | 0.21 | 0.19 | 0.21 | 0.19 | 500 |
| 10 | 5 | 50 | 250 | 0.5 | 0.5 | 1.5 | 2 | 2 | 63.4 | 62.2 | 63.8 | 62.2 | 0.00 | 0.06 | 0.01 | 0.06 | 0.22 | 0.19 | 0.21 | 0.19 | 500 |
| 10 | 5 | 50 | 250 | 0.5 | 0.5 | 2.5 | 0.5 | 1 | 65.2 | 65.0 | 65.4 | 64.8 | 0.01 | 0.05 | 0.01 | 0.05 | 0.21 | 0.19 | 0.22 | 0.19 | 500 |
| 10 | 5 | 50 | 250 | 0.5 | 0.5 | 2.5 | 0.5 | 2 | 64.6 | 63.0 | 64.6 | 63.0 | 0.01 | 0.06 | 0.01 | 0.06 | 0.21 | 0.19 | 0.21 | 0.19 | 500 |
| 10 | 5 | 50 | 250 | 2 | 0 | 1.5 | 2 | 1 | 4.4 | 4.0 | 4.4 | 3.8 | 0.01 | 0.01 | 0.01 | 0.01 | 0.22 | 0.17 | 0.22 | 0.18 | 500 |
| 10 | 5 | 50 | 250 | 2 | 0 | 1.5 | 2 | 2 | 5.8 | 5.6 | 6.0 | 5.2 | 0.01 | 0.01 | 0.01 | 0.01 | 0.22 | 0.18 | 0.22 | 0.18 | 500 |
| 10 | 5 | 50 | 250 | 2 | 0 | 2.5 | 0.5 | 1 | 6.0 | 5.8 | 6.0 | 5.8 | 0.01 | 0.01 | 0.01 | 0.01 | 0.22 | 0.18 | 0.23 | 0.19 | 500 |
| 10 | 5 | 50 | 250 | 2 | 0 | 2.5 | 0.5 | 2 | 5.0 | 4.2 | 5.0 | 4.8 | 0.01 | 0.01 | 0.01 | 0.01 | 0.21 | 0.17 | 0.22 | 0.18 | 500 |
| 10 | 5 | 50 | 250 | 2 | 0.2 | 1.5 | 2 | 1 | 12.8 | 12.4 | 12.8 | 12.6 | 0.01 | 0.05 | 0.00 | 0.04 | 0.23 | 0.19 | 0.24 | 0.19 | 500 |
| 10 | 5 | 50 | 250 | 2 | 0.2 | 1.5 | 2 | 2 | 15.0 | 14.4 | 15.0 | 14.0 | 0.01 | 0.03 | 0.01 | 0.03 | 0.22 | 0.18 | 0.22 | 0.18 | 500 |
| 10 | 5 | 50 | 250 | 2 | 0.2 | 2.5 | 0.5 | 1 | 14.2 | 14.0 | 14.4 | 14.0 | 0.01 | 0.05 | 0.01 | 0.05 | 0.23 | 0.19 | 0.23 | 0.19 | 500 |
| 10 | 5 | 50 | 250 | 2 | 0.2 | 2.5 | 0.5 | 2 | 14.0 | 13.0 | 14.0 | 12.8 | 0.01 | 0.04 | 0.01 | 0.04 | 0.24 | 0.20 | 0.24 | 0.20 | 500 |
| 10 | 5 | 50 | 250 | 2 | 0.5 | 1.5 | 2 | 1 | 59.8 | 58.2 | 60.0 | 58.0 | 0.02 | 0.09 | 0.02 | 0.08 | 0.25 | 0.20 | 0.25 | 0.20 | 500 |
| 10 | 5 | 50 | 250 | 2 | 0.5 | 1.5 | 2 | 2 | 60.0 | 58.6 | 59.8 | 59.4 | 0.00 | 0.10 | 0.01 | 0.09 | 0.23 | 0.19 | 0.23 | 0.19 | 500 |
| 10 | 5 | 50 | 250 | 2 | 0.5 | 2.5 | 0.5 | 1 | 63.8 | 62.6 | 64.0 | 63.0 | 0.02 | 0.07 | 0.03 | 0.07 | 0.22 | 0.19 | 0.23 | 0.19 | 500 |
| 10 | 5 | 50 | 250 | 2 | 0.5 | 2.5 | 0.5 | 2 | 64.4 | 62.2 | 64.6 | 62.4 | 0.02 | 0.07 | 0.02 | 0.07 | 0.23 | 0.19 | 0.23 | 0.20 | 500 |
| 10 | 5 | 50 | 500 | 0 | 0 | 1.5 | 2 | 1 | 3.2 | 3.0 | 3.2 | 3.2 | 0.00 | 0.00 | 0.00 | 0.00 | 0.21 | 0.18 | 0.20 | 0.18 | 500 |
| 10 | 5 | 50 | 500 | 0 | 0 | 1.5 | 2 | 2 | 5.4 | 5.0 | 5.6 | 5.0 | 0.01 | 0.01 | 0.01 | 0.01 | 0.22 | 0.20 | 0.22 | 0.19 | 500 |
| 10 | 5 | 50 | 500 | 0 | 0 | 2.5 | 0.5 | 1 | 7.0 | 5.8 | 7.6 | 5.8 | 0.01 | 0.01 | 0.01 | 0.01 | 0.23 | 0.20 | 0.23 | 0.20 | 500 |
| 10 | 5 | 50 | 500 | 0 | 0 | 2.5 | 0.5 | 2 | 4.8 | 4.2 | 5.0 | 4.0 | 0.01 | 0.00 | 0.01 | 0.00 | 0.21 | 0.18 | 0.21 | 0.18 | 500 |
| 10 | 5 | 50 | 500 | 0 | 0.2 | 1.5 | 2 | 1 | 16.2 | 15.0 | 17.4 | 15.6 | 0.01 | 0.03 | 0.01 | 0.03 | 0.22 | 0.20 | 0.22 | 0.20 | 500 |
| 10 | 5 | 50 | 500 | 0 | 0.2 | 1.5 | 2 | 2 | 16.2 | 14.8 | 17.4 | 15.4 | 0.01 | 0.03 | 0.01 | 0.03 | 0.22 | 0.20 | 0.22 | 0.20 | 500 |
| 10 | 5 | 50 | 500 | 0 | 0.2 | 2.5 | 0.5 | 1 | 15.8 | 14.8 | 16.2 | 14.4 | 0.01 | 0.02 | 0.01 | 0.02 | 0.21 | 0.19 | 0.21 | 0.18 | 500 |
| 10 | 5 | 50 | 500 | 0 | 0.2 | 2.5 | 0.5 | 2 | 17.0 | 16.4 | 17.2 | 16.4 | 0.01 | 0.02 | 0.00 | 0.02 | 0.22 | 0.19 | 0.21 | 0.19 | 500 |
| 10 | 5 | 50 | 500 | 0 | 0.5 | 1.5 | 2 | 1 | 63.4 | 62.8 | 64.4 | 62.8 | 0.01 | 0.04 | 0.01 | 0.05 | 0.22 | 0.20 | 0.22 | 0.20 | 500 |
| 10 | 5 | 50 | 500 | 0 | 0.5 | 1.5 | 2 | 2 | 63.4 | 62.8 | 64.4 | 62.8 | 0.01 | 0.04 | 0.01 | 0.05 | 0.22 | 0.20 | 0.22 | 0.20 | 500 |
| 10 | 5 | 50 | 500 | 0 | 0.5 | 2.5 | 0.5 | 1 | 64.6 | 62.8 | 64.6 | 63.4 | 0.00 | 0.06 | 0.00 | 0.07 | 0.22 | 0.20 | 0.22 | 0.20 | 500 |
| 10 | 5 | 50 | 500 | 0 | 0.5 | 2.5 | 0.5 | 2 | 65.4 | 64.0 | 66.2 | 64.2 | 0.01 | 0.06 | 0.00 | 0.06 | 0.22 | 0.19 | 0.21 | 0.19 | 500 |
| 10 | 5 | 50 | 500 | 0.5 | 0 | 1.5 | 2 | 1 | 7.0 | 6.8 | 7.2 | 6.8 | 0.00 | 0.00 | 0.00 | 0.00 | 0.23 | 0.20 | 0.23 | 0.20 | 500 |
| 10 | 5 | 50 | 500 | 0.5 | 0 | 1.5 | 2 | 2 | 5.6 | 4.8 | 5.6 | 4.6 | 0.02 | 0.01 | 0.01 | 0.01 | 0.22 | 0.20 | 0.22 | 0.19 | 500 |
| 10 | 5 | 50 | 500 | 0.5 | 0 | 2.5 | 0.5 | 1 | 4.8 | 4.6 | 4.8 | 4.8 | 0.00 | 0.00 | 0.01 | 0.00 | 0.22 | 0.19 | 0.21 | 0.18 | 500 |
| 10 | 5 | 50 | 500 | 0.5 | 0 | 2.5 | 0.5 | 2 | 5.0 | 4.6 | 5.2 | 4.8 | 0.00 | 0.00 | 0.00 | 0.00 | 0.22 | 0.19 | 0.22 | 0.19 | 500 |
| 10 | 5 | 50 | 500 | 0.5 | 0.2 | 1.5 | 2 | 1 | 18.2 | 18.0 | 18.4 | 18.0 | 0.01 | 0.01 | 0.01 | 0.02 | 0.22 | 0.19 | 0.22 | 0.19 | 500 |
| 10 | 5 | 50 | 500 | 0.5 | 0.2 | 1.5 | 2 | 2 | 18.0 | 17.8 | 18.2 | 18.0 | 0.01 | 0.01 | 0.01 | 0.02 | 0.22 | 0.19 | 0.22 | 0.19 | 500 |
| 10 | 5 | 50 | 500 | 0.5 | 0.2 | 2.5 | 0.5 | 1 | 16.4 | 15.8 | 16.2 | 15.8 | 0.00 | 0.02 | 0.00 | 0.03 | 0.22 | 0.19 | 0.22 | 0.19 | 500 |
| 10 | 5 | 50 | 500 | 0.5 | 0.2 | 2.5 | 0.5 | 2 | 13.4 | 12.8 | 13.8 | 13.0 | 0.01 | 0.04 | 0.01 | 0.04 | 0.21 | 0.19 | 0.21 | 0.18 | 500 |
| 10 | 5 | 50 | 500 | 0.5 | 0.5 | 1.5 | 2 | 1 | 63.8 | 62.6 | 63.8 | 62.6 | 0.00 | 0.05 | 0.00 | 0.06 | 0.23 | 0.21 | 0.23 | 0.20 | 500 |
| 10 | 5 | 50 | 500 | 0.5 | 0.5 | 1.5 | 2 | 2 | 64.0 | 62.8 | 64.0 | 62.6 | 0.00 | 0.05 | 0.00 | 0.06 | 0.23 | 0.21 | 0.23 | 0.20 | 500 |
| 10 | 5 | 50 | 500 | 0.5 | 0.5 | 2.5 | 0.5 | 1 | 66.4 | 65.6 | 66.8 | 65.6 | 0.02 | 0.05 | 0.01 | 0.05 | 0.21 | 0.19 | 0.21 | 0.19 | 500 |
| 10 | 5 | 50 | 500 | 0.5 | 0.5 | 2.5 | 0.5 | 2 | 64.0 | 62.8 | 64.8 | 62.8 | 0.01 | 0.06 | 0.00 | 0.07 | 0.23 | 0.20 | 0.23 | 0.20 | 500 |
| 10 | 5 | 50 | 500 | 2 | 0 | 1.5 | 2 | 1 | 5.6 | 5.2 | 5.6 | 5.4 | 0.02 | 0.01 | 0.02 | 0.01 | 0.23 | 0.18 | 0.23 | 0.18 | 500 |
| 10 | 5 | 50 | 500 | 2 | 0 | 1.5 | 2 | 2 | 6.0 | 5.8 | 6.0 | 5.8 | 0.00 | 0.00 | 0.00 | 0.00 | 0.23 | 0.18 | 0.23 | 0.18 | 500 |
| 10 | 5 | 50 | 500 | 2 | 0 | 2.5 | 0.5 | 1 | 5.2 | 4.2 | 5.2 | 4.4 | 0.00 | 0.00 | 0.00 | 0.00 | 0.23 | 0.18 | 0.23 | 0.19 | 500 |
| 10 | 5 | 50 | 500 | 2 | 0 | 2.5 | 0.5 | 2 | 6.0 | 5.8 | 6.2 | 5.6 | 0.01 | 0.01 | 0.01 | 0.01 | 0.23 | 0.19 | 0.23 | 0.19 | 500 |
| 10 | 5 | 50 | 500 | 2 | 0.2 | 1.5 | 2 | 1 | 15.4 | 14.2 | 15.2 | 14.2 | 0.02 | 0.03 | 0.02 | 0.02 | 0.23 | 0.18 | 0.23 | 0.18 | 500 |
| 10 | 5 | 50 | 500 | 2 | 0.2 | 1.5 | 2 | 2 | 15.4 | 14.2 | 15.2 | 14.0 | 0.02 | 0.03 | 0.02 | 0.02 | 0.23 | 0.18 | 0.23 | 0.18 | 500 |
| 10 | 5 | 50 | 500 | 2 | 0.2 | 2.5 | 0.5 | 1 | 15.8 | 15.6 | 15.8 | 15.8 | 0.00 | 0.04 | 0.00 | 0.04 | 0.23 | 0.19 | 0.24 | 0.19 | 500 |
| 10 | 5 | 50 | 500 | 2 | 0.2 | 2.5 | 0.5 | 2 | 16.8 | 16.4 | 16.8 | 16.4 | 0.01 | 0.03 | 0.01 | 0.03 | 0.23 | 0.19 | 0.23 | 0.19 | 500 |
| 10 | 5 | 50 | 500 | 2 | 0.5 | 1.5 | 2 | 1 | 58.2 | 57.6 | 58.2 | 57.6 | 0.00 | 0.10 | 0.00 | 0.10 | 0.23 | 0.19 | 0.23 | 0.19 | 500 |
| 10 | 5 | 50 | 500 | 2 | 0.5 | 1.5 | 2 | 2 | 58.2 | 57.8 | 58.4 | 57.8 | 0.00 | 0.10 | 0.00 | 0.10 | 0.23 | 0.19 | 0.23 | 0.19 | 500 |
| 10 | 5 | 50 | 500 | 2 | 0.5 | 2.5 | 0.5 | 1 | 58.6 | 57.8 | 58.6 | 57.8 | 0.00 | 0.10 | 0.00 | 0.09 | 0.24 | 0.20 | 0.24 | 0.20 | 500 |
| 10 | 5 | 50 | 500 | 2 | 0.5 | 2.5 | 0.5 | 2 | 61.8 | 60.8 | 62.0 | 60.4 | 0.01 | 0.08 | 0.01 | 0.08 | 0.22 | 0.19 | 0.22 | 0.19 | 500 |
| 10 | 5 | 100 | 100 | 0 | 0 | 1.5 | 2 | 1 | 4.5 | 4.2 | 4.2 | 4.2 | 0.01 | 0.00 | 0.01 | 0.00 | 0.15 | 0.13 | 0.15 | 0.13 | 469 |
| 10 | 5 | 100 | 100 | 0 | 0 | 1.5 | 2 | 2 | 4.2 | 4.0 | 4.2 | 4.2 | 0.00 | 0.00 | 0.00 | 0.00 | 0.15 | 0.13 | 0.15 | 0.13 | 498 |
| 10 | 5 | 100 | 100 | 0 | 0 | 2.5 | 0.5 | 1 | 5.9 | 5.8 | 6.4 | 5.8 | 0.00 | 0.00 | 0.00 | 0.00 | 0.16 | 0.14 | 0.16 | 0.14 | 494 |
| 10 | 5 | 100 | 100 | 0 | 0 | 2.5 | 0.5 | 2 | 7.0 | 7.0 | 7.2 | 6.8 | 0.02 | 0.01 | 0.02 | 0.01 | 0.16 | 0.14 | 0.16 | 0.14 | 498 |
| 10 | 5 | 100 | 100 | 0 | 0.2 | 1.5 | 2 | 1 | 29.1 | 29.1 | 29.1 | 29.1 | 0.00 | 0.02 | 0.01 | 0.02 | 0.16 | 0.14 | 0.16 | 0.14 | 471 |
| 10 | 5 | 100 | 100 | 0 | 0.2 | 1.5 | 2 | 2 | 26.1 | 25.8 | 26.4 | 26.2 | 0.00 | 0.02 | 0.00 | 0.02 | 0.16 | 0.14 | 0.16 | 0.14 | 499 |
| 10 | 5 | 100 | 100 | 0 | 0.2 | 2.5 | 0.5 | 1 | 23.4 | 22.8 | 23.0 | 23.0 | 0.01 | 0.04 | 0.01 | 0.03 | 0.15 | 0.13 | 0.15 | 0.13 | 492 |
| 10 | 5 | 100 | 100 | 0 | 0.2 | 2.5 | 0.5 | 2 | 24.9 | 24.4 | 25.0 | 24.2 | 0.00 | 0.03 | 0.00 | 0.03 | 0.15 | 0.13 | 0.15 | 0.14 | 499 |
| 10 | 5 | 100 | 100 | 0 | 0.5 | 1.5 | 2 | 1 | 93.8 | 93.8 | 93.8 | 93.8 | 0.02 | 0.03 | 0.02 | 0.03 | 0.16 | 0.14 | 0.16 | 0.14 | 485 |
| 10 | 5 | 100 | 100 | 0 | 0.5 | 1.5 | 2 | 2 | 91.8 | 91.2 | 91.6 | 91.2 | 0.00 | 0.05 | 0.00 | 0.05 | 0.16 | 0.14 | 0.16 | 0.15 | 498 |
| 10 | 5 | 100 | 100 | 0 | 0.5 | 2.5 | 0.5 | 1 | 88.8 | 88.8 | 88.8 | 88.8 | 0.01 | 0.05 | 0.02 | 0.05 | 0.16 | 0.14 | 0.16 | 0.15 | 493 |
| 10 | 5 | 100 | 100 | 0 | 0.5 | 2.5 | 0.5 | 2 | 90.2 | 89.4 | 89.8 | 89.4 | 0.00 | 0.06 | 0.00 | 0.06 | 0.15 | 0.14 | 0.16 | 0.14 | 499 |
| 10 | 5 | 100 | 100 | 0.5 | 0 | 1.5 | 2 | 1 | 4.9 | 4.6 | 4.6 | 4.6 | 0.02 | 0.02 | 0.02 | 0.02 | 0.15 | 0.13 | 0.15 | 0.13 | 471 |
| 10 | 5 | 100 | 100 | 0.5 | 0 | 1.5 | 2 | 2 | 5.4 | 5.4 | 5.4 | 5.4 | 0.00 | 0.00 | 0.00 | 0.00 | 0.15 | 0.13 | 0.16 | 0.14 | 500 |
| 10 | 5 | 100 | 100 | 0.5 | 0 | 2.5 | 0.5 | 1 | 5.1 | 5.0 | 5.0 | 5.0 | 0.00 | 0.00 | 0.00 | 0.00 | 0.16 | 0.13 | 0.16 | 0.14 | 490 |
| 10 | 5 | 100 | 100 | 0.5 | 0 | 2.5 | 0.5 | 2 | 5.4 | 4.8 | 5.4 | 5.2 | 0.00 | 0.00 | 0.00 | 0.00 | 0.15 | 0.13 | 0.15 | 0.13 | 500 |
| 10 | 5 | 100 | 100 | 0.5 | 0.2 | 1.5 | 2 | 1 | 24.8 | 23.2 | 24.4 | 23.4 | 0.01 | 0.03 | 0.00 | 0.03 | 0.15 | 0.13 | 0.15 | 0.13 | 483 |
| 10 | 5 | 100 | 100 | 0.5 | 0.2 | 1.5 | 2 | 2 | 26.5 | 25.4 | 26.2 | 25.0 | 0.00 | 0.03 | 0.00 | 0.02 | 0.15 | 0.13 | 0.15 | 0.13 | 498 |
| 10 | 5 | 100 | 100 | 0.5 | 0.2 | 2.5 | 0.5 | 1 | 24.9 | 24.2 | 24.8 | 23.8 | 0.00 | 0.03 | 0.00 | 0.03 | 0.15 | 0.13 | 0.16 | 0.13 | 495 |
| 10 | 5 | 100 | 100 | 0.5 | 0.2 | 2.5 | 0.5 | 2 | 25.8 | 25.0 | 25.6 | 24.6 | 0.00 | 0.03 | 0.00 | 0.02 | 0.15 | 0.13 | 0.15 | 0.13 | 500 |
| 10 | 5 | 100 | 100 | 0.5 | 0.5 | 1.5 | 2 | 1 | 88.7 | 88.7 | 88.7 | 88.7 | 0.00 | 0.06 | 0.00 | 0.05 | 0.16 | 0.15 | 0.16 | 0.15 | 478 |
| 10 | 5 | 100 | 100 | 0.5 | 0.5 | 1.5 | 2 | 2 | 93.0 | 92.4 | 93.0 | 92.6 | 0.01 | 0.05 | 0.02 | 0.04 | 0.14 | 0.13 | 0.15 | 0.13 | 500 |
| 10 | 5 | 100 | 100 | 0.5 | 0.5 | 2.5 | 0.5 | 1 | 89.2 | 89.4 | 89.2 | 89.4 | 0.00 | 0.06 | 0.00 | 0.06 | 0.15 | 0.14 | 0.16 | 0.14 | 481 |
| 10 | 5 | 100 | 100 | 0.5 | 0.5 | 2.5 | 0.5 | 2 | 91.6 | 91.2 | 91.6 | 91.2 | 0.00 | 0.06 | 0.01 | 0.06 | 0.15 | 0.14 | 0.16 | 0.14 | 500 |
| 10 | 5 | 100 | 100 | 2 | 0 | 1.5 | 2 | 1 | 7.4 | 6.6 | 7.0 | 6.6 | 0.00 | 0.00 | 0.00 | 0.00 | 0.17 | 0.13 | 0.18 | 0.14 | 475 |
| 10 | 5 | 100 | 100 | 2 | 0 | 1.5 | 2 | 2 | 3.4 | 3.4 | 3.4 | 3.2 | 0.00 | 0.00 | 0.00 | 0.00 | 0.15 | 0.12 | 0.15 | 0.12 | 500 |
| 10 | 5 | 100 | 100 | 2 | 0 | 2.5 | 0.5 | 1 | 5.3 | 5.0 | 5.2 | 5.0 | 0.00 | 0.00 | 0.00 | 0.00 | 0.16 | 0.13 | 0.16 | 0.13 | 494 |
| 10 | 5 | 100 | 100 | 2 | 0 | 2.5 | 0.5 | 2 | 5.0 | 4.8 | 5.0 | 4.8 | 0.01 | 0.01 | 0.01 | 0.01 | 0.16 | 0.13 | 0.16 | 0.13 | 500 |
| 10 | 5 | 100 | 100 | 2 | 0.2 | 1.5 | 2 | 1 | 29.1 | 27.0 | 28.6 | 27.0 | 0.01 | 0.03 | 0.02 | 0.03 | 0.17 | 0.13 | 0.17 | 0.14 | 485 |
| 10 | 5 | 100 | 100 | 2 | 0.2 | 1.5 | 2 | 2 | 24.7 | 24.4 | 25.4 | 24.4 | 0.00 | 0.04 | 0.01 | 0.04 | 0.16 | 0.13 | 0.17 | 0.13 | 499 |
| 10 | 5 | 100 | 100 | 2 | 0.2 | 2.5 | 0.5 | 1 | 24.3 | 23.8 | 24.2 | 23.6 | 0.01 | 0.04 | 0.00 | 0.03 | 0.16 | 0.13 | 0.17 | 0.14 | 494 |
| 10 | 5 | 100 | 100 | 2 | 0.2 | 2.5 | 0.5 | 2 | 23.0 | 22.2 | 23.2 | 22.0 | 0.00 | 0.04 | 0.01 | 0.03 | 0.16 | 0.13 | 0.17 | 0.14 | 500 |
| 10 | 5 | 100 | 100 | 2 | 0.5 | 1.5 | 2 | 1 | 87.7 | 82.6 | 82.8 | 82.6 | 0.00 | 0.10 | 0.02 | 0.09 | 0.15 | 0.13 | 0.16 | 0.13 | 471 |
| 10 | 5 | 100 | 100 | 2 | 0.5 | 1.5 | 2 | 2 | 88.0 | 87.6 | 87.8 | 87.4 | 0.01 | 0.09 | 0.03 | 0.08 | 0.16 | 0.14 | 0.17 | 0.14 | 499 |
| 10 | 5 | 100 | 100 | 2 | 0.5 | 2.5 | 0.5 | 1 | 86.8 | 86.8 | 86.8 | 86.8 | 0.01 | 0.10 | 0.01 | 0.08 | 0.16 | 0.14 | 0.17 | 0.15 | 492 |
| 10 | 5 | 100 | 100 | 2 | 0.5 | 2.5 | 0.5 | 2 | 87.8 | 87.8 | 87.8 | 87.8 | 0.00 | 0.09 | 0.02 | 0.07 | 0.17 | 0.14 | 0.18 | 0.15 | 500 |
| 10 | 5 | 100 | 250 | 0 | 0 | 1.5 | 2 | 1 | 6.2 | 5.2 | 6.2 | 5.4 | 0.01 | 0.01 | 0.01 | 0.01 | 0.15 | 0.14 | 0.15 | 0.14 | 500 |
| 10 | 5 | 100 | 250 | 0 | 0 | 1.5 | 2 | 2 | 7.6 | 7.2 | 7.4 | 7.2 | 0.01 | 0.01 | 0.01 | 0.01 | 0.16 | 0.15 | 0.16 | 0.14 | 500 |
| 10 | 5 | 100 | 250 | 0 | 0 | 2.5 | 0.5 | 1 | 7.2 | 7.2 | 7.2 | 7.2 | 0.00 | 0.00 | 0.00 | 0.00 | 0.16 | 0.14 | 0.16 | 0.14 | 500 |
| 10 | 5 | 100 | 250 | 0 | 0 | 2.5 | 0.5 | 2 | 4.0 | 3.8 | 4.0 | 3.8 | 0.00 | 0.00 | 0.00 | 0.00 | 0.15 | 0.13 | 0.15 | 0.13 | 500 |
| 10 | 5 | 100 | 250 | 0 | 0.2 | 1.5 | 2 | 1 | 28.0 | 27.8 | 28.2 | 27.8 | 0.01 | 0.02 | 0.01 | 0.02 | 0.14 | 0.13 | 0.14 | 0.13 | 500 |
| 10 | 5 | 100 | 250 | 0 | 0.2 | 1.5 | 2 | 2 | 26.6 | 25.4 | 26.8 | 25.4 | 0.01 | 0.03 | 0.01 | 0.03 | 0.15 | 0.14 | 0.15 | 0.14 | 500 |
| 10 | 5 | 100 | 250 | 0 | 0.2 | 2.5 | 0.5 | 1 | 27.4 | 26.4 | 27.6 | 26.6 | 0.01 | 0.03 | 0.01 | 0.03 | 0.16 | 0.14 | 0.16 | 0.14 | 500 |
| 10 | 5 | 100 | 250 | 0 | 0.2 | 2.5 | 0.5 | 2 | 28.6 | 28.0 | 29.0 | 28.2 | 0.00 | 0.03 | 0.00 | 0.03 | 0.16 | 0.14 | 0.16 | 0.14 | 500 |
| 10 | 5 | 100 | 250 | 0 | 0.5 | 1.5 | 2 | 1 | 91.8 | 91.8 | 92.0 | 91.6 | 0.00 | 0.05 | 0.00 | 0.05 | 0.16 | 0.14 | 0.16 | 0.14 | 500 |
| 10 | 5 | 100 | 250 | 0 | 0.5 | 1.5 | 2 | 2 | 91.6 | 91.6 | 91.6 | 91.4 | 0.01 | 0.05 | 0.01 | 0.05 | 0.16 | 0.14 | 0.16 | 0.14 | 500 |
| 10 | 5 | 100 | 250 | 0 | 0.5 | 2.5 | 0.5 | 1 | 92.2 | 92.0 | 92.2 | 92.2 | 0.01 | 0.05 | 0.01 | 0.05 | 0.15 | 0.14 | 0.15 | 0.14 | 500 |
| 10 | 5 | 100 | 250 | 0 | 0.5 | 2.5 | 0.5 | 2 | 90.8 | 90.4 | 91.0 | 90.4 | 0.01 | 0.06 | 0.01 | 0.06 | 0.16 | 0.14 | 0.16 | 0.14 | 500 |
| 10 | 5 | 100 | 250 | 0.5 | 0 | 1.5 | 2 | 1 | 3.8 | 3.6 | 3.8 | 3.6 | 0.00 | 0.00 | 0.00 | 0.00 | 0.15 | 0.13 | 0.15 | 0.13 | 500 |
| 10 | 5 | 100 | 250 | 0.5 | 0 | 1.5 | 2 | 2 | 4.6 | 4.2 | 4.6 | 4.2 | 0.02 | 0.01 | 0.02 | 0.01 | 0.15 | 0.13 | 0.15 | 0.13 | 500 |
| 10 | 5 | 100 | 250 | 0.5 | 0 | 2.5 | 0.5 | 1 | 6.4 | 6.2 | 6.6 | 6.2 | 0.00 | 0.00 | 0.00 | 0.00 | 0.17 | 0.14 | 0.17 | 0.14 | 500 |
| 10 | 5 | 100 | 250 | 0.5 | 0 | 2.5 | 0.5 | 2 | 5.6 | 5.6 | 5.6 | 5.4 | 0.00 | 0.00 | 0.00 | 0.00 | 0.16 | 0.13 | 0.15 | 0.13 | 500 |
| 10 | 5 | 100 | 250 | 0.5 | 0.2 | 1.5 | 2 | 1 | 24.6 | 24.6 | 25.0 | 24.6 | 0.00 | 0.03 | 0.01 | 0.03 | 0.15 | 0.13 | 0.15 | 0.13 | 500 |
| 10 | 5 | 100 | 250 | 0.5 | 0.2 | 1.5 | 2 | 2 | 28.2 | 26.6 | 27.6 | 27.0 | 0.01 | 0.02 | 0.01 | 0.02 | 0.15 | 0.13 | 0.15 | 0.13 | 500 |
| 10 | 5 | 100 | 250 | 0.5 | 0.2 | 2.5 | 0.5 | 1 | 24.2 | 22.8 | 24.4 | 23.4 | 0.01 | 0.03 | 0.01 | 0.03 | 0.16 | 0.14 | 0.16 | 0.13 | 500 |
| 10 | 5 | 100 | 250 | 0.5 | 0.2 | 2.5 | 0.5 | 2 | 26.6 | 26.4 | 26.8 | 26.4 | 0.00 | 0.03 | 0.00 | 0.03 | 0.16 | 0.14 | 0.16 | 0.14 | 500 |
| 10 | 5 | 100 | 250 | 0.5 | 0.5 | 1.5 | 2 | 1 | 90.4 | 90.0 | 90.6 | 90.2 | 0.01 | 0.05 | 0.01 | 0.05 | 0.16 | 0.14 | 0.16 | 0.14 | 499 |
| 10 | 5 | 100 | 250 | 0.5 | 0.5 | 1.5 | 2 | 2 | 92.8 | 92.8 | 92.8 | 92.8 | 0.01 | 0.05 | 0.01 | 0.05 | 0.15 | 0.14 | 0.15 | 0.14 | 500 |
| 10 | 5 | 100 | 250 | 0.5 | 0.5 | 2.5 | 0.5 | 1 | 89.8 | 89.4 | 89.8 | 89.6 | 0.01 | 0.06 | 0.01 | 0.06 | 0.16 | 0.14 | 0.16 | 0.14 | 500 |
| 10 | 5 | 100 | 250 | 0.5 | 0.5 | 2.5 | 0.5 | 2 | 91.8 | 91.4 | 92.2 | 91.6 | 0.01 | 0.05 | 0.01 | 0.05 | 0.16 | 0.14 | 0.16 | 0.14 | 500 |
| 10 | 5 | 100 | 250 | 2 | 0 | 1.5 | 2 | 1 | 4.6 | 4.4 | 4.8 | 4.2 | 0.00 | 0.00 | 0.00 | 0.00 | 0.16 | 0.13 | 0.16 | 0.13 | 500 |
| 10 | 5 | 100 | 250 | 2 | 0 | 1.5 | 2 | 2 | 3.8 | 3.8 | 3.8 | 3.8 | 0.00 | 0.00 | 0.00 | 0.00 | 0.16 | 0.13 | 0.16 | 0.13 | 500 |
| 10 | 5 | 100 | 250 | 2 | 0 | 2.5 | 0.5 | 1 | 5.0 | 4.8 | 5.0 | 4.6 | 0.00 | 0.00 | 0.01 | 0.00 | 0.15 | 0.12 | 0.16 | 0.13 | 500 |
| 10 | 5 | 100 | 250 | 2 | 0 | 2.5 | 0.5 | 2 | 4.6 | 4.2 | 4.8 | 4.4 | 0.00 | 0.00 | 0.00 | 0.00 | 0.16 | 0.13 | 0.16 | 0.13 | 500 |
| 10 | 5 | 100 | 250 | 2 | 0.2 | 1.5 | 2 | 1 | 26.6 | 26.0 | 26.6 | 25.8 | 0.01 | 0.04 | 0.01 | 0.03 | 0.17 | 0.13 | 0.17 | 0.13 | 500 |
| 10 | 5 | 100 | 250 | 2 | 0.2 | 1.5 | 2 | 2 | 27.0 | 26.6 | 26.8 | 26.4 | 0.00 | 0.04 | 0.00 | 0.04 | 0.17 | 0.13 | 0.17 | 0.14 | 500 |
| 10 | 5 | 100 | 250 | 2 | 0.2 | 2.5 | 0.5 | 1 | 24.8 | 23.8 | 24.8 | 23.6 | 0.00 | 0.04 | 0.00 | 0.04 | 0.16 | 0.13 | 0.16 | 0.13 | 500 |
| 10 | 5 | 100 | 250 | 2 | 0.2 | 2.5 | 0.5 | 2 | 25.6 | 25.0 | 26.0 | 25.0 | 0.00 | 0.04 | 0.01 | 0.03 | 0.16 | 0.13 | 0.17 | 0.14 | 500 |
| 10 | 5 | 100 | 250 | 2 | 0.5 | 1.5 | 2 | 1 | 86.2 | 85.6 | 86.0 | 85.6 | 0.01 | 0.10 | 0.01 | 0.09 | 0.17 | 0.14 | 0.17 | 0.14 | 500 |
| 10 | 5 | 100 | 250 | 2 | 0.5 | 1.5 | 2 | 2 | 88.0 | 87.6 | 88.0 | 87.6 | 0.01 | 0.09 | 0.02 | 0.09 | 0.17 | 0.14 | 0.18 | 0.14 | 500 |
| 10 | 5 | 100 | 250 | 2 | 0.5 | 2.5 | 0.5 | 1 | 90.8 | 90.6 | 91.2 | 90.8 | 0.00 | 0.09 | 0.01 | 0.08 | 0.15 | 0.13 | 0.15 | 0.13 | 500 |
| 10 | 5 | 100 | 250 | 2 | 0.5 | 2.5 | 0.5 | 2 | 87.8 | 87.6 | 87.6 | 87.6 | 0.02 | 0.07 | 0.03 | 0.07 | 0.16 | 0.14 | 0.17 | 0.14 | 500 |
| 10 | 5 | 100 | 500 | 0 | 0 | 1.5 | 2 | 1 | 5.8 | 5.6 | 5.8 | 5.6 | 0.00 | 0.00 | 0.00 | 0.00 | 0.16 | 0.14 | 0.15 | 0.14 | 500 |
| 10 | 5 | 100 | 500 | 0 | 0 | 1.5 | 2 | 2 | 6.0 | 6.0 | 6.0 | 6.0 | 0.02 | 0.01 | 0.02 | 0.01 | 0.16 | 0.14 | 0.16 | 0.14 | 500 |
| 10 | 5 | 100 | 500 | 0 | 0 | 2.5 | 0.5 | 1 | 4.0 | 3.8 | 4.0 | 4.0 | 0.01 | 0.01 | 0.01 | 0.01 | 0.15 | 0.13 | 0.15 | 0.13 | 500 |
| 10 | 5 | 100 | 500 | 0 | 0 | 2.5 | 0.5 | 2 | 6.8 | 6.2 | 7.0 | 6.4 | 0.02 | 0.01 | 0.02 | 0.01 | 0.17 | 0.15 | 0.17 | 0.15 | 500 |
| 10 | 5 | 100 | 500 | 0 | 0.2 | 1.5 | 2 | 1 | 23.2 | 22.8 | 23.0 | 22.8 | 0.00 | 0.03 | 0.01 | 0.03 | 0.15 | 0.13 | 0.15 | 0.13 | 500 |
| 10 | 5 | 100 | 500 | 0 | 0.2 | 1.5 | 2 | 2 | 23.2 | 22.8 | 23.0 | 22.8 | 0.00 | 0.03 | 0.01 | 0.03 | 0.15 | 0.13 | 0.15 | 0.13 | 500 |
| 10 | 5 | 100 | 500 | 0 | 0.2 | 2.5 | 0.5 | 1 | 25.0 | 24.6 | 25.2 | 24.6 | 0.01 | 0.03 | 0.01 | 0.03 | 0.16 | 0.14 | 0.16 | 0.14 | 500 |
| 10 | 5 | 100 | 500 | 0 | 0.2 | 2.5 | 0.5 | 2 | 25.4 | 24.4 | 25.2 | 24.4 | 0.00 | 0.03 | 0.00 | 0.03 | 0.15 | 0.13 | 0.15 | 0.13 | 500 |
| 10 | 5 | 100 | 500 | 0 | 0.5 | 1.5 | 2 | 1 | 89.0 | 88.8 | 89.2 | 89.0 | 0.01 | 0.06 | 0.01 | 0.06 | 0.16 | 0.14 | 0.16 | 0.14 | 500 |
| 10 | 5 | 100 | 500 | 0 | 0.5 | 1.5 | 2 | 2 | 91.0 | 89.8 | 91.0 | 90.0 | 0.01 | 0.05 | 0.00 | 0.05 | 0.16 | 0.14 | 0.16 | 0.14 | 500 |
| 10 | 5 | 100 | 500 | 0 | 0.5 | 2.5 | 0.5 | 1 | 90.8 | 90.6 | 91.2 | 90.2 | 0.00 | 0.07 | 0.01 | 0.07 | 0.15 | 0.13 | 0.15 | 0.13 | 500 |
| 10 | 5 | 100 | 500 | 0 | 0.5 | 2.5 | 0.5 | 2 | 91.4 | 90.6 | 91.6 | 90.4 | 0.01 | 0.06 | 0.01 | 0.06 | 0.15 | 0.14 | 0.15 | 0.13 | 500 |
| 10 | 5 | 100 | 500 | 0.5 | 0 | 1.5 | 2 | 1 | 5.8 | 5.2 | 6.2 | 5.6 | 0.00 | 0.00 | 0.00 | 0.00 | 0.15 | 0.13 | 0.15 | 0.13 | 500 |
| 10 | 5 | 100 | 500 | 0.5 | 0 | 1.5 | 2 | 2 | 4.6 | 4.6 | 4.6 | 4.6 | 0.01 | 0.01 | 0.01 | 0.01 | 0.15 | 0.13 | 0.15 | 0.13 | 500 |
| 10 | 5 | 100 | 500 | 0.5 | 0 | 2.5 | 0.5 | 1 | 5.4 | 5.4 | 5.4 | 5.2 | 0.01 | 0.01 | 0.01 | 0.01 | 0.16 | 0.14 | 0.16 | 0.14 | 500 |
| 10 | 5 | 100 | 500 | 0.5 | 0 | 2.5 | 0.5 | 2 | 6.4 | 6.2 | 6.4 | 6.2 | 0.00 | 0.00 | 0.00 | 0.00 | 0.16 | 0.14 | 0.16 | 0.13 | 500 |
| 10 | 5 | 100 | 500 | 0.5 | 0.2 | 1.5 | 2 | 1 | 28.2 | 27.6 | 28.2 | 27.6 | 0.00 | 0.02 | 0.00 | 0.02 | 0.16 | 0.14 | 0.16 | 0.14 | 500 |
| 10 | 5 | 100 | 500 | 0.5 | 0.2 | 1.5 | 2 | 2 | 28.2 | 27.6 | 28.2 | 27.6 | 0.00 | 0.02 | 0.00 | 0.02 | 0.16 | 0.14 | 0.16 | 0.14 | 500 |
| 10 | 5 | 100 | 500 | 0.5 | 0.2 | 2.5 | 0.5 | 1 | 24.4 | 24.0 | 24.6 | 23.8 | 0.00 | 0.03 | 0.00 | 0.03 | 0.16 | 0.14 | 0.16 | 0.14 | 500 |
| 10 | 5 | 100 | 500 | 0.5 | 0.2 | 2.5 | 0.5 | 2 | 26.0 | 25.6 | 26.0 | 25.4 | 0.00 | 0.03 | 0.00 | 0.03 | 0.15 | 0.13 | 0.15 | 0.13 | 500 |
| 10 | 5 | 100 | 500 | 0.5 | 0.5 | 1.5 | 2 | 1 | 91.2 | 91.0 | 91.2 | 91.0 | 0.01 | 0.05 | 0.00 | 0.05 | 0.15 | 0.13 | 0.15 | 0.13 | 500 |
| 10 | 5 | 100 | 500 | 0.5 | 0.5 | 1.5 | 2 | 2 | 91.0 | 90.8 | 91.0 | 90.8 | 0.00 | 0.06 | 0.00 | 0.06 | 0.16 | 0.14 | 0.15 | 0.14 | 500 |
| 10 | 5 | 100 | 500 | 0.5 | 0.5 | 2.5 | 0.5 | 1 | 90.0 | 89.4 | 90.0 | 89.6 | 0.00 | 0.06 | 0.00 | 0.07 | 0.16 | 0.14 | 0.15 | 0.14 | 500 |
| 10 | 5 | 100 | 500 | 0.5 | 0.5 | 2.5 | 0.5 | 2 | 90.2 | 90.2 | 90.6 | 90.2 | 0.01 | 0.06 | 0.00 | 0.06 | 0.16 | 0.14 | 0.16 | 0.14 | 500 |
| 10 | 5 | 100 | 500 | 2 | 0 | 1.5 | 2 | 1 | 5.0 | 4.8 | 5.2 | 5.0 | 0.01 | 0.00 | 0.01 | 0.01 | 0.16 | 0.13 | 0.16 | 0.13 | 500 |
| 10 | 5 | 100 | 500 | 2 | 0 | 1.5 | 2 | 2 | 5.6 | 5.6 | 5.6 | 5.6 | 0.01 | 0.01 | 0.01 | 0.01 | 0.16 | 0.13 | 0.16 | 0.13 | 500 |
| 10 | 5 | 100 | 500 | 2 | 0 | 2.5 | 0.5 | 1 | 5.8 | 5.6 | 5.8 | 5.8 | 0.00 | 0.00 | 0.00 | 0.00 | 0.16 | 0.13 | 0.16 | 0.13 | 500 |
| 10 | 5 | 100 | 500 | 2 | 0 | 2.5 | 0.5 | 2 | 5.6 | 5.6 | 5.6 | 5.6 | 0.01 | 0.00 | 0.01 | 0.00 | 0.16 | 0.13 | 0.16 | 0.13 | 500 |
| 10 | 5 | 100 | 500 | 2 | 0.2 | 1.5 | 2 | 1 | 24.0 | 23.8 | 24.0 | 23.8 | 0.01 | 0.04 | 0.01 | 0.04 | 0.15 | 0.12 | 0.15 | 0.12 | 500 |
| 10 | 5 | 100 | 500 | 2 | 0.2 | 1.5 | 2 | 2 | 24.8 | 24.4 | 24.8 | 24.4 | 0.01 | 0.04 | 0.01 | 0.04 | 0.15 | 0.12 | 0.15 | 0.12 | 500 |
| 10 | 5 | 100 | 500 | 2 | 0.2 | 2.5 | 0.5 | 1 | 22.6 | 22.0 | 22.6 | 21.8 | 0.00 | 0.04 | 0.00 | 0.03 | 0.16 | 0.13 | 0.16 | 0.13 | 500 |
| 10 | 5 | 100 | 500 | 2 | 0.2 | 2.5 | 0.5 | 2 | 27.6 | 26.8 | 27.6 | 27.0 | 0.01 | 0.03 | 0.01 | 0.03 | 0.17 | 0.14 | 0.17 | 0.14 | 500 |
| 10 | 5 | 100 | 500 | 2 | 0.5 | 1.5 | 2 | 1 | 85.2 | 85.0 | 85.2 | 84.6 | 0.01 | 0.11 | 0.00 | 0.10 | 0.17 | 0.14 | 0.17 | 0.14 | 500 |
| 10 | 5 | 100 | 500 | 2 | 0.5 | 1.5 | 2 | 2 | 86.6 | 86.6 | 86.8 | 86.4 | 0.00 | 0.10 | 0.00 | 0.10 | 0.16 | 0.13 | 0.16 | 0.14 | 500 |
| 10 | 5 | 100 | 500 | 2 | 0.5 | 2.5 | 0.5 | 1 | 89.4 | 89.2 | 89.6 | 89.2 | 0.01 | 0.08 | 0.02 | 0.07 | 0.17 | 0.14 | 0.17 | 0.14 | 500 |
| 10 | 5 | 100 | 500 | 2 | 0.5 | 2.5 | 0.5 | 2 | 88.6 | 88.4 | 88.6 | 88.2 | 0.01 | 0.08 | 0.02 | 0.08 | 0.16 | 0.13 | 0.16 | 0.13 | 500 |
| 10 | 5 | 200 | 100 | 0 | 0 | 1.5 | 2 | 1 | 4.4 | 4.2 | 4.2 | 4.2 | 0.00 | 0.00 | 0.00 | 0.00 | 0.10 | 0.09 | 0.11 | 0.09 | 481 |
| 10 | 5 | 200 | 100 | 0 | 0 | 1.5 | 2 | 2 | 3.4 | 3.4 | 3.6 | 3.4 | 0.00 | 0.00 | 0.00 | 0.00 | 0.10 | 0.09 | 0.10 | 0.09 | 499 |
| 10 | 5 | 200 | 100 | 0 | 0 | 2.5 | 0.5 | 1 | 5.1 | 4.8 | 4.8 | 4.6 | 0.01 | 0.01 | 0.01 | 0.01 | 0.11 | 0.09 | 0.11 | 0.10 | 493 |
| 10 | 5 | 200 | 100 | 0 | 0 | 2.5 | 0.5 | 2 | 4.2 | 4.2 | 4.2 | 4.2 | 0.00 | 0.00 | 0.00 | 0.00 | 0.11 | 0.09 | 0.11 | 0.09 | 500 |
| 10 | 5 | 200 | 100 | 0 | 0.2 | 1.5 | 2 | 1 | 44.9 | 44.9 | 44.9 | 44.9 | 0.00 | 0.02 | 0.00 | 0.02 | 0.11 | 0.09 | 0.11 | 0.09 | 474 |
| 10 | 5 | 200 | 100 | 0 | 0.2 | 1.5 | 2 | 2 | 46.9 | 46.8 | 46.6 | 46.6 | 0.01 | 0.02 | 0.01 | 0.01 | 0.10 | 0.09 | 0.11 | 0.10 | 499 |
| 10 | 5 | 200 | 100 | 0 | 0.2 | 2.5 | 0.5 | 1 | 40.0 | 39.2 | 40.2 | 39.6 | 0.01 | 0.04 | 0.01 | 0.04 | 0.11 | 0.09 | 0.11 | 0.10 | 492 |
| 10 | 5 | 200 | 100 | 0 | 0.2 | 2.5 | 0.5 | 2 | 41.2 | 40.8 | 42.0 | 41.6 | 0.01 | 0.03 | 0.00 | 0.03 | 0.11 | 0.09 | 0.11 | 0.10 | 500 |
| 10 | 5 | 200 | 100 | 0 | 0.5 | 1.5 | 2 | 1 | 99.8 | 99.8 | 99.8 | 99.8 | 0.01 | 0.05 | 0.02 | 0.04 | 0.10 | 0.09 | 0.10 | 0.09 | 476 |
| 10 | 5 | 200 | 100 | 0 | 0.5 | 1.5 | 2 | 2 | 99.2 | 99.0 | 99.0 | 99.0 | 0.00 | 0.06 | 0.01 | 0.05 | 0.11 | 0.10 | 0.11 | 0.10 | 499 |
| 10 | 5 | 200 | 100 | 0 | 0.5 | 2.5 | 0.5 | 1 | 99.6 | 99.6 | 99.6 | 99.6 | 0.01 | 0.06 | 0.01 | 0.05 | 0.11 | 0.10 | 0.11 | 0.10 | 493 |
| 10 | 5 | 200 | 100 | 0 | 0.5 | 2.5 | 0.5 | 2 | 99.8 | 99.8 | 99.8 | 99.8 | 0.00 | 0.06 | 0.01 | 0.05 | 0.11 | 0.10 | 0.12 | 0.11 | 500 |
| 10 | 5 | 200 | 100 | 0.5 | 0 | 1.5 | 2 | 1 | 4.1 | 3.6 | 4.0 | 3.6 | 0.01 | 0.01 | 0.01 | 0.01 | 0.11 | 0.09 | 0.11 | 0.09 | 466 |
| 10 | 5 | 200 | 100 | 0.5 | 0 | 1.5 | 2 | 2 | 4.4 | 4.4 | 4.4 | 4.4 | 0.00 | 0.00 | 0.00 | 0.00 | 0.10 | 0.09 | 0.11 | 0.09 | 499 |
| 10 | 5 | 200 | 100 | 0.5 | 0 | 2.5 | 0.5 | 1 | 4.8 | 4.6 | 4.6 | 4.6 | 0.00 | 0.00 | 0.00 | 0.00 | 0.10 | 0.09 | 0.11 | 0.09 | 484 |
| 10 | 5 | 200 | 100 | 0.5 | 0 | 2.5 | 0.5 | 2 | 4.8 | 4.8 | 4.8 | 4.8 | 0.00 | 0.00 | 0.00 | 0.00 | 0.11 | 0.09 | 0.11 | 0.10 | 500 |
| 10 | 5 | 200 | 100 | 0.5 | 0.2 | 1.5 | 2 | 1 | 48.6 | 48.6 | 48.6 | 48.6 | 0.00 | 0.02 | 0.01 | 0.02 | 0.11 | 0.10 | 0.11 | 0.10 | 477 |
| 10 | 5 | 200 | 100 | 0.5 | 0.2 | 1.5 | 2 | 2 | 49.6 | 49.2 | 49.8 | 49.2 | 0.01 | 0.02 | 0.01 | 0.01 | 0.11 | 0.09 | 0.11 | 0.10 | 500 |
| 10 | 5 | 200 | 100 | 0.5 | 0.2 | 2.5 | 0.5 | 1 | 46.1 | 46.1 | 46.1 | 46.1 | 0.00 | 0.03 | 0.00 | 0.03 | 0.11 | 0.10 | 0.11 | 0.10 | 488 |
| 10 | 5 | 200 | 100 | 0.5 | 0.2 | 2.5 | 0.5 | 2 | 43.2 | 43.0 | 43.8 | 43.4 | 0.00 | 0.03 | 0.00 | 0.02 | 0.11 | 0.09 | 0.11 | 0.09 | 500 |
| 10 | 5 | 200 | 100 | 0.5 | 0.5 | 1.5 | 2 | 1 | 100.0 | 96.0 | 96.0 | 96.0 | 0.00 | 0.06 | 0.01 | 0.05 | 0.11 | 0.09 | 0.11 | 0.10 | 480 |
| 10 | 5 | 200 | 100 | 0.5 | 0.5 | 1.5 | 2 | 2 | 99.6 | 99.6 | 99.6 | 99.6 | 0.00 | 0.06 | 0.00 | 0.05 | 0.10 | 0.09 | 0.11 | 0.10 | 500 |
| 10 | 5 | 200 | 100 | 0.5 | 0.5 | 2.5 | 0.5 | 1 | 99.6 | 99.6 | 99.6 | 99.6 | 0.00 | 0.07 | 0.01 | 0.06 | 0.11 | 0.10 | 0.11 | 0.10 | 487 |
| 10 | 5 | 200 | 100 | 0.5 | 0.5 | 2.5 | 0.5 | 2 | 99.6 | 99.6 | 99.6 | 99.6 | 0.00 | 0.06 | 0.01 | 0.05 | 0.12 | 0.10 | 0.12 | 0.11 | 500 |
| 10 | 5 | 200 | 100 | 2 | 0 | 1.5 | 2 | 1 | 4.6 | 4.2 | 4.2 | 4.2 | 0.00 | 0.00 | 0.00 | 0.00 | 0.11 | 0.09 | 0.11 | 0.09 | 475 |
| 10 | 5 | 200 | 100 | 2 | 0 | 1.5 | 2 | 2 | 5.0 | 5.0 | 5.2 | 5.2 | 0.00 | 0.00 | 0.00 | 0.00 | 0.11 | 0.09 | 0.11 | 0.09 | 498 |
| 10 | 5 | 200 | 100 | 2 | 0 | 2.5 | 0.5 | 1 | 5.4 | 5.2 | 5.2 | 5.2 | 0.00 | 0.00 | 0.00 | 0.00 | 0.11 | 0.09 | 0.11 | 0.09 | 486 |
| 10 | 5 | 200 | 100 | 2 | 0 | 2.5 | 0.5 | 2 | 5.4 | 5.2 | 5.2 | 5.2 | 0.00 | 0.00 | 0.00 | 0.00 | 0.11 | 0.09 | 0.11 | 0.09 | 499 |
| 10 | 5 | 200 | 100 | 2 | 0.2 | 1.5 | 2 | 1 | 38.5 | 38.5 | 38.5 | 38.5 | 0.01 | 0.05 | 0.00 | 0.04 | 0.11 | 0.09 | 0.12 | 0.09 | 478 |
| 10 | 5 | 200 | 100 | 2 | 0.2 | 1.5 | 2 | 2 | 40.2 | 39.6 | 40.2 | 39.6 | 0.00 | 0.04 | 0.01 | 0.04 | 0.11 | 0.09 | 0.11 | 0.09 | 500 |
| 10 | 5 | 200 | 100 | 2 | 0.2 | 2.5 | 0.5 | 1 | 44.6 | 42.8 | 43.2 | 42.2 | 0.01 | 0.03 | 0.02 | 0.02 | 0.11 | 0.09 | 0.12 | 0.10 | 491 |
| 10 | 5 | 200 | 100 | 2 | 0.2 | 2.5 | 0.5 | 2 | 47.9 | 47.2 | 48.0 | 47.2 | 0.00 | 0.04 | 0.01 | 0.03 | 0.11 | 0.09 | 0.12 | 0.10 | 499 |
| 10 | 5 | 200 | 100 | 2 | 0.5 | 1.5 | 2 | 1 | 99.6 | 92.4 | 92.4 | 92.4 | 0.01 | 0.11 | 0.01 | 0.09 | 0.11 | 0.09 | 0.12 | 0.10 | 464 |
| 10 | 5 | 200 | 100 | 2 | 0.5 | 1.5 | 2 | 2 | 99.6 | 99.4 | 99.4 | 99.4 | 0.00 | 0.10 | 0.01 | 0.09 | 0.11 | 0.09 | 0.12 | 0.10 | 499 |
| 10 | 5 | 200 | 100 | 2 | 0.5 | 2.5 | 0.5 | 1 | 99.6 | 99.6 | 99.6 | 99.6 | 0.00 | 0.09 | 0.02 | 0.07 | 0.11 | 0.09 | 0.12 | 0.10 | 487 |
| 10 | 5 | 200 | 100 | 2 | 0.5 | 2.5 | 0.5 | 2 | 99.6 | 99.6 | 99.6 | 99.6 | 0.00 | 0.09 | 0.02 | 0.07 | 0.12 | 0.10 | 0.12 | 0.10 | 500 |
| 10 | 5 | 200 | 250 | 0 | 0 | 1.5 | 2 | 1 | 5.0 | 5.0 | 5.2 | 5.0 | 0.00 | 0.00 | 0.00 | 0.00 | 0.11 | 0.09 | 0.11 | 0.09 | 500 |
| 10 | 5 | 200 | 250 | 0 | 0 | 1.5 | 2 | 2 | 5.4 | 5.2 | 5.4 | 5.2 | 0.00 | 0.00 | 0.00 | 0.00 | 0.11 | 0.10 | 0.11 | 0.10 | 500 |
| 10 | 5 | 200 | 250 | 0 | 0 | 2.5 | 0.5 | 1 | 6.2 | 6.0 | 6.4 | 6.2 | 0.01 | 0.01 | 0.01 | 0.01 | 0.11 | 0.10 | 0.11 | 0.10 | 500 |
| 10 | 5 | 200 | 250 | 0 | 0 | 2.5 | 0.5 | 2 | 3.2 | 3.0 | 2.8 | 2.8 | 0.00 | 0.00 | 0.00 | 0.00 | 0.10 | 0.09 | 0.10 | 0.09 | 500 |
| 10 | 5 | 200 | 250 | 0 | 0.2 | 1.5 | 2 | 1 | 46.0 | 45.8 | 45.4 | 45.4 | 0.00 | 0.02 | 0.00 | 0.02 | 0.10 | 0.09 | 0.10 | 0.09 | 500 |
| 10 | 5 | 200 | 250 | 0 | 0.2 | 1.5 | 2 | 2 | 45.2 | 45.0 | 45.0 | 45.0 | 0.00 | 0.03 | 0.00 | 0.02 | 0.11 | 0.10 | 0.11 | 0.10 | 500 |
| 10 | 5 | 200 | 250 | 0 | 0.2 | 2.5 | 0.5 | 1 | 46.4 | 45.8 | 46.2 | 46.0 | 0.00 | 0.02 | 0.00 | 0.02 | 0.11 | 0.09 | 0.11 | 0.09 | 500 |
| 10 | 5 | 200 | 250 | 0 | 0.2 | 2.5 | 0.5 | 2 | 47.0 | 46.8 | 46.8 | 46.8 | 0.00 | 0.03 | 0.00 | 0.03 | 0.11 | 0.10 | 0.11 | 0.10 | 500 |
| 10 | 5 | 200 | 250 | 0 | 0.5 | 1.5 | 2 | 1 | 98.8 | 98.8 | 98.8 | 98.8 | 0.00 | 0.05 | 0.00 | 0.05 | 0.11 | 0.10 | 0.11 | 0.10 | 500 |
| 10 | 5 | 200 | 250 | 0 | 0.5 | 1.5 | 2 | 2 | 99.4 | 99.2 | 99.4 | 99.2 | 0.00 | 0.06 | 0.00 | 0.06 | 0.11 | 0.10 | 0.11 | 0.10 | 500 |
| 10 | 5 | 200 | 250 | 0 | 0.5 | 2.5 | 0.5 | 1 | 99.2 | 99.2 | 99.2 | 99.2 | 0.00 | 0.06 | 0.00 | 0.06 | 0.11 | 0.10 | 0.11 | 0.10 | 500 |
| 10 | 5 | 200 | 250 | 0 | 0.5 | 2.5 | 0.5 | 2 | 99.8 | 99.8 | 99.8 | 99.8 | 0.00 | 0.06 | 0.00 | 0.06 | 0.10 | 0.09 | 0.10 | 0.09 | 500 |
| 10 | 5 | 200 | 250 | 0.5 | 0 | 1.5 | 2 | 1 | 5.6 | 5.6 | 6.0 | 5.6 | 0.01 | 0.01 | 0.01 | 0.01 | 0.10 | 0.09 | 0.10 | 0.09 | 500 |
| 10 | 5 | 200 | 250 | 0.5 | 0 | 1.5 | 2 | 2 | 5.4 | 5.4 | 5.4 | 5.4 | 0.01 | 0.01 | 0.01 | 0.01 | 0.11 | 0.09 | 0.11 | 0.10 | 500 |
| 10 | 5 | 200 | 250 | 0.5 | 0 | 2.5 | 0.5 | 1 | 4.4 | 4.4 | 4.4 | 4.4 | 0.00 | 0.00 | 0.00 | 0.00 | 0.11 | 0.09 | 0.11 | 0.09 | 500 |
| 10 | 5 | 200 | 250 | 0.5 | 0 | 2.5 | 0.5 | 2 | 6.4 | 6.4 | 6.4 | 6.2 | 0.00 | 0.00 | 0.00 | 0.00 | 0.11 | 0.09 | 0.11 | 0.09 | 500 |
| 10 | 5 | 200 | 250 | 0.5 | 0.2 | 1.5 | 2 | 1 | 47.2 | 46.8 | 47.2 | 46.4 | 0.00 | 0.02 | 0.00 | 0.02 | 0.11 | 0.09 | 0.11 | 0.10 | 500 |
| 10 | 5 | 200 | 250 | 0.5 | 0.2 | 1.5 | 2 | 2 | 46.6 | 46.4 | 46.6 | 46.6 | 0.00 | 0.02 | 0.00 | 0.02 | 0.11 | 0.09 | 0.11 | 0.09 | 500 |
| 10 | 5 | 200 | 250 | 0.5 | 0.2 | 2.5 | 0.5 | 1 | 48.0 | 48.0 | 48.0 | 47.8 | 0.00 | 0.03 | 0.00 | 0.03 | 0.11 | 0.09 | 0.11 | 0.09 | 500 |
| 10 | 5 | 200 | 250 | 0.5 | 0.2 | 2.5 | 0.5 | 2 | 45.2 | 45.0 | 45.4 | 44.8 | 0.01 | 0.03 | 0.01 | 0.03 | 0.11 | 0.10 | 0.11 | 0.10 | 500 |
| 10 | 5 | 200 | 250 | 0.5 | 0.5 | 1.5 | 2 | 1 | 99.6 | 99.6 | 99.6 | 99.6 | 0.00 | 0.05 | 0.01 | 0.05 | 0.11 | 0.10 | 0.11 | 0.10 | 500 |
| 10 | 5 | 200 | 250 | 0.5 | 0.5 | 1.5 | 2 | 2 | 99.8 | 99.6 | 99.8 | 99.6 | 0.00 | 0.05 | 0.01 | 0.05 | 0.11 | 0.09 | 0.11 | 0.10 | 500 |
| 10 | 5 | 200 | 250 | 0.5 | 0.5 | 2.5 | 0.5 | 1 | 99.4 | 99.4 | 99.4 | 99.4 | 0.00 | 0.06 | 0.00 | 0.06 | 0.11 | 0.10 | 0.12 | 0.10 | 500 |
| 10 | 5 | 200 | 250 | 0.5 | 0.5 | 2.5 | 0.5 | 2 | 99.8 | 99.8 | 99.8 | 99.8 | 0.01 | 0.06 | 0.01 | 0.06 | 0.11 | 0.09 | 0.11 | 0.10 | 500 |
| 10 | 5 | 200 | 250 | 2 | 0 | 1.5 | 2 | 1 | 4.4 | 4.2 | 4.4 | 4.0 | 0.00 | 0.00 | 0.00 | 0.00 | 0.11 | 0.09 | 0.11 | 0.09 | 499 |
| 10 | 5 | 200 | 250 | 2 | 0 | 1.5 | 2 | 2 | 5.2 | 5.0 | 5.2 | 5.0 | 0.01 | 0.01 | 0.01 | 0.01 | 0.11 | 0.09 | 0.11 | 0.09 | 500 |
| 10 | 5 | 200 | 250 | 2 | 0 | 2.5 | 0.5 | 1 | 5.2 | 4.8 | 5.2 | 5.0 | 0.00 | 0.00 | 0.00 | 0.00 | 0.11 | 0.09 | 0.11 | 0.09 | 500 |
| 10 | 5 | 200 | 250 | 2 | 0 | 2.5 | 0.5 | 2 | 5.0 | 4.8 | 4.8 | 4.8 | 0.00 | 0.00 | 0.00 | 0.00 | 0.11 | 0.09 | 0.12 | 0.09 | 500 |
| 10 | 5 | 200 | 250 | 2 | 0.2 | 1.5 | 2 | 1 | 42.0 | 41.6 | 42.0 | 41.6 | 0.00 | 0.04 | 0.00 | 0.04 | 0.11 | 0.09 | 0.11 | 0.09 | 500 |
| 10 | 5 | 200 | 250 | 2 | 0.2 | 1.5 | 2 | 2 | 44.6 | 44.2 | 44.4 | 44.2 | 0.01 | 0.04 | 0.01 | 0.04 | 0.12 | 0.09 | 0.12 | 0.09 | 500 |
| 10 | 5 | 200 | 250 | 2 | 0.2 | 2.5 | 0.5 | 1 | 46.2 | 45.6 | 46.2 | 45.8 | 0.01 | 0.03 | 0.01 | 0.03 | 0.12 | 0.09 | 0.12 | 0.10 | 500 |
| 10 | 5 | 200 | 250 | 2 | 0.2 | 2.5 | 0.5 | 2 | 44.2 | 43.6 | 44.0 | 43.8 | 0.00 | 0.04 | 0.01 | 0.03 | 0.11 | 0.09 | 0.11 | 0.09 | 500 |
| 10 | 5 | 200 | 250 | 2 | 0.5 | 1.5 | 2 | 1 | 99.6 | 99.6 | 99.6 | 99.4 | 0.01 | 0.10 | 0.01 | 0.09 | 0.12 | 0.10 | 0.12 | 0.10 | 500 |
| 10 | 5 | 200 | 250 | 2 | 0.5 | 1.5 | 2 | 2 | 99.8 | 99.8 | 99.8 | 99.8 | 0.00 | 0.11 | 0.00 | 0.10 | 0.11 | 0.09 | 0.12 | 0.09 | 500 |
| 10 | 5 | 200 | 250 | 2 | 0.5 | 2.5 | 0.5 | 1 | 99.4 | 99.4 | 99.4 | 99.4 | 0.01 | 0.08 | 0.02 | 0.08 | 0.11 | 0.09 | 0.11 | 0.10 | 500 |
| 10 | 5 | 200 | 250 | 2 | 0.5 | 2.5 | 0.5 | 2 | 99.4 | 99.4 | 99.4 | 99.4 | 0.01 | 0.08 | 0.02 | 0.08 | 0.11 | 0.09 | 0.11 | 0.10 | 500 |
| 10 | 5 | 200 | 500 | 0 | 0 | 1.5 | 2 | 1 | 4.8 | 4.6 | 4.6 | 4.6 | 0.00 | 0.00 | 0.00 | 0.00 | 0.11 | 0.09 | 0.11 | 0.09 | 500 |
| 10 | 5 | 200 | 500 | 0 | 0 | 1.5 | 2 | 2 | 6.0 | 6.0 | 6.0 | 6.0 | 0.00 | 0.00 | 0.00 | 0.00 | 0.11 | 0.09 | 0.11 | 0.09 | 500 |
| 10 | 5 | 200 | 500 | 0 | 0 | 2.5 | 0.5 | 1 | 5.2 | 5.2 | 5.2 | 5.0 | 0.02 | 0.01 | 0.02 | 0.01 | 0.11 | 0.09 | 0.11 | 0.09 | 500 |
| 10 | 5 | 200 | 500 | 0 | 0 | 2.5 | 0.5 | 2 | 4.6 | 4.6 | 4.8 | 4.6 | 0.01 | 0.01 | 0.01 | 0.01 | 0.11 | 0.09 | 0.11 | 0.09 | 500 |
| 10 | 5 | 200 | 500 | 0 | 0.2 | 1.5 | 2 | 1 | 52.4 | 52.2 | 52.8 | 52.4 | 0.00 | 0.02 | 0.00 | 0.02 | 0.11 | 0.09 | 0.11 | 0.09 | 500 |
| 10 | 5 | 200 | 500 | 0 | 0.2 | 1.5 | 2 | 2 | 52.4 | 52.2 | 52.8 | 52.4 | 0.00 | 0.02 | 0.00 | 0.02 | 0.11 | 0.09 | 0.11 | 0.09 | 500 |
| 10 | 5 | 200 | 500 | 0 | 0.2 | 2.5 | 0.5 | 1 | 41.2 | 41.0 | 41.6 | 41.2 | 0.01 | 0.04 | 0.01 | 0.04 | 0.10 | 0.09 | 0.10 | 0.09 | 500 |
| 10 | 5 | 200 | 500 | 0 | 0.2 | 2.5 | 0.5 | 2 | 42.0 | 41.0 | 42.0 | 41.4 | 0.00 | 0.03 | 0.00 | 0.03 | 0.10 | 0.09 | 0.10 | 0.09 | 500 |
| 10 | 5 | 200 | 500 | 0 | 0.5 | 1.5 | 2 | 1 | 99.4 | 99.4 | 99.4 | 99.4 | 0.00 | 0.05 | 0.00 | 0.05 | 0.11 | 0.10 | 0.11 | 0.10 | 500 |
| 10 | 5 | 200 | 500 | 0 | 0.5 | 1.5 | 2 | 2 | 99.8 | 99.8 | 99.8 | 99.8 | 0.01 | 0.04 | 0.01 | 0.05 | 0.10 | 0.09 | 0.10 | 0.09 | 500 |
| 10 | 5 | 200 | 500 | 0 | 0.5 | 2.5 | 0.5 | 1 | 99.2 | 99.2 | 99.2 | 99.2 | 0.00 | 0.07 | 0.00 | 0.07 | 0.11 | 0.10 | 0.11 | 0.10 | 500 |
| 10 | 5 | 200 | 500 | 0 | 0.5 | 2.5 | 0.5 | 2 | 100.0 | 100.0 | 100.0 | 100.0 | 0.00 | 0.06 | 0.00 | 0.06 | 0.11 | 0.10 | 0.11 | 0.10 | 500 |
| 10 | 5 | 200 | 500 | 0.5 | 0 | 1.5 | 2 | 1 | 3.6 | 3.6 | 3.6 | 3.6 | 0.00 | 0.00 | 0.00 | 0.00 | 0.10 | 0.09 | 0.10 | 0.09 | 500 |
| 10 | 5 | 200 | 500 | 0.5 | 0 | 1.5 | 2 | 2 | 3.6 | 3.6 | 3.6 | 3.6 | 0.00 | 0.00 | 0.00 | 0.00 | 0.10 | 0.09 | 0.10 | 0.09 | 500 |
| 10 | 5 | 200 | 500 | 0.5 | 0 | 2.5 | 0.5 | 1 | 3.8 | 3.8 | 3.8 | 3.8 | 0.00 | 0.00 | 0.00 | 0.00 | 0.10 | 0.09 | 0.10 | 0.09 | 500 |
| 10 | 5 | 200 | 500 | 0.5 | 0 | 2.5 | 0.5 | 2 | 6.2 | 6.2 | 6.4 | 6.2 | 0.00 | 0.00 | 0.00 | 0.00 | 0.11 | 0.10 | 0.11 | 0.10 | 500 |
| 10 | 5 | 200 | 500 | 0.5 | 0.2 | 1.5 | 2 | 1 | 46.0 | 45.4 | 46.0 | 45.6 | 0.00 | 0.02 | 0.00 | 0.02 | 0.11 | 0.09 | 0.11 | 0.09 | 500 |
| 10 | 5 | 200 | 500 | 0.5 | 0.2 | 1.5 | 2 | 2 | 46.0 | 45.4 | 46.0 | 45.6 | 0.00 | 0.02 | 0.00 | 0.02 | 0.11 | 0.09 | 0.11 | 0.09 | 500 |
| 10 | 5 | 200 | 500 | 0.5 | 0.2 | 2.5 | 0.5 | 1 | 47.4 | 47.2 | 47.6 | 47.4 | 0.00 | 0.02 | 0.00 | 0.02 | 0.11 | 0.09 | 0.11 | 0.10 | 500 |
| 10 | 5 | 200 | 500 | 0.5 | 0.2 | 2.5 | 0.5 | 2 | 45.8 | 45.6 | 45.8 | 45.6 | 0.00 | 0.03 | 0.00 | 0.03 | 0.11 | 0.10 | 0.11 | 0.10 | 500 |
| 10 | 5 | 200 | 500 | 0.5 | 0.5 | 1.5 | 2 | 1 | 99.8 | 99.8 | 99.8 | 99.8 | 0.01 | 0.05 | 0.00 | 0.05 | 0.11 | 0.10 | 0.11 | 0.09 | 500 |
| 10 | 5 | 200 | 500 | 0.5 | 0.5 | 1.5 | 2 | 2 | 99.6 | 99.6 | 99.8 | 99.6 | 0.00 | 0.06 | 0.00 | 0.06 | 0.11 | 0.10 | 0.11 | 0.10 | 500 |
| 10 | 5 | 200 | 500 | 0.5 | 0.5 | 2.5 | 0.5 | 1 | 99.2 | 99.2 | 99.2 | 99.2 | 0.00 | 0.07 | 0.00 | 0.07 | 0.11 | 0.10 | 0.11 | 0.10 | 500 |
| 10 | 5 | 200 | 500 | 0.5 | 0.5 | 2.5 | 0.5 | 2 | 99.8 | 99.8 | 99.8 | 99.8 | 0.00 | 0.06 | 0.00 | 0.06 | 0.11 | 0.09 | 0.11 | 0.09 | 500 |
| 10 | 5 | 200 | 500 | 2 | 0 | 1.5 | 2 | 1 | 6.0 | 5.6 | 6.0 | 5.4 | 0.00 | 0.00 | 0.00 | 0.00 | 0.11 | 0.09 | 0.11 | 0.09 | 500 |
| 10 | 5 | 200 | 500 | 2 | 0 | 1.5 | 2 | 2 | 6.2 | 5.6 | 6.2 | 5.4 | 0.00 | 0.00 | 0.00 | 0.00 | 0.11 | 0.09 | 0.11 | 0.09 | 500 |
| 10 | 5 | 200 | 500 | 2 | 0 | 2.5 | 0.5 | 1 | 5.2 | 4.8 | 5.2 | 5.0 | 0.01 | 0.01 | 0.01 | 0.01 | 0.11 | 0.09 | 0.11 | 0.09 | 500 |
| 10 | 5 | 200 | 500 | 2 | 0 | 2.5 | 0.5 | 2 | 4.2 | 4.0 | 3.8 | 3.8 | 0.01 | 0.00 | 0.01 | 0.00 | 0.11 | 0.09 | 0.11 | 0.09 | 500 |
| 10 | 5 | 200 | 500 | 2 | 0.2 | 1.5 | 2 | 1 | 42.8 | 42.2 | 42.6 | 42.0 | 0.00 | 0.04 | 0.00 | 0.04 | 0.11 | 0.09 | 0.11 | 0.09 | 500 |
| 10 | 5 | 200 | 500 | 2 | 0.2 | 1.5 | 2 | 2 | 42.8 | 42.2 | 42.6 | 42.0 | 0.00 | 0.04 | 0.00 | 0.04 | 0.11 | 0.09 | 0.11 | 0.09 | 500 |
| 10 | 5 | 200 | 500 | 2 | 0.2 | 2.5 | 0.5 | 1 | 46.0 | 45.4 | 46.0 | 45.4 | 0.00 | 0.04 | 0.00 | 0.03 | 0.11 | 0.09 | 0.11 | 0.09 | 500 |
| 10 | 5 | 200 | 500 | 2 | 0.2 | 2.5 | 0.5 | 2 | 43.8 | 43.6 | 43.6 | 43.6 | 0.00 | 0.04 | 0.00 | 0.04 | 0.11 | 0.09 | 0.11 | 0.09 | 500 |
| 10 | 5 | 200 | 500 | 2 | 0.5 | 1.5 | 2 | 1 | 98.6 | 98.6 | 98.6 | 98.6 | 0.01 | 0.10 | 0.01 | 0.09 | 0.11 | 0.09 | 0.12 | 0.09 | 500 |
| 10 | 5 | 200 | 500 | 2 | 0.5 | 1.5 | 2 | 2 | 99.0 | 99.0 | 99.2 | 99.0 | 0.01 | 0.10 | 0.01 | 0.10 | 0.12 | 0.10 | 0.12 | 0.10 | 500 |
| 10 | 5 | 200 | 500 | 2 | 0.5 | 2.5 | 0.5 | 1 | 99.8 | 99.8 | 99.8 | 99.8 | 0.00 | 0.09 | 0.00 | 0.09 | 0.11 | 0.09 | 0.11 | 0.09 | 500 |
| 10 | 5 | 200 | 500 | 2 | 0.5 | 2.5 | 0.5 | 2 | 99.2 | 99.2 | 99.2 | 99.2 | 0.01 | 0.09 | 0.01 | 0.08 | 0.12 | 0.10 | 0.12 | 0.10 | 500 |
| 10 | 5 | 500 | 100 | 0 | 0 | 1.5 | 2 | 1 | 6.6 | 6.4 | 6.6 | 6.6 | 0.00 | 0.00 | 0.00 | 0.00 | 0.07 | 0.06 | 0.07 | 0.06 | 483 |
| 10 | 5 | 500 | 100 | 0 | 0 | 1.5 | 2 | 2 | 5.2 | 5.2 | 5.2 | 5.2 | 0.00 | 0.00 | 0.00 | 0.00 | 0.07 | 0.06 | 0.07 | 0.06 | 499 |
| 10 | 5 | 500 | 100 | 0 | 0 | 2.5 | 0.5 | 1 | 4.7 | 4.4 | 4.6 | 4.6 | 0.00 | 0.00 | 0.00 | 0.00 | 0.06 | 0.06 | 0.07 | 0.06 | 486 |
| 10 | 5 | 500 | 100 | 0 | 0 | 2.5 | 0.5 | 2 | 6.6 | 6.6 | 6.6 | 6.4 | 0.00 | 0.00 | 0.00 | 0.00 | 0.07 | 0.06 | 0.07 | 0.06 | 499 |
| 10 | 5 | 500 | 100 | 0 | 0.2 | 1.5 | 2 | 1 | 84.3 | 84.2 | 84.3 | 84.2 | 0.00 | 0.03 | 0.00 | 0.02 | 0.07 | 0.06 | 0.07 | 0.06 | 478 |
| 10 | 5 | 500 | 100 | 0 | 0.2 | 1.5 | 2 | 2 | 86.2 | 86.0 | 85.8 | 85.8 | 0.00 | 0.02 | 0.01 | 0.02 | 0.07 | 0.06 | 0.07 | 0.06 | 499 |
| 10 | 5 | 500 | 100 | 0 | 0.2 | 2.5 | 0.5 | 1 | 84.1 | 84.0 | 84.1 | 84.0 | 0.00 | 0.02 | 0.01 | 0.02 | 0.07 | 0.06 | 0.07 | 0.06 | 491 |
| 10 | 5 | 500 | 100 | 0 | 0.2 | 2.5 | 0.5 | 2 | 83.6 | 83.6 | 83.6 | 83.6 | 0.00 | 0.03 | 0.01 | 0.02 | 0.07 | 0.06 | 0.07 | 0.06 | 500 |
| 10 | 5 | 500 | 100 | 0 | 0.5 | 1.5 | 2 | 1 | 100.0 | 100.0 | 100.0 | 100.0 | 0.00 | 0.05 | 0.01 | 0.04 | 0.07 | 0.06 | 0.07 | 0.07 | 474 |
| 10 | 5 | 500 | 100 | 0 | 0.5 | 1.5 | 2 | 2 | 100.0 | 100.0 | 100.0 | 100.0 | 0.00 | 0.05 | 0.01 | 0.04 | 0.07 | 0.06 | 0.07 | 0.07 | 499 |
| 10 | 5 | 500 | 100 | 0 | 0.5 | 2.5 | 0.5 | 1 | 100.0 | 100.0 | 100.0 | 100.0 | 0.00 | 0.06 | 0.01 | 0.05 | 0.06 | 0.06 | 0.07 | 0.06 | 486 |
| 10 | 5 | 500 | 100 | 0 | 0.5 | 2.5 | 0.5 | 2 | 100.0 | 100.0 | 100.0 | 100.0 | 0.00 | 0.06 | 0.01 | 0.05 | 0.07 | 0.06 | 0.07 | 0.07 | 499 |
| 10 | 5 | 500 | 100 | 0.5 | 0 | 1.5 | 2 | 1 | 4.9 | 4.6 | 4.6 | 4.6 | 0.00 | 0.00 | 0.00 | 0.00 | 0.07 | 0.06 | 0.07 | 0.06 | 472 |
| 10 | 5 | 500 | 100 | 0.5 | 0 | 1.5 | 2 | 2 | 3.4 | 3.2 | 3.4 | 3.4 | 0.00 | 0.00 | 0.00 | 0.00 | 0.06 | 0.06 | 0.07 | 0.06 | 499 |
| 10 | 5 | 500 | 100 | 0.5 | 0 | 2.5 | 0.5 | 1 | 6.7 | 6.6 | 6.4 | 6.4 | 0.00 | 0.00 | 0.00 | 0.00 | 0.07 | 0.06 | 0.07 | 0.06 | 490 |
| 10 | 5 | 500 | 100 | 0.5 | 0 | 2.5 | 0.5 | 2 | 4.8 | 4.8 | 4.8 | 4.8 | 0.00 | 0.00 | 0.00 | 0.00 | 0.07 | 0.06 | 0.07 | 0.06 | 499 |
| 10 | 5 | 500 | 100 | 0.5 | 0.2 | 1.5 | 2 | 1 | 80.4 | 80.4 | 80.4 | 80.4 | 0.01 | 0.03 | 0.00 | 0.03 | 0.07 | 0.06 | 0.07 | 0.06 | 479 |
| 10 | 5 | 500 | 100 | 0.5 | 0.2 | 1.5 | 2 | 2 | 84.1 | 83.4 | 83.6 | 83.2 | 0.00 | 0.03 | 0.00 | 0.02 | 0.07 | 0.06 | 0.07 | 0.06 | 497 |
| 10 | 5 | 500 | 100 | 0.5 | 0.2 | 2.5 | 0.5 | 1 | 83.4 | 82.2 | 82.2 | 82.2 | 0.00 | 0.03 | 0.01 | 0.02 | 0.07 | 0.06 | 0.07 | 0.06 | 493 |
| 10 | 5 | 500 | 100 | 0.5 | 0.2 | 2.5 | 0.5 | 2 | 79.0 | 78.8 | 79.0 | 78.4 | 0.01 | 0.03 | 0.00 | 0.03 | 0.07 | 0.06 | 0.07 | 0.06 | 500 |
| 10 | 5 | 500 | 100 | 0.5 | 0.5 | 1.5 | 2 | 1 | 100.0 | 100.0 | 100.0 | 100.0 | 0.00 | 0.05 | 0.01 | 0.05 | 0.07 | 0.06 | 0.08 | 0.07 | 473 |
| 10 | 5 | 500 | 100 | 0.5 | 0.5 | 1.5 | 2 | 2 | 100.0 | 100.0 | 100.0 | 100.0 | 0.00 | 0.06 | 0.01 | 0.05 | 0.07 | 0.06 | 0.07 | 0.07 | 499 |
| 10 | 5 | 500 | 100 | 0.5 | 0.5 | 2.5 | 0.5 | 1 | 100.0 | 100.0 | 100.0 | 100.0 | 0.00 | 0.07 | 0.01 | 0.06 | 0.07 | 0.06 | 0.07 | 0.07 | 487 |
| 10 | 5 | 500 | 100 | 0.5 | 0.5 | 2.5 | 0.5 | 2 | 100.0 | 100.0 | 100.0 | 100.0 | 0.00 | 0.06 | 0.01 | 0.06 | 0.07 | 0.06 | 0.08 | 0.07 | 500 |
| 10 | 5 | 500 | 100 | 2 | 0 | 1.5 | 2 | 1 | 5.2 | 5.0 | 5.0 | 5.0 | 0.00 | 0.00 | 0.00 | 0.00 | 0.07 | 0.06 | 0.07 | 0.06 | 478 |
| 10 | 5 | 500 | 100 | 2 | 0 | 1.5 | 2 | 2 | 4.8 | 4.8 | 4.8 | 4.8 | 0.00 | 0.00 | 0.00 | 0.00 | 0.07 | 0.06 | 0.07 | 0.06 | 496 |
| 10 | 5 | 500 | 100 | 2 | 0 | 2.5 | 0.5 | 1 | 5.3 | 5.2 | 5.0 | 5.0 | 0.00 | 0.00 | 0.00 | 0.00 | 0.07 | 0.06 | 0.07 | 0.06 | 487 |
| 10 | 5 | 500 | 100 | 2 | 0 | 2.5 | 0.5 | 2 | 4.4 | 4.4 | 4.2 | 4.2 | 0.00 | 0.00 | 0.00 | 0.00 | 0.07 | 0.06 | 0.07 | 0.06 | 499 |
| 10 | 5 | 500 | 100 | 2 | 0.2 | 1.5 | 2 | 1 | 81.4 | 81.2 | 81.4 | 81.2 | 0.00 | 0.04 | 0.01 | 0.04 | 0.07 | 0.05 | 0.07 | 0.06 | 478 |
| 10 | 5 | 500 | 100 | 2 | 0.2 | 1.5 | 2 | 2 | 81.0 | 80.8 | 81.6 | 81.2 | 0.00 | 0.04 | 0.01 | 0.04 | 0.07 | 0.06 | 0.07 | 0.06 | 499 |
| 10 | 5 | 500 | 100 | 2 | 0.2 | 2.5 | 0.5 | 1 | 79.0 | 77.1 | 79.0 | 77.1 | 0.00 | 0.04 | 0.00 | 0.03 | 0.07 | 0.06 | 0.07 | 0.06 | 490 |
| 10 | 5 | 500 | 100 | 2 | 0.2 | 2.5 | 0.5 | 2 | 82.6 | 82.2 | 82.4 | 82.4 | 0.01 | 0.03 | 0.01 | 0.03 | 0.07 | 0.06 | 0.07 | 0.06 | 500 |
| 10 | 5 | 500 | 100 | 2 | 0.5 | 1.5 | 2 | 1 | 100.0 | 100.0 | 100.0 | 100.0 | 0.00 | 0.11 | 0.01 | 0.09 | 0.07 | 0.06 | 0.08 | 0.07 | 476 |
| 10 | 5 | 500 | 100 | 2 | 0.5 | 1.5 | 2 | 2 | 100.0 | 100.0 | 100.0 | 100.0 | 0.00 | 0.10 | 0.01 | 0.09 | 0.07 | 0.06 | 0.07 | 0.06 | 499 |
| 10 | 5 | 500 | 100 | 2 | 0.5 | 2.5 | 0.5 | 1 | 100.0 | 100.0 | 100.0 | 100.0 | 0.00 | 0.09 | 0.02 | 0.07 | 0.07 | 0.06 | 0.08 | 0.07 | 490 |
| 10 | 5 | 500 | 100 | 2 | 0.5 | 2.5 | 0.5 | 2 | 100.0 | 100.0 | 100.0 | 100.0 | 0.00 | 0.09 | 0.02 | 0.08 | 0.07 | 0.06 | 0.08 | 0.07 | 500 |
| 10 | 5 | 500 | 250 | 0 | 0 | 1.5 | 2 | 1 | 5.6 | 5.4 | 5.6 | 5.6 | 0.00 | 0.00 | 0.00 | 0.00 | 0.07 | 0.06 | 0.07 | 0.06 | 499 |
| 10 | 5 | 500 | 250 | 0 | 0 | 1.5 | 2 | 2 | 4.2 | 4.2 | 4.2 | 4.2 | 0.01 | 0.01 | 0.01 | 0.01 | 0.06 | 0.06 | 0.07 | 0.06 | 500 |
| 10 | 5 | 500 | 250 | 0 | 0 | 2.5 | 0.5 | 1 | 4.2 | 4.2 | 3.8 | 3.8 | 0.00 | 0.00 | 0.00 | 0.00 | 0.07 | 0.06 | 0.07 | 0.06 | 500 |
| 10 | 5 | 500 | 250 | 0 | 0 | 2.5 | 0.5 | 2 | 3.4 | 3.4 | 3.6 | 3.6 | 0.00 | 0.00 | 0.00 | 0.00 | 0.07 | 0.06 | 0.07 | 0.06 | 500 |
| 10 | 5 | 500 | 250 | 0 | 0.2 | 1.5 | 2 | 1 | 84.4 | 84.2 | 84.4 | 84.2 | 0.00 | 0.02 | 0.00 | 0.02 | 0.07 | 0.06 | 0.07 | 0.06 | 500 |
| 10 | 5 | 500 | 250 | 0 | 0.2 | 1.5 | 2 | 2 | 83.2 | 83.2 | 83.4 | 83.2 | 0.00 | 0.03 | 0.00 | 0.02 | 0.07 | 0.06 | 0.07 | 0.06 | 500 |
| 10 | 5 | 500 | 250 | 0 | 0.2 | 2.5 | 0.5 | 1 | 84.8 | 84.8 | 84.8 | 84.8 | 0.00 | 0.02 | 0.01 | 0.02 | 0.07 | 0.06 | 0.07 | 0.06 | 500 |
| 10 | 5 | 500 | 250 | 0 | 0.2 | 2.5 | 0.5 | 2 | 82.2 | 82.2 | 82.6 | 82.2 | 0.00 | 0.03 | 0.00 | 0.03 | 0.07 | 0.06 | 0.07 | 0.06 | 500 |
| 10 | 5 | 500 | 250 | 0 | 0.5 | 1.5 | 2 | 1 | 100.0 | 100.0 | 100.0 | 100.0 | 0.00 | 0.06 | 0.00 | 0.05 | 0.07 | 0.06 | 0.07 | 0.06 | 500 |
| 10 | 5 | 500 | 250 | 0 | 0.5 | 1.5 | 2 | 2 | 100.0 | 100.0 | 100.0 | 100.0 | 0.00 | 0.05 | 0.00 | 0.05 | 0.07 | 0.06 | 0.07 | 0.06 | 500 |
| 10 | 5 | 500 | 250 | 0 | 0.5 | 2.5 | 0.5 | 1 | 100.0 | 100.0 | 100.0 | 100.0 | 0.01 | 0.06 | 0.01 | 0.06 | 0.07 | 0.06 | 0.07 | 0.06 | 500 |
| 10 | 5 | 500 | 250 | 0 | 0.5 | 2.5 | 0.5 | 2 | 100.0 | 100.0 | 100.0 | 100.0 | 0.01 | 0.06 | 0.01 | 0.06 | 0.07 | 0.06 | 0.07 | 0.06 | 500 |
| 10 | 5 | 500 | 250 | 0.5 | 0 | 1.5 | 2 | 1 | 6.2 | 6.2 | 6.2 | 6.2 | 0.00 | 0.00 | 0.00 | 0.00 | 0.07 | 0.06 | 0.07 | 0.06 | 500 |
| 10 | 5 | 500 | 250 | 0.5 | 0 | 1.5 | 2 | 2 | 5.2 | 5.2 | 5.2 | 5.2 | 0.00 | 0.00 | 0.00 | 0.00 | 0.07 | 0.06 | 0.07 | 0.06 | 500 |
| 10 | 5 | 500 | 250 | 0.5 | 0 | 2.5 | 0.5 | 1 | 4.4 | 4.4 | 4.4 | 4.4 | 0.00 | 0.00 | 0.00 | 0.00 | 0.07 | 0.06 | 0.07 | 0.06 | 500 |
| 10 | 5 | 500 | 250 | 0.5 | 0 | 2.5 | 0.5 | 2 | 6.4 | 6.4 | 6.4 | 6.4 | 0.00 | 0.00 | 0.00 | 0.00 | 0.07 | 0.06 | 0.07 | 0.06 | 500 |
| 10 | 5 | 500 | 250 | 0.5 | 0.2 | 1.5 | 2 | 1 | 83.8 | 83.8 | 84.2 | 83.8 | 0.00 | 0.02 | 0.00 | 0.02 | 0.07 | 0.06 | 0.07 | 0.06 | 500 |
| 10 | 5 | 500 | 250 | 0.5 | 0.2 | 1.5 | 2 | 2 | 86.2 | 86.0 | 86.2 | 85.8 | 0.00 | 0.02 | 0.00 | 0.02 | 0.06 | 0.06 | 0.07 | 0.06 | 500 |
| 10 | 5 | 500 | 250 | 0.5 | 0.2 | 2.5 | 0.5 | 1 | 83.8 | 83.8 | 83.8 | 83.8 | 0.00 | 0.03 | 0.00 | 0.03 | 0.07 | 0.06 | 0.07 | 0.06 | 500 |
| 10 | 5 | 500 | 250 | 0.5 | 0.2 | 2.5 | 0.5 | 2 | 81.2 | 81.2 | 81.4 | 81.2 | 0.00 | 0.02 | 0.01 | 0.02 | 0.07 | 0.06 | 0.07 | 0.06 | 500 |
| 10 | 5 | 500 | 250 | 0.5 | 0.5 | 1.5 | 2 | 1 | 100.0 | 100.0 | 100.0 | 100.0 | 0.00 | 0.06 | 0.00 | 0.05 | 0.07 | 0.06 | 0.07 | 0.06 | 500 |
| 10 | 5 | 500 | 250 | 0.5 | 0.5 | 1.5 | 2 | 2 | 100.0 | 100.0 | 100.0 | 100.0 | 0.00 | 0.06 | 0.00 | 0.06 | 0.07 | 0.06 | 0.07 | 0.06 | 500 |
| 10 | 5 | 500 | 250 | 0.5 | 0.5 | 2.5 | 0.5 | 1 | 100.0 | 100.0 | 100.0 | 100.0 | 0.00 | 0.06 | 0.00 | 0.06 | 0.07 | 0.06 | 0.07 | 0.06 | 500 |
| 10 | 5 | 500 | 250 | 0.5 | 0.5 | 2.5 | 0.5 | 2 | 100.0 | 100.0 | 100.0 | 100.0 | 0.00 | 0.06 | 0.00 | 0.06 | 0.07 | 0.06 | 0.07 | 0.07 | 500 |
| 10 | 5 | 500 | 250 | 2 | 0 | 1.5 | 2 | 1 | 6.6 | 6.4 | 6.6 | 6.4 | 0.00 | 0.00 | 0.00 | 0.00 | 0.07 | 0.06 | 0.07 | 0.06 | 500 |
| 10 | 5 | 500 | 250 | 2 | 0 | 1.5 | 2 | 2 | 4.2 | 4.2 | 4.4 | 4.2 | 0.01 | 0.01 | 0.01 | 0.01 | 0.07 | 0.06 | 0.07 | 0.06 | 500 |
| 10 | 5 | 500 | 250 | 2 | 0 | 2.5 | 0.5 | 1 | 5.4 | 5.2 | 5.6 | 5.4 | 0.00 | 0.00 | 0.00 | 0.00 | 0.07 | 0.06 | 0.07 | 0.06 | 500 |
| 10 | 5 | 500 | 250 | 2 | 0 | 2.5 | 0.5 | 2 | 6.4 | 6.4 | 6.4 | 6.4 | 0.01 | 0.01 | 0.01 | 0.01 | 0.07 | 0.06 | 0.07 | 0.06 | 500 |
| 10 | 5 | 500 | 250 | 2 | 0.2 | 1.5 | 2 | 1 | 81.8 | 81.6 | 81.8 | 81.6 | 0.00 | 0.04 | 0.00 | 0.04 | 0.07 | 0.06 | 0.07 | 0.06 | 500 |
| 10 | 5 | 500 | 250 | 2 | 0.2 | 1.5 | 2 | 2 | 81.2 | 81.2 | 81.2 | 81.2 | 0.00 | 0.04 | 0.00 | 0.04 | 0.07 | 0.06 | 0.07 | 0.06 | 500 |
| 10 | 5 | 500 | 250 | 2 | 0.2 | 2.5 | 0.5 | 1 | 80.4 | 80.2 | 80.4 | 80.4 | 0.01 | 0.04 | 0.00 | 0.04 | 0.07 | 0.05 | 0.07 | 0.06 | 500 |
| 10 | 5 | 500 | 250 | 2 | 0.2 | 2.5 | 0.5 | 2 | 82.8 | 82.6 | 83.0 | 82.4 | 0.00 | 0.04 | 0.00 | 0.03 | 0.07 | 0.06 | 0.07 | 0.06 | 500 |
| 10 | 5 | 500 | 250 | 2 | 0.5 | 1.5 | 2 | 1 | 100.0 | 100.0 | 100.0 | 100.0 | 0.00 | 0.10 | 0.01 | 0.10 | 0.07 | 0.06 | 0.07 | 0.06 | 500 |
| 10 | 5 | 500 | 250 | 2 | 0.5 | 1.5 | 2 | 2 | 100.0 | 100.0 | 100.0 | 100.0 | 0.00 | 0.10 | 0.01 | 0.10 | 0.07 | 0.06 | 0.07 | 0.06 | 500 |
| 10 | 5 | 500 | 250 | 2 | 0.5 | 2.5 | 0.5 | 1 | 100.0 | 100.0 | 100.0 | 100.0 | 0.00 | 0.09 | 0.00 | 0.09 | 0.07 | 0.06 | 0.07 | 0.06 | 500 |
| 10 | 5 | 500 | 250 | 2 | 0.5 | 2.5 | 0.5 | 2 | 100.0 | 100.0 | 100.0 | 100.0 | 0.00 | 0.09 | 0.01 | 0.08 | 0.07 | 0.06 | 0.07 | 0.06 | 500 |
| 10 | 5 | 500 | 500 | 0 | 0 | 1.5 | 2 | 1 | 4.8 | 4.8 | 4.8 | 4.8 | 0.00 | 0.00 | 0.00 | 0.00 | 0.07 | 0.06 | 0.07 | 0.06 | 500 |
| 10 | 5 | 500 | 500 | 0 | 0 | 1.5 | 2 | 2 | 4.8 | 4.8 | 4.8 | 4.8 | 0.00 | 0.00 | 0.00 | 0.00 | 0.07 | 0.06 | 0.07 | 0.06 | 500 |
| 10 | 5 | 500 | 500 | 0 | 0 | 2.5 | 0.5 | 1 | 5.2 | 5.2 | 5.2 | 5.2 | 0.00 | 0.00 | 0.00 | 0.00 | 0.07 | 0.06 | 0.07 | 0.06 | 500 |
| 10 | 5 | 500 | 500 | 0 | 0 | 2.5 | 0.5 | 2 | 6.2 | 6.2 | 6.2 | 6.0 | 0.00 | 0.00 | 0.00 | 0.00 | 0.07 | 0.06 | 0.07 | 0.06 | 500 |
| 10 | 5 | 500 | 500 | 0 | 0.2 | 1.5 | 2 | 1 | 81.8 | 81.8 | 81.8 | 81.8 | 0.00 | 0.02 | 0.00 | 0.02 | 0.07 | 0.06 | 0.07 | 0.06 | 500 |
| 10 | 5 | 500 | 500 | 0 | 0.2 | 1.5 | 2 | 2 | 81.8 | 81.8 | 81.8 | 81.8 | 0.00 | 0.02 | 0.00 | 0.02 | 0.07 | 0.06 | 0.07 | 0.06 | 500 |
| 10 | 5 | 500 | 500 | 0 | 0.2 | 2.5 | 0.5 | 1 | 86.4 | 86.4 | 86.4 | 86.4 | 0.01 | 0.02 | 0.01 | 0.02 | 0.07 | 0.06 | 0.07 | 0.06 | 500 |
| 10 | 5 | 500 | 500 | 0 | 0.2 | 2.5 | 0.5 | 2 | 85.6 | 85.4 | 85.8 | 85.6 | 0.00 | 0.02 | 0.00 | 0.02 | 0.07 | 0.06 | 0.07 | 0.06 | 500 |
| 10 | 5 | 500 | 500 | 0 | 0.5 | 1.5 | 2 | 1 | 100.0 | 100.0 | 100.0 | 100.0 | 0.00 | 0.05 | 0.00 | 0.05 | 0.07 | 0.06 | 0.07 | 0.06 | 500 |
| 10 | 5 | 500 | 500 | 0 | 0.5 | 1.5 | 2 | 2 | 100.0 | 100.0 | 100.0 | 100.0 | 0.01 | 0.05 | 0.01 | 0.05 | 0.07 | 0.06 | 0.07 | 0.06 | 500 |
| 10 | 5 | 500 | 500 | 0 | 0.5 | 2.5 | 0.5 | 1 | 100.0 | 100.0 | 100.0 | 100.0 | 0.00 | 0.07 | 0.00 | 0.07 | 0.06 | 0.06 | 0.06 | 0.06 | 500 |
| 10 | 5 | 500 | 500 | 0 | 0.5 | 2.5 | 0.5 | 2 | 100.0 | 100.0 | 100.0 | 100.0 | 0.00 | 0.07 | 0.00 | 0.06 | 0.07 | 0.06 | 0.07 | 0.06 | 500 |
| 10 | 5 | 500 | 500 | 0.5 | 0 | 1.5 | 2 | 1 | 5.8 | 5.6 | 5.8 | 5.6 | 0.00 | 0.00 | 0.00 | 0.00 | 0.07 | 0.06 | 0.07 | 0.06 | 500 |
| 10 | 5 | 500 | 500 | 0.5 | 0 | 1.5 | 2 | 2 | 5.8 | 5.6 | 5.8 | 5.6 | 0.00 | 0.00 | 0.00 | 0.00 | 0.07 | 0.06 | 0.07 | 0.06 | 500 |
| 10 | 5 | 500 | 500 | 0.5 | 0 | 2.5 | 0.5 | 1 | 5.6 | 5.6 | 5.6 | 5.6 | 0.00 | 0.00 | 0.00 | 0.00 | 0.07 | 0.06 | 0.07 | 0.06 | 500 |
| 10 | 5 | 500 | 500 | 0.5 | 0 | 2.5 | 0.5 | 2 | 4.6 | 4.6 | 4.6 | 4.6 | 0.00 | 0.00 | 0.00 | 0.00 | 0.07 | 0.06 | 0.07 | 0.06 | 500 |
| 10 | 5 | 500 | 500 | 0.5 | 0.2 | 1.5 | 2 | 1 | 83.0 | 82.8 | 83.0 | 82.8 | 0.00 | 0.03 | 0.00 | 0.02 | 0.07 | 0.06 | 0.07 | 0.06 | 500 |
| 10 | 5 | 500 | 500 | 0.5 | 0.2 | 1.5 | 2 | 2 | 83.0 | 82.8 | 83.0 | 82.8 | 0.00 | 0.03 | 0.00 | 0.02 | 0.07 | 0.06 | 0.07 | 0.06 | 500 |
| 10 | 5 | 500 | 500 | 0.5 | 0.2 | 2.5 | 0.5 | 1 | 82.0 | 82.0 | 82.4 | 82.0 | 0.00 | 0.03 | 0.00 | 0.03 | 0.07 | 0.06 | 0.07 | 0.06 | 500 |
| 10 | 5 | 500 | 500 | 0.5 | 0.2 | 2.5 | 0.5 | 2 | 86.4 | 86.4 | 86.4 | 86.4 | 0.00 | 0.03 | 0.00 | 0.03 | 0.07 | 0.06 | 0.07 | 0.06 | 500 |
| 10 | 5 | 500 | 500 | 0.5 | 0.5 | 1.5 | 2 | 1 | 100.0 | 100.0 | 100.0 | 100.0 | 0.00 | 0.05 | 0.00 | 0.05 | 0.07 | 0.06 | 0.07 | 0.06 | 500 |
| 10 | 5 | 500 | 500 | 0.5 | 0.5 | 1.5 | 2 | 2 | 100.0 | 100.0 | 100.0 | 100.0 | 0.00 | 0.05 | 0.00 | 0.05 | 0.07 | 0.06 | 0.07 | 0.06 | 500 |
| 10 | 5 | 500 | 500 | 0.5 | 0.5 | 2.5 | 0.5 | 1 | 100.0 | 100.0 | 100.0 | 100.0 | 0.00 | 0.06 | 0.00 | 0.06 | 0.07 | 0.06 | 0.07 | 0.06 | 500 |
| 10 | 5 | 500 | 500 | 0.5 | 0.5 | 2.5 | 0.5 | 2 | 100.0 | 100.0 | 100.0 | 100.0 | 0.00 | 0.06 | 0.00 | 0.06 | 0.07 | 0.06 | 0.07 | 0.06 | 500 |
| 10 | 5 | 500 | 500 | 2 | 0 | 1.5 | 2 | 1 | 5.6 | 5.6 | 5.6 | 5.6 | 0.00 | 0.00 | 0.00 | 0.00 | 0.07 | 0.06 | 0.07 | 0.06 | 500 |
| 10 | 5 | 500 | 500 | 2 | 0 | 1.5 | 2 | 2 | 5.6 | 5.6 | 5.6 | 5.6 | 0.00 | 0.00 | 0.00 | 0.00 | 0.07 | 0.06 | 0.07 | 0.06 | 500 |
| 10 | 5 | 500 | 500 | 2 | 0 | 2.5 | 0.5 | 1 | 4.4 | 4.4 | 4.2 | 4.2 | 0.00 | 0.00 | 0.00 | 0.00 | 0.07 | 0.06 | 0.07 | 0.06 | 500 |
| 10 | 5 | 500 | 500 | 2 | 0 | 2.5 | 0.5 | 2 | 3.8 | 3.8 | 3.8 | 3.8 | 0.00 | 0.00 | 0.00 | 0.00 | 0.07 | 0.06 | 0.07 | 0.06 | 500 |
| 10 | 5 | 500 | 500 | 2 | 0.2 | 1.5 | 2 | 1 | 81.2 | 81.0 | 81.4 | 81.0 | 0.00 | 0.04 | 0.00 | 0.04 | 0.07 | 0.05 | 0.07 | 0.05 | 500 |
| 10 | 5 | 500 | 500 | 2 | 0.2 | 1.5 | 2 | 2 | 81.2 | 81.0 | 81.4 | 81.0 | 0.00 | 0.04 | 0.00 | 0.04 | 0.07 | 0.05 | 0.07 | 0.05 | 500 |
| 10 | 5 | 500 | 500 | 2 | 0.2 | 2.5 | 0.5 | 1 | 80.0 | 80.0 | 80.0 | 80.0 | 0.00 | 0.04 | 0.00 | 0.04 | 0.07 | 0.06 | 0.07 | 0.06 | 500 |
| 10 | 5 | 500 | 500 | 2 | 0.2 | 2.5 | 0.5 | 2 | 80.8 | 80.6 | 80.8 | 80.6 | 0.00 | 0.04 | 0.00 | 0.04 | 0.07 | 0.06 | 0.07 | 0.06 | 500 |
| 10 | 5 | 500 | 500 | 2 | 0.5 | 1.5 | 2 | 1 | 100.0 | 100.0 | 100.0 | 100.0 | 0.00 | 0.10 | 0.01 | 0.10 | 0.07 | 0.06 | 0.07 | 0.06 | 500 |
| 10 | 5 | 500 | 500 | 2 | 0.5 | 1.5 | 2 | 2 | 100.0 | 100.0 | 100.0 | 100.0 | 0.00 | 0.10 | 0.00 | 0.10 | 0.07 | 0.06 | 0.07 | 0.06 | 500 |
| 10 | 5 | 500 | 500 | 2 | 0.5 | 2.5 | 0.5 | 1 | 100.0 | 100.0 | 100.0 | 100.0 | 0.00 | 0.09 | 0.00 | 0.09 | 0.07 | 0.06 | 0.07 | 0.06 | 500 |
| 10 | 5 | 500 | 500 | 2 | 0.5 | 2.5 | 0.5 | 2 | 100.0 | 100.0 | 100.0 | 100.0 | 0.00 | 0.09 | 0.00 | 0.09 | 0.07 | 0.06 | 0.07 | 0.06 | 500 |
